# Kumada–Tamao–Corriu Type Reaction of Aromatic Bromo- and Iodoamines with Grignard Reagents

**DOI:** 10.1021/acs.joc.3c01553

**Published:** 2023-11-20

**Authors:** Alicja A. Zielińska, Piotr Trzaska, Marcin Budny, Mariusz J. Bosiak

**Affiliations:** † Department of Organic Chemistry, Faculty of Chemistry, Nicolaus Copernicus University in Toruń, 7 Gagarin Street, Toruń 87-100, Poland; ‡ Doctoral School of Exact and Natural Sciences “Academia Scientiarum Thoruniensis”, Nicolaus Copernicus University in Toruń, 5 Grudziądzka Street, Toruń 87-100, Poland; § Noctiluca SA, 7/41B Gagarina Street, Toruń 87-100, Poland; ∥ Synthex Technologies Sp. z o.o., 7/134B Gagarina Street, Toruń 87-100, Poland

## Abstract

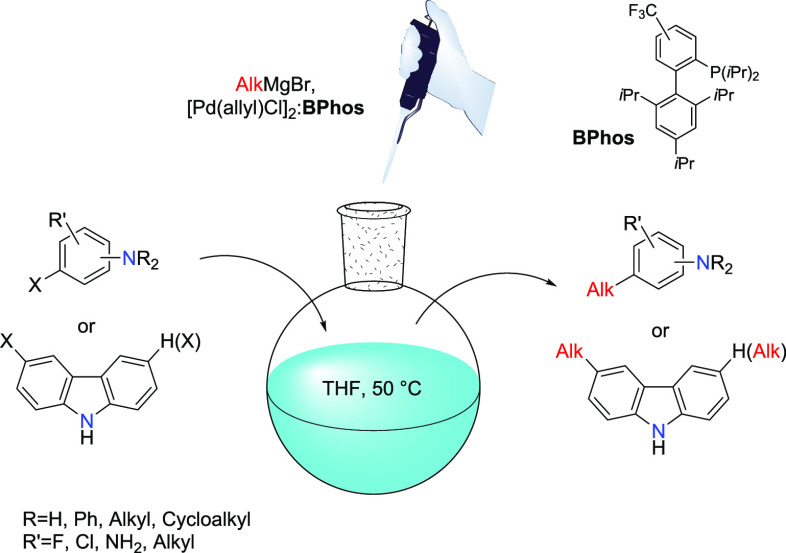

The first example of the Kumada–Tamao–Corriu type reaction of unprotected bromoanilines with Grignard reagents is described. The method uses a palladium source and a newly designed Buchwald-type ligand as the catalytic system. Secondary and tertiary bromo- and iodoamines were also successfully coupled to alkyl Grignard reagents. The products of the competitive β-hydride elimination reaction were successfully reduced using a highly efficient electron-deficient phosphine ligand (**BPhos**). Mechanistic considerations allowed us to establish that the less electron-rich phosphine ligands stabilize the transition state much better than the electron-rich ones; hence, they increase the reaction yield and reduce the amount of β-hydride elimination products. The developed method proved to be tolerant of many functional groups and can be applied to many different aromatic bromo- and iodoamines. Multigram synthesis of *p*-toluidine from 4-bromoaniline was achieved with a palladium catalyst loading of only 0.03 mol%.

## Introduction

Direct introduction of an alkyl group to an aromatic ring of primary aromatic amines remains a challenging transformation. Electrophilic Friedel–Crafts-type alkylation reactions are of limited scope due to the deactivation of the Lewis acid by an unprotected NH_2_ group and/or competitive alkylation reactions on the nitrogen atom. However, few synthetic methods were reported, including catalytic alkylations with organozinc compounds,^1−5^ and a few other catalytic methods^6−10^ or reactions utilizing protonated anilines.^11,12^ The C(sp^2^)–C(sp^3^) catalytic coupling reactions between aromatic halides and aliphatic organometallics have attracted significant attention in recent years. These types of C–C coupling reactions constitute an efficient tool for rapid and predictive increase of molecular complexity of aromatic compounds. The advantage of these reactions is the availability of the starting materials, organometallic reagents (i.e., Zn, B), catalysts (Pd, Ni, Fe), and phosphine ligands.^2,3,13−19^ Importantly, the newly formed C–C bond is introduced with a high degree of regioselectivity, making this method suitable for late-stage modification of complex molecules as a large-scale synthesis of intermediates. However, if organometallics more complex than methyl derivatives are used, β-hydride elimination takes place as a side reaction, resulting in an overall decrease in yield.

The utilization of the Grignard reagents (the Kumada–Tamao–Corriu reaction) in such couplings is particularly attractive due to their commercial availability and easy synthetic accessibility.^20^ This article reports the coupling of the unprotected iodo- and bromosubstituted aromatic amines with simple alkylmagnesium bromides under mild conditions. We also report a highly efficient Buchwald-type phosphine ligand that allows for the reduction of β-hydride elimination side reactions.

## Results and Discussion

Our work commenced with screening commercially available phosphine ligands for coupling 4-iodooaniline (**1a**) and 4-bromoaniline (**1b**) with methylmagnesium bromide and *n*-butylmagnesium bromide. The reactions were performed by the addition of phosphine ligand and a palladium source mixture to the vial or a Schlenk tube containing the starting material and the Grignard reagent in anhydrous THF under inert gas. After the initial screening, Pd_2_(dba)_3_ and phosphine ligand in a 1:3 molar ratio were used as the catalytic system for reactions with MeMgBr. In the case of *n*-BuMgBr, [Pd(allyl)Cl]_2_ proved to be more efficient (see the Supporting Information for details). In all experiments, 3-fold excess of the Grignard reagent was used as we assumed that at least 1 equiv of this reagent is consumed for the deprotonation of the free NH_2_ group. Indeed, the addition of the Grignard reagent is an exothermic process proceeding with a gas release that results in the warming up of the reaction mixture, which in turn leads to the acceleration of the process and completion of the reaction within minutes. Although this phenomenon may be considered beneficial, it generates difficulties in comparing individual catalytic systems. Therefore, we decided to add the Grignard reagents at 0 °C, then the reaction mixtures were allowed to reach the ambient temperature for 10 min, the catalytic system was then added, and the time required to achieve reaction completion was determined by GC-MS.

**Scheme 1:**
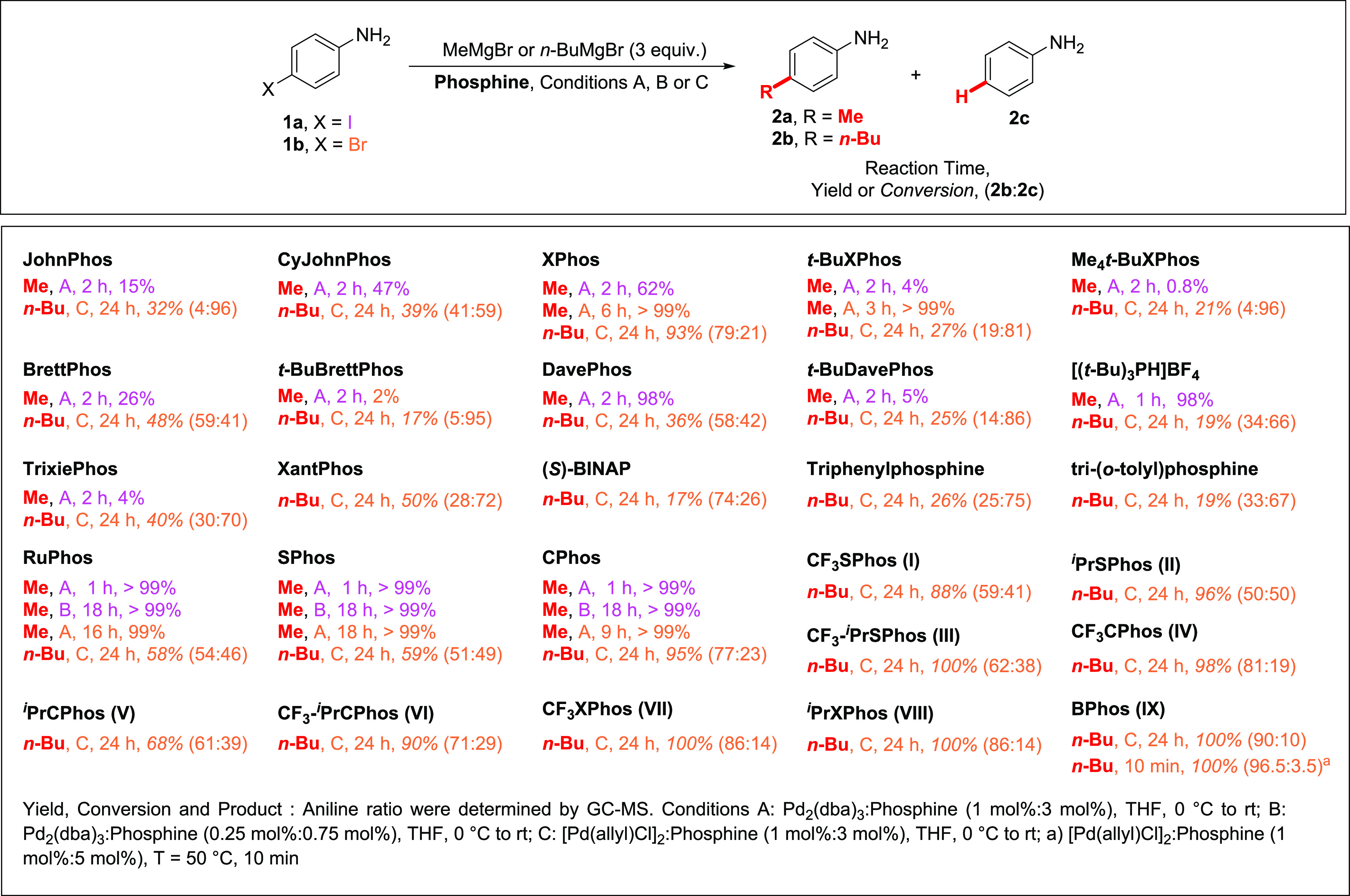
Screening of Suitable Phosphine Ligands for the Coupling of **1a**/**2a** with Grignard Reagents

Commercially available Buchwald ligands (see Supporting Information for structures), containing *t*-butyl groups on the phosphorus atom (JohnPhos, *t*-BuXPhos, *t*-BuBrettPhos, *t*-BuDavePhos, Me_4_*t*-BuXPhos, and TrixiePhos) gave poor results in the coupling of MeMgBr with **1a** (Scheme 1). This is in sharp contrast to the cyclohexyl substituted phosphines (CyJohnPhos, BrettPhos, DavePhos, CPhos, RuPhos, and SPhos), which gave much higher yields of *p*-toluidine than their *t*-butyl analogs. In the case of CPhos, RuPhos, and SPhos, the reaction was complete in less than 1 h, and after a 4-fold reduction in the catalyst load, the reaction came to an end within 18 h.

Encouraged by these results, we found that also the 4-bromoaniline (**1b**) can be transformed into *p*-toluidine with XPhos, *t*-BuXPhos, SPhos, and CPhos ligands, although longer reaction times were required in most cases. It should be noted that the best result was achieved using *t*-BuXPhos, a ligand that was inefficient in the coupling of **1a**.

**Figure 1:**
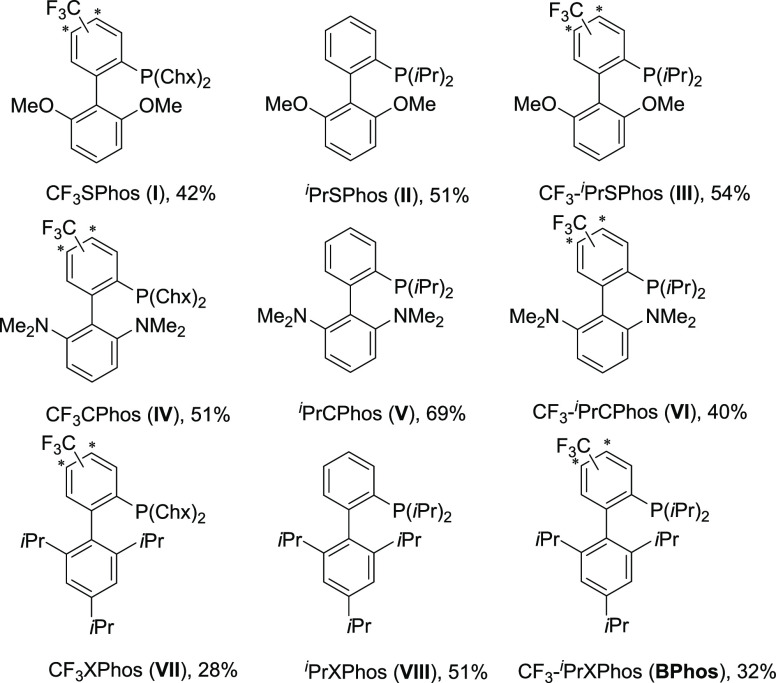
Newly synthesized phosphine ligands

Further tests, using *n*-BuMgBr instead of MeMgBr, revealed that Grignard reagents of longer primary alkyl chain give worse results in the coupling with **1b**, and the significant contribution of the β-hydride elimination product **2c** was observed in these cases. The screening reactions were performed for 24 h, and it was observed that again phosphines containing *t*-butyl groups proved to be less effective than those containing cyclohexyl groups. Both groups gave large amounts of aniline side product, and only for XPhos, CPhos, SPhos, and RuPhos, conversions were higher than 50%. A comparison of ligands that differ only in the substituents on the phosphine-containing ring (*t*-BuXPhos vs *t*-BuBrettPhos vs Me_4_*t*-BuXPhos or XPhos vs BrettPhos) shows that electron-donating substituents on the ring slow the reaction and increase the amount of **2c**. This observation raised the question of whether electron-withdrawing substituents present in phosphine ligands may accelerate the reaction and reduce the β-hydride elimination products. To address this question, we designed and synthesized the CF_3_-substituted analogs of XPhos, CPhos, and SPhos. Moreover, *P*-isopropyl derivatives of those ligands were also synthesized to check if reduced steric hindrance on the phosphorus atom alters reactivity and contribution of β-hydride elimination in the overall process (see Figure 1 for the structures of newly synthesized phosphine ligands). All newly synthesized compounds were obtained in one-pot syntheses, starting from the corresponding bromobenzenes or 2-bromobiphenyls, by metalation and reaction with chlorophosphines (for synthetic details, see Supporting Information).

For analogs of SPhos (**I**, **II**, **III**) and CPhos (**IV**, **V**, **VI**) ligand series, some improvement both in conversion degree and limiting β-hydride elimination contribution was observed (Scheme 1). All derivatives of the XPhos ligand series (**VIII**, **VII**, **IX**) gave very good results, and for **IX** (**BPhos**) the complete conversion of **1b** was furnished within 24 h with 90:10 selectivity of **2b** synthesis. Further improvement was achieved by increasing the amount of **BPhos** to 5 mol% and temperature to 50 °C, which allowed us to obtain **2b** with 96.5:3.5 selectivity and complete conversion after 10 min.

**Scheme 2:**
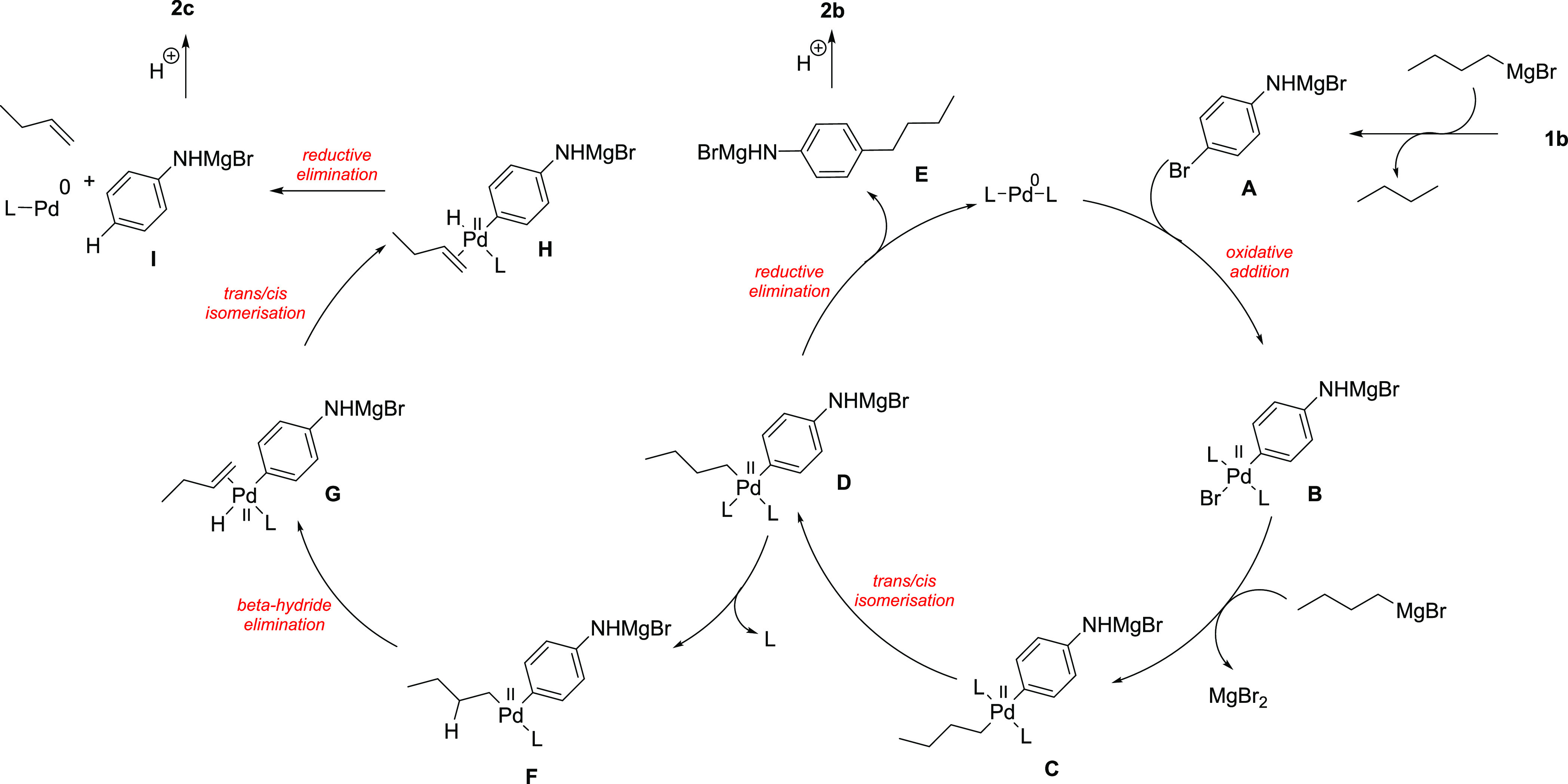
Plausible Mechanism of the Coupling of **1a** with *n*-BuMgBr

The plausible mechanism of the reaction is presented in Scheme 2. First, the amino group in **1b** is deprotonated by basic Grignard reagent, *n*-BuMgBr, to form the corresponding adduct **A**, which undergoes oxidative addition with Pd(0)L_2_ catalyst, pregenerated from the phosphine ligand and palladium source. Thus, square Pd(II) complex **B** is formed which is later transmetalated by another molecule of the Grignard reagent. *Trans*/*cis*-isomerization of **C** provides **D**, which may undergo reductive elimination forming alkylarene **E** and after aqueous workup, amine **2b** is obtained. Intermediate **D** can lose one of the phosphine ligands to form **F**, which is suitable for the β-hydride elimination process. Thus, the π-complex **G** is furnished, which isomerizes to **H**. Then, another reductive elimination takes place, providing **I**, 1-butene, and palladium species. Intermediate **I** gives, after protonation, aniline (**2c**).

The effect of CF_3_ and isopropyl substituents present in ligand molecules on the reaction outcome can be explained in multiple ways, but the rate-limiting factor of the overall reaction seems to be the reductive elimination, which explains the high content of aniline, a side product of β-hydride elimination. The inefficiency of most Buchwald phosphines palladium complexes may be attributed to their easy dissociation from the palladium center, which promotes β-hydride elimination by forming coordinatively unsaturated species.^21−24^

As stated, the introduction of electron-donating substituents on the phosphine-containing ring slows the reaction and increases the amount of aniline formed. These electron-rich ligands, known to promote the coupling of weaker nucleophiles with aryl halides,^25,26^ seem to fail in the case of strong carbon-based nucleophiles such as Grignard reagents. This may be because in the intermediate **D** electron-rich phenylene ring from aniline and alkynyl nucleophile increases electron density on the palladium and thus make it less prone to binding phosphine ligands which more easily dissociate from the reaction center leading to intermediate **F**. On the other hand, ligands bearing electron-deficient substituents bind more strongly to the negatively charged palladium. As a result, intermediate **D** is stable enough to follow the reductive elimination pathway and form **E**, rather than losing ligand and initiating the β-hydride elimination sequence.

**Scheme 3:**
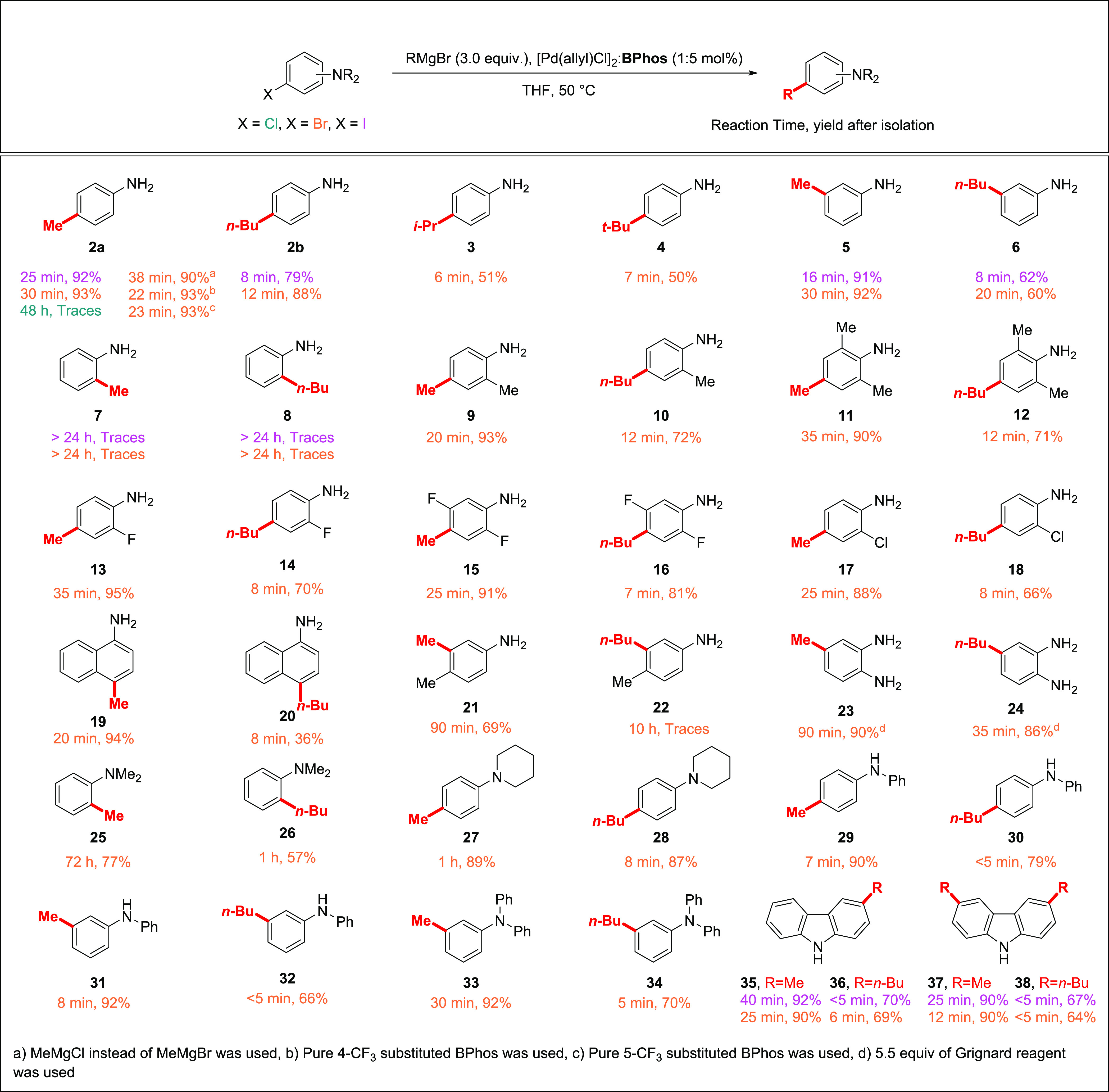
Scope of the Reaction

With the modified conditions in hand, we investigated the scope of the reaction (Scheme 3). The reaction of MeMgBr with anilines substituted with iodine at positions 3 and 4, relative to the NH_2_ group, proceeded smoothly within a few minutes and for corresponding bromoanilines in most cases within up to 35 min in yields exceeding 90%. In all these examples, only traces (less than 0.2%) of reduction products (e.g., **2c** for of 3-, and 4-iodo- and bromoanilines) were detected. Notably, MeMgCl is only slightly less reactive in comparison to MeMgBr. Isopropyl and *tert*-butyl groups were also introduced in this manner; however, β-hydride elimination had a significant contribution to overall processes, and corresponding products **3** and **4** were obtained in a rather moderate yield. Other halogens than I and Br were unreactive under these conditions, making possible the selective transformation of C(sp^2^)-Br(I) in the presence of C(sp^2^)-F and C(sp^2^)-Cl (compounds **13**, **14**, **15**, **17**, and **18**). Alkyl and additional amino substituents in the benzene ring were mostly well tolerated (compounds **9**–**12**, **21**, **23**, and **24**).

**Scheme 4:**
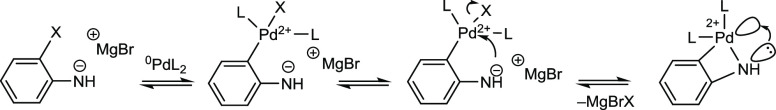
Deactivation of the Catalyst

The *ortho* effect for amino substituents in position 2 relative to the reaction center was observable. When syntheses of **7** and **8** were attempted from brominated and iodinated precursors, only traces of products were detected. One of the reasons for the lack of reactivity of 2-substituted anilines can be the steric hindrance caused by the bromomagnesium salt NHMgBr or N(MgBr)_2_, formed in the reaction of the NH_2_ group with the Grignard reagent, at position 2 of the aromatic ring. The other reason can be a strong stabilization of palladium by some Pd–N double bond character due to p donation of electron lone pair of the amido group to an “empty” *d* orbital of palladium (Scheme 4) as suggested by Moncho et al.^27^

The latter mechanism is supported by the fact that having NMe_2_ in position 2 to the reaction center, the reaction occurs albeit longer reaction times are required (**25** and **26**). Carbazole derivatives can also be synthesized in this reaction with good to excellent yields (**35**–**38**).

**Scheme 5:**
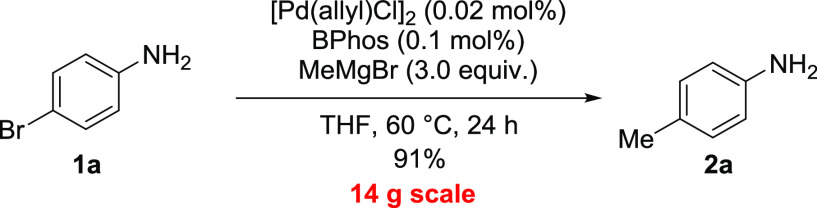
Large-Scale Coupling

Finally, the coupling of **1b** and MeMgBr on a 14 g scale was attempted in the presence of only 0.03 mol% [Pd(allyl)Cl]_2_ and 0.15 mol% **BPhos** in anhydrous THF under inert gas at 60 °C (Scheme 5). Under these conditions, **2b** was obtained in 91% yield as an analytically pure sample after distillation and crystallization.

## Conclusions

In conclusion, a smooth and rapid method for the catalytic coupling of bromo- and iodoanilines, secondary and tertiary aromatic amines, and halogenated carbazoles with alkyl Grignard reagents was developed. The method uses a palladium source and the newly designed CF_3_ substituted Buchwald-type diisopropylphosphine ligand (**BPhos**), and it is particularly useful for coupling methylmagnesium bromide or the Grignard reagents derived from primary alkyl halides. The usage of **BPhos** as a ligand significantly improves the reaction yields and helps to reduce the undesired β-hydride elimination products. Multigram synthesis of *p*-toluidine from 4-bromoaniline was achieved with a palladium catalyst loading of only 0.03 mol%.

## Experimental Section

### General Experimental Methods

Experiments with air- and moisture-sensitive materials were carried out under an argon atmosphere. Glassware was oven-dried for several hours, assembled hot, and cooled in a stream of argon. Silica gel 60, Merck 230–400 mesh, was used for preparative column flash chromatography. Analytical TLC was performed using Merck TLC Silica gel 60 F254 0.2 mm plates. Allylpalladium(II) chloride dimer, Pd_2_(dba)_3_, other palladium catalysts, JohnPhos, *t*-BuXPhos, *t*-BuBrettPhos, *t*-BuDavePhos, Me_4_*t*-BuXPhos, TrixiePhos, CyJohnPhos, XPhos, BrettPhos, DavePhos, CPhos, RuPhos, SPhos, [(*t*-Bu)_3_PH]BF_4_, S-BINAP, tri(o-tolyl)phosphine, XantPhos, PPh_3_, methylmagnesium halides, *n*-butylmagnesium bromide, iodo- and bromoamines, and other commercially available reagents were purchased from Sigma-Aldrich, Merck, TCI, Enamine, or Fluorochem and were used without further purification. Solvents were purchased from Avantor, VWR, and Sigma-Aldrich. Toluene and THF were distilled from sodium benzophenone ketyl before use. *n*-Hexane was dried with molecular sieves and used without further purification. ^1^H and ^13^C NMR spectra were recorded on Bruker Advance III 400 MHz or Bruker Avance III 700 MHz instruments at ambient temperature. Chemical shifts are reported in parts per million (d scale), and coupling constants (*J* values) are given in Hertz. IR spectra were recorded on a Perkin-Elmer FT-IR Spectrometer Spectrum Two. GCMS analyses were performed on a Shimadzu GCMS-TQ8040 system via autoinjection and detector response was calibrated on the substrate and product standards. Melting points were determined with BarnsteadeThermolyne Mel-Temp II apparatus in open capillaries and are uncorrected. Elemental analyses were performed at Elementary Analysensysteme GmbH VarioMACRO CHNanalyzer.

### General Procedure for Alkylation of Bromo- and Iodoamines with Grignard Reagents Using [Pd]:**BPhos** Catalytic System

#### Screenings

Amine (2 mmol, 1.0 equiv) was dissolved in a septum capped vial in degassed anhydrous THF (3 mL, 0.66 mol·L^–1^) and cooled to 0 °C, and the Grignard reagent (6 mmol, 3.0 equiv) was added slowly (note: it foams, the resulting gas must be removed through a septum). The mixture was left for 10 min to achieve room temperature and then degassed by using vacuum/argon cycles (3×), and the [Pd(allyl)Cl]_2_:**BPhos** (0.02 mmol, 0.01 equiv and 0.1 mmol, 0.05 equiv, respectively) catalyst prepared in a separate vial in degassed THF (1.5 mL) was added. The mixture was stirred under argon (bubbler) monitoring the disappearance of the substrate with GCMS, and after completion of the reaction, it was quenched with water, extracted with diethyl ether, and purified by column chromatography.

#### Typical Procedure for Large-Scale Reaction

In a 500 mL pressure flask closed with a septum, 4-bromoaniline (25.8 g, 0.15 mol, 1 equiv) was dissolved under argon in dry, degassed THF (200 mL, 0.75 mol·L^–1^), cooled to 0 °C, and stirred for 5 min. Methylmagnesium bromide (3 M in Et_2_O, 155 mL, 0.465 mmol, 3.1 equiv) was added dropwise, and the resulting gas was removed by a bubbler. The mixture was degassed by using vacuum/argon cycles (3×), and the [Pd(allyl)Cl]_2_:**BPhos** (0.016 g, 0.045 mmol, 0.0003 equiv and 0.104 g, 0.225 mmol, 0.0015 equiv, respectively) catalyst prepared in degassed THF (5 mL) in a separate vial was added. The septum was replaced with a Teflon stopper, and the flask was immersed in an oil bath preheated to 60 °C and stirred for 24 h. It was cooled in an ice bath; the mixture was poured into the crushed ice (ca. 500 mL); Et_2_O (300 mL) was added; and the mixture was filtered. The Et_2_O layer was separated; the water was extracted with Et_2_O (3 × 80 mL); and combined organic layers were washed with brine and dried with anhydrous magnesium sulfate. After the solvent was removed on a rotary evaporator, the crude product was purified by vacuum distillation followed by crystallization from PE to give 14.64 g (91%) of analytically pure *p*-toluidine as white flakes. Mp = 45–46 °C, NMR spectra are the same as in the literature.

##### Synthesis of **BPhos** (**IX**)

In a Schlenk tube, magnesium powder (1.04 g, 43 mmol, 2.1 equiv) was dispersed in dry THF (20 mL, 1.025 mol·L^–1^) and immersed in an oil bath preheated to 80 °C. 2-Bromo-1,3,5-triisopropylbenzene (∼100 mg) was added followed by a few drops of ethylene dibromide. After the reaction has started the rest of 2-bromo-1,3,5-triisopropylbenzene was added dropwise (5.80 g, 20.5 mmol, 1.0 equiv in total). After completion of the Grignard reagent forming confirmed by GCMS analysis (a small sample was quenched with NH_4_Cl_aq_ and extracted with Et_2_O), 1,2-dibromo-4-(trifluoromethyl)benzene (6.38 g, 21 mmol, 1.02 equiv) was added and the reaction was heated at 80 °C to the disappearance of substrates on GC (∼3.5 h). CuCl (1.0 g, 10 mmol, 0.49 equiv) was added at 80 °C, and the temperature was lowered to 60 °C. Chlorodiisopropylphosphine (3.60 g, 20 mmol, 0.98 equiv) was added, and the reaction was stirred at 60 °C for 1 h. It was cooled to RT; 10% Na_2_S_2_O_5_ (20 mL) was added followed by Et_2_O (30 mL); and the layers were separated. The aqueous layer was extracted with Et_2_O (2 × 20 mL), and the combined organic layers were washed several times with saturated aqueous ammonia until the blue color of the copper complex disappeared and then with water and brine and dried with anhydrous magnesium sulfate. After removing the solvent on a rotary evaporator, the crude product was purified by flash chromatography, eluent PE, and crystallized from methanol to give 2.95 g (32% yield) of white crystalline solid as a mixture of two isomers. mp = 79–81 °C. NMR (CDCl_3_, 700 MHz): δ 8.0 (bs, 1H), 7.86 (s, 1H), 7.70 (d, 8.0 Hz, 1H), 7.61 (d, *J* = 7.8 Hz, 1H), 7.50 (bs, 1H), 7.32–7.30 (dd, J = 7.7, 3.5 Hz, 1H), 7.08 (s, 2H), 7.06 (s, 2H), 2.99–2.93 (m, 2H), 2.35–2.28 (m, 6H), 2.08–2.03 (m, 2H), 1.33 (d, *J* = 7.0 Hz, 12H), 1.24–1.21 (m, 12H), 1.16 (dd, *J* = 13.0, 7.0 Hz, 6H), 1.12 (dd, *J* = 11.8, 7.0 Hz, 6H), 1.06 (dd, *J* = 15.7, 7.0 Hz, 6H), δ = 1.03–0.98 (m, 18H). ^13^C{1H} NMR (CDCl_3_, 75.5 MHz,): δ 151.4, 151.0, 148.6, 148.4, 147.7, 145.9, 145.7, 142.0, 139.5, 139.2, 134.8 (q, *J* = 4.4 Hz), 132.5, 131.6, 131.5, 129.9 (q, *J* = 32.7 Hz), 128.8, 128.3, 127.9, 124.4 (q, *J* = 274.8 Hz), 124.3, 124.1 (q, *J* = 271.6 Hz), 122.9, 120.6, 120.6, 34.1, 34.1, 30.8, 30.7, 25.8, 25.7, 24.0, 23.7, 23.6, 23.5, 23.5, 22.5, 21.1, 21.0, 20.8, 20.8, 18.7, 18.6.

Anal. calcd for C_28_H_40_F_3_P: C, 72.38; H, 8.68; found: C, 72.55; H, 8.62. Isomers 4-CF_3_ and 5-CF_3_ were separated by double crystallization from the MeOH:H_2_O mixture (98:2).

##### 4-CF_3_**BPhos** White Crystalline Solid, 0.93 g (10% Yield) ^1^H NMR (CDCl_3_, 700 MHz)

δ 7.99 (bs, 1H), 7.69 (d, *J* = 7.0 Hz, 1H), 7.50 (bs, 1H), 7.07 (s, 2H), 2.96 (sept, *J* = 7.0 Hz, 1H), 2.35–2.29 (m, 4H), 1.33 (d, *J* = 7.0 Hz, 6H), 1.22 (d, *J* = 7.0 Hz, 6H), 1.16 (dd, *J* = 13.0, 7.0 Hz, 6H), 1.06 (dd, *J* = 15.4, 7.0 Hz, 6H), 1.0 (d, *J* = 7.0 Hz, 6H). ^13^C{1H} NMR (CDCl_3_, 75.5 MHz,): δ 148.8, 148.0, 147.6, 145.9, 134.4, 132.6, 130.0, 127.9, 123.1, 122.2, 120.7, 34.1, 30.8, 25.7, 24.0, 23.5, 23.4, 22.5, 20.9, 20.7, 18.7, 18.6. Anal. calcd for C_28_H_40_F_3_P: C, 72.38; H, 8.68; found: C, 72.51; H, 8.65.

##### 5-CF_3_**BPhos** White Crystalline Solid, 1.02 g (13% Yield) ^1^H NMR (CDCl_3_, 700 MHz)

δ 7.99 (s, 1H), 7.64 (d, *J* = 7.0 Hz, 1H), 7.33 (dd, *J* = 7.7, 3.5 Hz, 1H), 7.06 (s, 2H), 2.97 (sept, *J* = 7.0 Hz, 1H), 2.32 (sept, *J* = 7.0 Hz, 2H), 2.15–2.09 (m, 2H), 1.33 (d, *J* = 7.0, 6H), 1.25 (d, *J* = 7.0, 6H), 1.13 (dd, *J* = 12.2, 7.0 Hz, 6H), 1.03 (dd, *J* = 15.0, 7.0 Hz, 6H), 0.99 (d, *J* = 7.0 Hz, 6H). ^13^C{1H} NMR (75.5 MHz, CDCl_3_): δ = 18.6, 18.7, 20.6, 20.7, 22.5, 23.5, 23.6, 24.0, 25.9, 30.8, 34.1, 120.7, 125.0, 125.1, 128.7, 128.8, 131.8, 131.9, 134.3, 145.8, 146.1, 148.9. Anal. calcd for C_28_H_40_F_3_P: C, 72.38; H, 8.68; found: C, 72.45; H, 8.67. IR: (neat) ṽ 3048, 2914, 2856, 2210, 1902, 1694, 1589, 1508, 1475, 1438, 1279, 1181, 1164, 1145, 1101, 1037, 947, 913, 827, 813, 782, 751, 694, 578, 531, 512, 494, 455, 413 cm^–1^.

##### 2-Bromo-2′,6′-dimethoxy-4-(trifluoromethyl)-1,1′-biphenyl and 2-bromo-2′,6′-dimethoxy-5-(trifluoromethyl)-1,1′-biphenyl

1,3-Dimethoxybenzene (5.80 g, 42 mmol, 1.0 equiv) was placed in a Schlenk tube, and dry THF (40 mL, 1.05 mol·L^–1^) was added. The mixture was cooled to 0 °C, and *n*-BuLi (2.5 M in hexane, 18.4 mL, 46 mmol, 1.1 equiv) was added dropwise. The cooling bath was removed, and the reaction mixture was stirred at RT for 4 h. The Schlenk tube was immersed in the water bath (18 °C), and 2-bromo-1-chloro-4-(trifluoromethyl) benzene (11.91 g, 46 mmol, 1.1 equiv) was added dropwise over 10 min. The water bath was removed, and the mixture was stirred at RT for an additional 30 min, then cooled to 0 °C, and poured into the water (80 mL); diethyl ether (60 mL) was added; and the layers were separated. The aqueous layer was washed with diethyl ether (35 mL); combined organic layers were washed with brine, dried with anhydrous magnesium sulfate, and concentrated. The crude product was purified by flash chromatography, PE → PE:AcOEt (95:5) to give 8.19 g (54% yield) of the desired product as a colorless liquid. bp = 115–120 °C/0.1 mmHg. Mp = 29–30 °C^1^H NMR (CDCl_3_, 400 MHz): δ 7.94–7.93 (m, 1H), 7.78 (d, *J* = 8.5 Hz, 1H), 7.61 (d, *J* = 8.5 Hz, 1H), 7.51 (m, 1H), 7.46–7.43 (m, 1H), 7.41–7.36 (m, 3H), 6.69–6.66 (m, 4H), 3.76–3.75 (m, 12H). ^13^C{1H} NMR (CDCl_3_, 175 MHz): δ 157.6, 157.5, 140.4, 137.2, 132.9, 132.8, 130. 62 (q, *J* = 33.3 Hz), 130.1, 129.4, 129.4 (q, *J* = 3.5 Hz), 125.6, 125.2, 125.2,124.1 (q, *J* = 271.1 Hz), 123.8, 123.8, 123.5 (q, *J* = 273.5 Hz), 117.6, 117.4, 104.0, 104.0, 55.9. Anal. calcd for C_15_H_12_BrF_3_O_2_: C, 49.89; H, 3.35; found: C, 50.11; H, 3.31.

##### CF_3_SPhos·HBF_4_ (**I**)

The 1:1 mixture of 2-bromo-2′,6′-dimethoxy(trifluoromethyl)-1,1′-biphenyls from previous step (0.722 g, 2 mmol, 1.0 equiv) was placed in a Schlenk tube, and dry THF (15 mL, 0.13 mol·L^–1^) was added. The mixture was cooled to −78 °C, *n*-BuLi (2.5 M in hexane, 1 mL, 2.5 mmol, 1.25 equiv) was added dropwise, and it was stirred for 10 min. Chlorodicyclohexylphosphine (0.699 g, 11 mmol, 5.5 equiv) was added dropwise over 10 min, and the mixture was stirred at −78 °C for an additional 20 min. The reaction was quenched with MeOH (2 mL), concentrated, and purified by flash chromatography, PE → PE:AcOEt (96:4), to give 0.76 g of white solid. As the NMR spectra were difficult to analyze with broad signals, the phosphine product was converted to its HBF_4_ salt by dissolving in Et_2_O (10 mL), adding 48%HBF_4_ aq until the precipitate, formed when the first drops of the acid were added, disappeared (ca. 2 mL), and was diluted with 0.01 M HBF_4_ aq (50 mL). The thus-formed precipitate was filtered, washed with a small amount of deionized water and Et_2_O, and dried in air to give 0.40 g (42% yield) of white solid. It was used for further reactions in the HBF_4_ salt form. Mp = 177–179 °C. ^1^H NMR (CDCl_3_, 700 MHz): δ 8.02–7.98 (m, 2H), 7.88–7.86 (m, 1H), 7.82–7.81 (m, 1H), 7.65–7.64 (m, 1H), 7.59–7.58 (m, 1H), 7.52–7.48 (m, 2H), 6.82–6.80 (m, 0.5H), 6.75–6.70 (m, 4H), 6.49–6.47 (m, 0.5H), 6.09–6.06 (m, 0.5H), 5.78–5.76 (m, 0.5H), 3.74 (s, 6H), 3.73 (s, 6H), 2.79–2.74 (m, 2H), 2.69–2.62 (m, 2H), 1.87–1.76 (m, 16H), 1.74–1.69 (m, 4H), 1.47–1.31 (m, 16H), 1.23–1.17 (m, 4H). ^13^C{1H} NMR (CDCl_3_, 100 MHz): δ 156.6, 156.4, 135.1, 134.9, 132.8, 132.6, 130.8, 130.7, 104.7, 104.6, 55.7, 55.7, 29.5, 29.4, 28.9, 28.8, 26.5, 26.4, 26.4, 26.2, 26.2, 26.1, 25.9, 25.8, 25.8, 25.0, 25.0. Anal. calcd for free phosphine C_27_H_34_F_3_O_2_P: C, 67.77; H, 7.16; found: C, 67.58; H, 7.15.

##### ^i^PrSPhos·HBF_4_ (**II**)

2′-Bromo-2,6-dimethoxy-1,1′-biphenyl (0.584 g, 2 mmol, 1.0 equiv) was placed in a Schlenk tube, and dry THF (25 mL, 0.08 mol·L^–1^) was added. The mixture was cooled to −78 °C, *n*-BuLi (2.5 M in hexane, 1.05 mL, 2.625 mmol) was added dropwise, and it was stirred for 30 min. Chlorodiisopropylphosphane (0.352 g, 2.3 mmol, 1.15 equiv) was added dropwise over 10 min. The cooling bath was removed, and after heating to −10 °C, the mixture was filtered through a 3 cm pad of silica gel. The filtrate was concentrated to obtain 0.63 g of white solid. It was dissolved in Et_2_O (12 mL); 48%HBF_4_ aq was added until the precipitate, formed when the first drops of the acid were added, disappeared (ca. 2 mL), and diluted with 0.01 M HBF_4_ aq. (50 mL), and it was stirred for 5 min. The precipitate was filtered, washed with Et_2_O (2 × 3 mL), and dried in air to give 0.43 g (51% yield) of white solid. Mp = 176–178 °C. ^1^H NMR (CDCl_3_, 700 MHz): δ 7.66 (t, *J* = 9.0 Hz, 1H), 7.63 (t, *J* = 7.7 Hz, 1H), 7.46 (t, *J* = 8.5 Hz, 1H), 7.43–7.42 (m, 1H), 6.72 (d, *J* = 8.5 Hz, 2H), 6.50–6.47 (m, 0.5H), 5.79–5.77 (m, 0.5H), 3.72 (s, 6H), 2.95–2.91 (m, 2H), 1.28–1.24 (m, 12H). ^13^C{1H} NMR (CDCl_3_, 75 MHz): δ 156.7, 134.4, 134.4, 134.1, 133.9, 132.4, 132.3, 132.0, 128.5, 128.3, 104.6, 55.5, 21.0, 20.5, 16.8, 16.3, 16.2. Anal. calcd for free phosphine C_20_H_27_O_2_P: C, 72.70; H, 8.24; found: C,73.00; H, 8.24.

##### CF_3_^i^PrSPhos·HBF_4_ (**III**)

The 1:1 mixture of 2-bromo-2′,6′-dimethoxy(trifluoromethyl)-1,1′-biphenyls (0.722 g, 2 mmol, 1.0 equiv) was placed in a Schlenk tube, and dry THF (5 mL, 0.4 mol·L^–1^) was added. The mixture was cooled to −78 °C, *n*-BuLi (2.5 M in hexane, 1 mL, 2.5 mmol, 1.25 equiv) was added dropwise, and it was stirred for 20 min. It was heated to 0 °C and cooled again to −78 °C. Chlorodiisopropylphosphine (0.383 g, 2.5 mmol, 1.25 equiv) was added dropwise, and the mixture was stirred at −78 °C for 20 min. It was heated to 0 °C, cooled again to −78 °C, and quenched with HBF_4_·Et_2_O (0.50 g, 3.1 mmol, 1.55 equiv). It was stirred for 10 min, and the cooling bath was removed. Solvents were removed in a stream of argon; 48% HBF_4_ aq (5 mL) was added to obtain a clear solution, which was washed with Et_2_O (2 × 5 mL), diluted with deionized water, and stirred overnight. The white precipitate was filtered, washed with deionized water (3 × 5 mL), and dried under reduced pressure to obtain the desired product. A total of 0.525 g (54% yield) of white solid was obtained. Mp = 160–162 °C. ^1^H NMR (CDCl_3_, 700 MHz): δ 8.03 (d, *J* = 8.6 Hz, 1H), 7.99 (t, *J* = 9.3 Hz, 1H), 7.90 (d, *J* = 8.0 Hz, 1H), 7.84 (d, *J* = 11.5 Hz, 1H), 7.72–7.71 (m, 1H), 7.66–7.64 (m, 1H), 7.56–7.52 (m, 2H), 6.99–6.97 (m, 0.5H), 6.79–6.76 (m, 4H), 6.71–6.69 (m, 0.5H), 6.25–6.24 (m, 0.5H), 6.00–5.98 (m, 0.5H), 3.77 (s, 6H), 3.77 (s, 6H), 3.05–3.01 (m, 2H), 2.97–2.93 (m, 2H), 1.34–1.28 (m, 24H). ^13^C{1H} NMR (acetone-*d*_6_, 100 MHz): δ 156.9, 156.8, 143.2, 135.0, 134.2, 134.1, 132.3, 131.2, 130.2, 129.5, 125.1, 104.6, 55.5, 20.5, 20.1, 16.0, 15.6. Anal. calcd for free phosphine C_21_H_26_F_3_O_2_P: C, 63.31; H, 6.58; found: C, 63.48; H, 6.55.

##### 2′-Bromo-*N*^2^,*N*^2^,*N*^6^,*N*^6^-tetramethyl-4′-(trifluoromethyl)-[1,1′-biphenyl]-2,6-diamine and 2′-Bromo-*N*^2^,*N*^2^,*N*^6^,*N*^6^-tetramethyl-5′-(trifluoromethyl)-[1,1′-biphenyl]-2,6-diamine

*N*^1^,*N*^1^,*N*^3^,*N*^3^-Tetramethylbenzene-1,3-diamine (3.28 g, 20 mmol, 1.0 equiv) was placed in a Schlenk tube, and dry *n*-hexane (30 mL, 0.67 mol·L^–1^) was added. The n-BuLi (2.5 M in hexane, 8.4 mL, 21 mmol, 1.05 equiv) was added dropwise at RT, and the mixture was heated to 65 °C and stirred for 1 h. It was cooled to 0 °C, 2-bromo-1-chloro-4-(trifluoromethyl)benzene (5.12 g, 20 mmol, 1.0 equiv) was added, heated again to 65 °C, and stirred overnight. It was quenched with water (30 mL); Et_2_O was added (35 mL); and the layers were separated. The aqueous layer was extracted with Et_2_O (2 × 30 mL); combined organic fractions were washed with brine, dried with anhydrous MgSO_4_, and concentrated. The crude product was purified by flash chromatography (PE:AcOEt 98:2) and crystallized from MeOH:H_2_O 9:1 to obtain 3.8 g (49% yield) of a white solid. Mp = 74–76^1^H NMR (CDCl_3_, 700 MHz): δ 7.94–7.93 (m, 1H), 7.79 (d, J = 8.7 Hz, 1H), 7.62–7.61 (m, 2H), 7.47 (d, J = 7.9 Hz, 1H), 7.41–7.40 (m, 1H), 7.37–7.34 (m, 2H), 6.94–6.92 (m, 4H), 2.45 (m, 24H). ^13^C{1H} NMR (CDCl_3_, 100 MHz): δ 153.2, 133.6, 133.2, 130.3, 130.1, 130.1, 129.6, 129.4, 129.3, 126.4, 125.5, 124.1, 123.6, 123.5 (q, *J* = 270.7 Hz), 122.8, 114.3, 44.0.

Anal. calcd for C_17_H_18_BrF_3_N_2_: C, 52.73; H, 4.69; N, 7.23 found: C, 52.52; H, 4.66; N, 7.18.

##### CF_3_CPhos (**IV**)

The 1:1 mixture of 2′-bromo-*N*^2^,*N*^2^,*N*^6^,*N*^6^-tetramethyl(trifluoromethyl)-[1,1′-biphenyl]-2,6-diamines (0.387 g, 1 mmol, 1.0 equiv) was placed in a Schlenk tube, and dry THF (10 mL, 0.1 mol·L^–1^) was added. The mixture was cooled to −78 °C, *n*-BuLi (2.5 M in hexane, 0.5 mL, 1.25 mmol, 1.25 equiv) was added dropwise, and it was stirred for 20 min. Chlorodicyclohexylphosphine (0.349 g, 1.5 mmol, 1.5 equiv) was added dropwise over 10 min; the mixture was stirred at −78 °C for an additional 5 min and RT for 30 min. Solvents were removed by rotary evaporation; PE was added (80 mL), stirred for 5 min, and filtered. The filtrate was concentrated; methanol was added to the resulting oil; and it was stirred until crystallized. The precipitate was filtered off and dried to give 0.225 g (51% yield) of a white powder. Mp = 122–124. ^1^H NMR (acetone-*d*_6_, 400 MHz): δ 7.88–7.86 (m, 2H), 7.69–7.57 (m, 4H), 7.29 (t, *J* = 8.1 Hz, 2H), 6.92 (d, *J* = 7.9 Hz, 4H), 2.42 (s, 24H), 1.96–1.83 (m, 8H), 1.79–1.70 (m, 4H), 1.68–1.59 (m, 8H), 1.56–1.47 (m, 4H), 1.28–1.05 (m, 20H). ^13^C{1H} NMR (CDCl_3_, 100 MHz): δ 153.4, 153.4, 133.4, 133.3, 132.9, 129.7, 129.1, 124.0, 121.9, 114.5, 114.3, 44.9, 35.1, 34.9, 31.2, 31.0, 29.5, 29.3, 27.8, 27.6, 27.5, 27.4, 26.5.

Anal. calcd for C_29_H_40_F_3_N_2_P: C, 69.03; H, 7.99; N, 5.55; found: C, 69.74; H, 8.02; N, 5.68.

##### 2′-Bromo-*N*^2^,*N*^2^,*N*^6^,*N*^6^-tetramethyl-[1,1′-biphenyl]-2,6-diamine

*N*^1^,*N*^1^,*N*^3^,*N*^3^-Tetramethylbenzene-1,3-diamine (1.12g, 6.8 mmol, 1.0 equiv) was placed in a Schlenk tube, and dry hexane (20 mL, 0.34 mol·L^–1^) was added. The *n*-BuLi (2.5 M in hexane, 3 mL, 7.5 mmol, 1.1 equiv) was added dropwise at RT, and the mixture was heated to 65 °C and stirred for 1 h. It was cooled to 0 °C; 1-bromo-2-chlorobenzene (1.30 g, 6.8 mmol, 1.0 equiv) was added; it was heated again to 65 °C and stirred overnight. It was quenched with water (10 mL), and the layers were separated. The aqueous layer was extracted with Et_2_O (3 × 15 mL); combined organic fractions were washed with water and brine, dried with anhydrous MgSO_4_, and concentrated. The crude product was purified by flash chromatography (PE:AcOEt 98:2) to obtain 1.40 g (64% yield) of a white solid. Mp = 70–72 °C, NMR spectra are the same as in the literature.^28^

##### ^i^PrCPhos·HBF_4_ (**V**)

2′-Bromo-*N*^2^,*N*^2^,*N*^6^,*N*^6^-tetramethyl-[1,1′-biphenyl]-2,6-diamine (0.477 g, 1.5 mmol, 1.0 equiv) was placed in a Schlenk tube, and dry THF (3 mL, 0.5 mol·L^–1^) was added. The mixture was cooled to −78 °C, *n*-BuLi (2.5 M in hexane, 0.60 mL, 1.5 mmol, 1.5 equiv) was added dropwise, and it was stirred for 1 h. Chlorodiisopropylphosphine (0.237 g, 1.55 mmol, 1.03 equiv) was added dropwise, and the mixture was stirred at −78 °C for an additional 30 min. The cooling bath was removed; the mixture was allowed to achieve 0 °C and then cooled again to −78 °C. HBF_4_·Et_2_O (0.486 g, 3 mmol, 2.0 equiv) was added dropwise, and the mixture was stirred at −78 °C for 10 min and RT for 30 min. The precipitate formed was filtered off, washed with THF (3 × 2 mL) and Et_2_O (2 × 3 mL), and dried to obtain 0.69 g of crude product that was crystallized from methanol to give 0.46 g (69% yield) of white solid. Mp = 198–200 °C. ^1^H NMR (MeOD, 400 MHz): δ 7.63–7.60 (m, 1H), 7.34–7.22 (m, 4H), 6.87–6.85 (m, 2H), 2.39 (m, 12H), 2.13–2.05 (m, 2H), 1.12–1.07 (m, 6H), 0.95–0.89 (m, 6H). ^13^C{1H} NMR (MeOD, 100 MHz): δ 153.3, 146.0, 145.7, 137.2, 137.0, 132.9, 132.3, 131.9, 128.2, 127.4, 125.6, 114.0, 43.9, 24.1, 24.00, 20.8, 20.6, 18.4, 18.2. Anal. calcd for free phosphine C_22_H_33_N_2_P: C, 74.12; H, 9.33; found: C, 74.21; H, 9.34.

##### CF_3_^i^PrCPhos·HBF_4_ (**VI**)

The 1:1 mixture of 2′-bromo-*N*^2^,*N*^2^,*N*^6^,*N*^6^-tetramethyl(trifluoromethyl)-[1,1′-biphenyl]-2,6-diamines (0.290 g, 0.75 mmol, 1.0 equiv) was placed in a Schlenk tube, and dry THF (3 mL, 0.25 mol·L^–1^) was added. The mixture was cooled to −78 °C, *n*-BuLi (2.5 M in hexane, 0.33 mL, 0.82 mmol, 1.09 equiv) was added dropwise, and it was stirred for 10 min. Chlorodiisopropylphosphine (0.153 g, 1 mmol, 1.33 equiv) was added dropwise; the mixture was stirred at −78 °C for an additional 10 min and RT for 1.5 h. It was cooled again to −78 °C; HBF_4_·Et_2_O (0.486 g, 3 mmol, 4.0 equiv) was added dropwise followed by Et_2_O (25 mL); and the mixture was stirred at RT until sticky oil crystallized. The precipitate formed was filtered off, crystallized from a small amount of methanol, and dried to obtain 0.155 g (40% yield) of the pure product as a white solid. Mp = 175–177 °C. ^1^H NMR (MeOD, 400 MHz): δ 8.22–8.05 (m, 6H), 7.95–7.93 (m, 1H), 7.76 (t, *J* = 8.5 Hz, 2H), 7.56 (d, *J* = 8.3 Hz, 4H), 2.91 (s, 24H), 2.25 (s, 4H), 1.26–1.07 (m, 24H). ^13^C{1H} NMR (acetone-*d*_6_, 75 MHz): δ 153.4, 146.8, 138.5, 136.7, 136.6, 134.7, 133.3, 131.8, 130.0, 127.7, 123.8, 119.4, 46.3, 22.2, 21.8, 17.4, 16.7. Anal. calcd for free phosphine C_23_H_32_F_3_N_2_P: C, 65.08; H, 7.60; found: C, 64.86; H, 7.55.

##### CF_3_XPhos (**VII**)

In a Schlenk tube, magnesium powder (0.316 g, 13 mmol, 2.17 equiv) was dispersed in dry THF (10 mL, 0.6 mol·L^–1^) and immersed in an oil bath preheated to 80 °C. 2-Bromo-1,3,5-triisopropylbenzene (∼100 mg) was added followed by a few drops of ethylene dibromide. After the reaction has started, the rest of 2-bromo-1,3,5-triisopropylbenzene was added dropwise (1.70 g, 6 mmol in total, 1.0 equiv). After completion of the Grignard reagent forming, confirmed by GCMS analysis (ca. 10 min), 1,2-dibromo-4-(trifluoromethyl)benzene (1.81 g, 6 mmol, 1.0 equiv) was added and the reaction was heated at 80 °C to the disappearance of substrates on GC (∼5 h). CuCl (0.49 g, 5 mmol, 0.83 equiv) was added at 80 °C in a few portions, and the temperature was lowered to 60 °C. Chlorodicyclohexylphosphine (1.63 g, 7 mmol, 1.16) was added, and the reaction was stirred at 60 °C overnight. It was cooled to RT; 10% Na_2_S_2_O_5_ (10 mL) was added followed by Et_2_O (30 mL); and the mixture was filtered by a 3 cm pad of silica. Layers were separated; the aqueous one was extracted with Et_2_O (2 × 20 mL), and the combined organic layers were washed several times with saturated aqueous ammonia until the blue color of the copper complex disappeared and dried with anhydrous magnesium sulfate. After removing the solvent on a rotary evaporator, the crude product was purified by flash chromatography, eluent PE, and crystallized from methanol to obtain 0.91 g (28% yield) of white crystalline solid as a mixture of two isomers. Mp = 101–103 °C. ^1^H NMR (CDCl_3_, 700 MHz): δ 7.79 (s, 2H), 7.56 (d, *J* = 8.1 Hz, 2H), 7.28–7.26 (m, 2H), 7.01 (s, 4H), 2.93 (m, *J* = 7.0 Hz, 2H), 2.30 (m, *J* = 7.0 Hz, 4H), 1.82–1.80 (m, 4H), 1.78–1.62 (m, 18H), 1.54–1.53 (m, 4H), 1.30 (d, *J* = 6.9 Hz, 12H), 1.21–1.07 (m, 30H), 0.96 (d, *J* = 6.7 Hz, 12H). ^13^C{1H} NMR (CDCl_3_, 75 MHz): δ 151.9, 151.5, 148.5, 148.3, 148.1, 145.9, 145.8, 138.3, 138.0, 135.2, 135.1, 135.0, 132.9, 131.8, 131.7, 130.1, 129.6, 128.9, 128.8, 128.6, 128.1, 124.4, 124.3, 122.7, 122.6, 122.3, 120.6, 120.5, 34.4, 34.3, 34.2, 34.1, 30.9, 30.8, 30.7, 30.7, 29.2, 29.1, 27.5, 27.4, 27.3, 27.2, 26.3, 25.9, 25.7, 24.0, 22.8. IR: (neat) ṽ 3048, 2914, 2856, 2210, 1902, 1694, 1589, 1508, 1475, 1438, 1279, 1181, 1164, 1145, 1101, 1037, 947, 913, 827, 813, 782, 751, 694, 578, 531, 512, 494, 455, 413 cm^–1^. Anal. calcd for C_34_H_48_F_3_P: C, 74.97; H, 8.88; found: C, 74.69; H, 8.91.

##### ^i^PrXPhos (**VIII**)

In a Schlenk tube, magnesium powder (0.583 g, 24 mmol, 2.36 equiv) was dispersed in dry THF (10 mL, 1.02 mol·L^–1^) and immersed in an oil bath preheated to 80 °C. 2-Bromo-1,3,5-triisopropylbenzene (∼100 mg) was added followed by a few drops of ethylene dibromide. After the reaction has started, the rest of 2-bromo-1,3,5-triisopropylbenzene was added dropwise (2.90 g, 10.2 mmol in total, 1.0 equiv). After completion of the Grignard reagent forming, confirmed by GCMS analysis (ca. 20 min), 1-bromo-2-chlorobenzene (2.10 g, 11 mmol, 1.08 equiv) was added and the reaction was heated at 80 °C to the disappearance of substrates on GC (∼1.5 h). CuCl (0.79 g, 8 mmol, 0.78 equiv) was added at 80 °C in a few portions, and the temperature was lowered to 60 °C. Chlorodiisopropylphosphine (1.68 g, 11 mmol, 1.08 equiv) was added, and the reaction was stirred at 60 °C for an additional 2 h. It was cooled to RT; 10% Na_2_S_2_O_5_ (10 mL) was added followed by Et_2_O (30 mL), and the mixture was filtered by a 3 cm pad of silica. Layers were separated; the aqueous one was extracted with Et_2_O (2 × 30 mL), and the combined organic layers were washed several times with saturated aqueous ammonia until the blue color of the copper complex disappeared and then washed with 5% citric acid, water, and brine and dried with anhydrous magnesium sulfate. After removing the solvent on a rotary evaporator, the crude product was purified by flash chromatography and eluent PE and crystallized from methanol to obtain 2.05 g (51% yield) of white crystalline solid. Mp = 105–107. ^1^H NMR (DMSO-*d*_6_, 400 MHz): δ 7.62–7.59 (m, 1H), 7.42–7.35 (m, 2H), 7.12–7.09 (m, 1H), 7.01 (s, 2H), 2.94–2.87 (m, 1H), 2.38–2.31 (m, 2H), 1.98–1.90 (m, 2H), 1.25 (d, *J* = 7.2 Hz, 6H), 1.14 (d, *J* = 6.9 Hz, 6H), 1.05–1.00 (m, 6H), 0.92–0.86 (m, 12H). ^13^C{1H} NMR (DMSO-*d*_6_,100 MHz): δ 147.7, 147.0, 146.8, 146.1, 132.2, 132.2, 131.4, 131.3, 128.4, 127.1, 120.3, 33.9, 30.7, 26.1, 24.5, 24.0, 23.8, 22.8, 22.8, 21.5, 21.3, 19.5, 19.3. IR: (neat) ṽ 3048, 2914, 2856, 2210, 1902, 1694, 1589, 1508, 1475, 1438, 1279, 1181, 1164, 1145, 1101, 1037, 947, 913, 827, 813, 782, 751, 694, 578, 531, 512, 494, 455, 413 cm^–1^. Anal. calcd for C_27_H_41_P: C, 81.77; H, 10.42; found: C, 81.79; H, 10.41.

## Data Availability

The data underlying this study are available in the published article and its Supporting Information.

## Supporting Information Available

Supporting Information and NMR spectra (PDF)
