# Supplementary material for: Kumada–Tamao–Corriu Type Reaction of Aromatic Bromo- and Iodoamines with Grignard Reagents
Source: J Org Chem. 2023 Nov 20;88(23):16167–75. doi: 10.1021/acs.joc.3c01553 (PMC10696545; doi:10.1021/acs.joc.3c01553)

Supporting Information

Kumada-Tamao-Corriu type reaction of aromatic bromo- and iodoamines with Grignard reagents

Alicja A. Zielińska[b,c], *Piotr Trzaska*[a,c], *Marcin Budny*[d], *Mariusz J. Bosiak**[,a]

[a] Department of Organic Chemistry, Faculty of Chemistry, Nicolaus Copernicus University in Toruń, 7 Gagarin Street, 87-100 Toruń, Poland

[b] Doctoral School of Exact and Natural Sciences "Academia Scientiarum Thoruniensis", Nicolaus Copernicus University in Toruń, 5 Grudziądzka Street, 87-100 Toruń, Poland

[c] Noctiluca SA, 7/41B Gagarina Street, 87-100 Toruń, Poland

[d] Synthex Technologies Sp. z o.o., 7/134B Gagarina Street, 87-100 Toruń, Poland

Corresponding Author: bosiu@umk.pl

Table of Contents

Table S1. Preliminary results for other palladium catalysts S2

Table S2. Screening for XPhos ligand loading S3

Figure S1. The Buchwald ligands S4

Scheme S1. Synthetic routes for ligand synthesis S5

a. Synthesis of **BPhos** (**IX**) S5

b. CF_3SPhos·HBF_4 (**I**) and $CF_3{}^{i}$PrSPhos·HBF_4 (**III**) S5

c. ^{i}PrSPhos·HBF_4 (**II**) S5

d. CF_3CPhos (**IV**) and $CF_3{}^{i}$PrCPhos·HBF_4 (**VI**) S5

e. ^{i}PrCPhos·HBF_4 (**V**) S6

f. CF_3XPhos (**VII**) S6

g. ^{i}PrXPhos (**VIII**) S6

Characterization of anilines (Entries Scheme 3) S7

^{1}H and ^{13}C{^{1}H} NMR spectra S12

Table S1. Preliminary results for other palladium catalysts

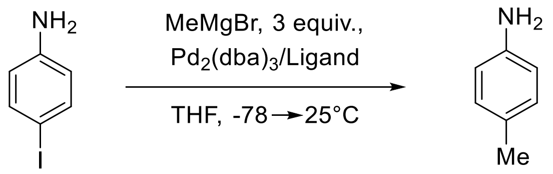

Entry	Catalyst (load)	$Pd_2(dba)_3$/Ligand [mol%]	Time [h]	Conversion[a] [%]
1	$Pd(PPh_3)_4$	1:0	24	1.8
2	$Pd(PPh_3)_2Cl_2$	1:0	24	0.6
3	Na_2PdCl_4	1:0	24	0.6
4	$Pd_2(dba)_3$	1:0	24	0
5	$PdCl_2$	1:0	24	0
6	$Pd(OAc)_2$	1:0	24	1.1
7	$Pd(ddpf)Cl_2$•DCM	1:0	24	0
8	$[Pd(allyl)]Cl]_2$	1:0	24	0
9	S-BINAP	1:3	2	4.4
10	PPh3	1:3	2	3.6
11	tri-(*o*-tolyl)phosphine	1:3	2	0.4
12	XantPhos	1:3	2	49.8

[a] GCMS, an average of two runs after 24 h at RT.

Table S2. Screening for XPhos ligand loading

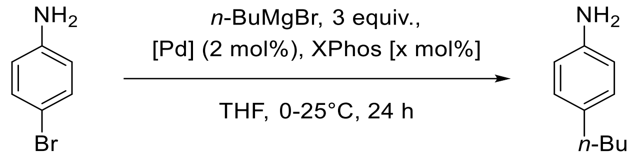

Entry	XPhos load	Aniline/product	Conversion[a] [%]
1	1.5 mol%	0.38	60.3
2	2 mol%	0.40	61.7
3	2.5 mol%	0.22	82.6
4	3 mol%	0.16	87.5
5	3.5 mol%	0.15	98.9
6	4 mol%	0.15	99.3
7	4.5 mol%	0.12	100
8	5 mol%	0.12	100
9	6 mol%	0.13	100
10	8 mol%	0.12	100

[a] GCMS, an average of two runs after 24 h at RT.

Figure S1. The Buchwald ligands

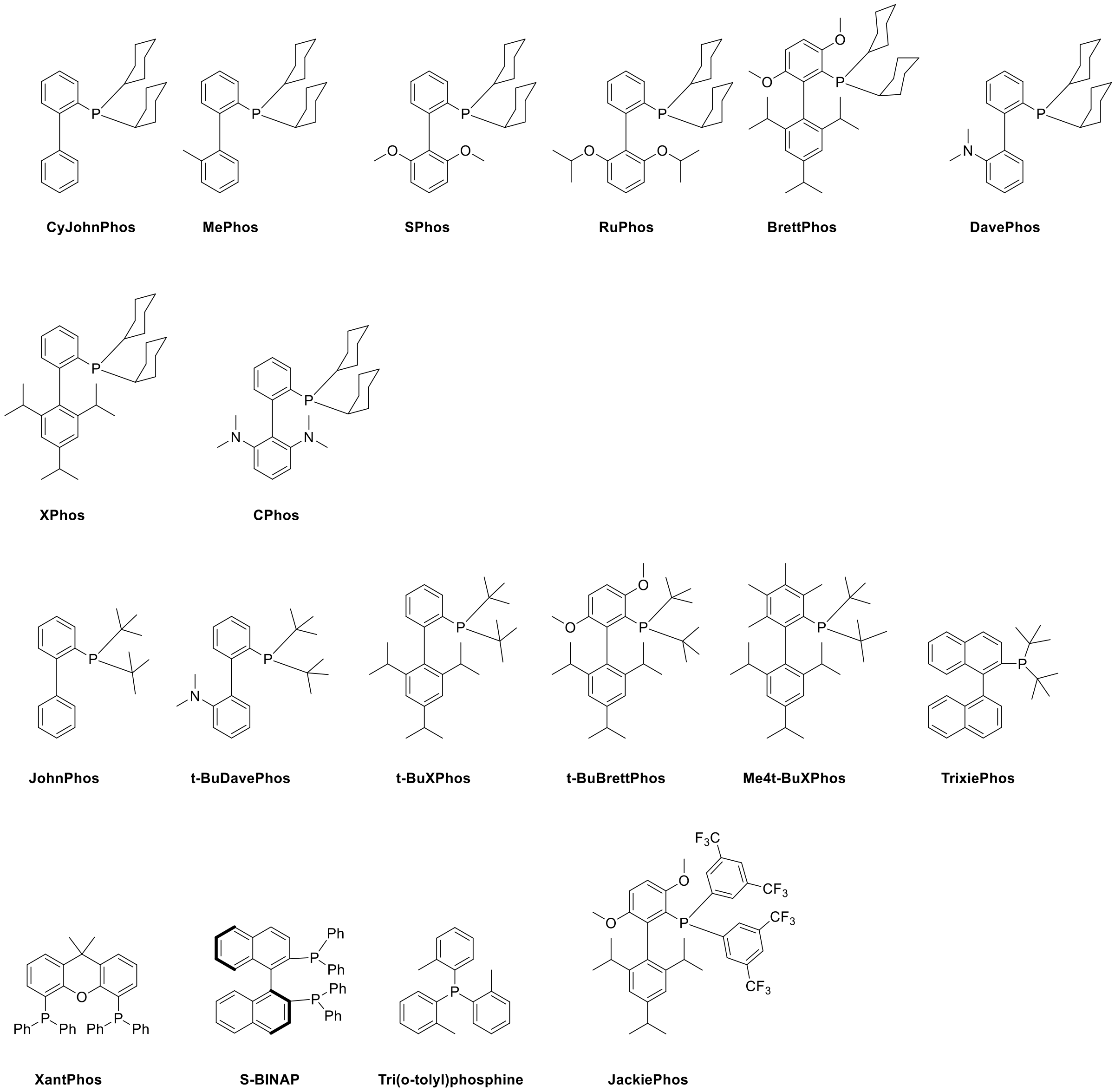

Scheme S1. Synthetic routes for ligand synthesis

a. Synthesis of **BPhos** (**IX**)

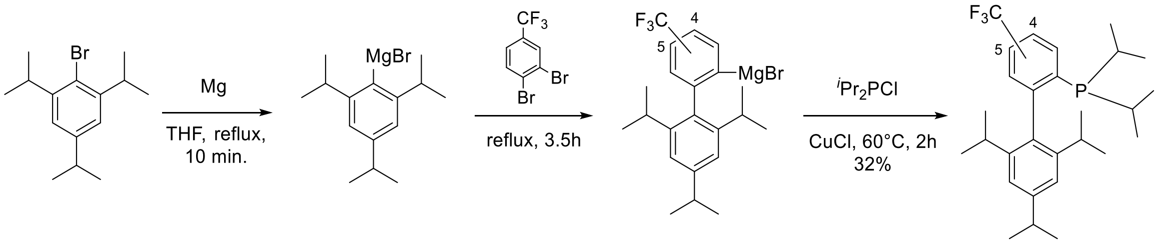

b. CF_3SPhos·HBF_4 (**I**) and $CF_3{}^i$PrSPhos·HBF_4 (**III**)

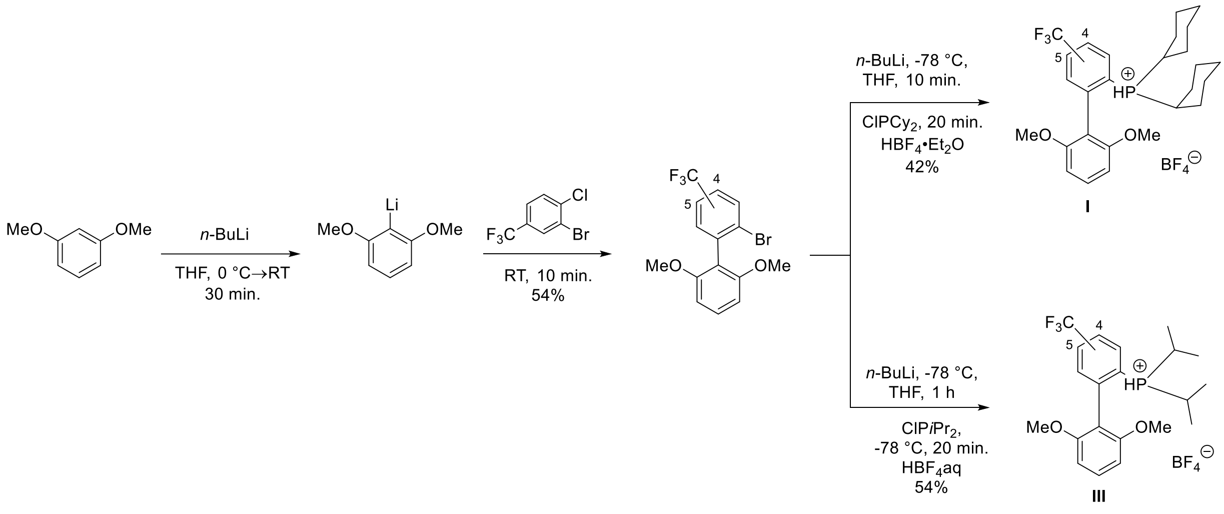

c. ^{i}PrSPhos·HBF_4 (**II**)

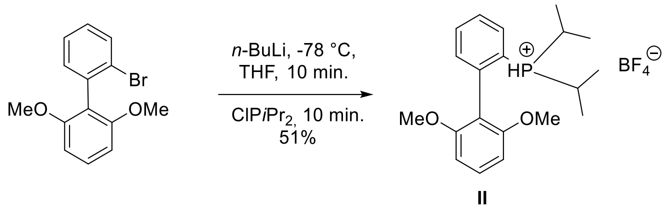

d. CF_3CPhos (**IV**) and $CF_3{}^i$PrCPhos·HBF_4 (**VI**)

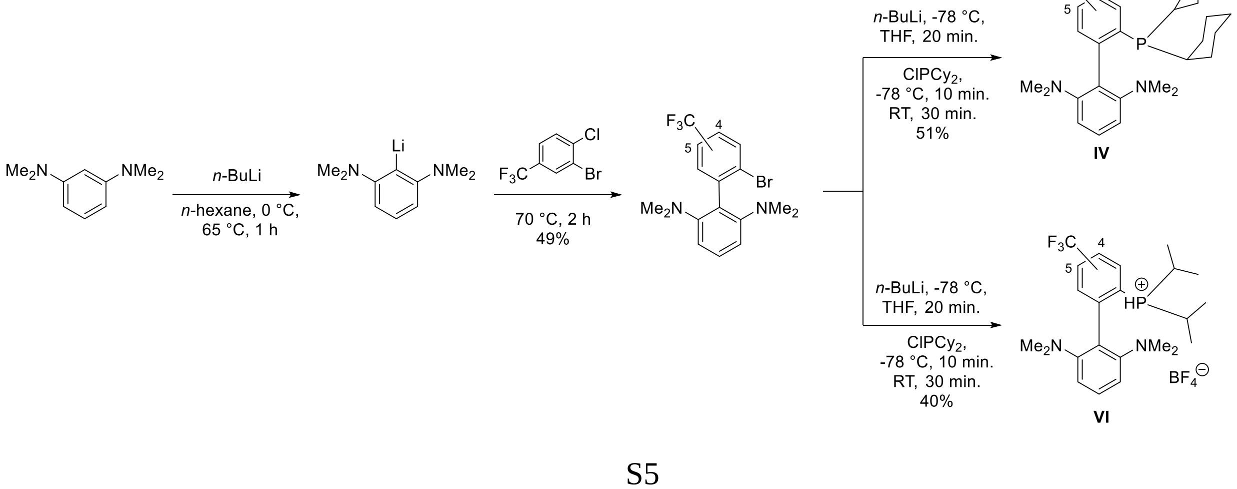

e. ^{i}PrCPhos·HBF$_4$ (**V**)

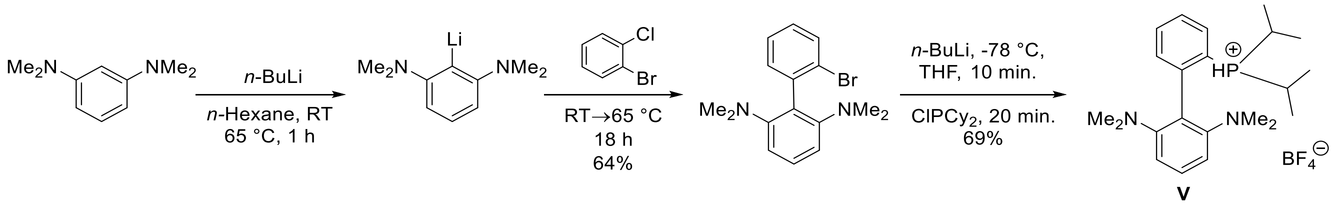

V

f. CF$_3$XPhos (**VII**)

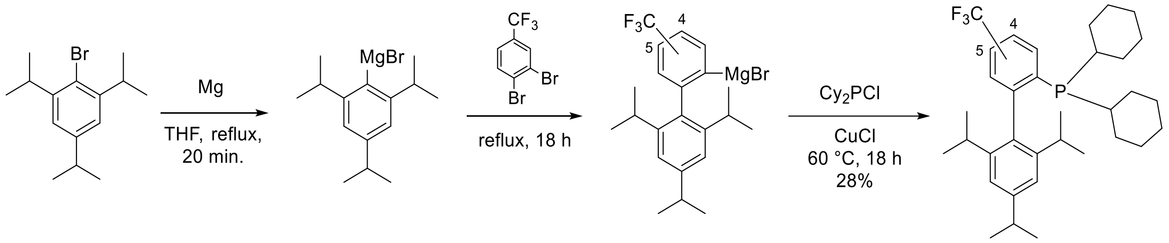

VII

g. ^{i}PrXPhos (**VIII**)

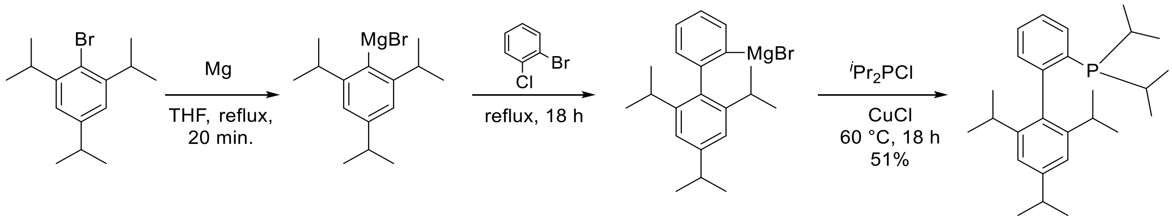

VIII

Characterization of anilines (Entries Scheme 3)

4-Toluidine (2a) Yield 93%, 199.2 mg (white solid); ^{1}H NMR ($CDCl_3$, 400 MHz): δ 6.97 (d, *J*=8.2 Hz, 2H), 6.61 (d, *J*=8.3 Hz, 2H), 3.46 (bs, 2H), 2.24 (s, 3H); $^{13}C\{^1H\}$ NMR ($CDCl_3$, 100 MHz): δ 143.8, 129.8, 127.8, 115.3, 20.5; Anal. Calcd. for C_7H_9N: C, 78.46; H, 8.47; N, 13.07; Found C, 78.07; H, 8.40; N, 13.54.

4-Butylaniline (2b) Yield 88%, 262.5 mg (brown oil); ^{1}H NMR ($CDCl_3$, 400 MHz): δ 6.97 (d, *J*=8.2 Hz, 2H), 6.61 (d, *J*=8.3 Hz, 2H), 3.46 (bs, 2H), 2.24 (s, 3H); $^{13}C\{^1H\}$ NMR ($CDCl_3$, 100 MHz): δ 144.0, 133.1, 129.1, 115.2, 34.8, 34.0, 22.3, 14.0; Anal. Calcd. for $C_{10}H_{15}N$: C, 80.48; H, 10.13; N, 9.39; Found: C, 80.65; H, 10.02; N, 9.54.

4-Isopropylaniline (3) Yield 51%, 137.8 mg (brown oil); ^{1}H NMR ($CDCl_3$, 400 MHz): δ 7.03 (d, *J*=8.3 Hz, 2H), 6.65 (d, *J*=8.3 Hz, 2H), 3.54 (bs, 1H), 2.81 (m, 2H), 1.21 (d, *J*=6.9 Hz, 6H); $^{13}C\{^1H\}$ NMR ($CDCl_3$, 100 MHz): δ 144.1, 139.2, 127.1, 115.2, 33.2, 24.2; Anal. Calcd. for $C_9H_{13}N$: C, 79.95; H, 9.69; N, 10.36; Found: C, 79.79; H, 9.46; N, 10.56.

4-(*tert*-Butyl)aniline (4) Yield 50%, 149.1 mg (brown oil); ^{1}H NMR ($CDCl_3$, 400 MHz): δ 7.19 (d, *J*=8.9 Hz, 2H), 6.65 (d, *J*=8.9 Hz, 2H), 3.55 (bs, 2H), 1.29 (s, 9H); $^{13}C\{^1H\}$ NMR ($CDCl_3$, 100 MHz): δ 143.8, 141.4, 126.1, 114.9, 33.9, 31.5; Anal. Calcd. for $C_{10}H_{15}N$: C, 80.48; H, 10.13; N, 9.39; Found: C, 80.63; H, 10.01; N, 9.13.

3-Toluidine (5) Yield 92%, 197.0 mg (brown oil); ^{1}H NMR ($CDCl_3$, 400 MHz): δ 7.07 (t, *J*=7.6 Hz, 1H), 6.61 (d, *J*=7.9 Hz, 1H), 6.54-6.50 (m, 2H), 3.52 (bs, 2H), 2.29 (s, 3H); $^{13}C\{^1H\}$ NMR ($CDCl_3$, 100 MHz): δ 146.4, 139.1, 129.2, 119.5, 116.0, 112.3, 21.5; Anal. Calcd. for C_7H_9N: C, 78.46; H, 8.47; N, 13.07; Found: C, 78.70; H, 8.53; N, 13.00.

3-Butylaniline (6) Yield 62%, 184.9 mg (brown oil); ^{1}H NMR ($CDCl_3$, 400 MHz): δ 7.08 (t, *J*=7.7 Hz, 1H), 6.61 (d, *J*=7.9 Hz, 1H), 6.54-6.52 (m, 2H), 3.60 (bs, 2H), 2.54 (m, 2H), 1.60 (m, *J*=7.7 Hz, 2H), 1.37 (m, *J*=7.5 Hz, 2H), 0.94 (t, *J*=7.3 Hz, 3H); $^{13}C\{^1H\}$ NMR ($CDCl_3$, 100 MHz): δ 146.3, 144.2, 129.1, 118.9, 115.3, 112.5, 35.7, 33.5, 22.4, 14.0; Anal. Calcd. for $C_{10}H_{15}N$: C, 80.48; H, 10.13; N, 9.39; Found: C, 80.31; H, 10.32; N, 9.51.

2-Toluidine (7) Not isolated.

2-Butylaniline (8) Not isolated. **2,4-Dimethylaniline (9)** Yield 93%, 226.2 mg (brown oil); ^{1}H NMR ($CDCl_3$, 400 MHz): δ 6.89-6.84 (m, 2H), 6.60 (d, *J*=7.8 Hz, 1H), 3.47 (bs, 1H), 2.24 (s, 3H), 2.15 (s, 3H); $^{13}C\{^1H\}$ NMR ($CDCl_3$, 100 MHz): δ 142.0, 131.1, 127.9, 127.3, 112.5, 115.1, 20.4, 17.3; Anal. Calcd. for $C_8H_{11}N$: C, 79.29; H, 9.15; N, 11.56; Found: C, 79.03; H, 9.33; N, 11.38.

4-Butyl-2-methylaniline (10) Yield 72%, 234.9 mg (brown oil); ^{1}H NMR ($CDCl_3$, 400 MHz): δ 6.89-6.85 (m, 2H), 6.61 (d, *J*=8.2 Hz, 1H), 3.51 (bs, 2H), 2.50 (m, 2H), 2.16 (s, 3H), 1.56 (m, *J*=7.7

Hz, 2H), 1.35 (m, *J*=7.2 Hz, 2H), 0.93 (t, *J*=7.3 Hz, 3H); $^{13}C\{^1H\}$ NMR ($CDCl_3$, 100 MHz): δ 142.2, 130.5, 127.0, 126.7, 122.4, 114.9, 34.8, 34.1, 22.4, 17.4, 14.0; Anal. Calcd. for $C_{11}H_{17}N$: C, 80.92; H, 10.50; N, 8.58; Found: C, 80.76; H, 10.72; N, 8.34.

2,4,6-Trimethylaniline (11) Yield 90%, 243.2 mg (brown oil); ^1H NMR ($CDCl_3$, 400 MHz): δ 6.78 (s, 2H), 3.45 (bs, 2H), 2.22 (s, 3H), 2.17 (s, 3H); $^{13}C\{^1H\}$ NMR ($CDCl_3$, 100 MHz): δ 140.1, 128.8, 127.2, 121.9, 20.4, 17.6; Anal. Calcd. for $C_9H_{13}N$: C, 79.95; H, 9.69; N, 10.36; Found: C, 79.54; H, 9.89; N, 10.61.

4-Butyl-2,6-dimethylaniline (12) Yield 71%, 251.6 mg (brown oil); ^1H NMR ($CDCl_3$, 400 MHz): δ 6.79 (s, 2H), 3.48 (bs, 2H), 2.48 (m, 2H), 2.18 (s, 6H), 1.56 (m, *J*=7.6 Hz, 2H), 1.36 (m, *J*=7.5 Hz, 2H), 0.94 (t, *J*=7.3 Hz, 3H); $^{13}C\{^1H\}$ NMR ($CDCl_3$, 100 MHz): δ 140.4, 132.5, 128.2, 121.8, 121.7, 118.0, 34.8, 34.2, 22.5, 17.7, 14.0; Anal. Calcd. for $C_{12}H_{19}N$: C, 81.30; H, 10.80; N, 7.90; Found: C, 81.62; H, 10.94; N, 7.57.

2-Fluoro-4-methylaniline (13) Yield 95%, 237.6 mg (brown oil); ^1H NMR ($CDCl_3$, 400 MHz): δ 6.81 (d, *J*=12.4 Hz, 1H), 6.75-6.66 (m, 2H), 3.57 (bs, 2H), 2.24 (s, 3H); $^{13}C\{^1H\}$ NMR ($CDCl_3$, 100 MHz): δ 152.8, 150.5, 131.7, 131.6, 128.7, 128.7, 124.8, 124.7, 117.0, 116.9, 115.9, 115.7, 20.4, 20.4; Anal. Calcd. for C_7H_8FN: C, 67.18; H, 6.44; F, 15.18; N, 11.19; Found: C, 67.47; H, 6.30; F, 15.33; N, 11.07.

4-Butyl-2-fluoroaniline (14) Yield 70%, 234.0 mg (brown oil); ^1H NMR ($CDCl_3$, 400 MHz): δ 6.81 (d, *J*=12.6 Hz, 1H), 6.76-6.73 (m, 1H), 6.71-6.67 (m, 1H), 3.57 (bs, 2H), 2.50 (m, 2H), 1.54 (m, *J*=7.4 Hz, 2H), 1.33 (m, *J*=7.8 Hz, 2H), 0.91 (t, *J*=7.5 Hz, 3H); $^{13}C\{^1H\}$ NMR ($CDCl_3$, 100 MHz): δ 152.9, 150.5, 134.0, 134.0, 131.9, 131.7, 124.1, 124.1, 116.9, 116.9, 115.2, 115.0, 34.6, 33.7, 22.2, 13.9; Anal. Calcd. for $C_{10}H_{14}FN$: C, 71.82; H, 8.44; F, 11.36; N, 8.38; Found: C, 71.98; H, 8.27; F, 11.19; N, 8.52.

2,5-Difluoro-4-methylaniline (15) Yield 91%, 260.4 mg (brown oil); ^1H NMR ($CDCl_3$, 400 MHz): δ 6.77 (dd, *J*=10.7, 6.4 Hz, 1H), 6.45 (dd, *J*=11.4, 6.4 Hz, 1H), 3.64 (bs, 2H), 2.14 (s, 3H); $^{13}C\{^1H\}$ NMR ($CDCl_3$, 100 MHz): δ 158.8, 155.6, 149.0, 145.9, 133.1, 132.9, 132.8, 117.1, 116.9, 114.1, 114.0, 113.8, 113.8, 103.8, 103.7, 103.4, 103.4, 13.7; Anal. Calcd. for $C_7H_7F_2N$: C, 58.74; H, 4.93; F, 26.55; N, 9.79; Found: C, 58.48; H, 5.07; F, 26.36; N, 9.95.

4-Butyl-2,5-difluoroaniline (16) Yield 81%, 299.9 mg (brown oil); ^1H NMR ($CDCl_3$, 400 MHz): δ6.80-6.75 (m, 1H), 6.46-6.42 (m, 1H), 3.65 (bs, 2H), 2.49 (m, 2H), 1.52 (m, *J*=7.7 Hz, 2H), 1.34 (m, *J*=8.0 Hz, 2H), 0.92 (t, *J*=6.4 Hz, 3H) ; $^{13}C\{^1H\}$ NMR ($CDCl_3$, 100 MHz): δ 116.3, 116.2, 116.1, 116.0, 103.8, 103.7, 103.5, 103.5, 32.4, 27.8, 22.2, 13.9; Anal. Calcd. for $C_{10}H_{13}F_2N$: C, 64.85; H, 7.07; F, 20.51; N, 7.56; Found: C, 64.66; H, 7.24; F, 20.38; N, 7.72.

2-Chloro-4-methylaniline (17) Yield 88%, 248.2 mg (brown oil); ^1H NMR ($CDCl_3$, 400 MHz): δ 7.07 (s, 1H), 6.87 (d, *J*=8.6 Hz, 1H), 6.68 (d, *J*=8.3 Hz, 1H), 3.87 (bs, 2h), 2.22 (s, 3H); $^{13}C\{^1H\}$

NMR ($CDCl_3$, 100 MHz): δ 140.3, 129.7, 128.7, 128.3, 119.2, 115.9, 20.2; Anal. Calcd. for C_7H_8ClN: C, 59.38; H, 5.69; Cl, 25.04; N, 9.89; Found: C, 59.61; H, 5.44; Cl, 25.30; N, 9.63.

4-Butyl-2-chloroaniline (18) Yield 66%, 241.7 mg (brown oil); ^{1}H NMR ($CDCl_3$, 400MHz): δ 7.07 (d, *J*=1.9 Hz, 1H), 6.88 (d, *J*=7.9 Hz, 1H), 6.69 (d, *J*=7.9 Hz, 1H), 3.92 (bs, 2H), 2.48 (m, 2H), 1.54 (m, *J*=7.7 Hz, 2H), 1.33 (m, *J*=7.1 Hz, 2H), 0.92 (t, *J*=7.1 Hz, 3H); $^{13}C\{^1H\}$ NMR ($CDCl_3$, 100 MHz): δ 140.5, 134.0, 129.0, 127.6, 119.2, 115.9, 34.5, 33.7, 22.2, 13.9; Anal. Calcd. for $C_{10}H_{14}ClN$: C, 65.39; H, 7.68; Cl, 19.30; N, 7.63; Found: C, 65.14; H, 7.74; Cl, 19.05; N, 7.81.

4-Methylnaphthalen-1-amine (19) Yield 94%, 295.3 mg (brownish solid); ^{1}H NMR ($CDCl_3$, 400MHz): δ 7.97 (d, *J*=8.2 Hz, 1H), 7.88 (d, *J*=8.2 Hz, 1H), 7.51 (m, 2H), 7.14 (d, *J*=7.5 Hz, 1H), 6.73 (d, *J*=7.5 Hz, 1H), 3.97 (bs, 2H), 2.61 (s, 3H); $^{13}C\{^1H\}$ NMR ($CDCl_3$, 100 MHz): δ 140.4, 133.2, 126.8, 125.7, 125.0, 124.9, 124.6, 124.2, 121.4, 109.7, 19.0; Anal. Calcd. for $C_{11}H_{11}N$: C, 84.04; H, 7.05; N, 8.91; Found: C, 84.23; H, 7.19; N, 8.78.

4-Butylnaphthalen-1-amine (20) Yield 36%, 143.4 mg (brown oil); ^{1}H NMR ($CDCl_3$, 400 MHz): δ 8.03 (d, *J*=8.3 Hz, 1H), 7.88 (d, *J*=8.3 Hz, 1H), 7.47-7.46 (m, 2H), 7.14 (d, *J*=7.2 Hz, 1H), 6.76 (d, *J*=7.2 Hz, 1H), 4.02 (bs, 2H), 2.98 (m, 2H), 1.70 (m, *J*=7.9 Hz, 2H), 1.45 (m, *J*=7.4 Hz, 2H), 0.97 (t, *J*=7.2 Hz, 3H); $^{13}C\{^1H\}$ NMR ($CDCl_3$, 100 MHz): δ 142.0, 140.0, 134.3, 132.5, 130.2, 126.1, 125.6, 124.7, 124.5, 123.7, 121.5, 110.0, 33.2, 32.5, 22.8, 14.0; Anal. Calcd. for $C_{14}H_{17}N$: C, 84.37; H, 8.60; N, 7.03; Found: C, 84.17; H, 8.89; N, 6.88.

3,4-Dimethylaniline (21) Yield 69%, 167.1 mg (brown oil); ^{1}H NMR ($CDCl_3$, 400 MHz): δ 6.92 (d, *J*=7.8 Hz, 1H), 6.52 (s, 1H), 6.47 (d, *J*=7.6 Hz, 1H), 3.48 (bs, 2H), 2.19 (s, 3H), 2.16 (s, 3H); $^{13}C\{^1H\}$ NMR ($CDCl_3$, 100 MHz): δ 144.2, 137.4, 130.3, 126.5, 116.9, 112.6, 19.9, 18.7; Anal. Calcd. for $C_8H_{11}N$: C, 79.29; H, 9.15; N, 11.56; Found: C, 79.49; H, 9.34; N, 11.29.

3-Butyl-4-methylaniline (22) Not isolated.

4-Methylbenzene-1,2-diamine (23) Yield 90%, 219.7 mg (brown oil); ^{1}H NMR ($CDCl_3$, 400 MHz): δ 6.61 (d, *J*=7.6 Hz, 1H), 6.54-6.51 (m, 2H), 3.32 (bs, 4H), 2.21 (s, 3H); $^{13}C\{^1H\}$ NMR ($CDCl_3$, 100 MHz): δ 135.0, 131.9, 130.0, 120.5, 117.4, 117.0, 20.7; Anal. Calcd. for $C_7H_{10}N_2$: C, 68.82; H, 8.25; N, 22.93; Found: C, 68.57; H, 8.43; N, 22.68.

4-Butylbenzene-1,2-diamine (24) Yield 86%, 282.3 mg (brown oil); ^{1}H NMR ($CDCl_3$, 400 MHz): δ 6.63 (d, *J*=7.9 Hz, 1H), 6.55-6.52 (m, 2H), 3.11 (bs, 4H), 2.47 (m, 2H), 1.54 (m, *J*=7.6 Hz, 2H), 1.34 (m, *J*=7.6 Hz, 2H), 0.92 (t, *J*=7.1 Hz, 3H); $^{13}C\{^1H\}$ NMR ($CDCl_3$, 100 MHz): δ 135.3, 134.9, 132.1, 119.9, 117.0, 116.8, 35.0, 33.9, 22.4, 14.0; Anal. Calcd. for $C_{10}H_{16}N_2$: C, 73.13; H, 9.82; N, 17.06; Found: C, 73.01; H, 9.98; N, 17.22.

***N,N*,2-Trimethylaniline (25)** Yield 77%, 208.1 mg (brown oil); ^{1}H NMR ($CDCl_3$, 400 MHz): δ 7.23-7.19 (m, 2H), 7.09 (dd, *J*=8.1, 1.2 Hz, 1H), 7.0 (td, *J*=7.2, 1.3 Hz, 1H), 2.75 (s, 6H), 2.39 (s, 3H);

$^{13}C\{^1H\}$ NMR ($CDCl_3$, 100 MHz): δ 152.8, 132.2, 131.2, 126.5, 122.6, 118.4, 44.3, 18.4; Anal. Calcd. for $C_9H_{13}N$: C, 79.95; H, 9.69; N, 10.36; Found: C, 79.95; H, 9.69; N, 10.36.

2-Butyl-*N,N*-dimethylaniline (26) Yield 57%, 202.0 mg (brown oil); ^1H NMR ($CDCl_3$, 400 MHz): δ 7.22-7.17 (m, 1H), 7.11-7.08 (m, 1H), 7.01 (t, *J*=7.7 Hz, 1H), 6.77-6.75 (m, 1H), 2.72-2.70 (m, 8H), 1.65-1.61 (m, 2H), 1.42 (m, *J*=7.0 Hz, 2H), 0.96 (t, *J*=7.3 Hz, 3H); $^{13}C\{^1H\}$ NMR ($CDCl_3$, 100 MHz): δ 152.7, 137.6, 129.1, 126.3, 123.2, 119.3, 45.2, 32.8, 30.3, 22.9, 14.1; Anal. Calcd. for $C_{12}H_{19}N$: C, 81.30; H, 10.80; N, 7.90; Found: C, 81.52; H, 10.61; N, 8.02.

1-(*p*-Tolyl)piperidine (27) Yield 89%, 311.7 mg (brown oil); ^1H NMR ($CDCl_3$, 400 MHz): δ 7.07 (d, *J*=8.9 Hz, 2H), 6.87 (d, *J*=8.4 Hz, 2H), 3.11-3.09 (m, 4H), 2.27 (s, 3H), 1.75-1.69 (m, 4H), 1.59-1.55 (m, 2H); $^{13}C\{^1H\}$ NMR ($CDCl_3$, 100 MHz): δ 150.3, 129.5, 128.7, 117.0, 51.3, 26.0, 24.3, 20.4; Anal. Calcd. for $C_{12}H_{17}N$: C, 82.23; H, 9.78; N, 7.99; Found: C, 82.46; H, 9.50; N, 7.63.

1-(4-Butylphenyl)piperidine (28) Yield 87%, 377.9 mg (brown oil); ^1H NMR ($CDCl_3$, 400 MHz): δ 7.07 (d, *J*=8.7 Hz, 2H), 6.87 (d, *J*=8.7 Hz, 2H), 3.12-3.09 (m, 4H), 2.53 (m, 2H), 1.74-1.69 (m, 4H), 1.61-1.53 (m, 4H), 1.35 (m, *J*=7.3 Hz, 2H), 0.92 (t, *J*=7.4 Hz, 3H); $^{13}C\{^1H\}$ NMR ($CDCl_3$, 100 MHz): δ 150.4, 133.8, 128.9, 116.8, 51.2, 34.7, 33.8, 26.0, 24.3, 22.4, 14.0; Anal. Calcd. for $C_{15}H_{23}N$: C, 82.89; H, 10.67; N, 6.44; Found: C, 82.71; H, 10.88; N, 6.12.

4-Methyl-*N*-phenylaniline (29) Yield 90%, 329.6 mg (yellowish solid); ^1H NMR ($CDCl_3$, 400 MHz): δ 7.26 (t, *J*=7.8 Hz, 2H), 7.11 (d, *J*=8.1 Hz, 2H), 7.04-7.01 (m, 4H), 6.90 (t, *J*=7.2 Hz, 1H), 5.64 (bs, 1H), 2.33 (s, 3H); $^{13}C\{^1H\}$ NMR ($CDCl_3$, 100 MHz): δ 144.0, 140.3, 131.0, 130.0, 129.3, 120.3, 119.0, 116.9, 20.7; Anal. Calcd. for $C_{13}H_{13}N$: C, 85.21; H, 7.15; N, 7.64; Found: C, 85.02; H, 7.34; N, 7.38.

4-Butyl-*N*-phenylaniline (30) Yield 79%, 355.7 mg (yellowish solid); ^1H NMR ($CDCl_3$, 400 MHz): δ 7.30-7.23 (m, 2H), 7.11-7.01 (m, 6H), 6.91-6.87 (m, 1H), 5.64 (bs, 1H), 2.57 (m, 2H), 1.60 (m, *J*=7.8 Hz, 2H), 1.37 (m, *J*=7.3Hz, 2H), 0.94 (t, *J*=7.1 H, 3H); $^{13}C\{^1H\}$ NMR ($CDCl_3$, 100 MHz): δ 143.9, 140.5, 136.1, 129.3, 129.2, 121.0, 120.3, 118.7, 117.8, 117.0, 34.9, 33.8, 22.4, 14.0; Anal. Calcd. for $C_{16}H_{19}N$: C, 85.28; H, 8.50; N, 6.22; Found: C, 85.55; H, 8.23; N, 6.40.

3-Methyl-*N*-phenylaniline (31) Yield 92%, 336.9 mg (yellowish solid); ^1H NMR ($CDCl_3$, 400 MHz): δ 7.29-7.25 (m, 2H), 7.16 (t, *J*=7.7 Hz, 1H), 7.07 (d, *J*=8.1 Hz), 6.95-6.88 (m, 3H), 6.76 (d, *J*=7.5 Hz, 1H), 5.66 (bs, 1H), 2.32 (s, 3H); $^{13}C\{^1H\}$ NMR ($CDCl_3$, 100 MHz): δ 143.3, 143.1, 139.2, 129.3, 129.2, 121.9, 120.9, 118.5, 117.8, 114.9, 21.5; Anal. Calcd. for $C_{13}H_{13}N$: C, 85.21; H, 7.15; N, 7.64; Found: C, 85.02; H, 7.34; N, 7.39.

3-Butyl-*N*-phenylaniline (32) Yield 66%, 297.2 mg (yellowish solid); ^1H NMR ($CDCl_3$, 400 MHz): δ 7.34 (d, *J*=8.3 Hz, 2H), 7.22 (t, *J*=7.8 Hz, 2H), 7.16-7.10 (m, 3H), 6.91 (t, *J*=7.2 Hz, 1H), 6.75 (d, *J*=7.8 Hz, 1H), 2.51 (m, 2H), 1.51 (m, *J*=7.6 Hz, 2H), 1.26 (m, *J*=7.4 Hz, 2H), 0.88 (t, *J*=7.4 Hz, 3H); $^{13}C\{^1H\}$ NMR ($CDCl_3$, 100 MHz): δ 143.8, 143.8, 143.4, 129.0, 128.8, 122.4, 121.7, 118.4, 118.0,

115.7, 35.7, 33.5, 22.2, 14.0; Anal. Calcd. for $C_{16}H_{19}N$: C, 85.28; H, 8.50; N, 6.22; Found C, 85.46; H, 8.76; N, 6.04.

3-Methyl-*N,N*-diphenylaniline (33) Yield 92%, 476.8 mg (yellowish solid); ^{1}H NMR ($CDCl_3$, 400 MHz): δ 7.24 (t, *J*=7.9 Hz, 4H), 7.14 (t, *J*=7.7 Hz, 1H), 7.08 (d, *J*=8.3 Hz, 4H), 7.00 (t, *J*=7.4 Hz, 2H), 6.92-6.83 (m, 3H), 2.26 (s, 3H); $^{13}C\{^1H\}$ NMR ($CDCl_3$, 100 MHz): δ 148.0, 147.8, 139.1, 129.2, 129.0, 125.0, 124.1, 123.7, 122.5, 121.6, 21.4; Anal. Calcd. for $C_{19}H_{17}N$: C, 87.99; H, 6.61; N, 5.40; Found: C, 87.78; H, 6.83; N, 5.32.

3-Butyl-*N,N*-diphenylaniline (34) Yield 70%, 421.7 mg (yellowish solid); ^{1}H NMR ($CDCl_3$, 400 MHz): δ 7.26-7.22 (m, 4H), 7.15 (t, *J*=7.7 Hz, 1H), 7.10-7.07 (m, 4H), 7.00 (t, *J*=7.1Hz, 2H), 6.95 (s, 1H), 6.90-6.84 (m, 2H), 2.52 (m, 2H), 1.54 (m, *J*=7.5 Hz, 2H), 1.32 (m, *J*=7.4 Hz, 2H), 0.90 (t, *J*=7.4 Hz, 3H); $^{13}C\{^1H\}$ NMR ($CDCl_3$, 100 MHz): δ 148.0, 147.7, 144.1, 129.2, 129.1, 129.0, 124.5, 124.2, 124.0, 123.1, 122.4, 121.8, 35.6, 33.5, 22.3, 13.9; Anal. Calcd. for $C_{22}H_{23}N$: C, 87.66; H, 7.69; N, 4.65; Found: C, 87.94; H, 7.96; N, 4.28.

3-Methyl-9*H*-carbazole (35) Yield 92%, 333.2 mg (white solid); ^{1}H NMR ($CDCl_3$, 400 MHz): δ 8.07 (d, *J*=8.0 Hz, 1H), 7.98 (bs, 1H), 7.90 (m, 1H), 7.44-7.41 (m, 2H), 7.35 (d, *J*=8.3 Hz, 1H), 7.26 (d, *J*=7.7 Hz, 1H), 7.24 (t, *J*=7.3 Hz, 1H), 2.56 (s, 3H); $^{13}C\{^1H\}$ NMR ($CDCl_3$, 100 MHz): δ 140.0, 137.7, 128.8, 127.2, 125.7, 123.6, 123.3, 120.3, 119.2, 110.5, 110.2, 21.4; Anal. Calcd. for $C_{13}H_{11}N$: C, 86.15; H, 6.12; N, 7.73; Found: C, 86.02; H, 6.37; N, 7.49.

3-Butyl-9*H*-carbazole (36) Yield 70%, 312.4 mg (white solid); ^{1}H NMR ($CDCl_3$, 400 MHz): δ8.07 (d, *J*=7.9 Hz, 1H), 7.93 (bs, 1H), 7.89 (s, 1H), 7.43-7.40 (m, 2H), 7.34 (d, *J*=8.7 Hz, 1H), 7.27-7.21 (m, 2H), 2.80 (m, 2H), 1.71 (m, *J*=7.4 Hz, 2H), 1.43 (m, *J*=7.5 Hz, 2H), 0.97 (t, *J*=7.4 Hz, 3H) ; $^{13}C\{^1H\}$ NMR ($CDCl_3$, 100 MHz): δ 139.8, 137.9, 134.1, 126.7, 125.6, 123.5, 123.3, 120.2, 119.7, 119.2, 110.6, 110.2, 35.8, 34.5, 22.4, 14.0; Anal. Calcd. for $C_{16}H_{17}N$: C, 86.05; H, 7.67; N, 6.27; Found: C, 86.41; H, 7.50; N, 6.04

3,6-Dimethyl-9*H*-carbazole (37) Yield 90%, 351.2 mg (white solid); ^{1}H NMR ($CDCl_3$, 400 MHz): δ 7.86 (m, 3H), 7.32 (d, *J*=8.4Hz, 2H), 7.24 (d, *J*=8.4 Hz, 2H), 2.55 (s, 6H); $^{13}C\{^1H\}$ NMR ($CDCl_3$, 100 MHz): δ 138.1, 128.5, 127.0, 123.4, 120.2, 110.2, 21.4; Anal. Calcd. for $C_{14}H_{13}N$: C, 86.12; H, 6.71; N, 7.17; Found: , 86.38; H, 6.88; N, 7.02.

3,6-Dibutyl-9*H*-carbazole (38) Yield 67%, 374.1 mg (white solid); ^{1}H NMR ($CDCl_3$, 400 MHz): δ 7.86 (s, 2H), 7.83 (bs, 1H), 7.35-7.28 (m, 2H), 7.23 (dd, *J*=8.2, 1.7 Hz, 2H), 2.79 (m, 4H), 1.71 (m, 4H), 1.46-1.37 (m, 4H), 0.97 (*t*, *J*=7.4 Hz, 6H); $^{13}C\{^1H\}$ NMR ($CDCl_3$, 100 MHz): δ 138.3, 133.8, 126.4, 123.4, 119.6, 110.2, 35.7, 34.5, 22.4, 14.1; Anal. Calcd. for $C_{20}H_{25}N$: C, 85.97; H, 9.02; N, 5.01; Found: C, 85.87; H, 9.17; N, 5.24.

^{1}H and ^{13}C{^{1}H} NMR spectra

BPhos

^{1}H NMR (CDCl3, 700 MHz)

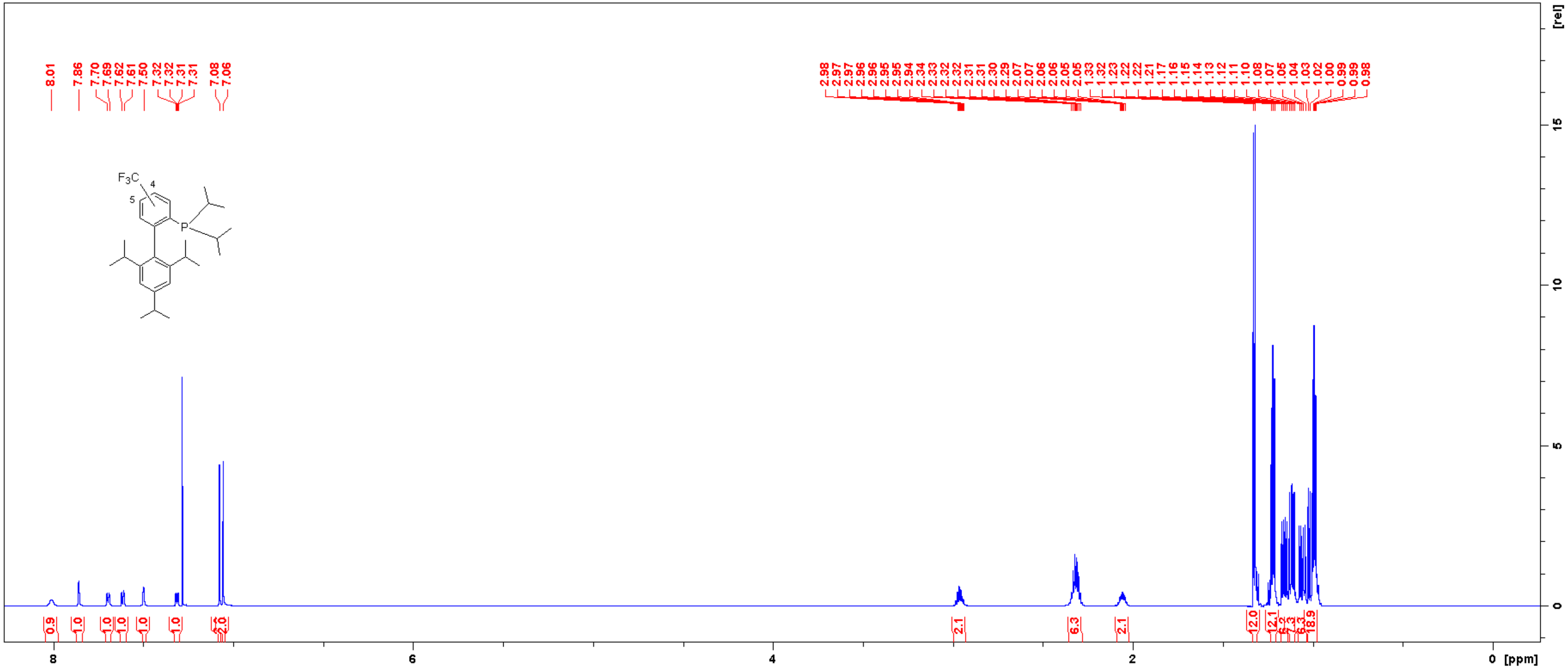

BPhos

$^{13}C\{^{1}H\}$NMR (CDCl3, 75.5 MHz)

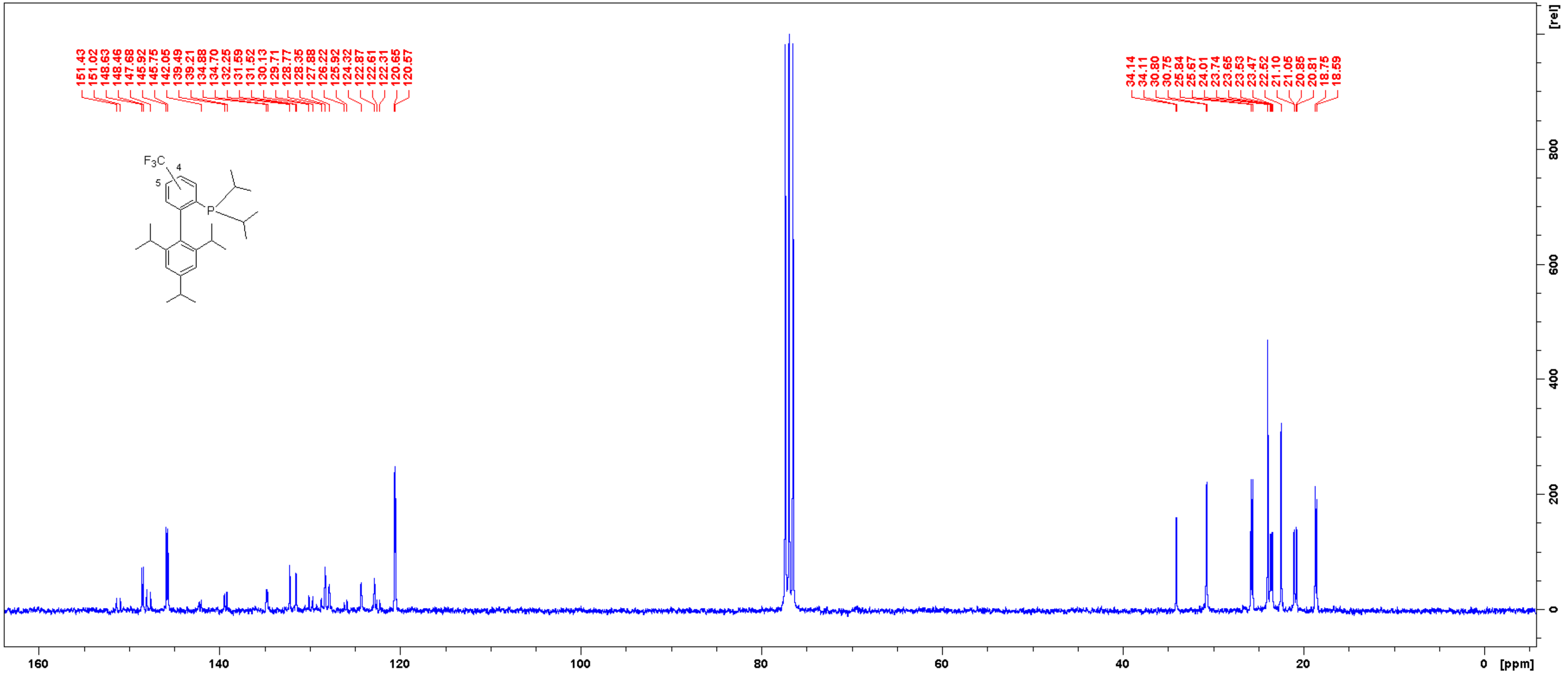

4-CF_3 BPhos

^{1}H NMR (CDCl3, 700 MHz)

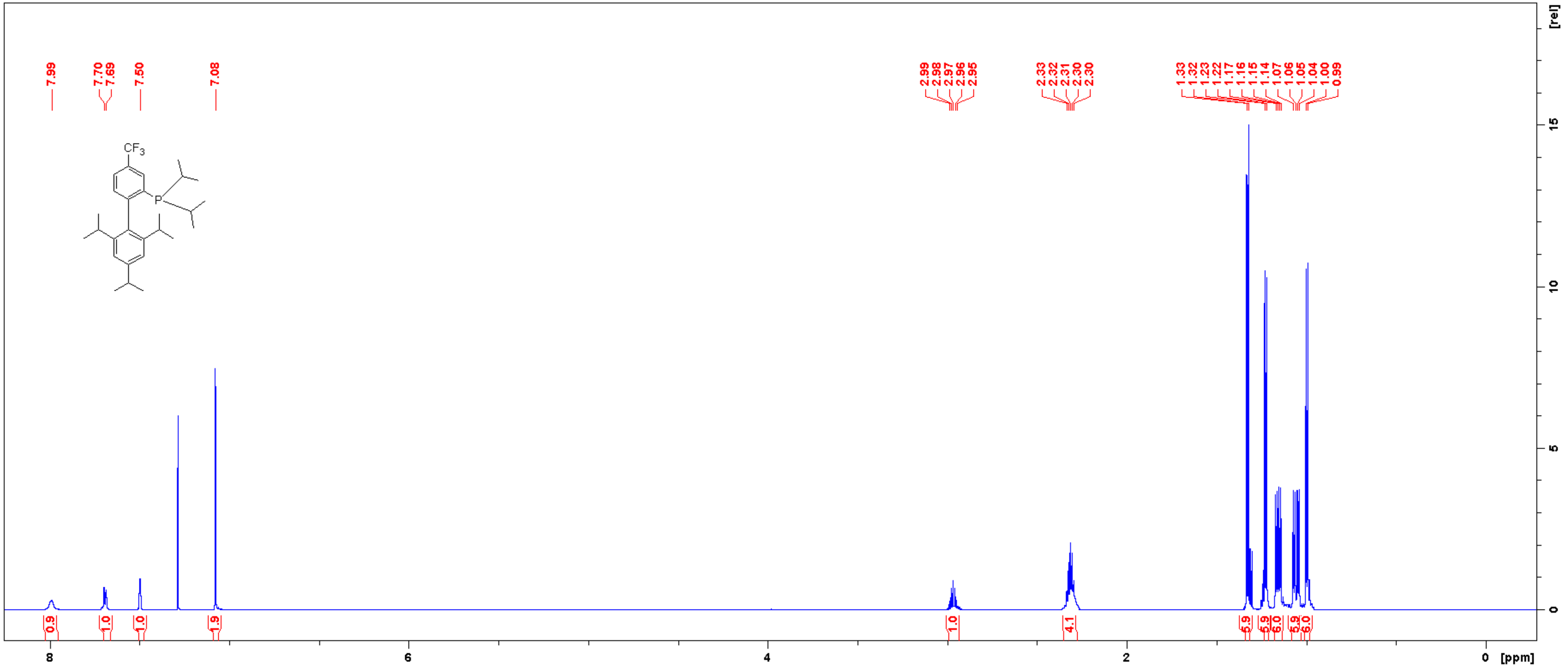

4-CF3 BPhos

$^{13}C\{^{1}H\}$NMR (CDCl3, 75.5 MHz)

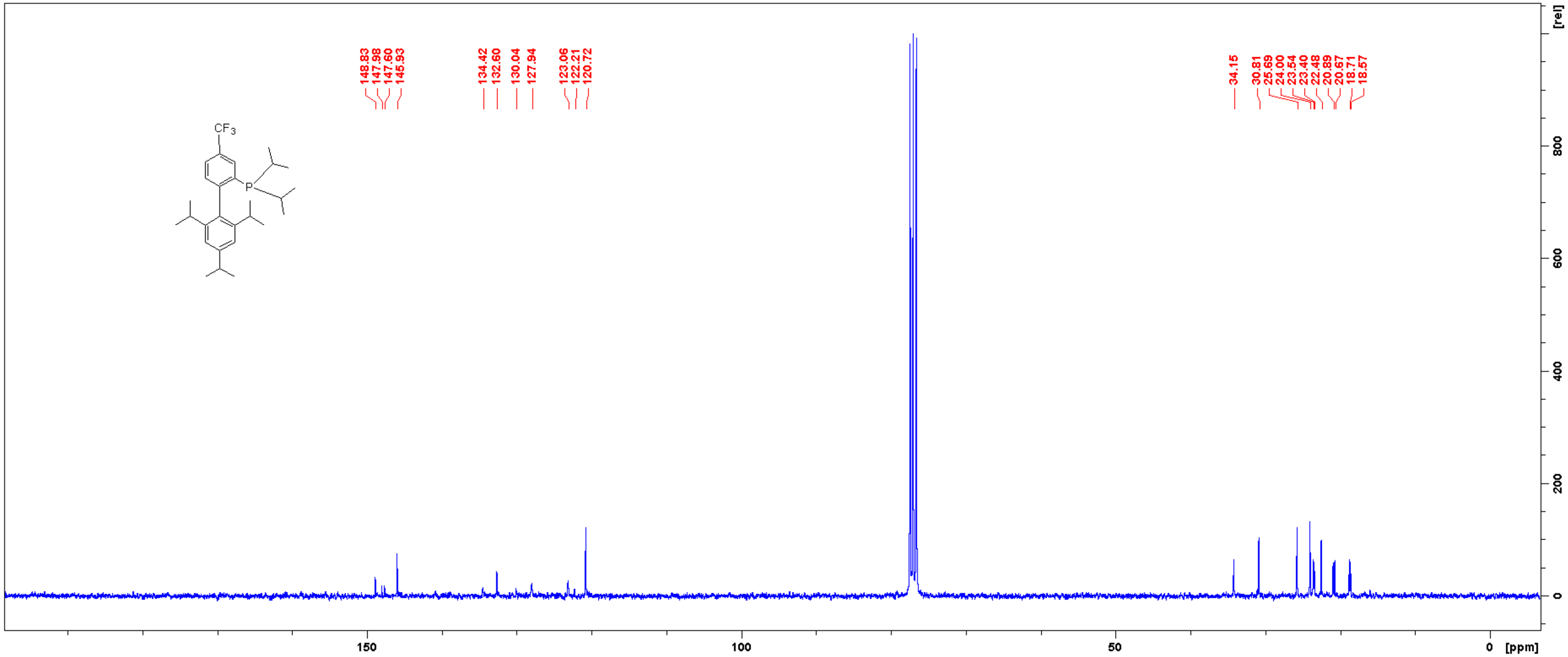

5-CF_3 BPhos

^{1}H NMR ($CDCl_3$, 700 MHz)

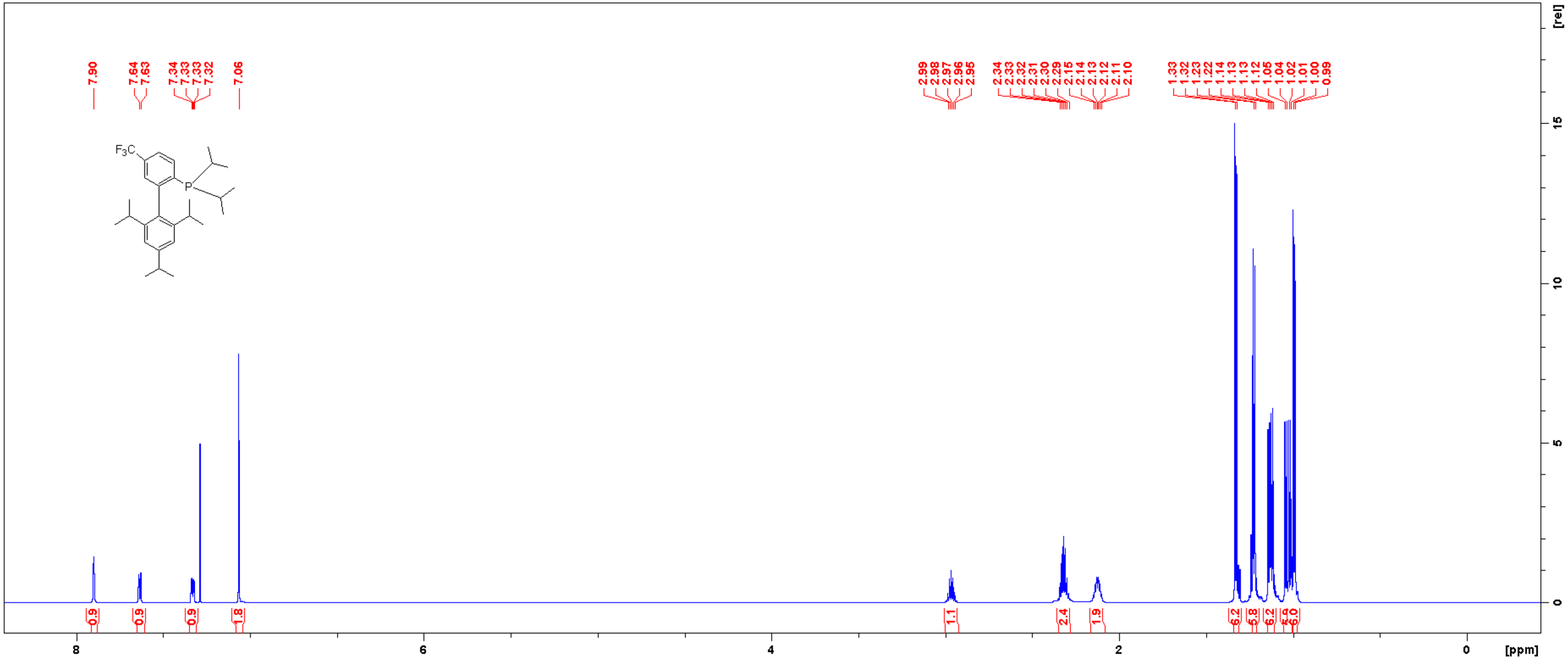

5-CF_3 BPhos

$^{13}C\{^1H\}$NMR (75.5 MHz, CDCl3)

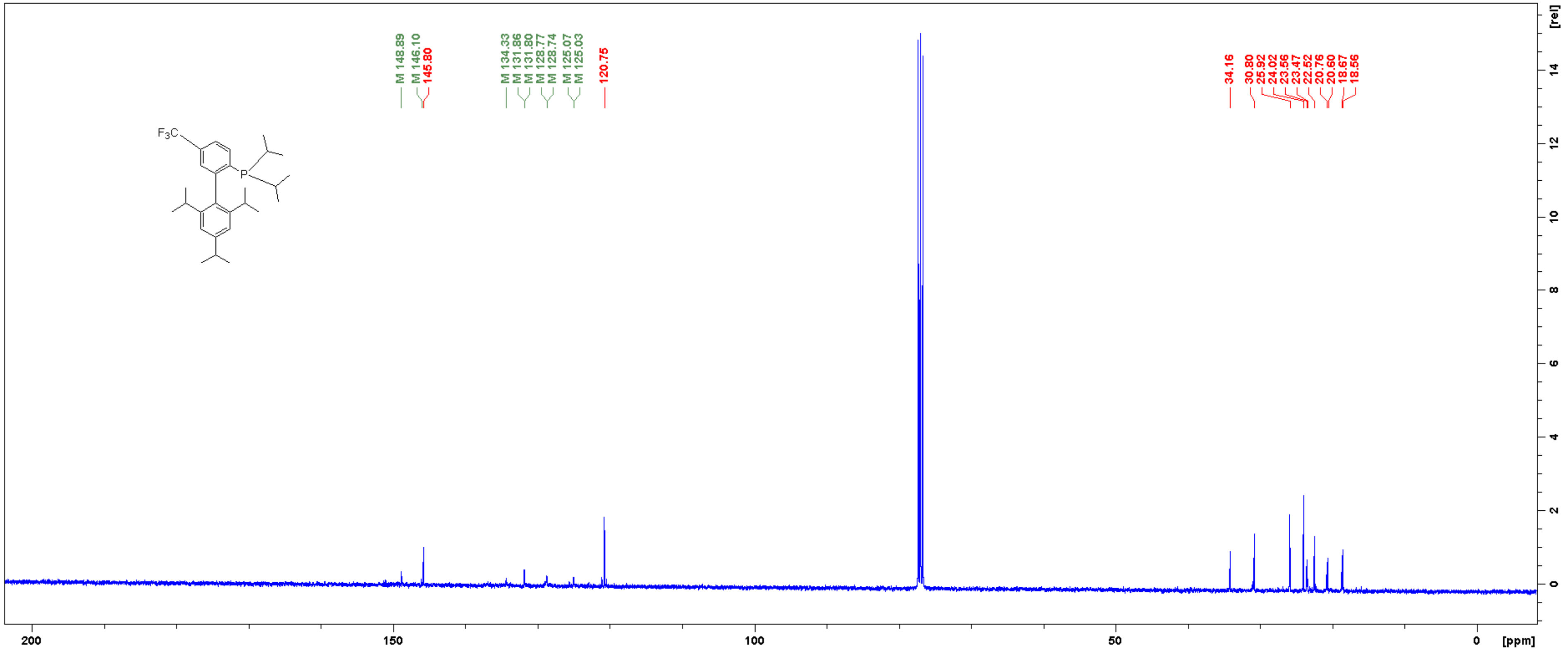

2-Bromo-2',6'-dimethoxy-4-(trifluoromethyl)-1,1'-biphenyl and 2-bromo-2',6'-dimethoxy-5-(trifluoromethyl)-1,1'-biphenyl

^{1}H NMR (CDCl3, 400 MHz)

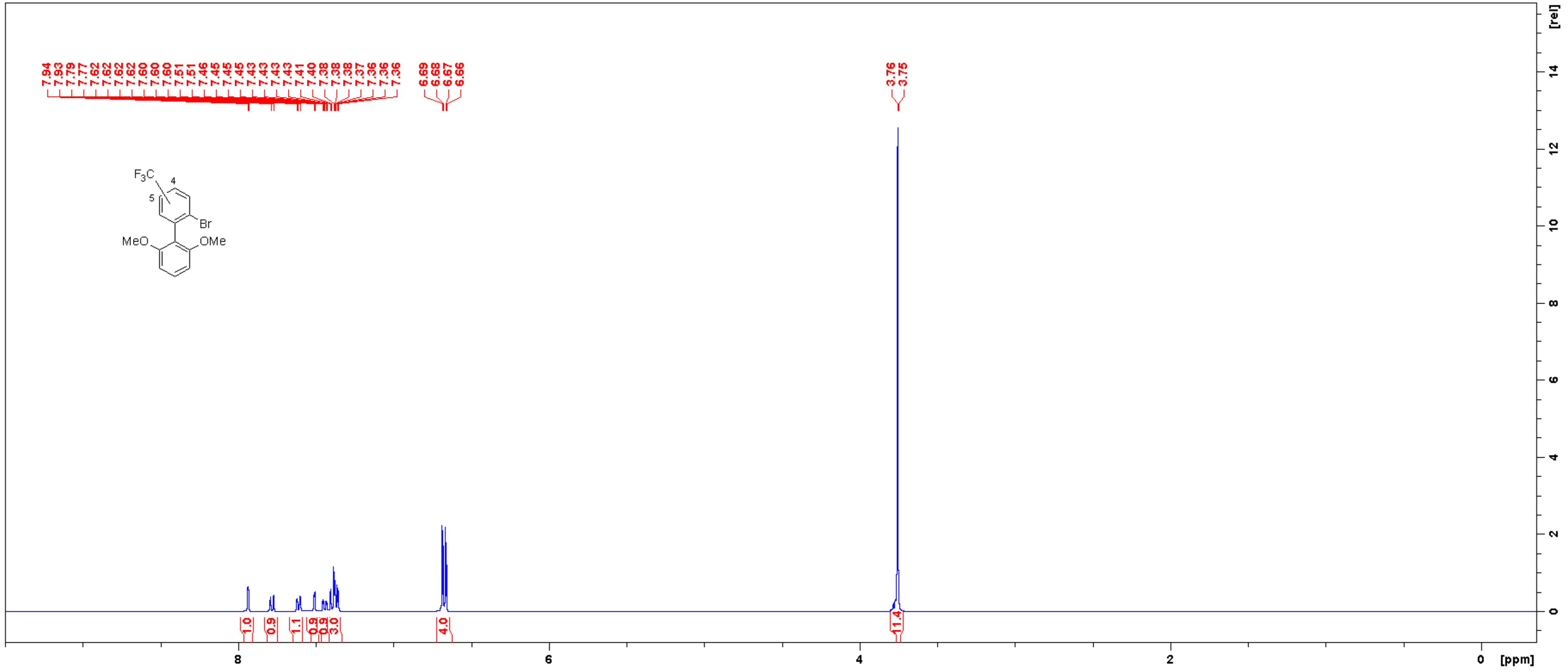

2-Bromo-2',6'-dimethoxy-4-(trifluoromethyl)-1,1'-biphenyl and 2-bromo-2',6'-dimethoxy-5-(trifluoromethyl)-1,1'-biphenyl

$^{13}C\{^1H\}$NMR (CDCl3, 175 MHz)

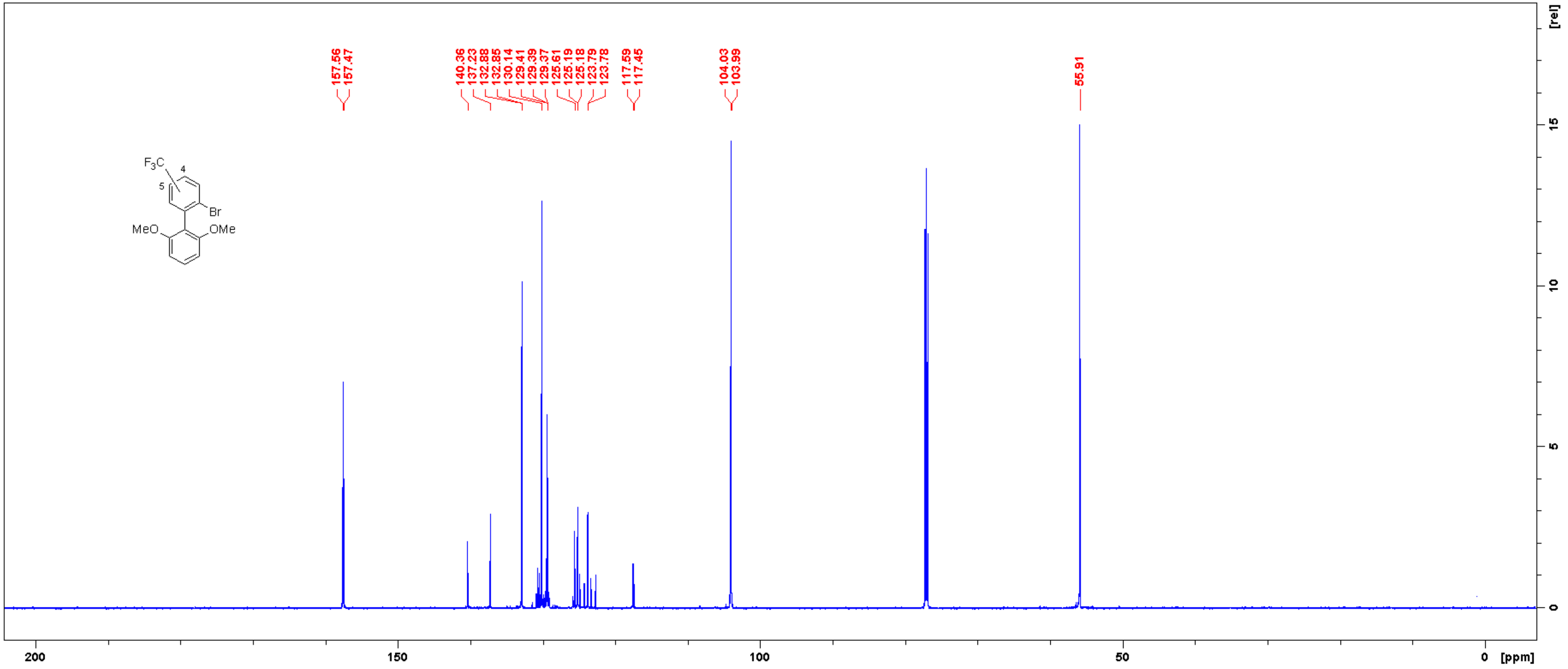

CF$_3$SPhos (I)

^{1}H NMR (CDCl3, 700 MHz)

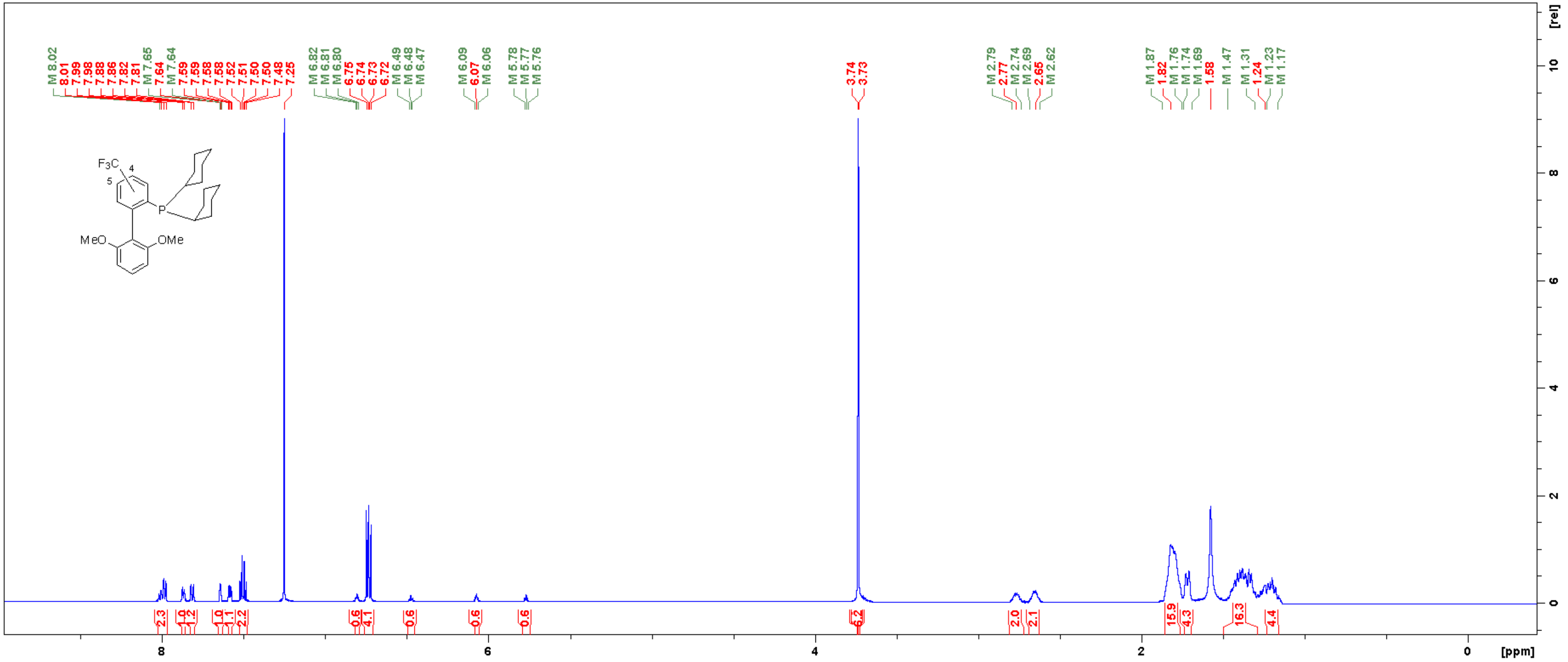

CF$_3$SPhos (I)

$^{13}C\{^1H\}$NMR (CDCl3, 100 MHz)

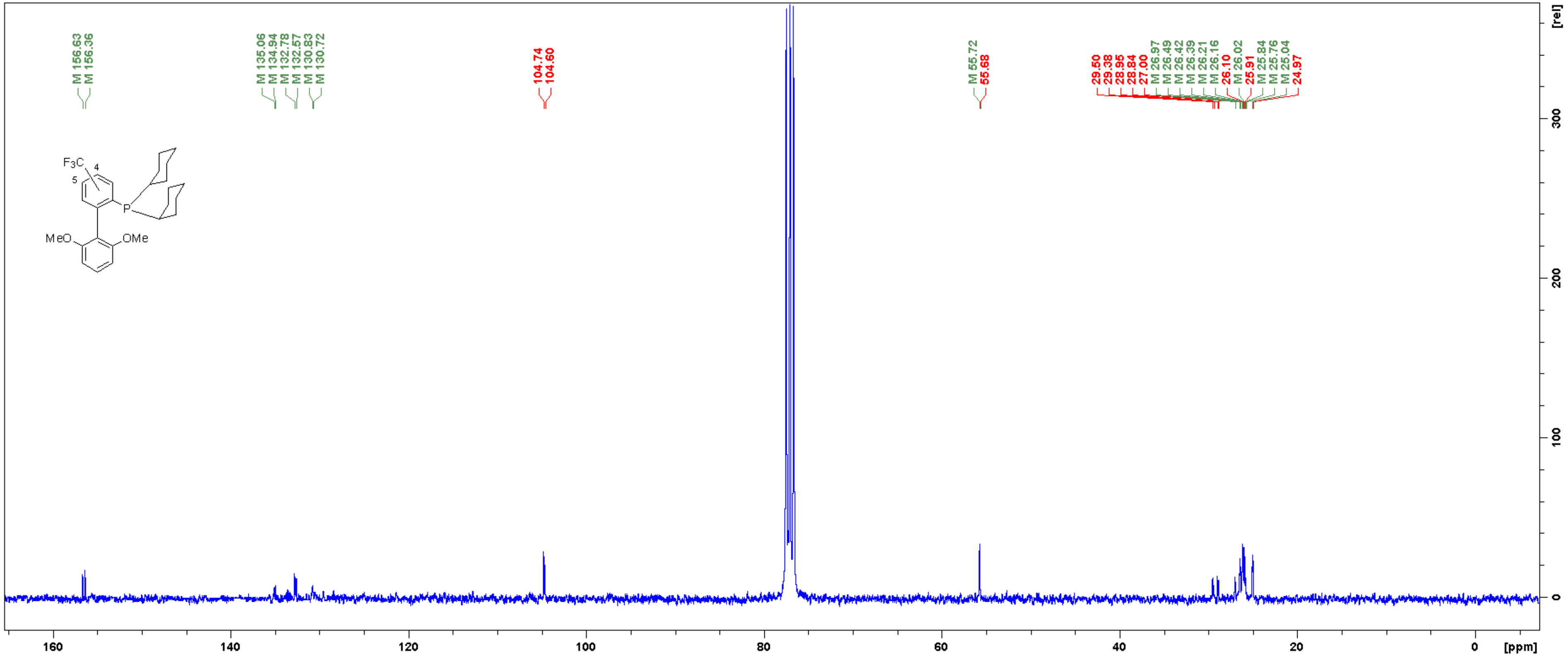

^{i}PrSPhos·HBF_4 (II)

^{1}H NMR (CDCl3, 700 MHz)

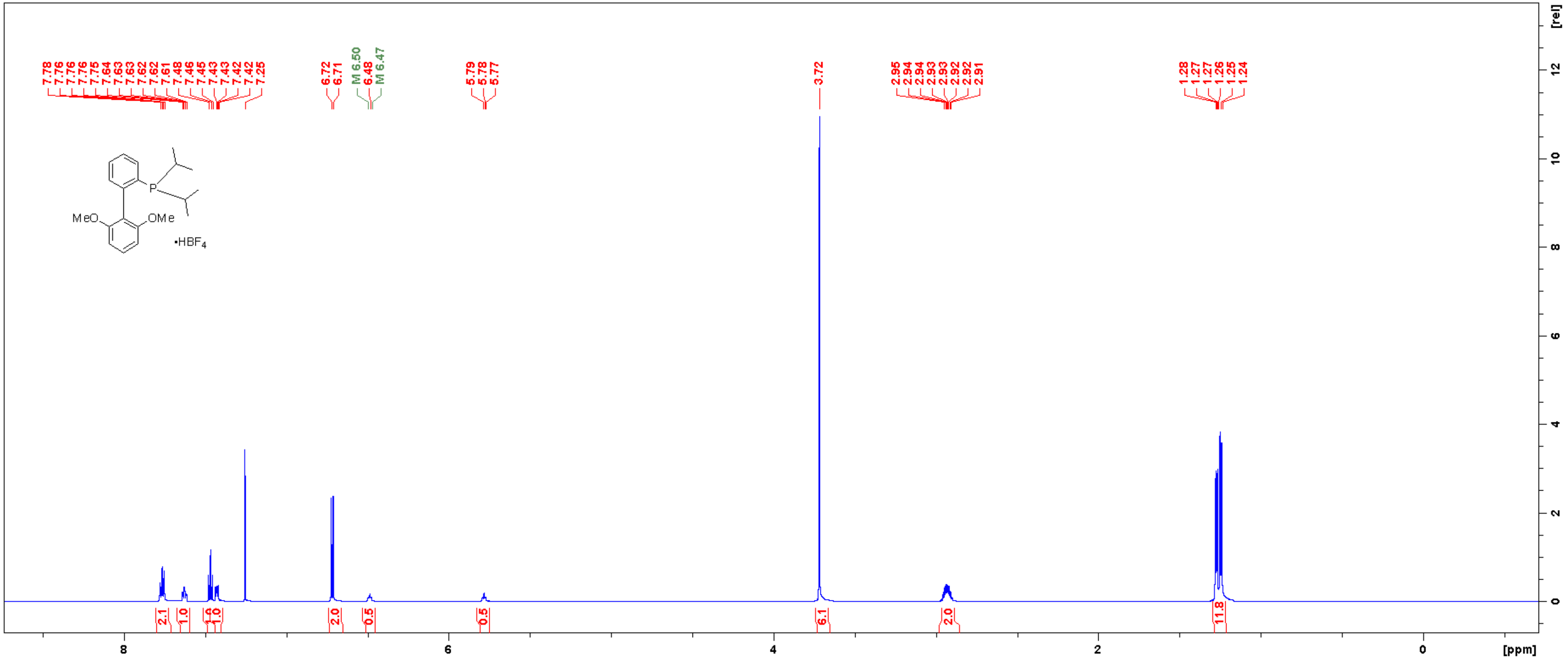

^{i}PrSPhos·HBF_4 (II)

$^{13}C\{^{1}H\}$NMR (CDCl3, 75 MHz)

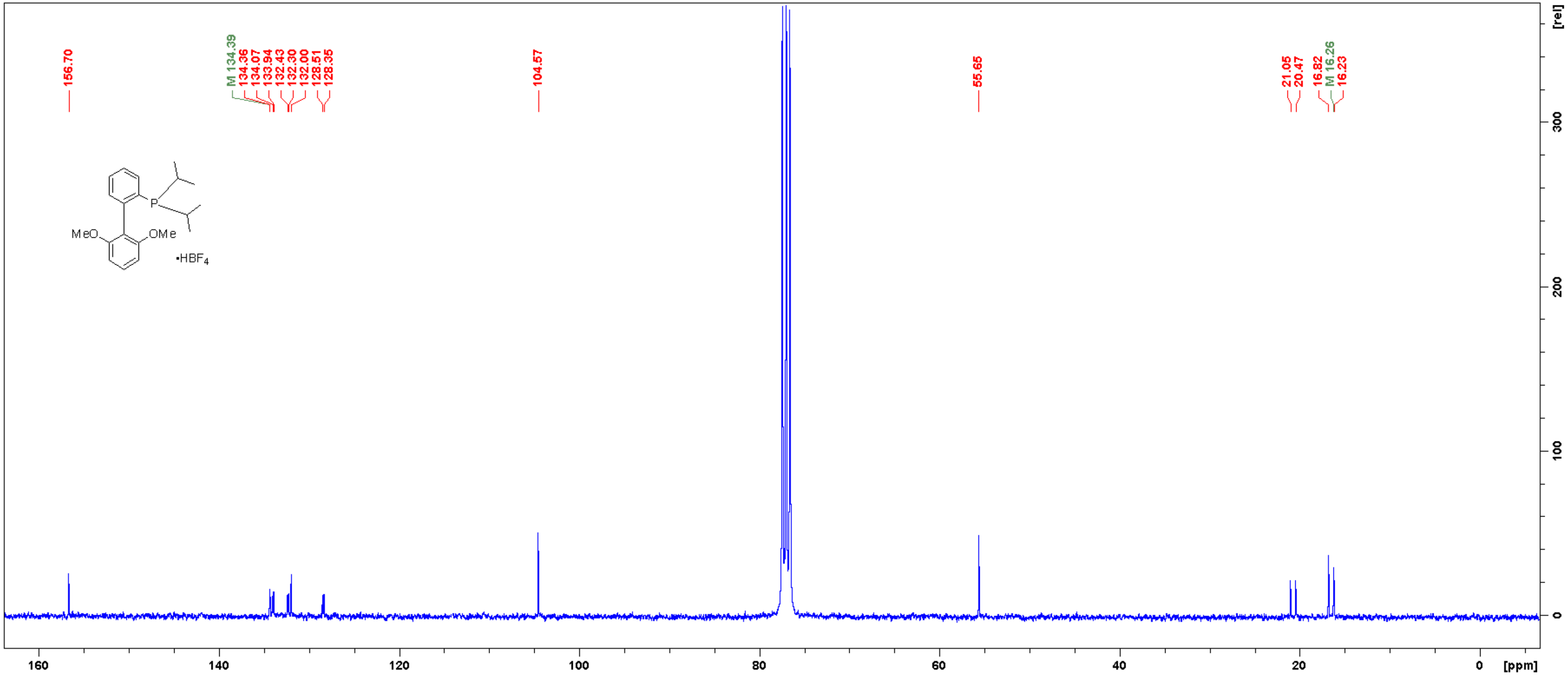

CF$_3$^{i}PrSPhos (III)

^{1}H NMR (CDCl3, 700 MHz)

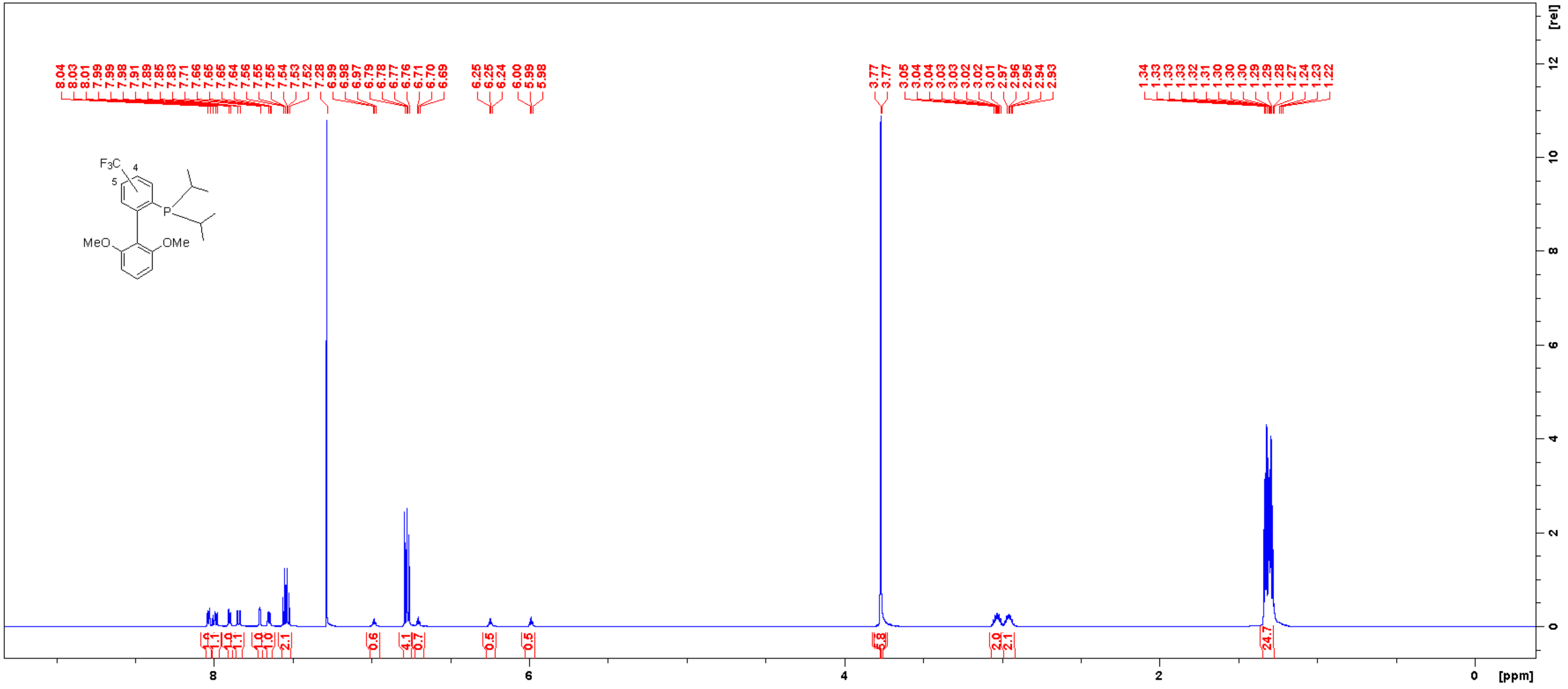

CF$_3$^{i}PrSPhos (III)

^{13}C{^{1}H}NMR (acetone-d6, 100 MHz)

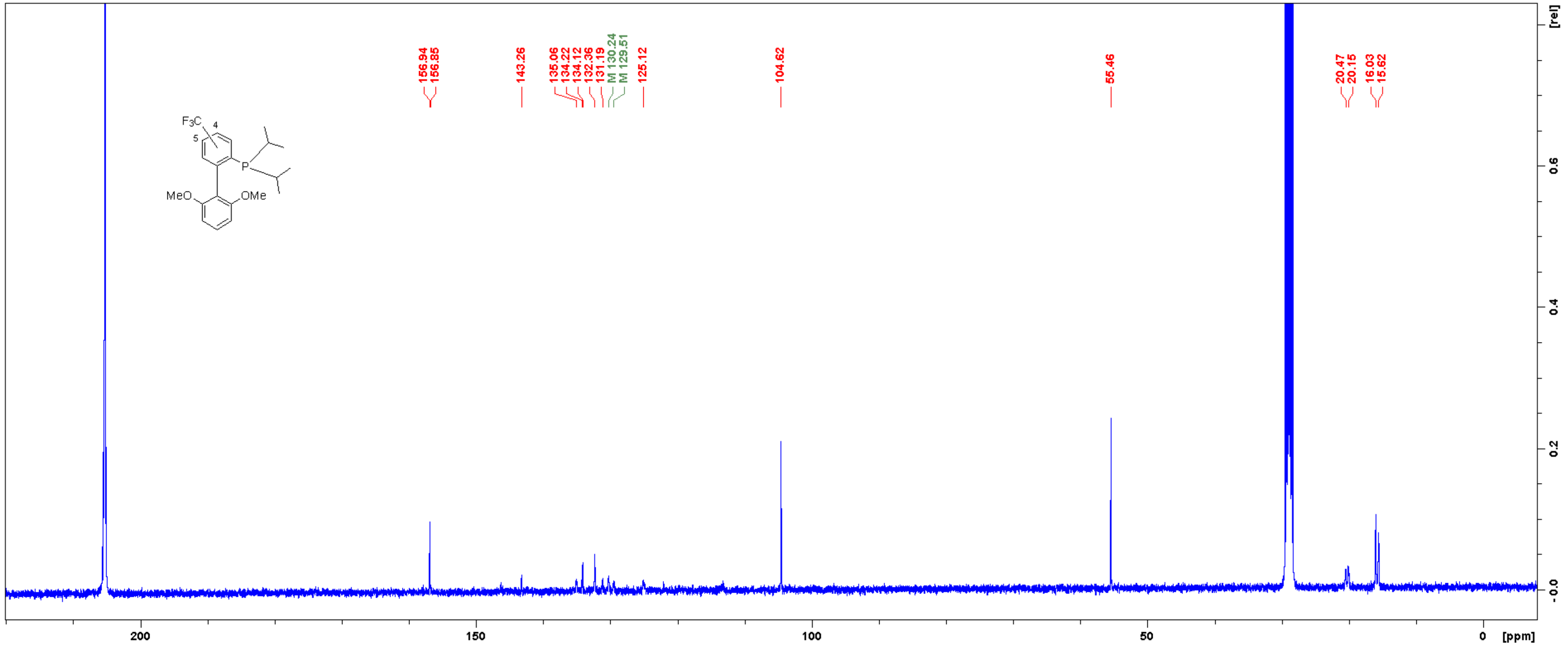

2'-Bromo-*N*²,*N*²,*N*⁶,*N*⁶-tetramethyl-4'-(trifluoromethyl)-[1,1'-biphenyl]-2,6-diamine and 2'-bromo-*N*²,*N*²,*N*⁶,*N*⁶-tetramethyl-5'-(trifluoromethyl)-[1,1'-biphenyl]-2,6-diamine

^{1}H NMR (CDCl3, 700 MHz)

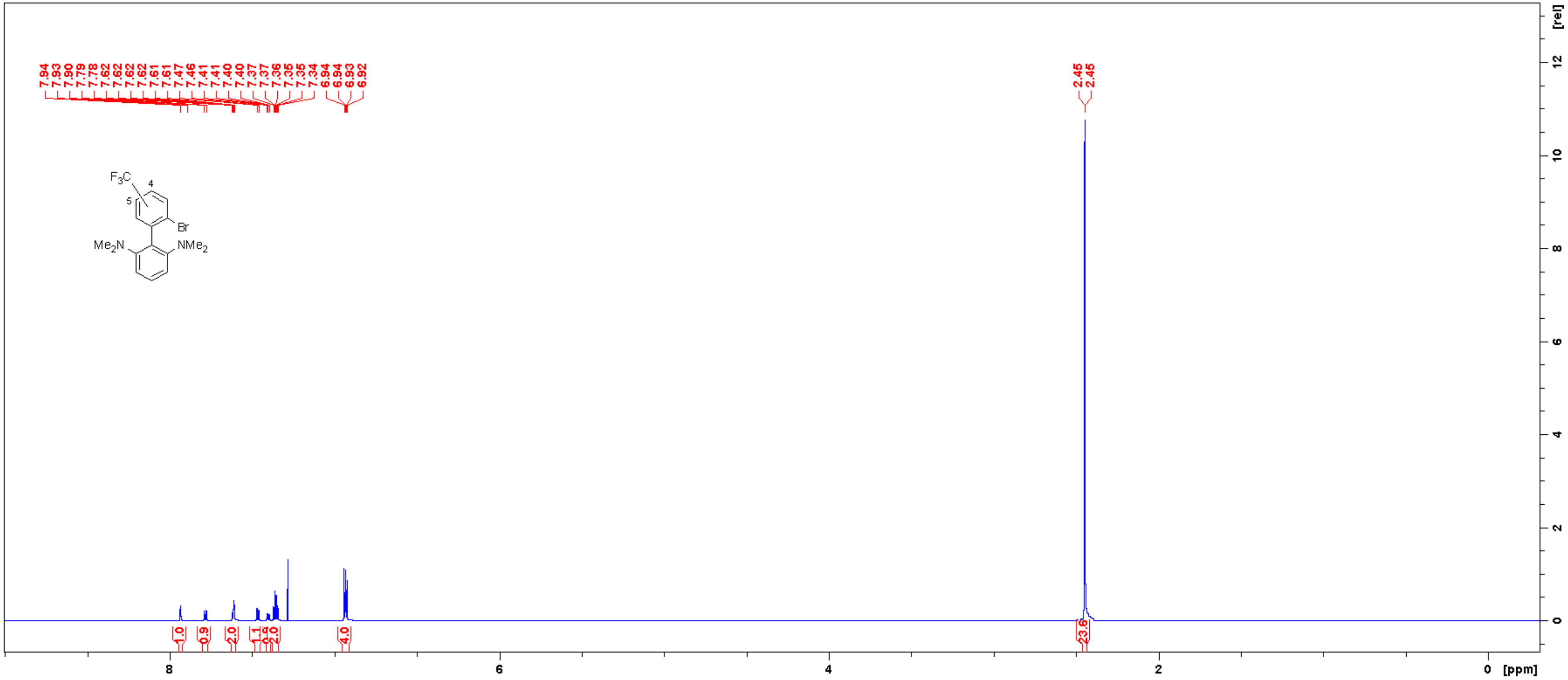

2'-Bromo-N^2,N^2,N^6,N^6-tetramethyl-4'-(trifluoromethyl)-[1,1'-biphenyl]-2,6-diamine and 2'-bromo-N^2,N^2,N^6,N^6-tetramethyl-5'-(trifluoromethyl)-[1,1'-biphenyl]-2,6-diamine

^{13}C{^{1}H}NMR (CDCl3, 100 MHz)

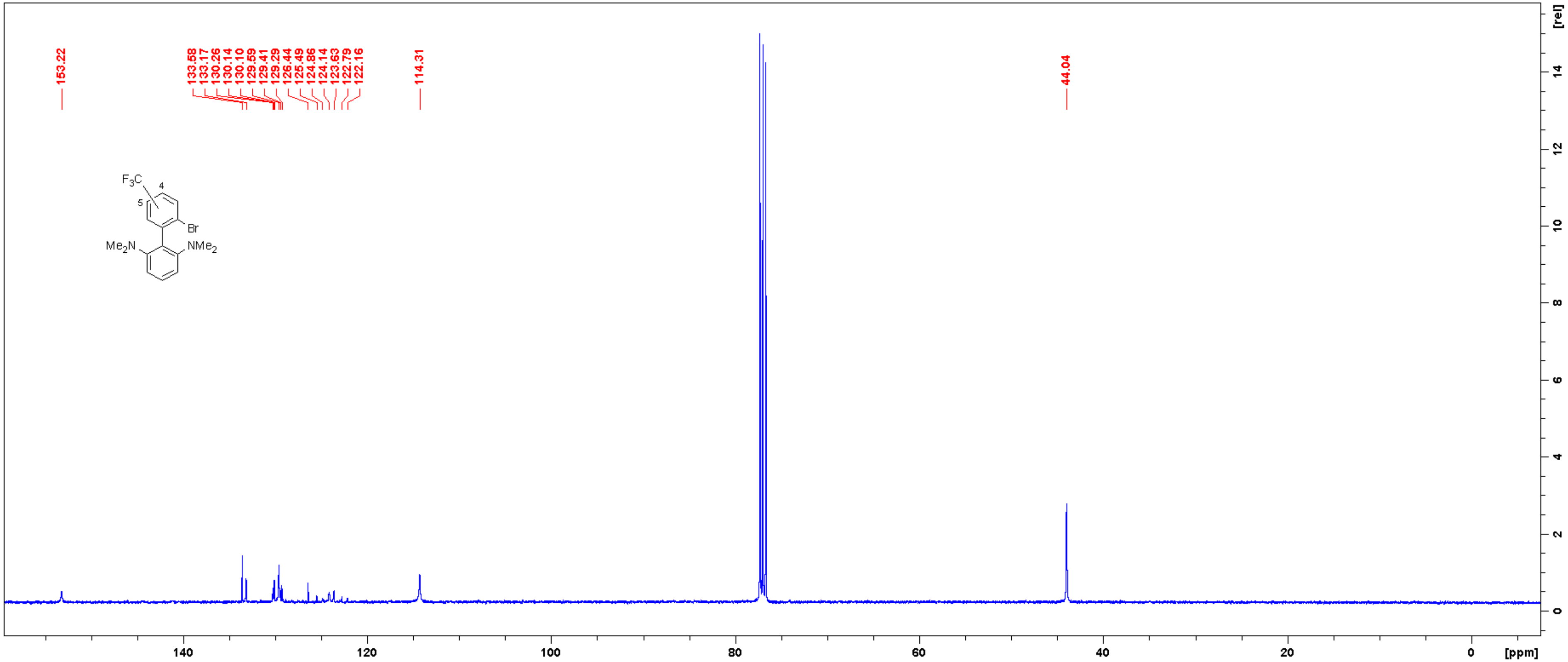

CF$_3$CPhos (IV)

^{1}H NMR (acetone-d6, 400 MHz)

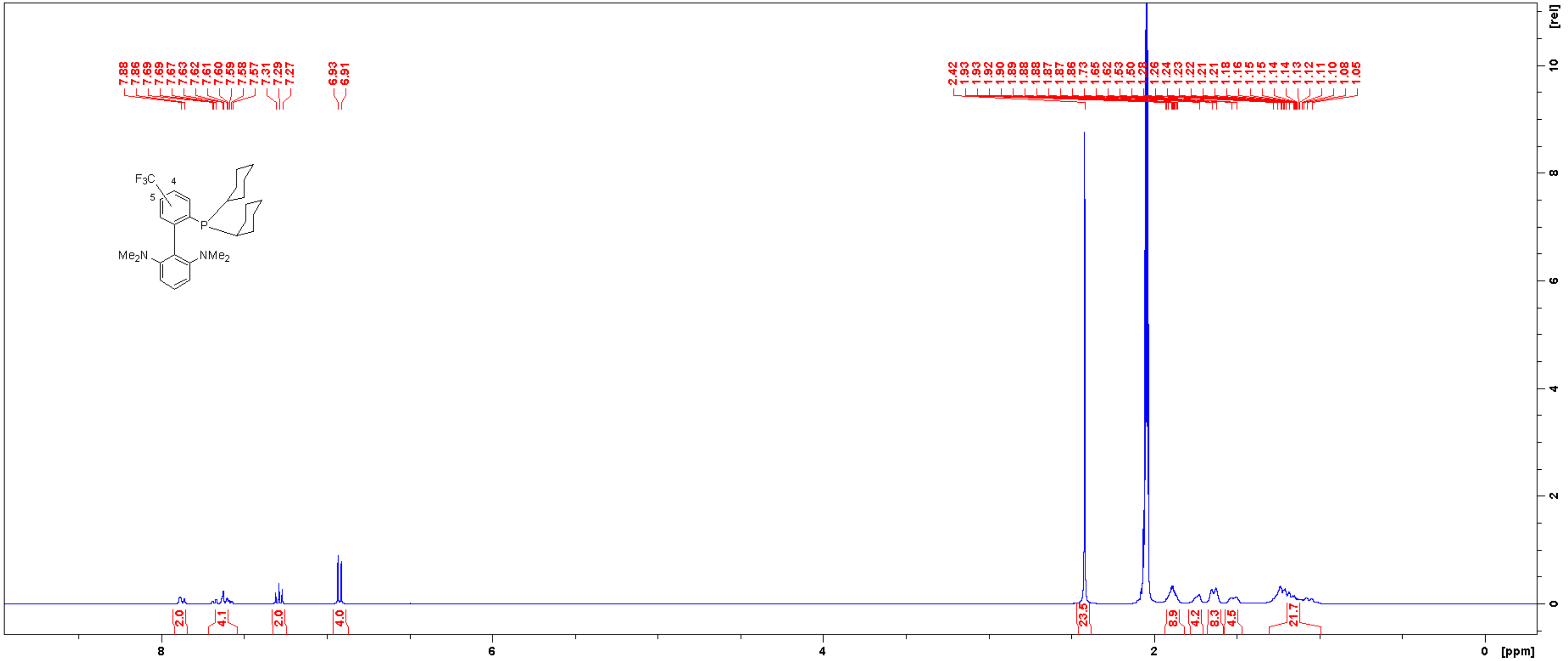

CF$_3$CPhos (IV)

$^{13}C\{^1H\}$NMR (CDCl3, 100 MHz)

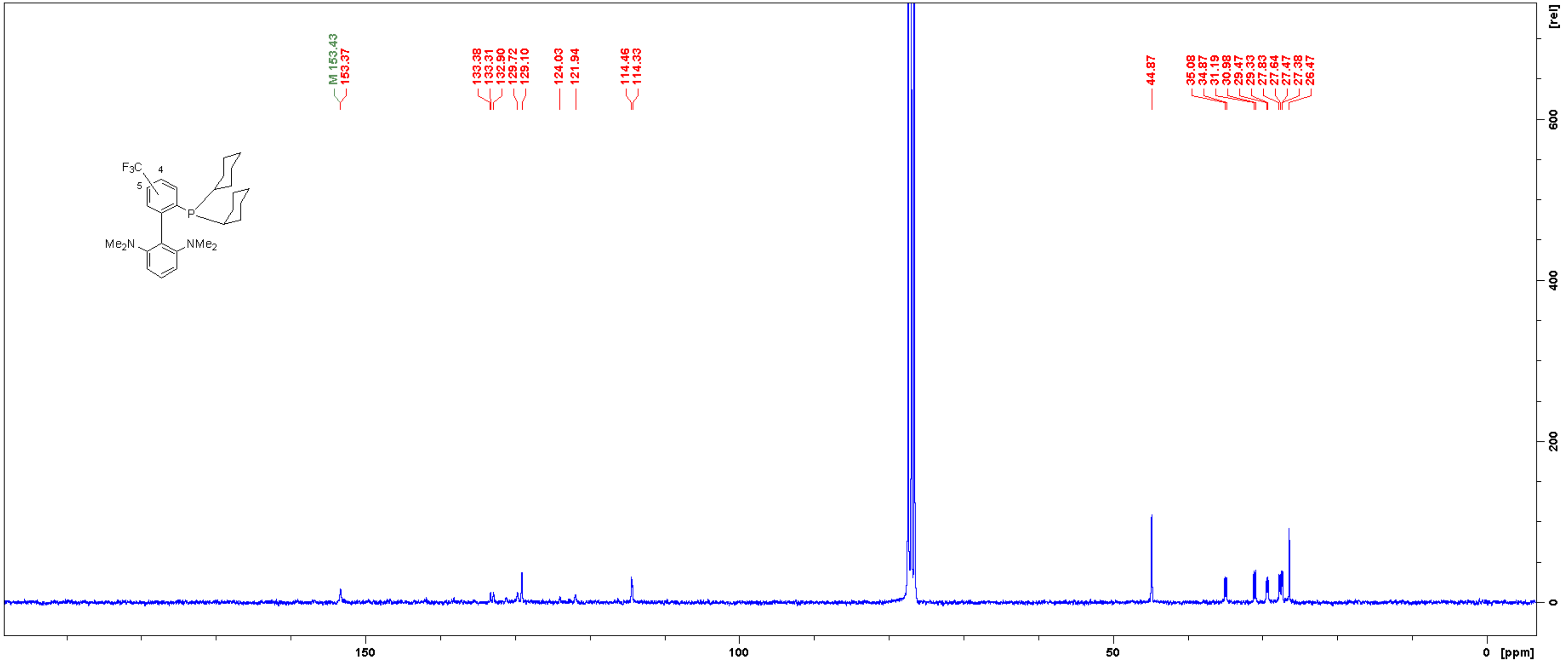

^{i}PrCPhos·HBF_4 (V)

^{1}H NMR (MeOD, 400 MHz)

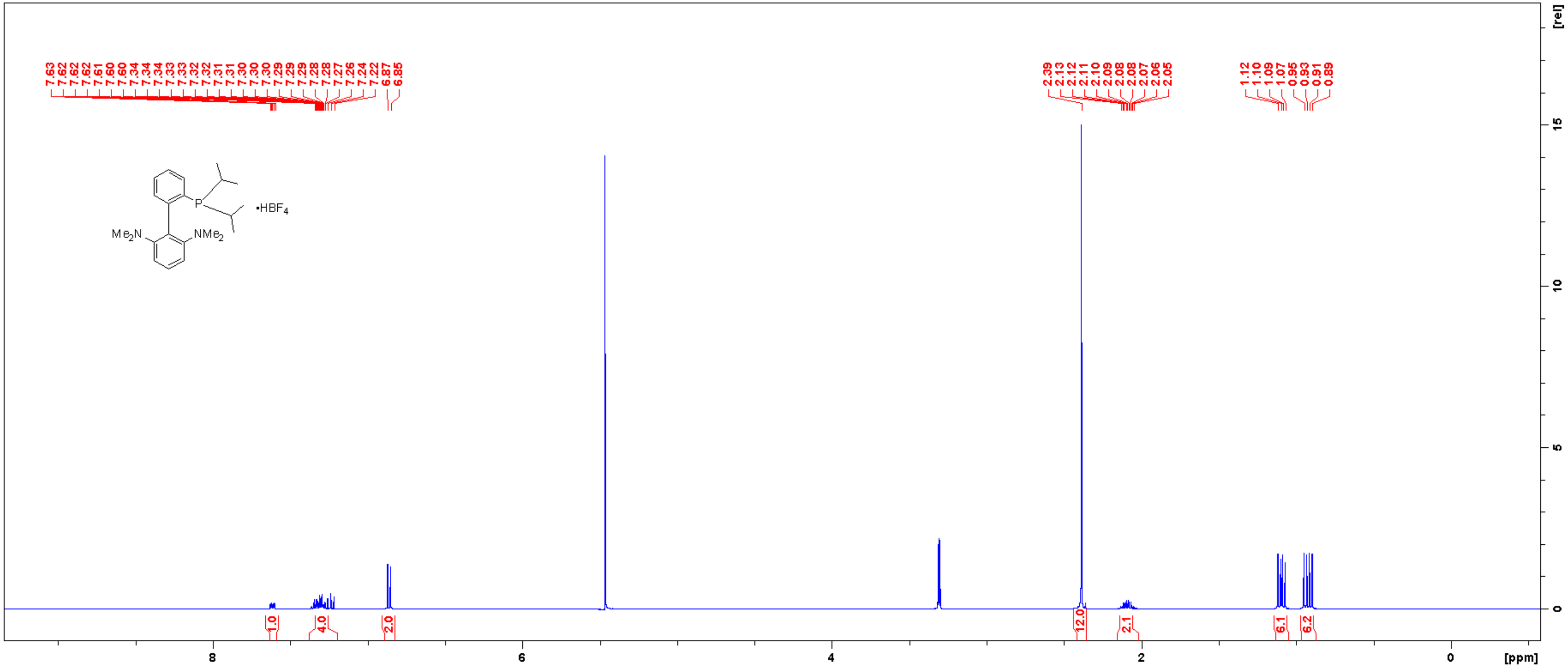

^{i}PrCPhos·HBF_4 (V)

$^{13}C\{^{1}H\}$NMR (MeOD, 100 MHz)

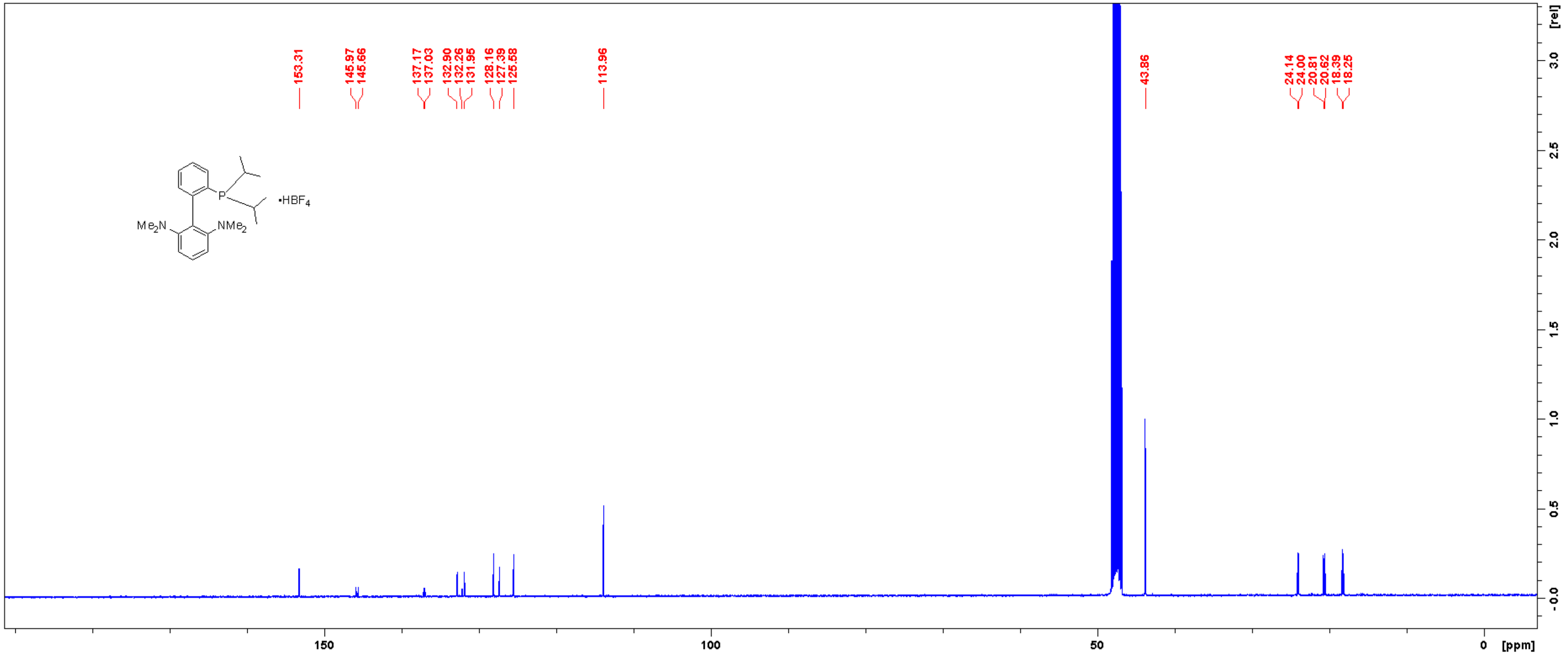

CF$_3$^{i}PrCPhos·HBF$_4$ (VI)

^{1}H NMR (MeOD, 400 MHz)

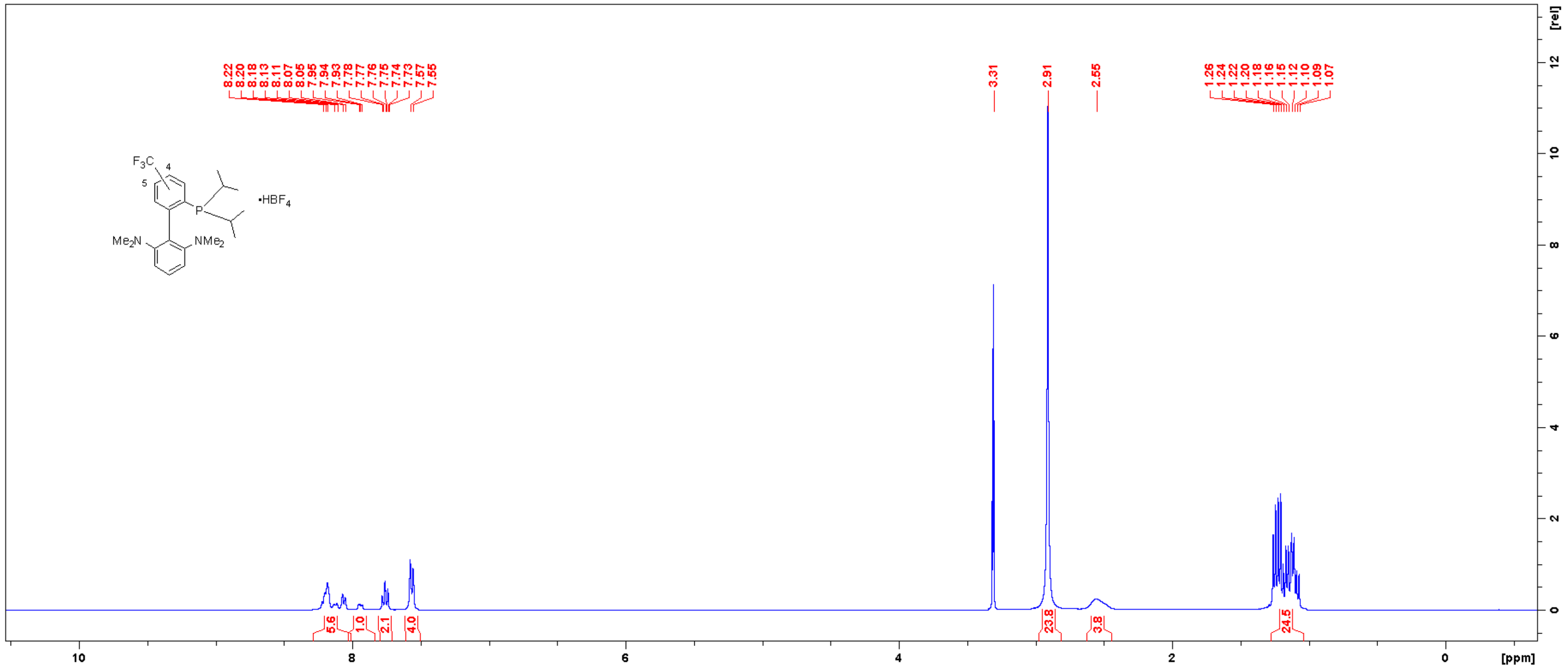

CF$_3{}^i$PrCPhos·HBF$_4$ (VI)

^{13}C{^{1}H}NMR (acetone-d6, 75 MHz)

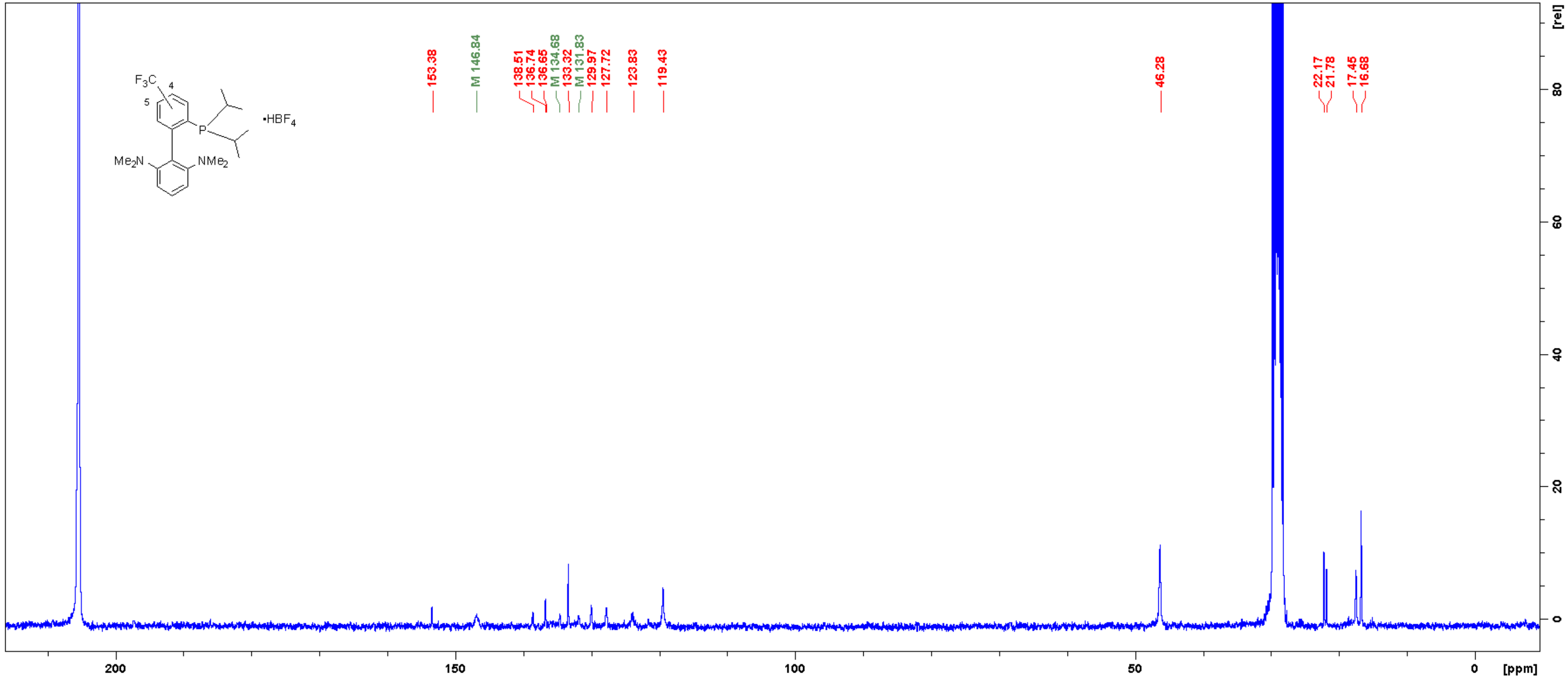

CF$_3$XPhos (VII)

^{1}H NMR (CDCl3, 700 MHz)

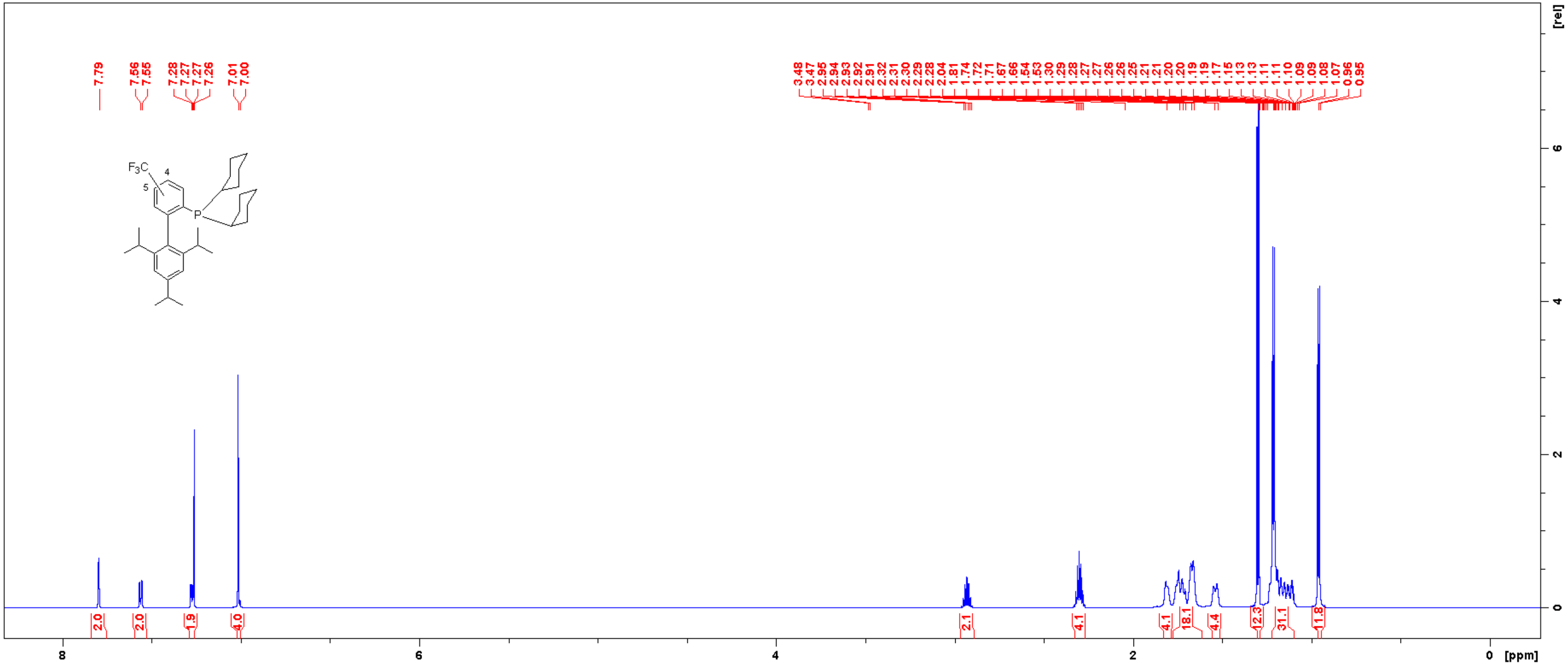

CF$_3$XPhos (VII)

$^{13}C\{^1H\}$NMR (CDCl3, 75 MHz)

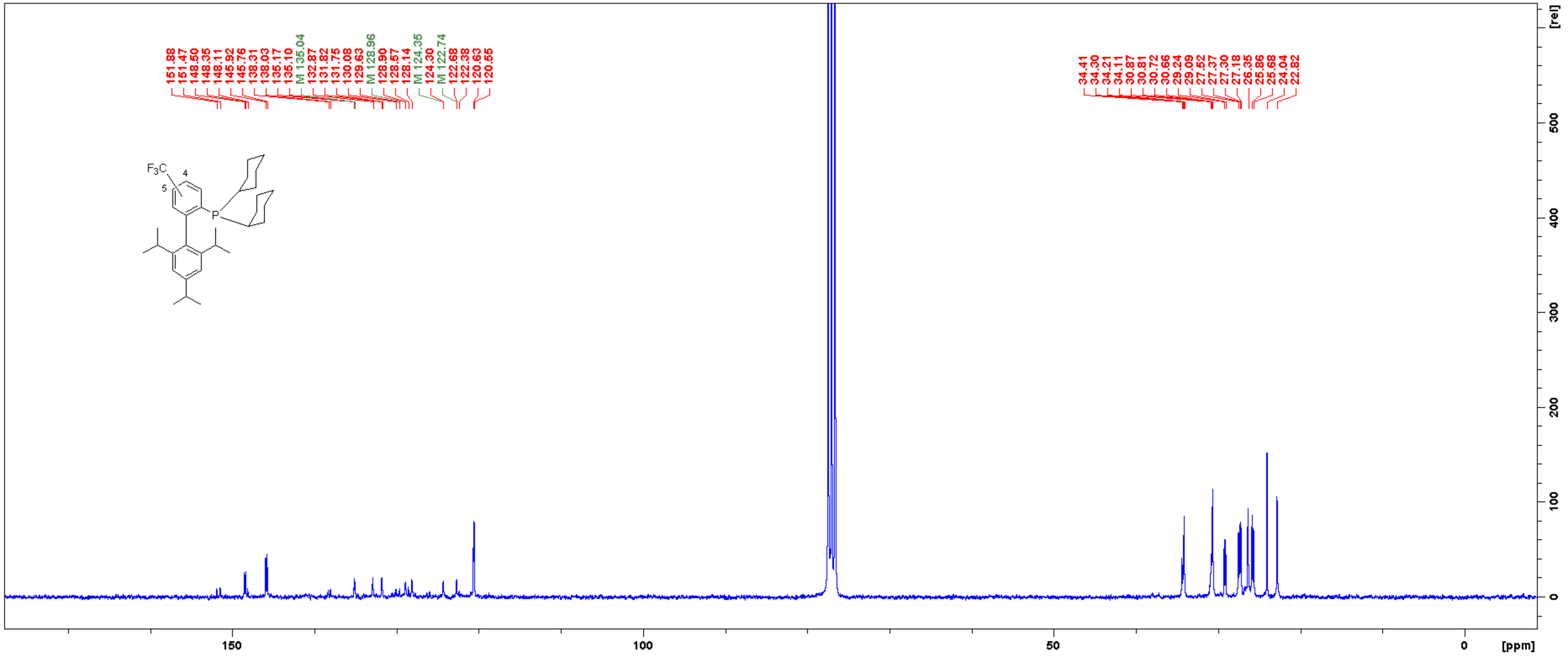

^{i}PrXPhos·HBF_4 (VIII)

^{1}H NMR (DMSO-d6, 400 MHz)

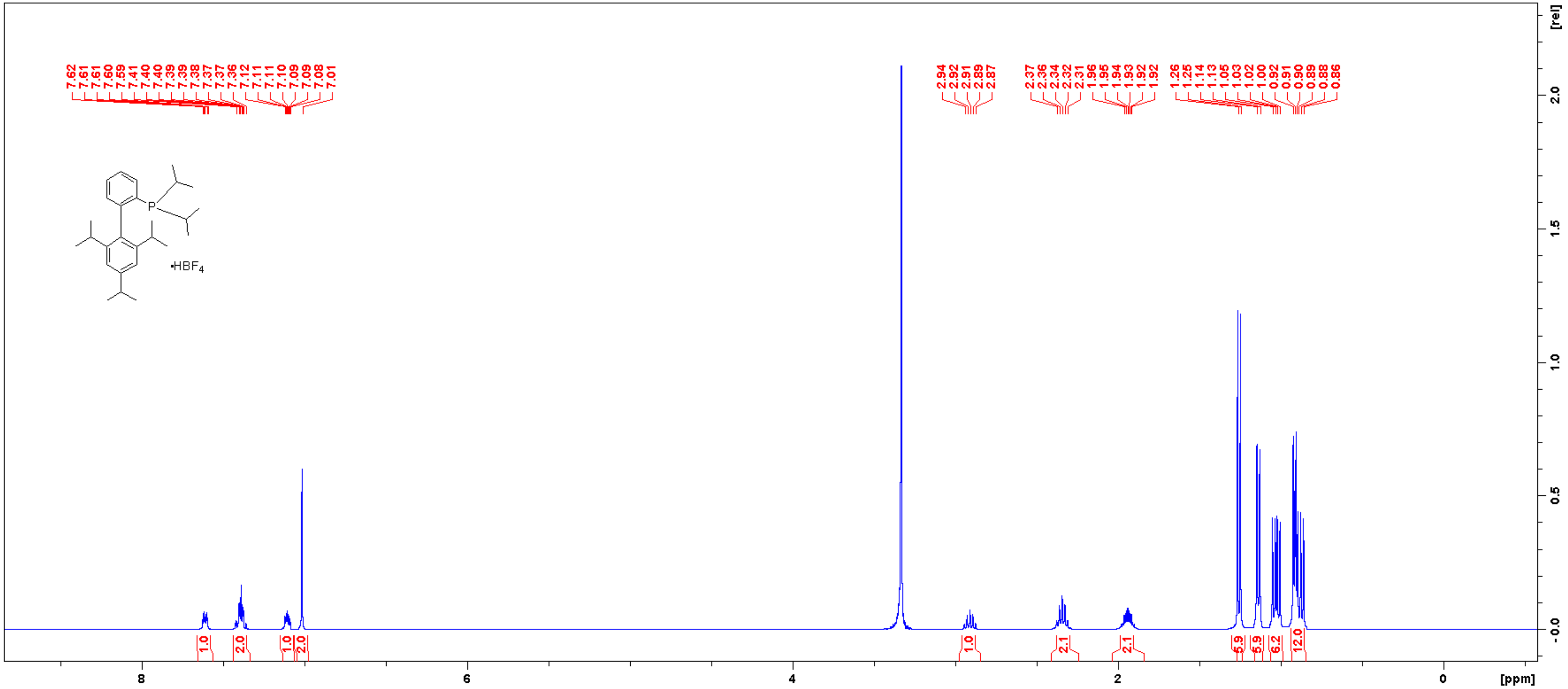

^{i}PrXPhos·HBF_4 (VIII)

$^{13}C\{^{1}H\}$NMR (DMSO-d6,100 MHz)

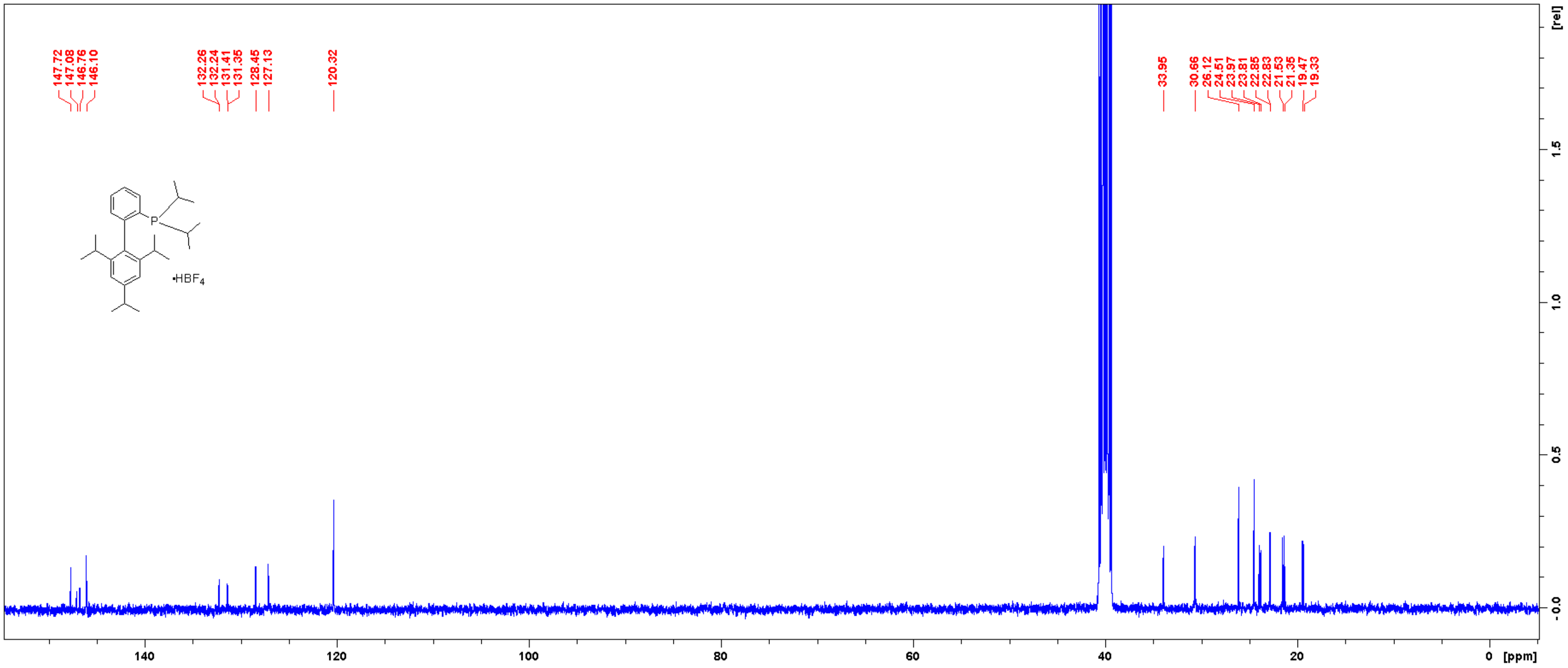

***p*-toluidine (2a)**

^{1}H NMR ($CDCl_3$, 400MHz)

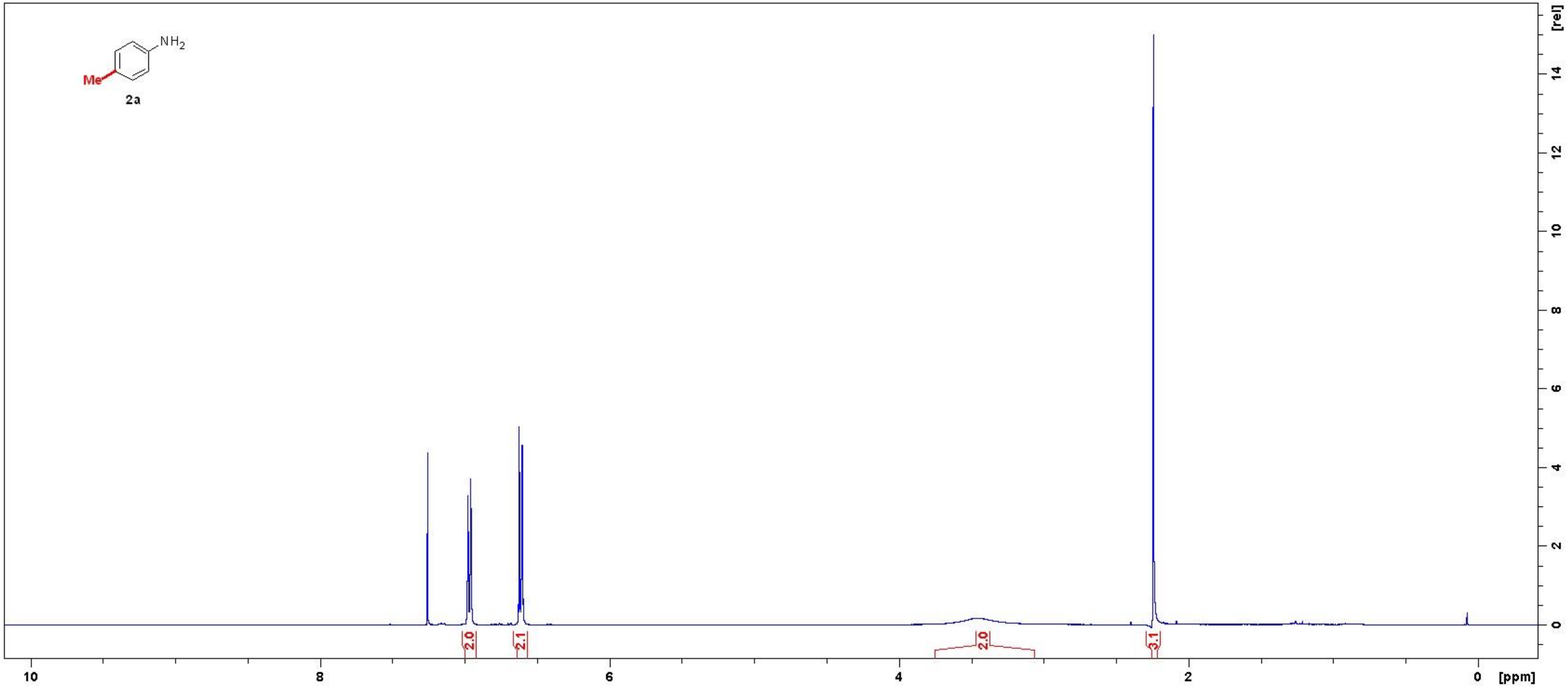

***p*-toluidine (2a)**

$^{13}C\{^{1}H\}$NMR ($CDCl_3$, 100MHz)

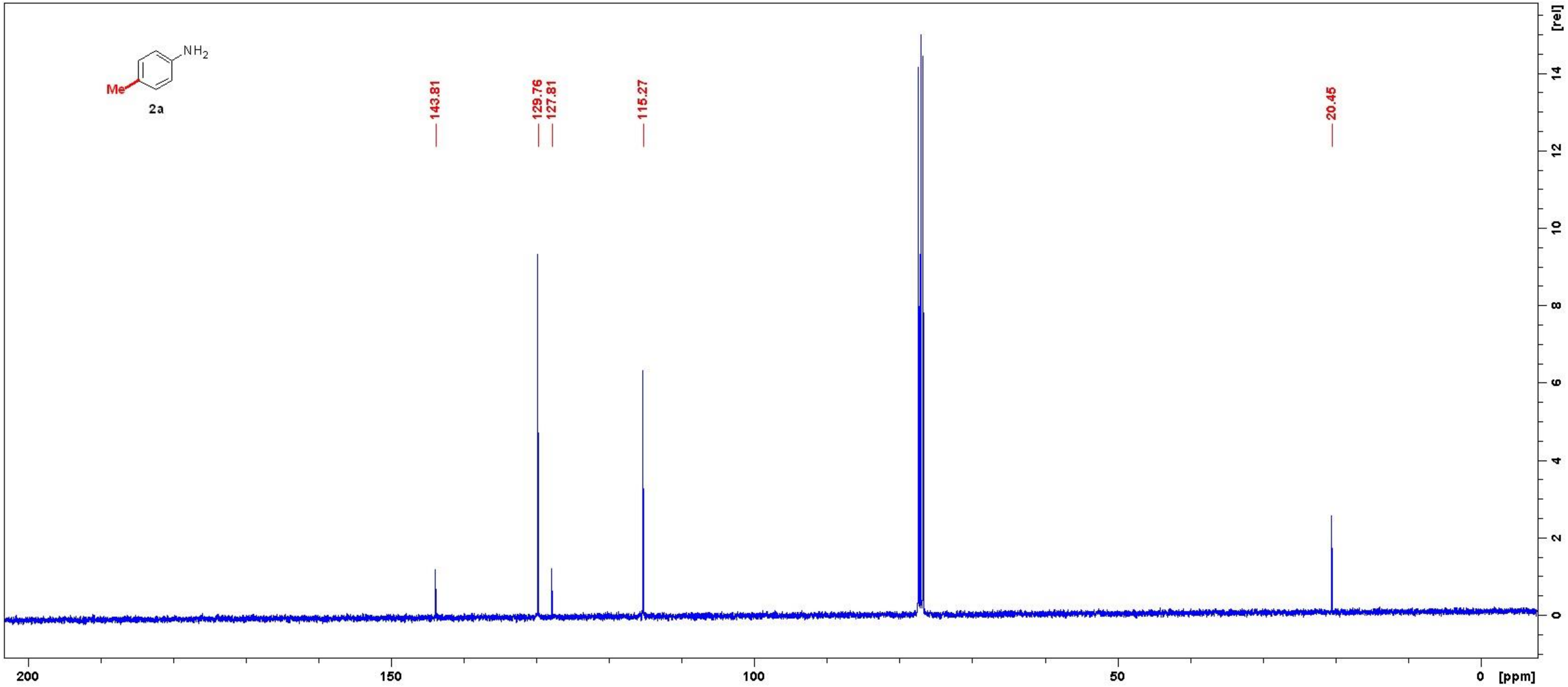

4-butylaniline (2b)

^{1}H NMR ($CDCl_3$, 400MHz)

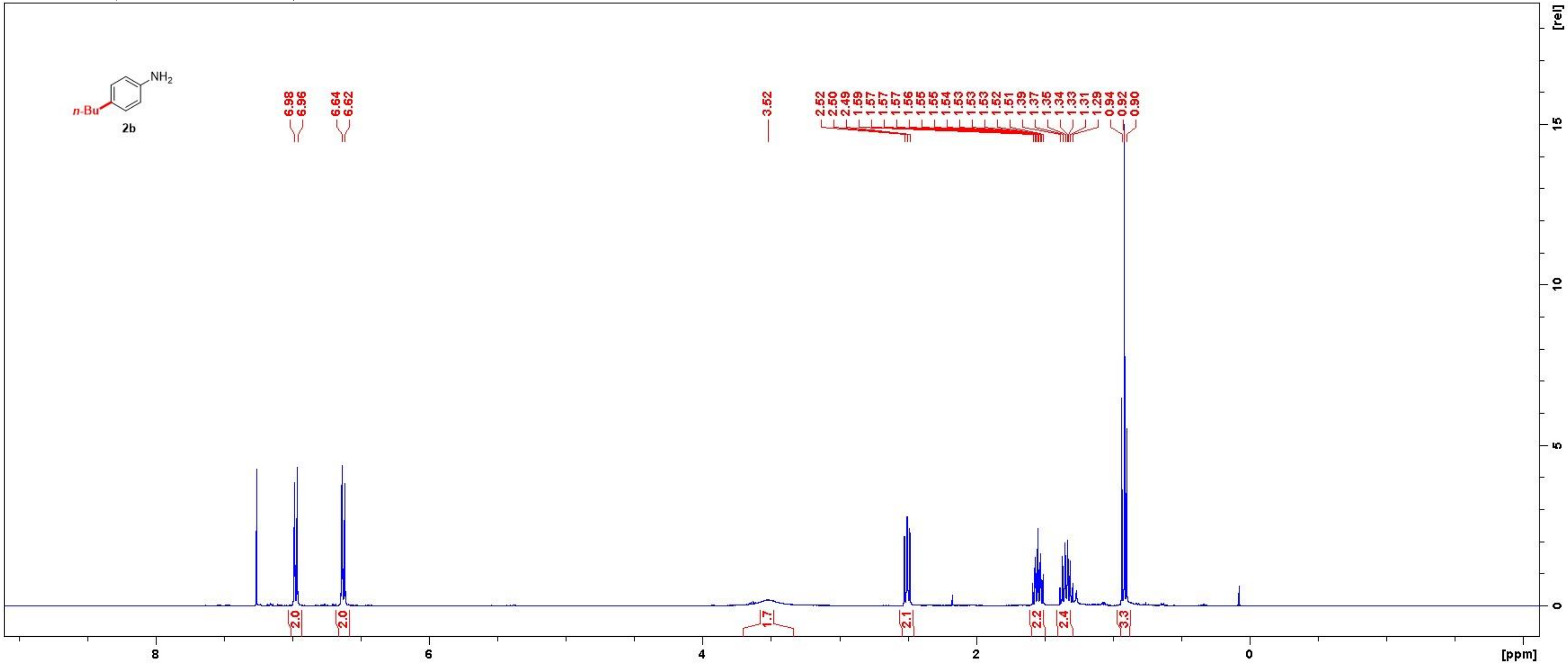

4-butylaniline (2b)

$^{13}C\{^1H\}$NMR ($CDCl_3$, 100MHz)

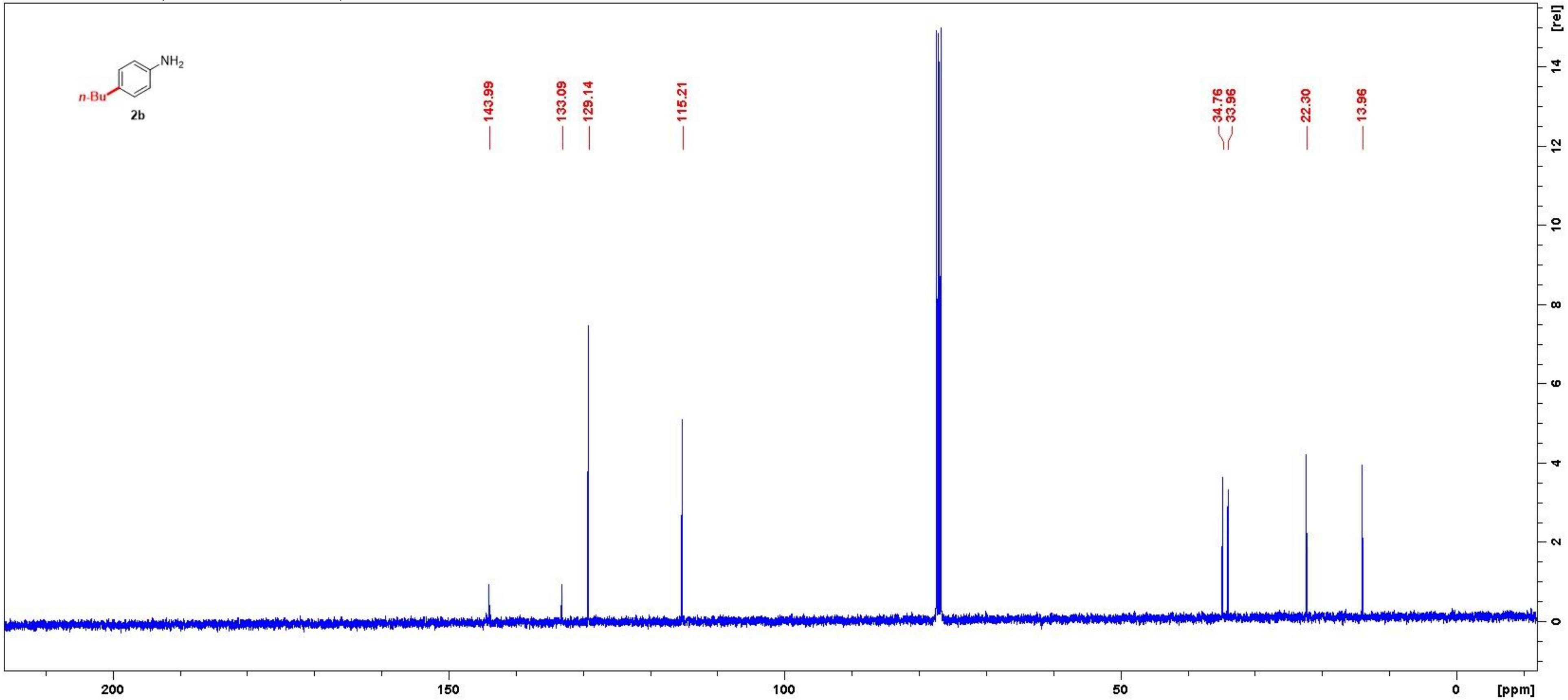

4-isopropylaniline (3)

^{1}H NMR ($CDCl_3$, 400MHz)

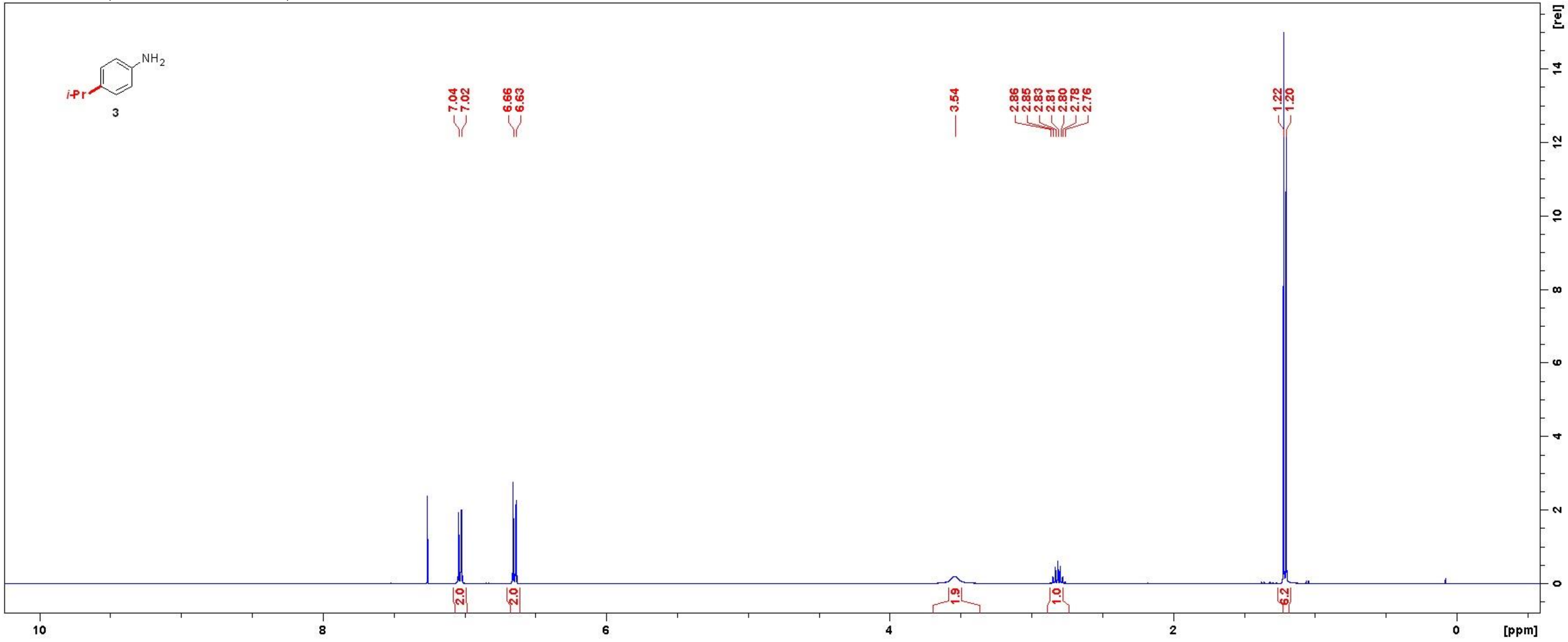

4-isopropylaniline (3)

$^{13}C\{^1H\}$NMR NMR ($CDCl_3$, 100MHz)

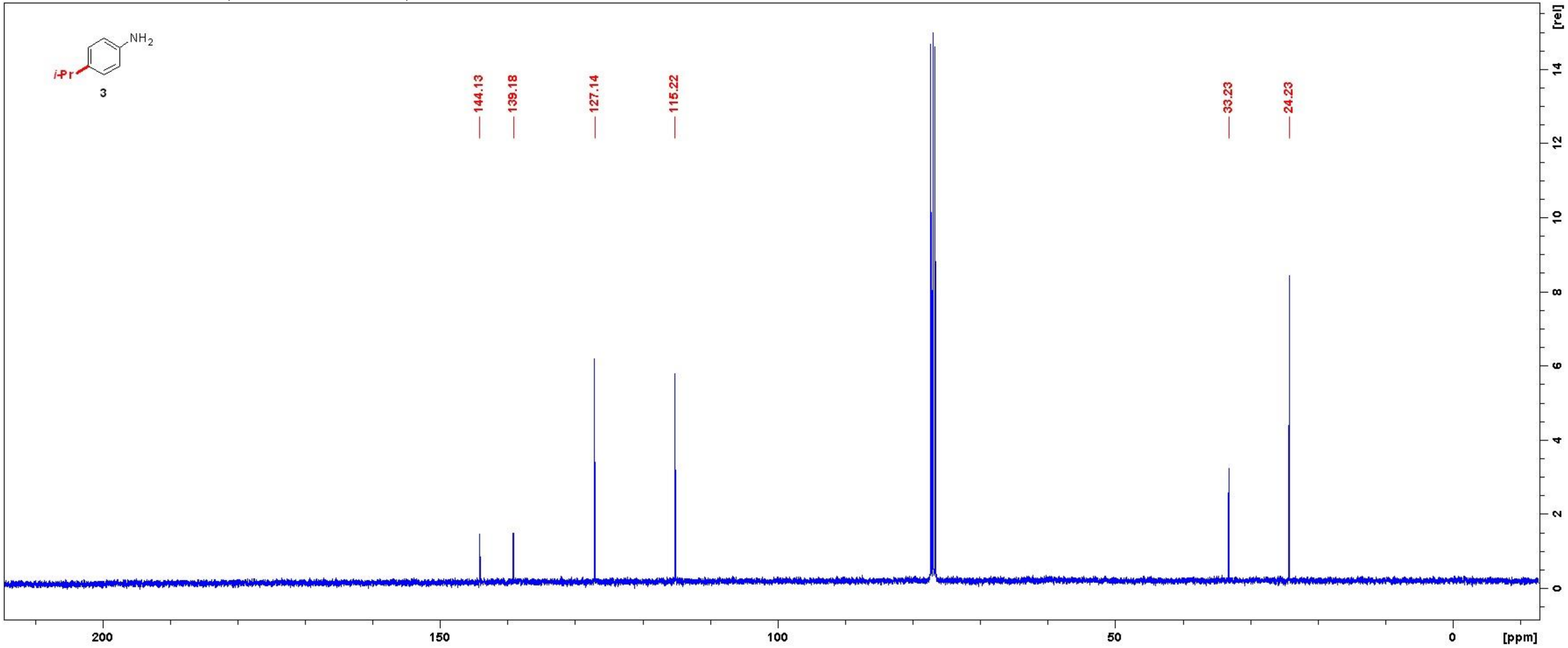

4-(*tert*-butyl)aniline (4)

^{1}H NMR ($CDCl_3$, 400MHz)

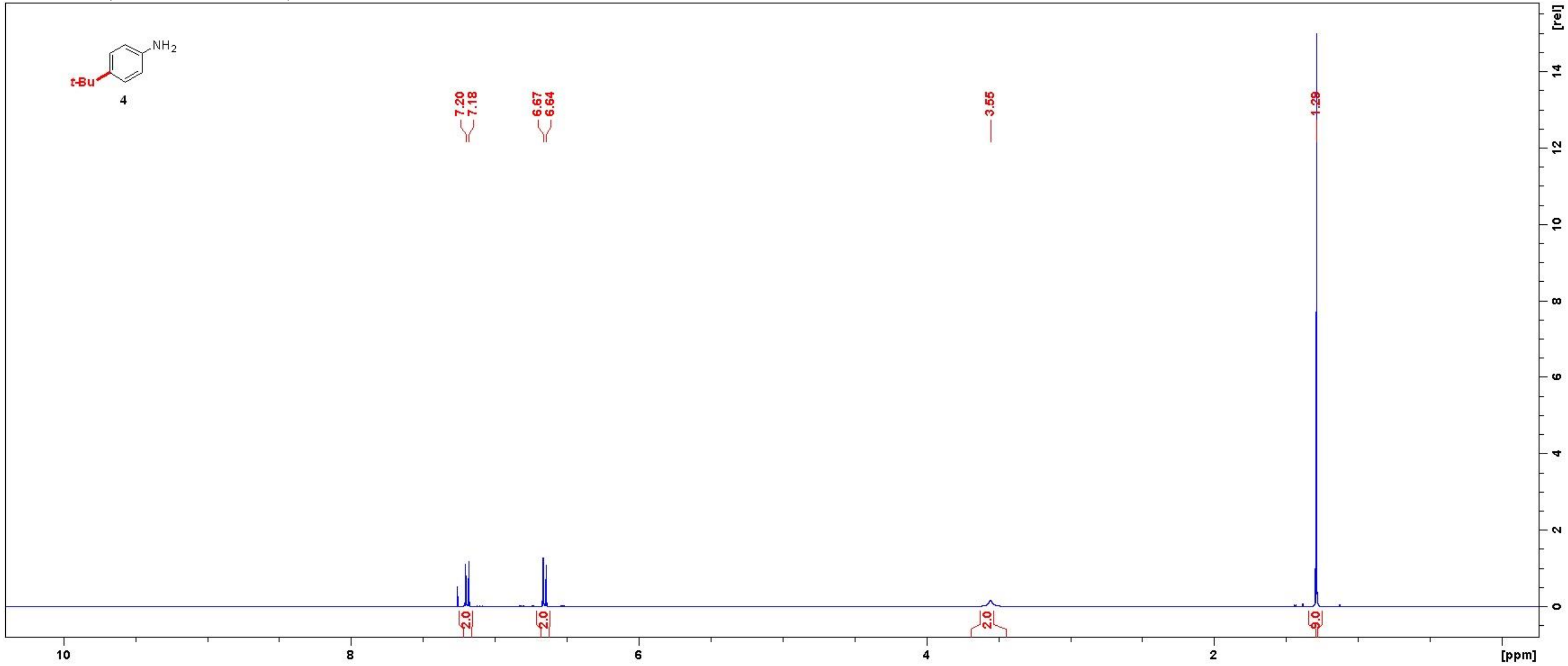

4-(*tert*-butyl)aniline (4)

$^{13}C\{^{1}H\}$NMR NMR ($CDCl_3$, 100MHz)

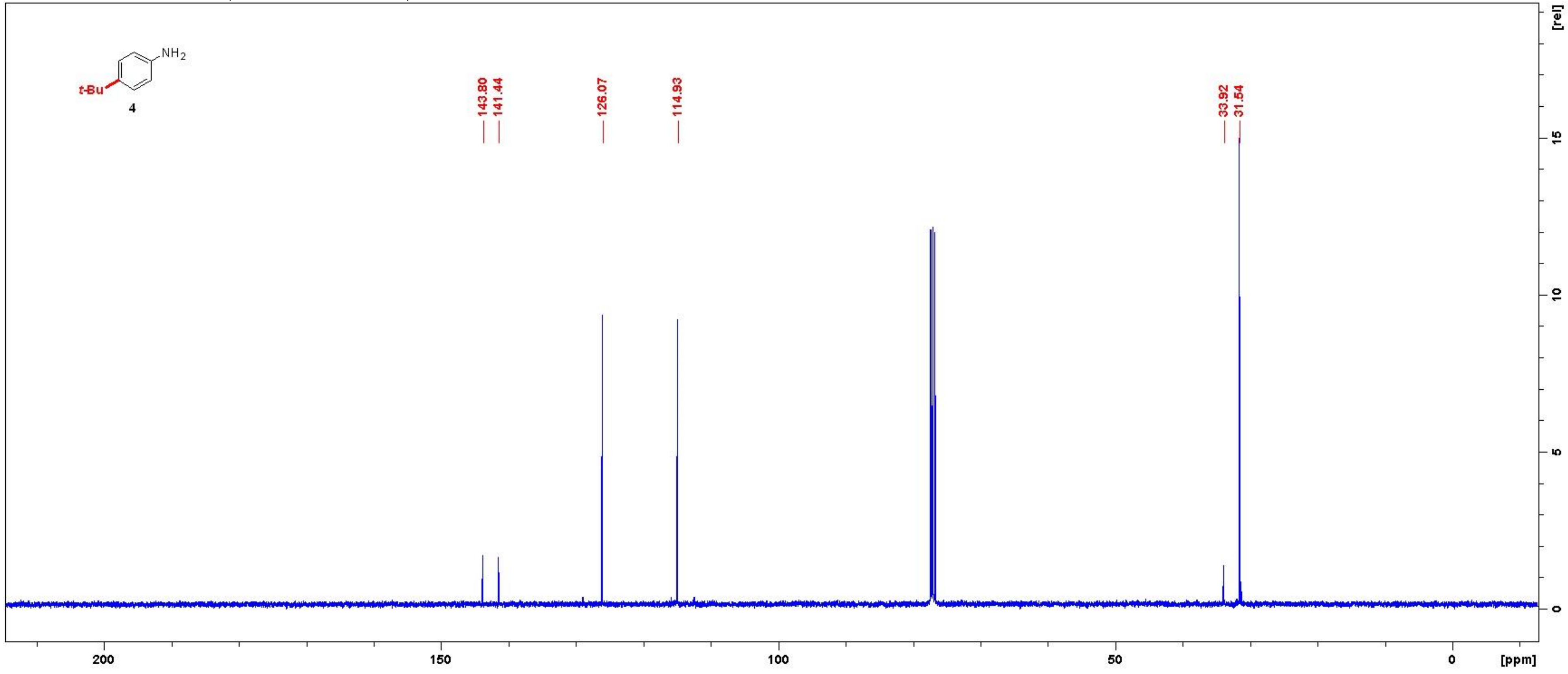

***m*-toluidine (5)**

^{1}H NMR ($CDCl_3$, 400MHz)

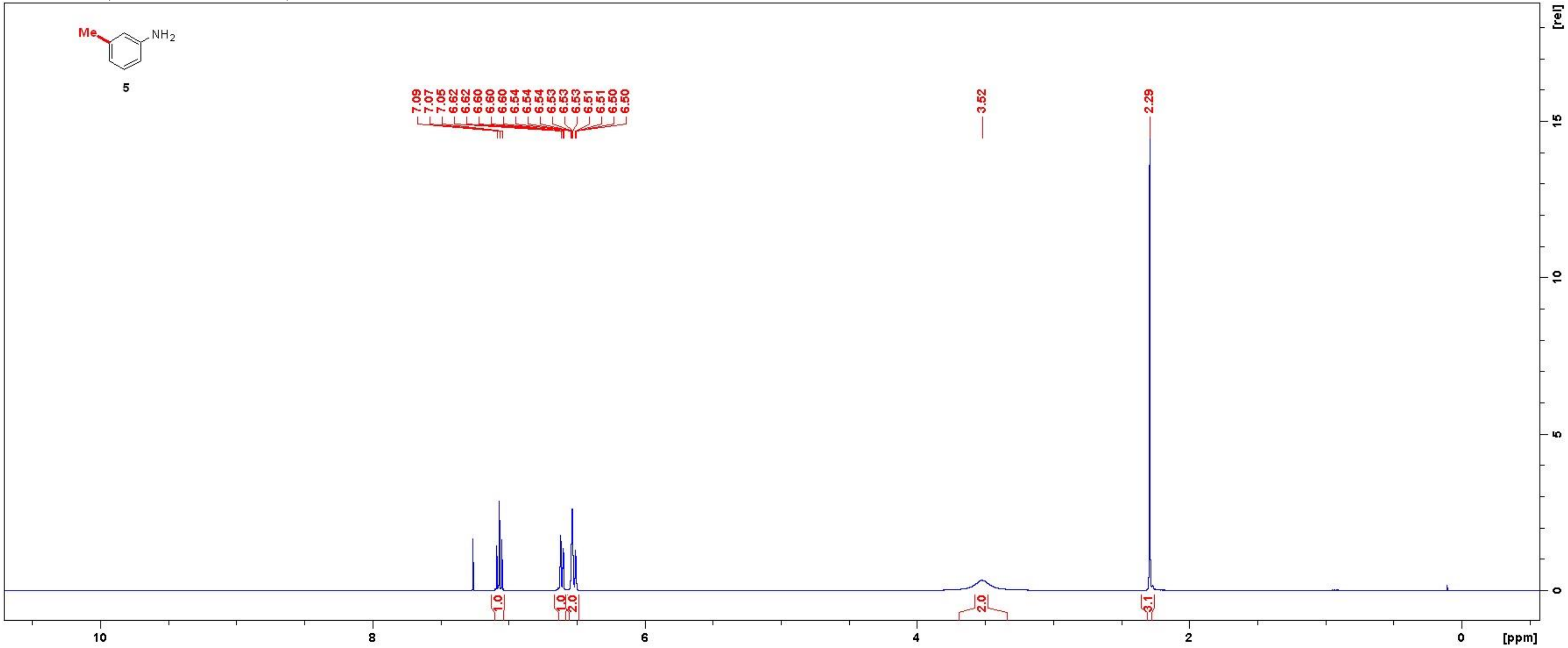

***m*-toluidine (5)**

$^{13}C\{^{1}H\}$NMR ($CDCl_3$, 100MHz)

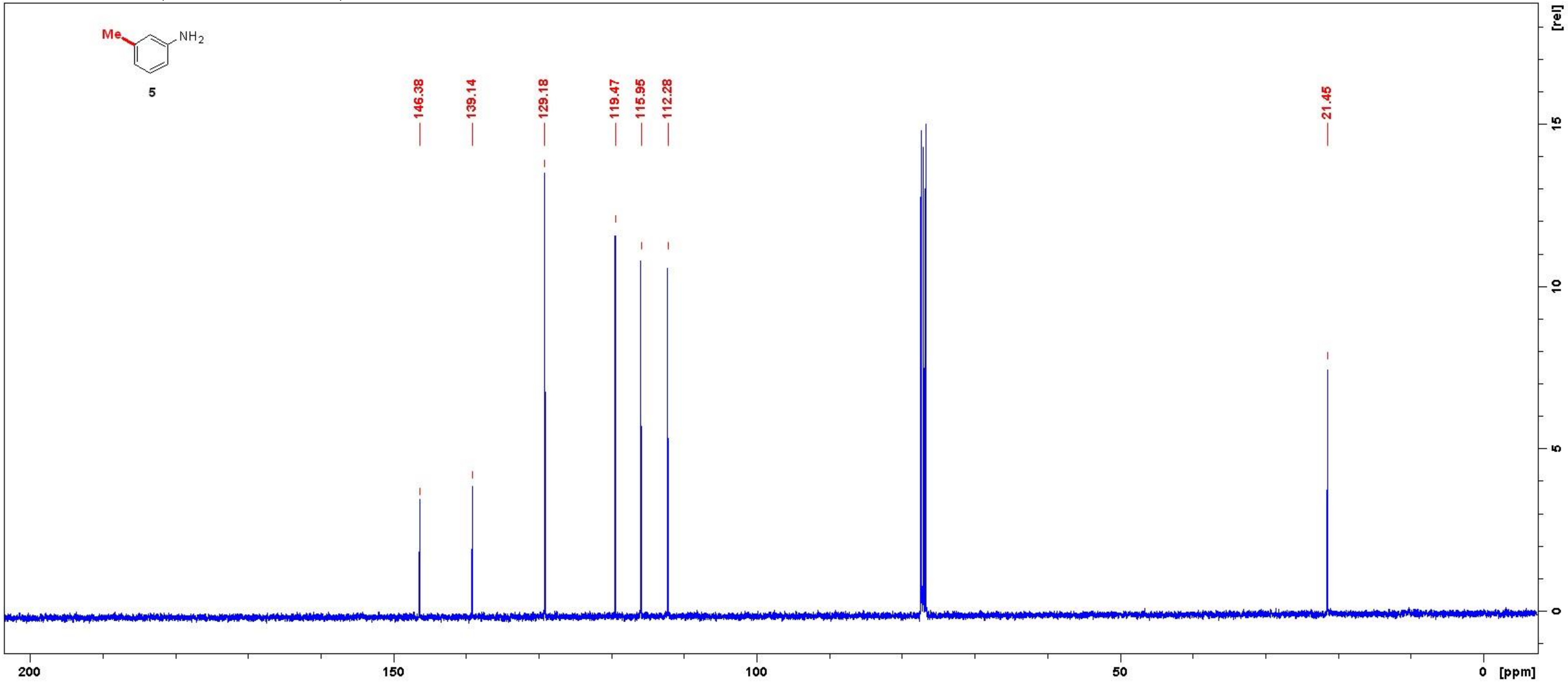

3-butylaniline (6)

^{1}H NMR ($CDCl_3$, 400MHz)

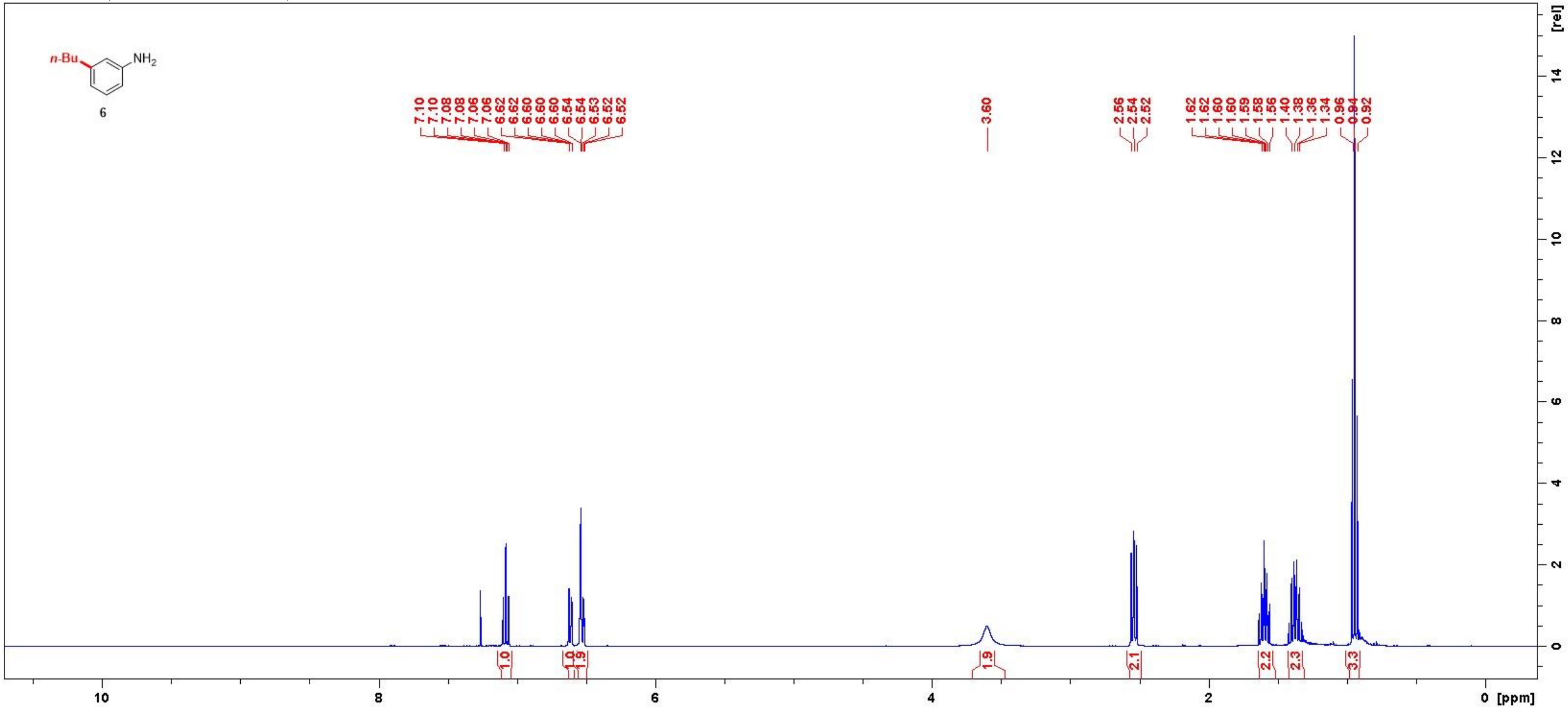

3-butylaniline (6)

$^{13}C\{^1H\}$NMR ($CDCl_3$, 100MHz)

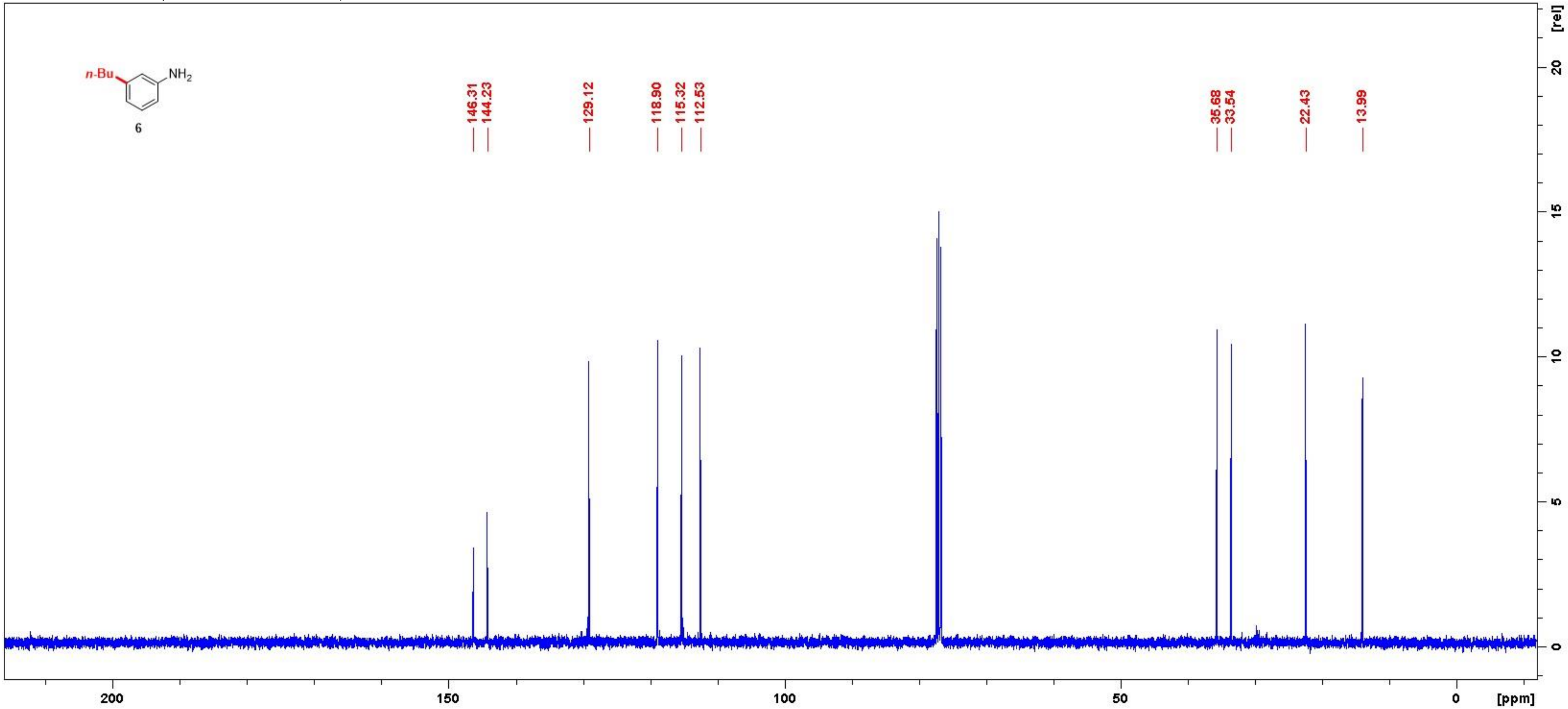

2,4-dimethylaniline (9)

^{1}H NMR ($CDCl_3$, 400MHz)

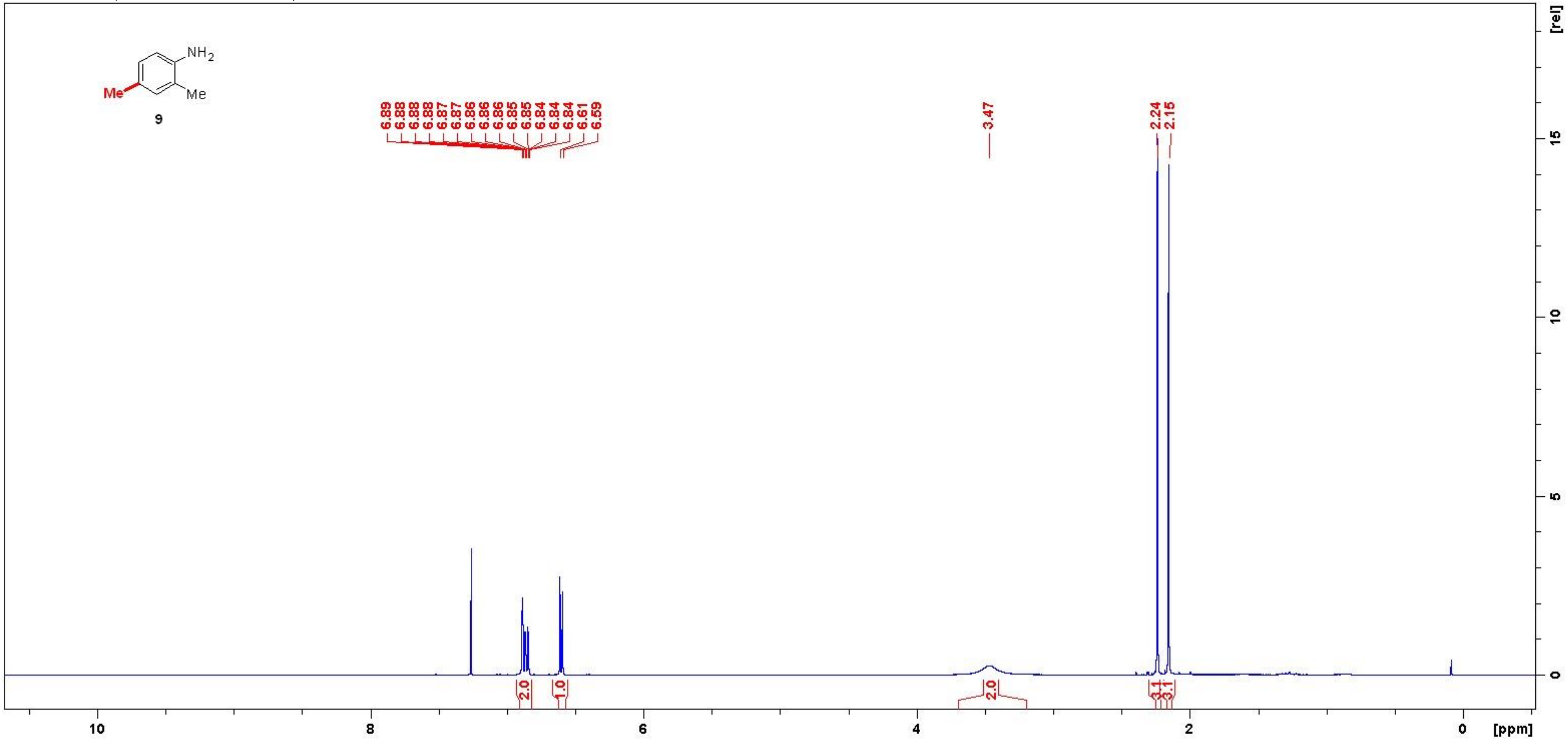

2,4-dimethylaniline (9)

$^{13}C\{^{1}H\}$NMR ($CDCl_3$, 100MHz)

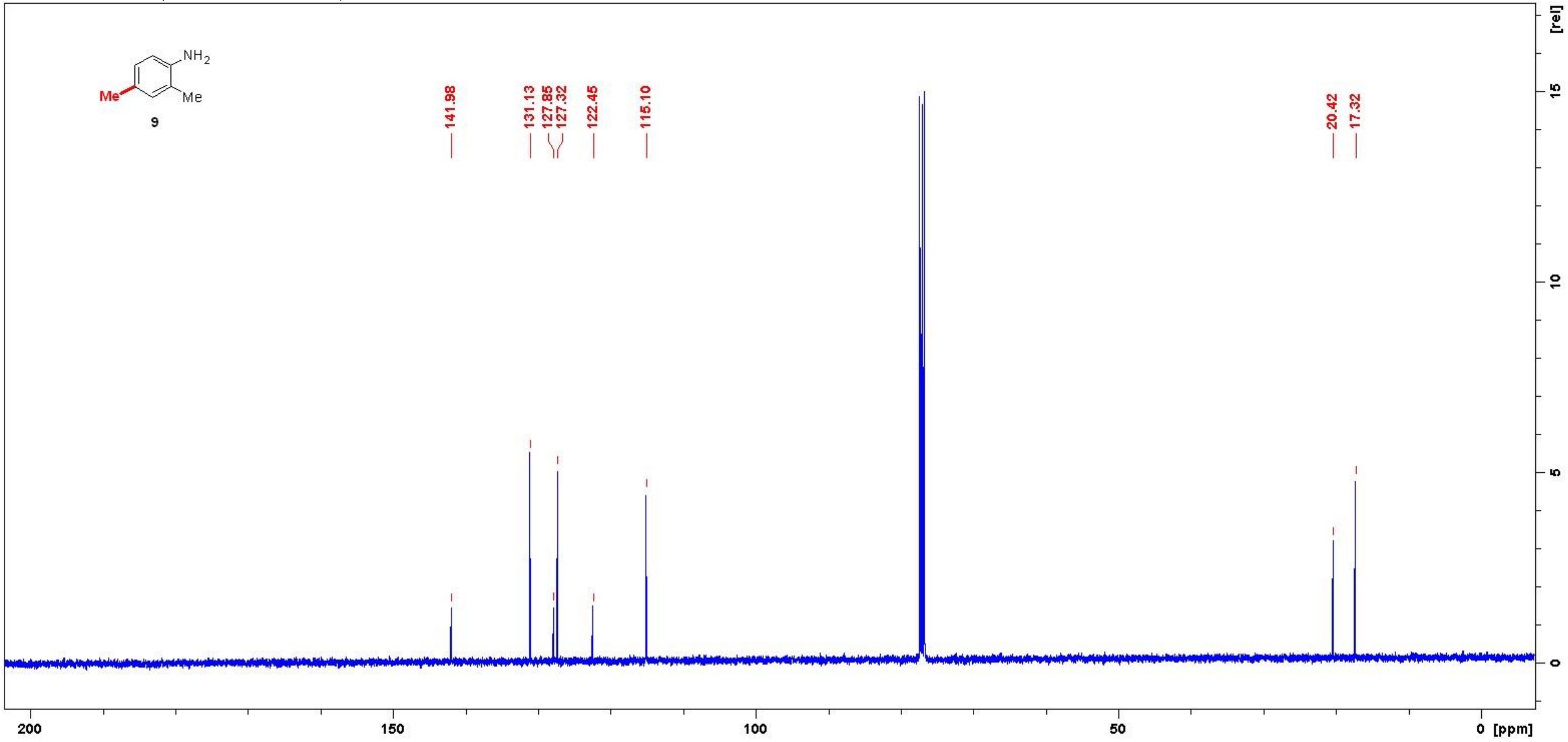

4-butyl-2-methylaniline (10)

^{1}H NMR ($CDCl_3$, 400MHz)

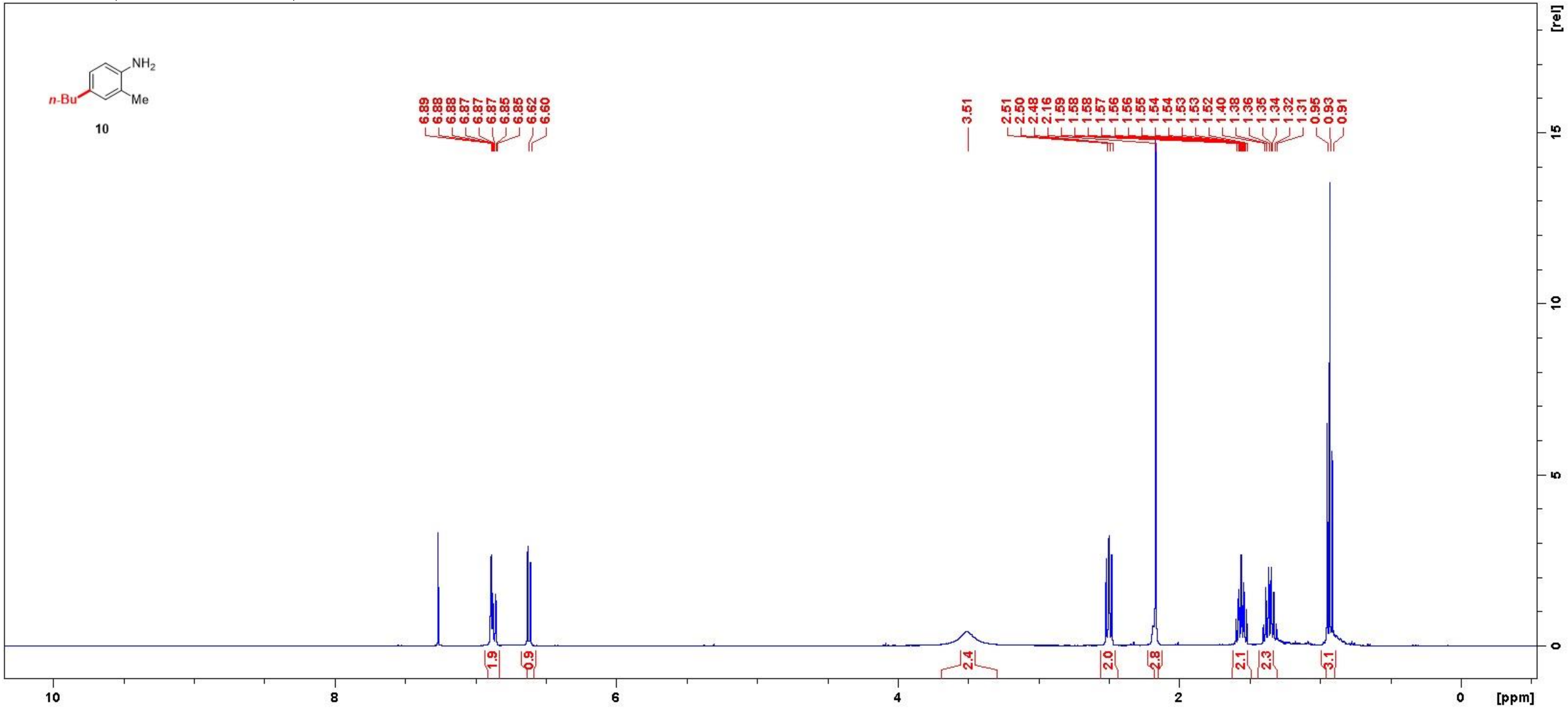

4-butyl-2-methylaniline (10)

$^{13}C\{^{1}H\}$NMR ($CDCl_3$, 100MHz)

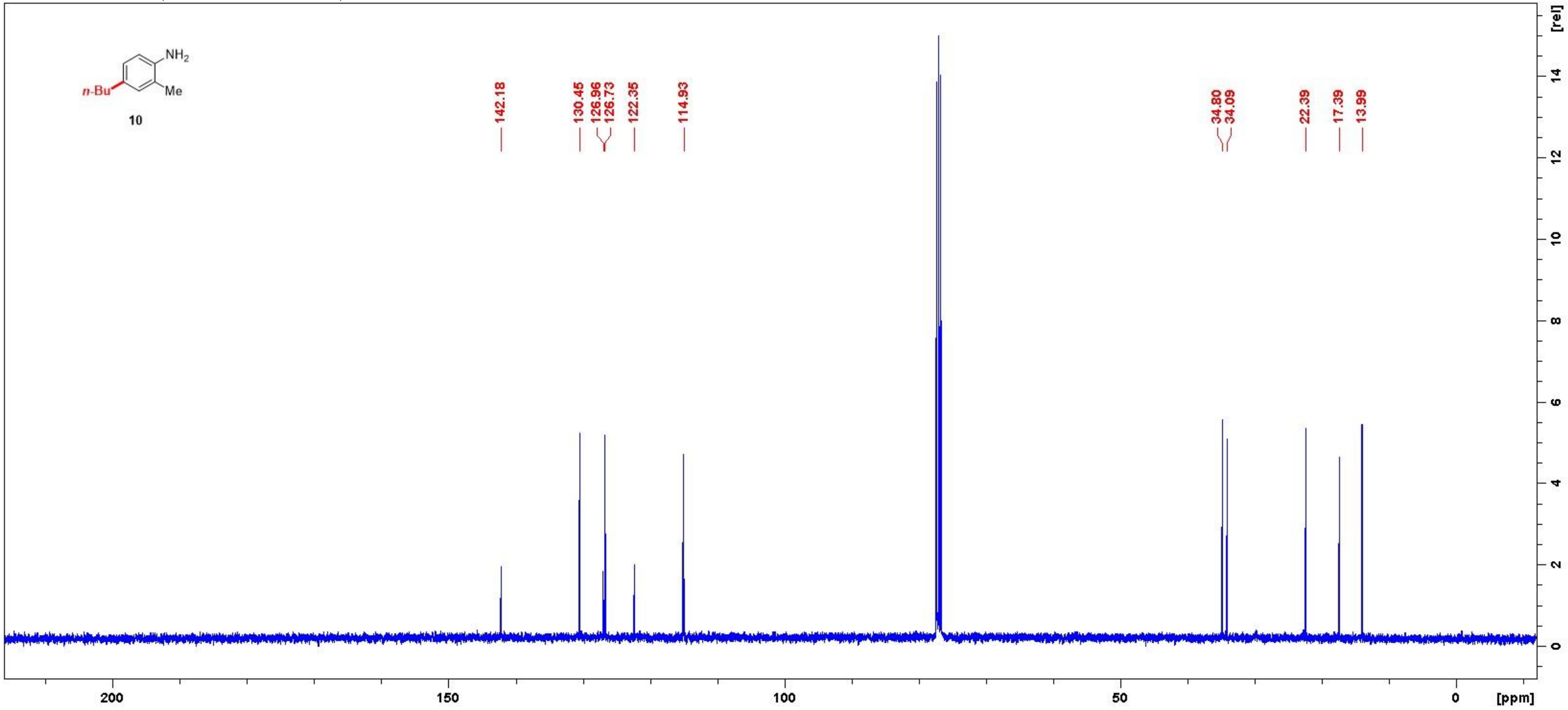

2,4,6-trimethylaniline (11)
^{1}H NMR ($CDCl_3$, 400MHz)

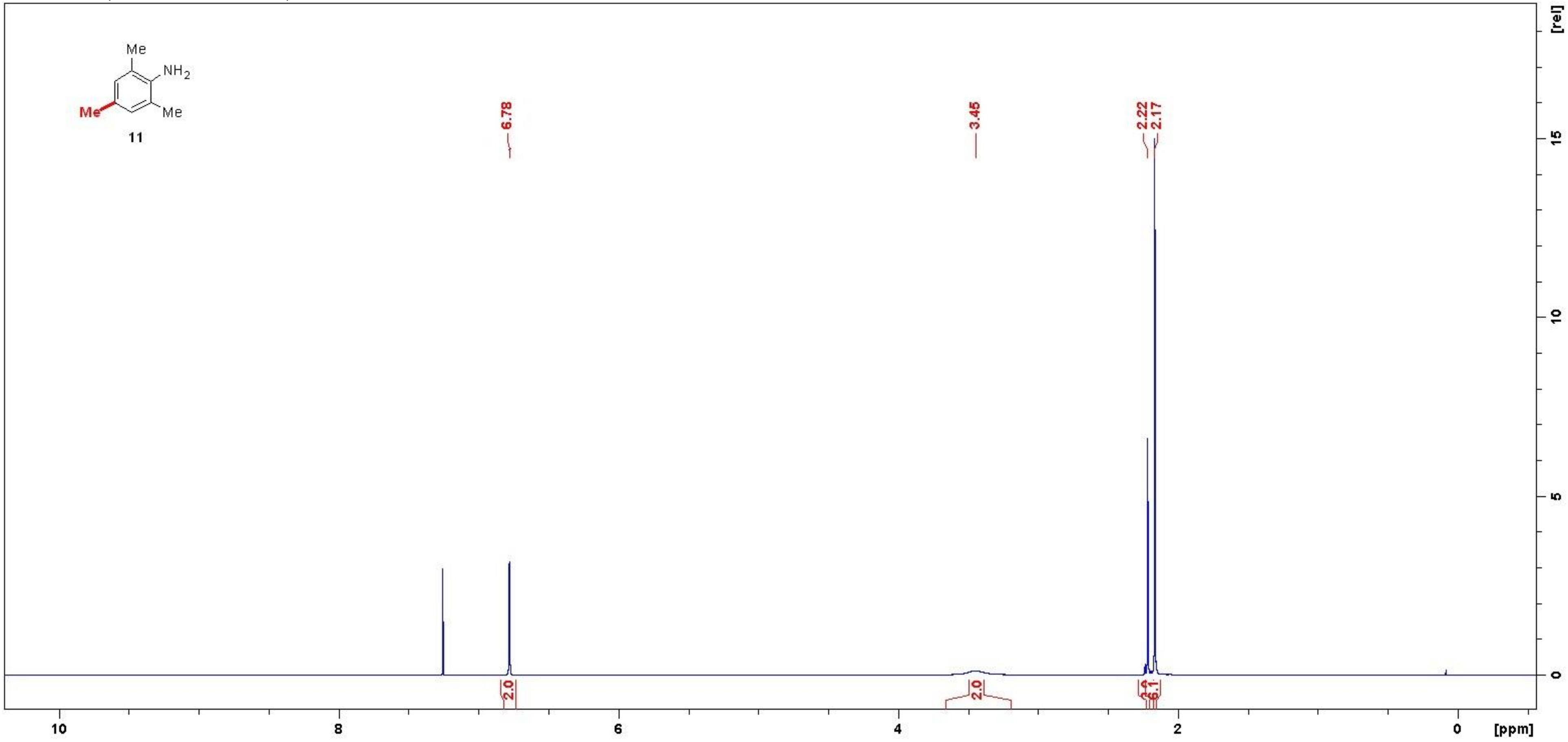

2,4,6-trimethylaniline (11)

$^{13}C\{^{1}H\}$NMR ($CDCl_3$, 100MHz)

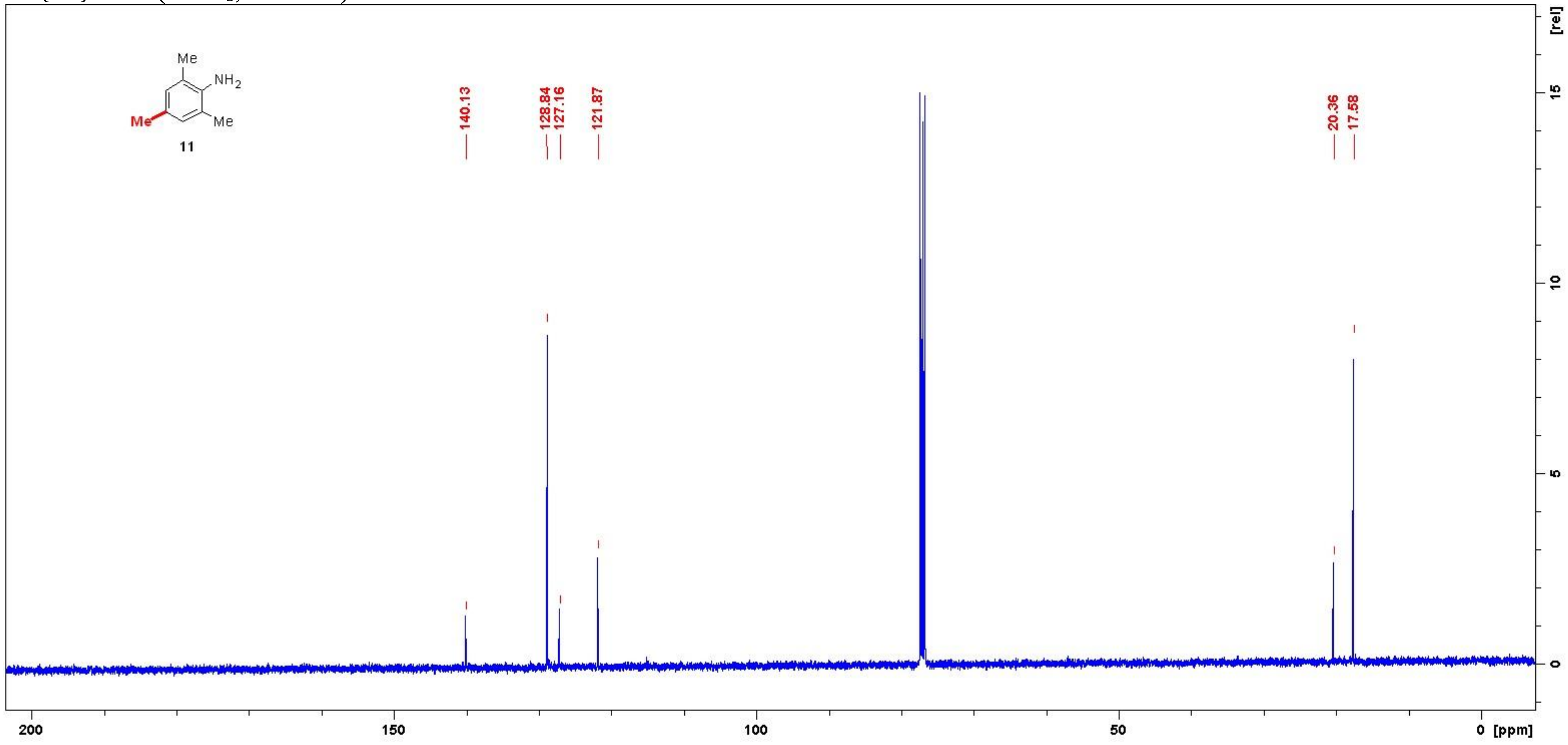

4-butyl-2,6-dimethylaniline (12)

^{1}H NMR ($CDCl_3$, 400MHz)

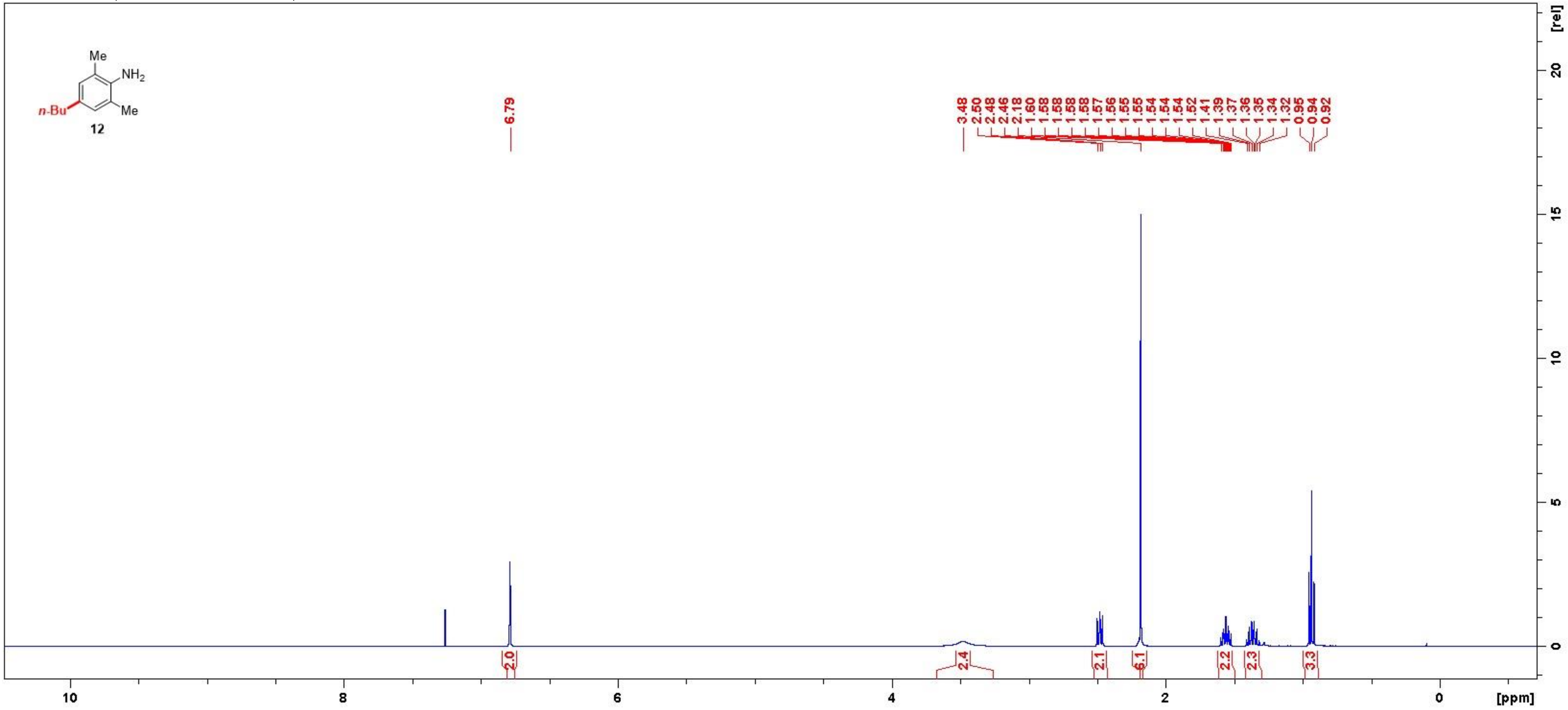

4-butyl-2,6-dimethylaniline (12)

$^{13}C\{^{1}H\}$NMR ($CDCl_3$, 100MHz)

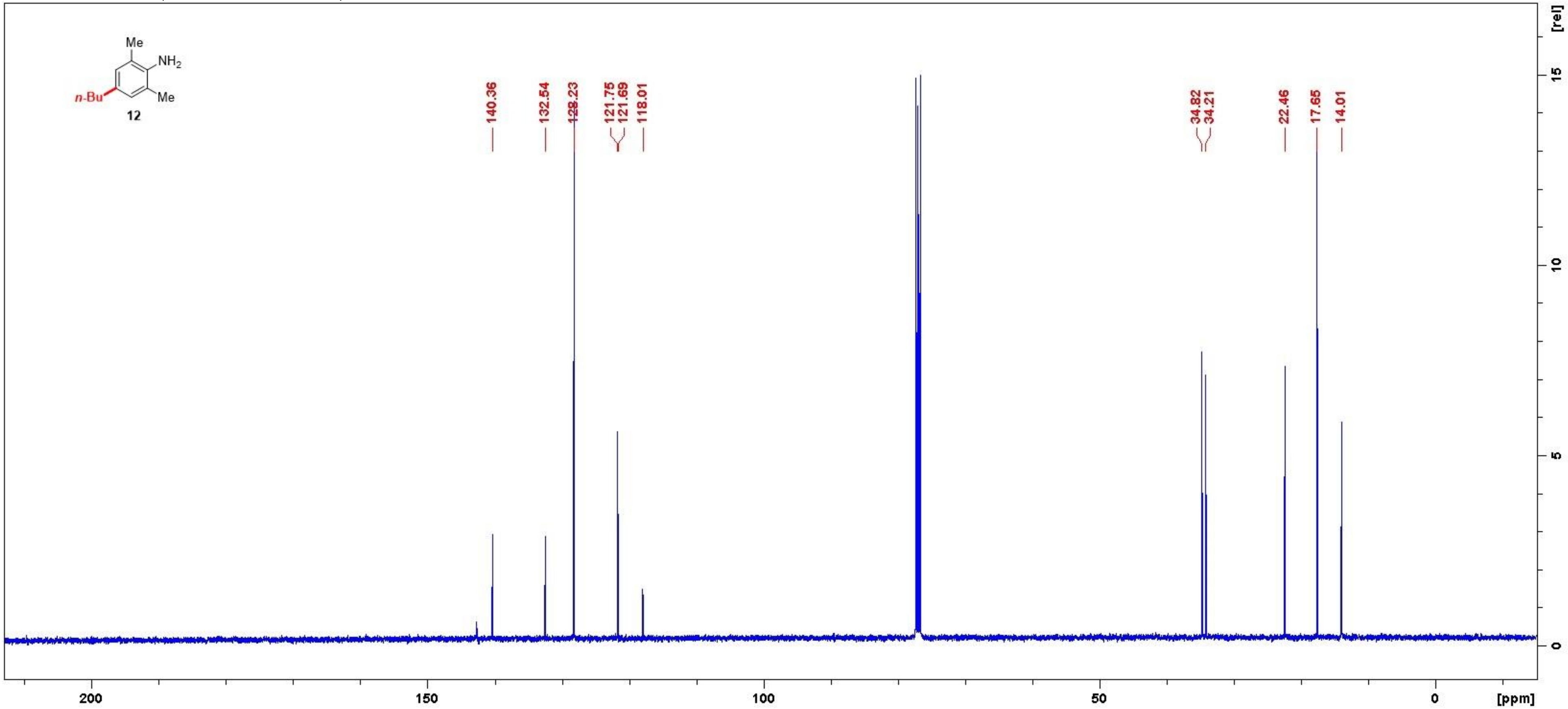

2-fluoro-4-methylaniline (13)

^{1}H NMR ($CDCl_3$, 400MHz)

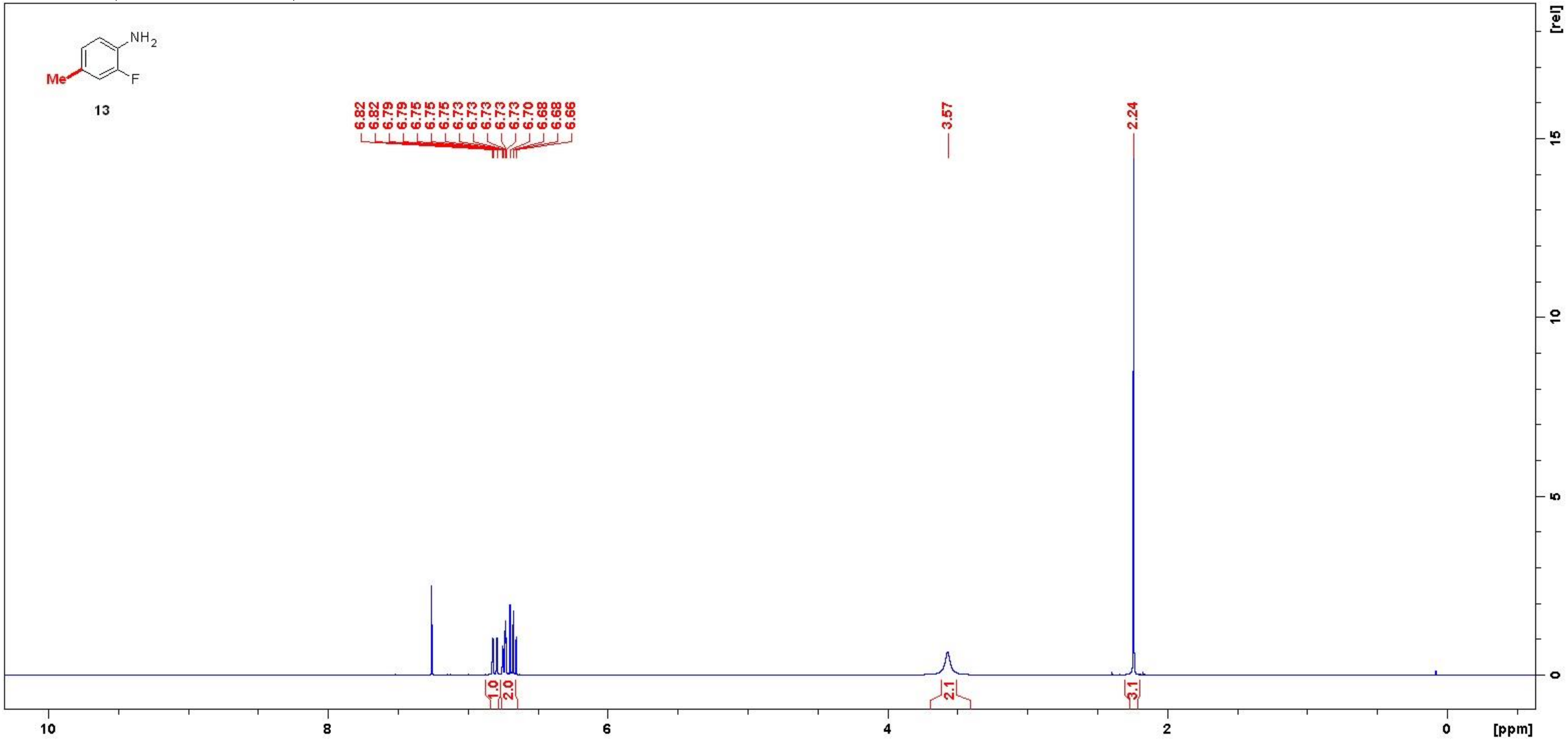

2-fluoro-4-methylaniline (13)

$^{13}C\{^{1}H\}$NMR ($CDCl_3$, 100MHz)

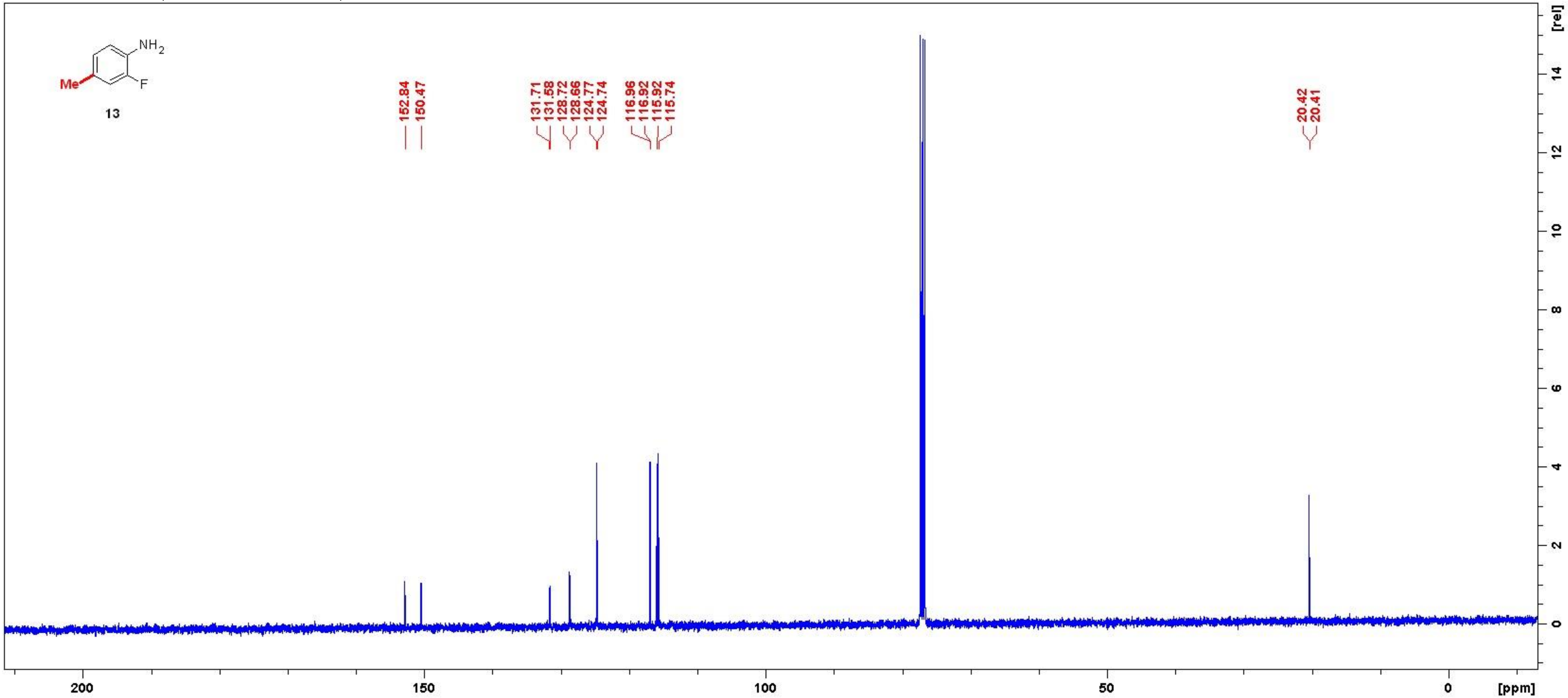

4-butyl-2-fluoroaniline (14)

^{1}H NMR ($CDCl_3$, 400MHz)

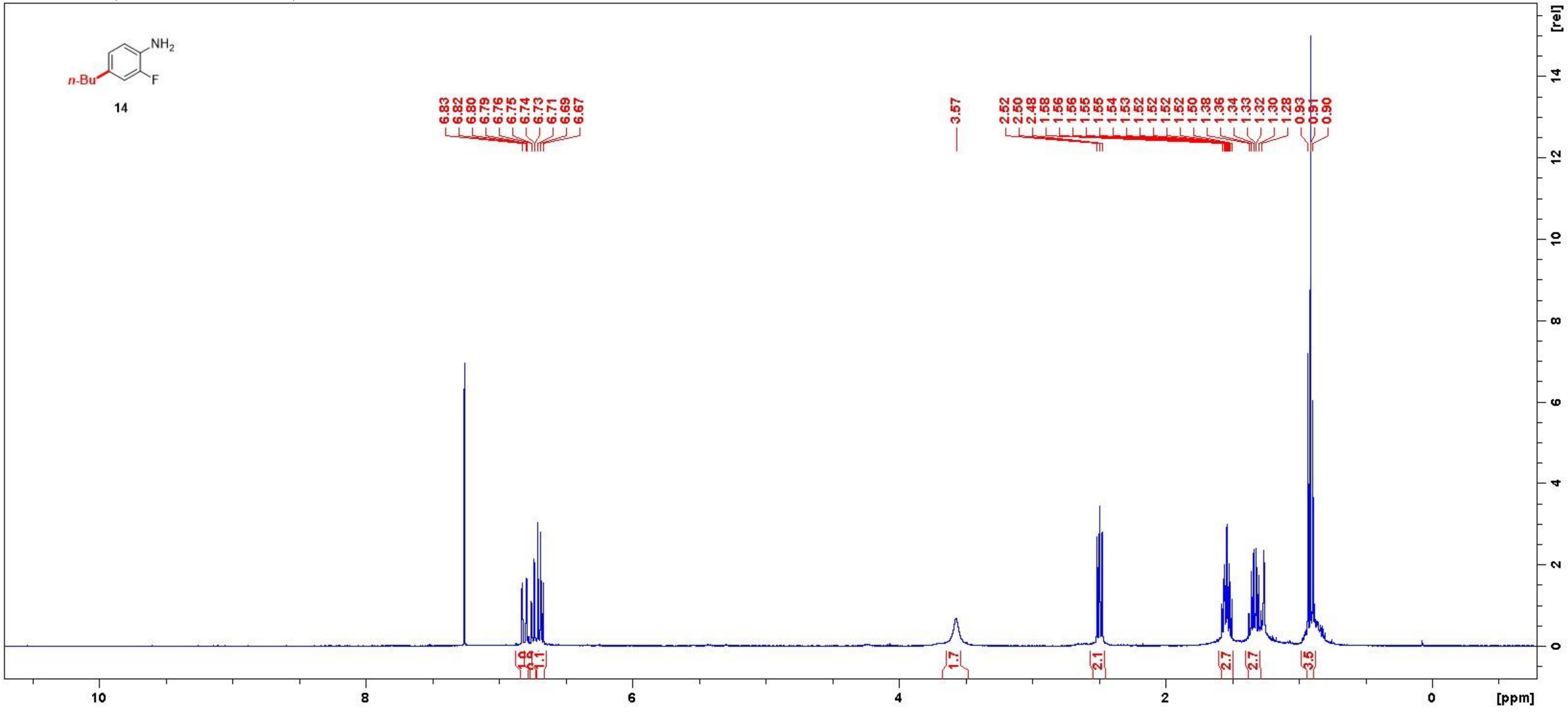

4-butyl-2-fluoroaniline (14)

$^{13}C\{^{1}H\}$NMR ($CDCl_3$, 100MHz)

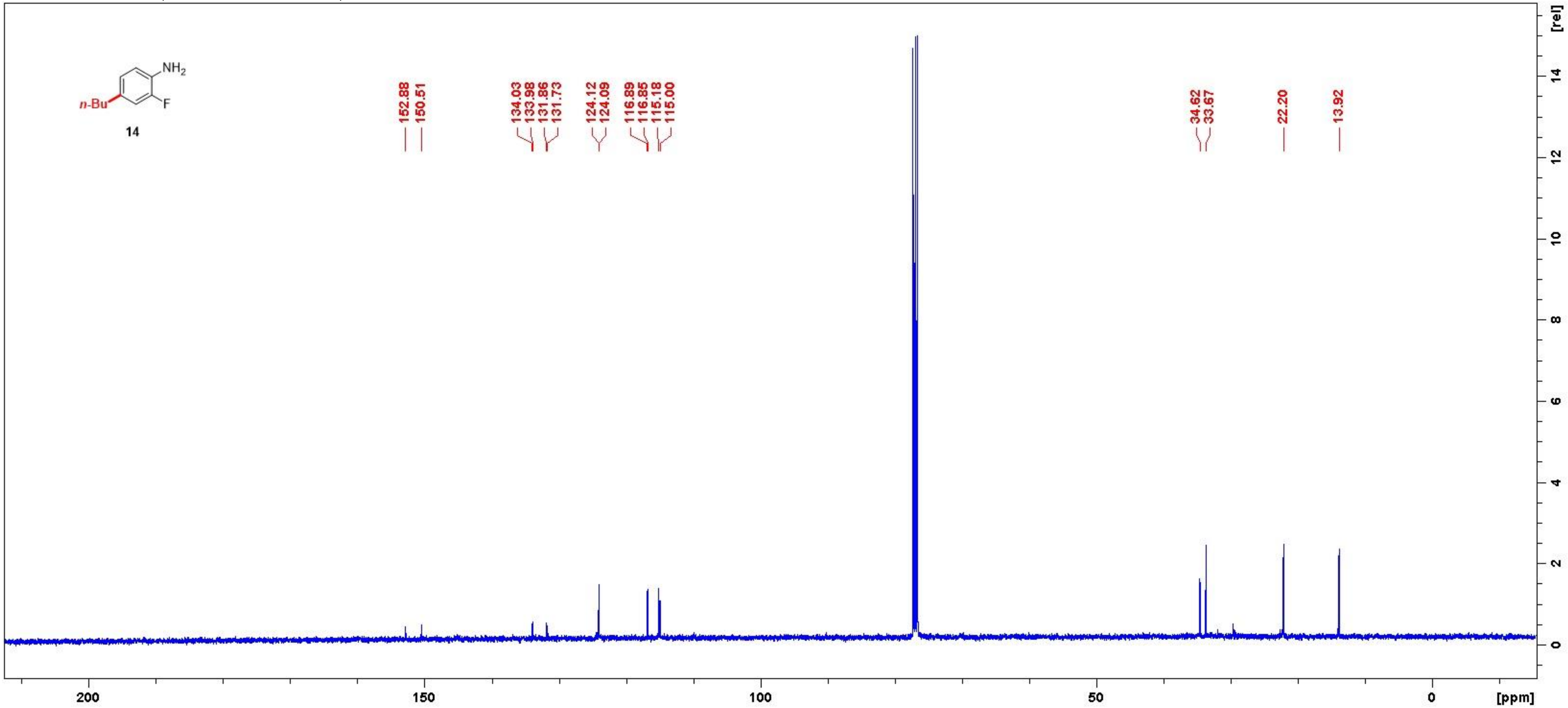

2,5-difluoro-4-methylaniline (15)

^{1}H NMR ($CDCl_3$, 400MHz)

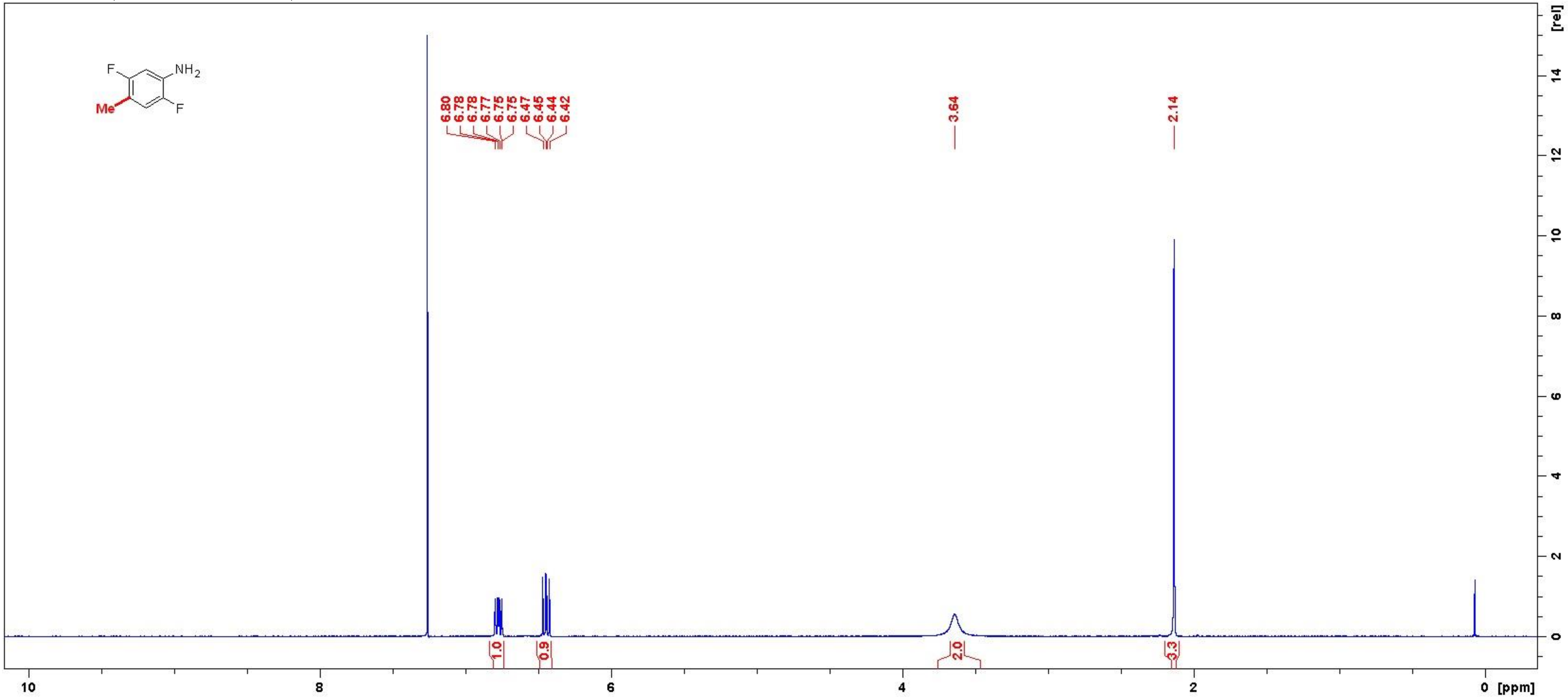

2,5-difluoro-4-methylaniline (15)

$^{13}C\{^1H\}$NMR ($CDCl_3$, 100MHz)

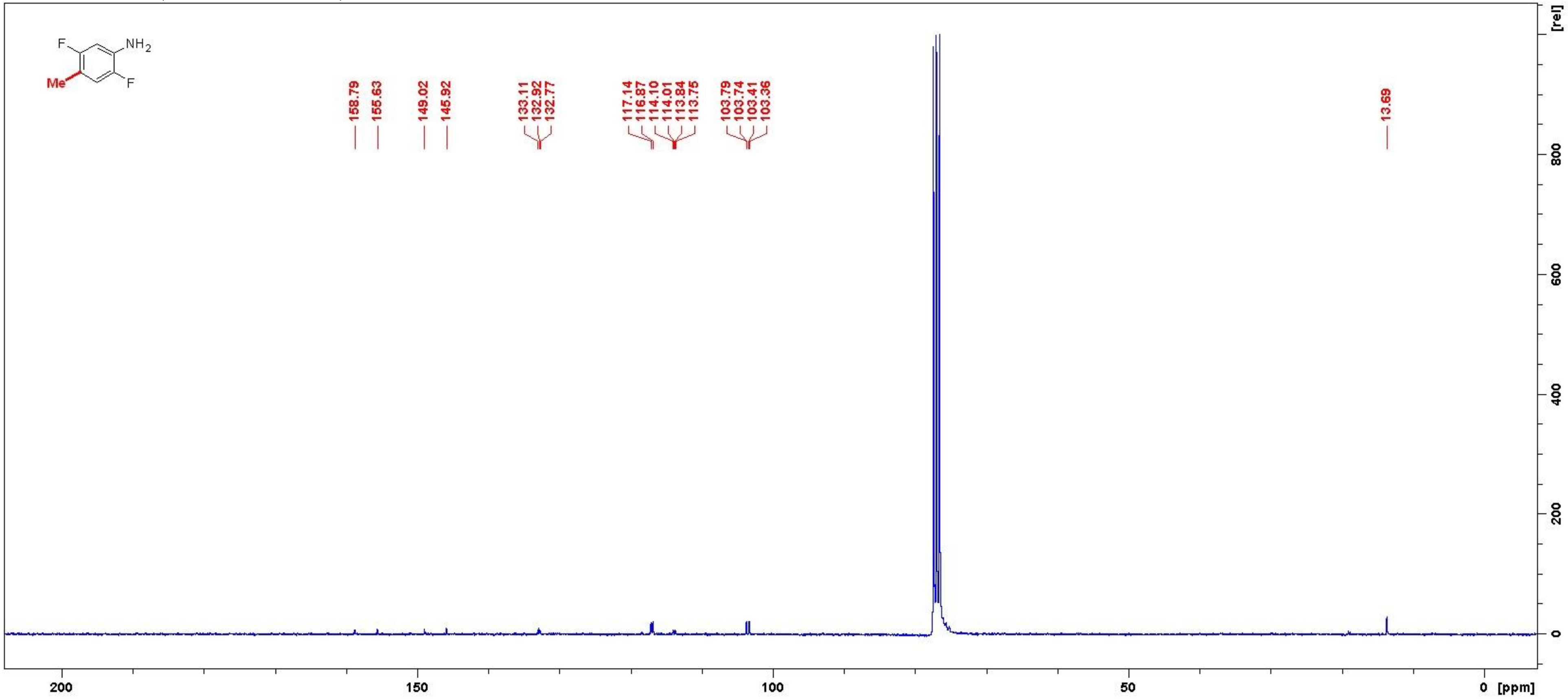

4-butyl-2,5-difluoroaniline (16)

^{1}H NMR ($CDCl_3$, 400MHz)

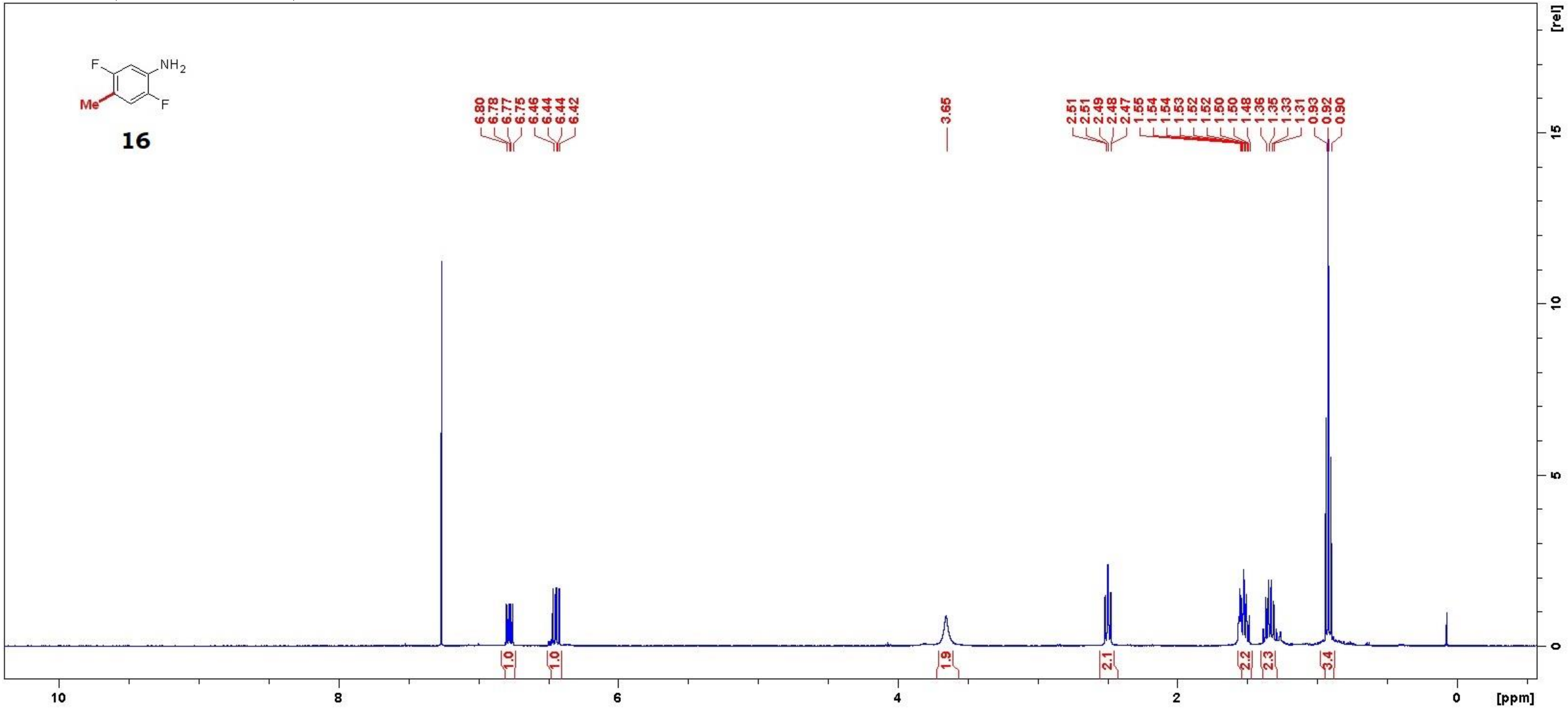

4-butyl-2,5-difluoroaniline (16)

$^{13}C\{^{1}H\}$NMR ($CDCl_3$, 100MHz)

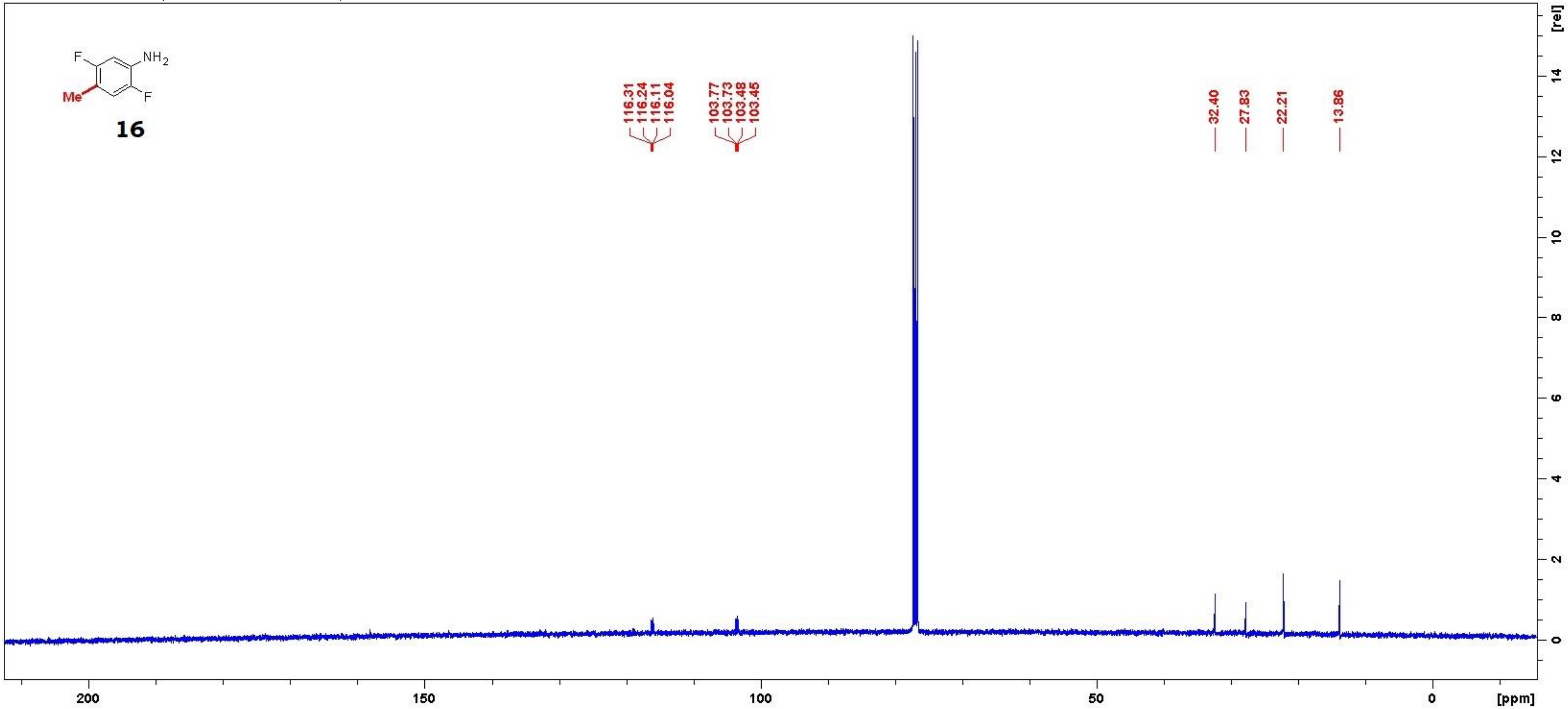

2-chloro-4-methylaniline (17)

^{1}H NMR ($CDCl_3$, 400MHz)

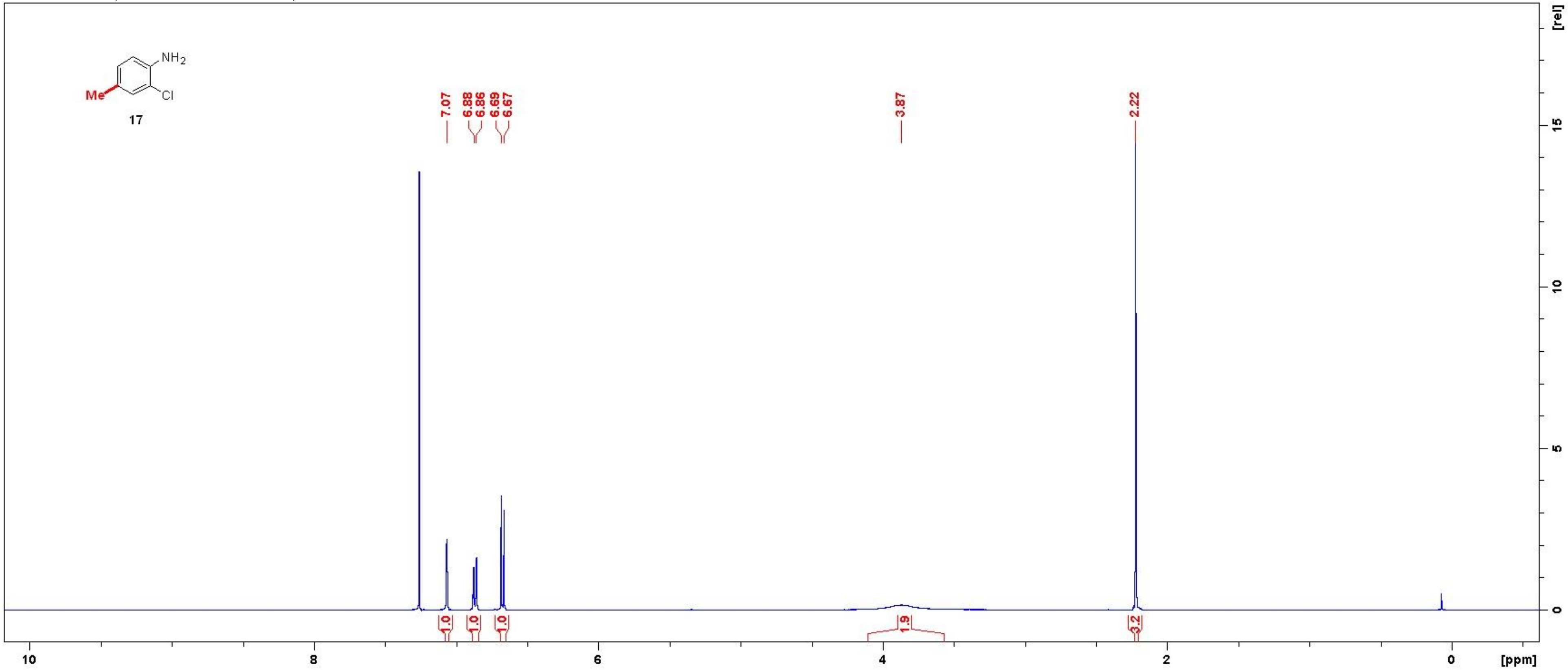

2-chloro-4-methylaniline (17)

$^{13}C\{^{1}H\}$NMR ($CDCl_3$, 100MHz)

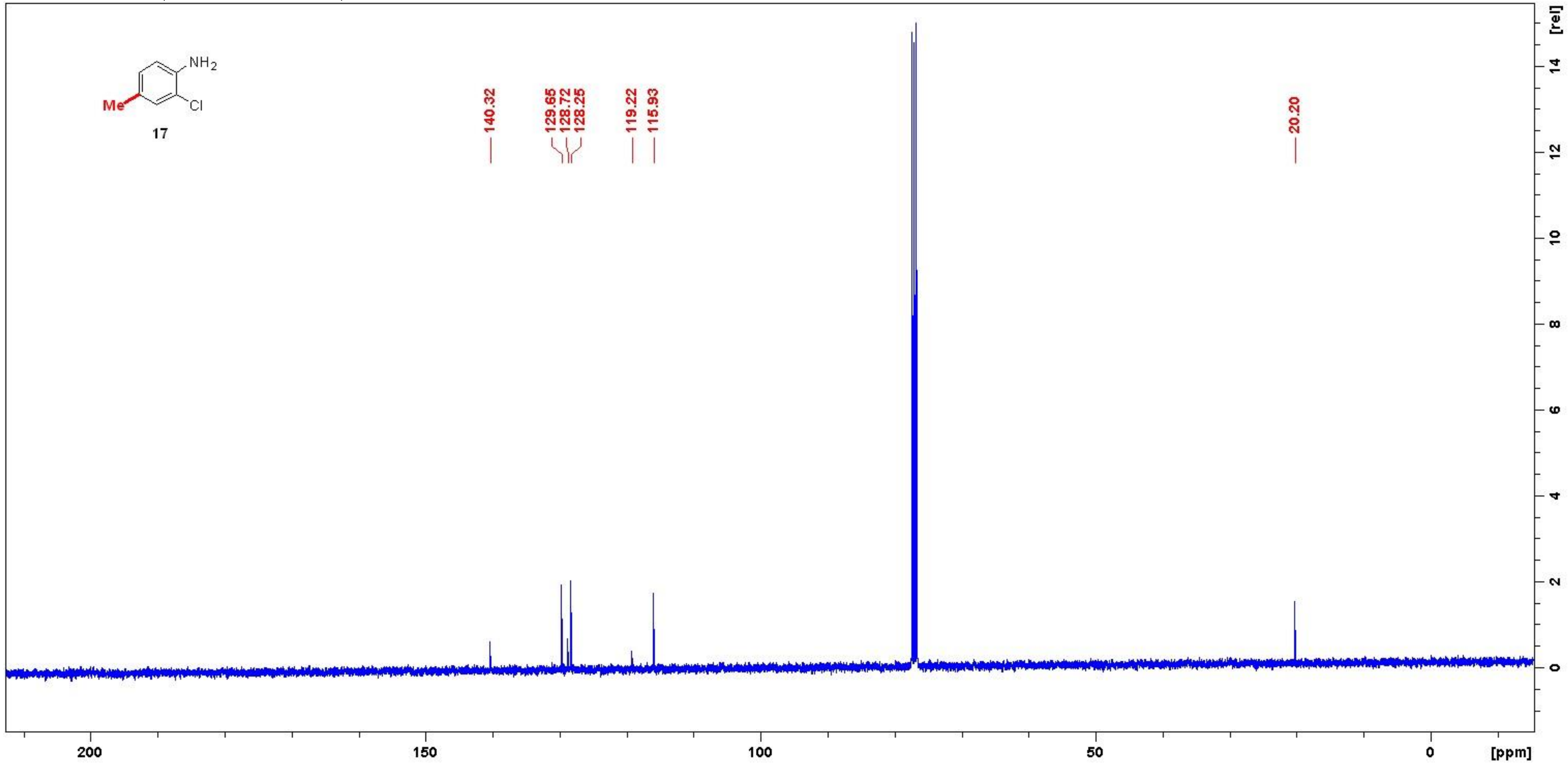

4-butyl-2-chloroaniline (18)

^{1}H NMR ($CDCl_3$, 400MHz)

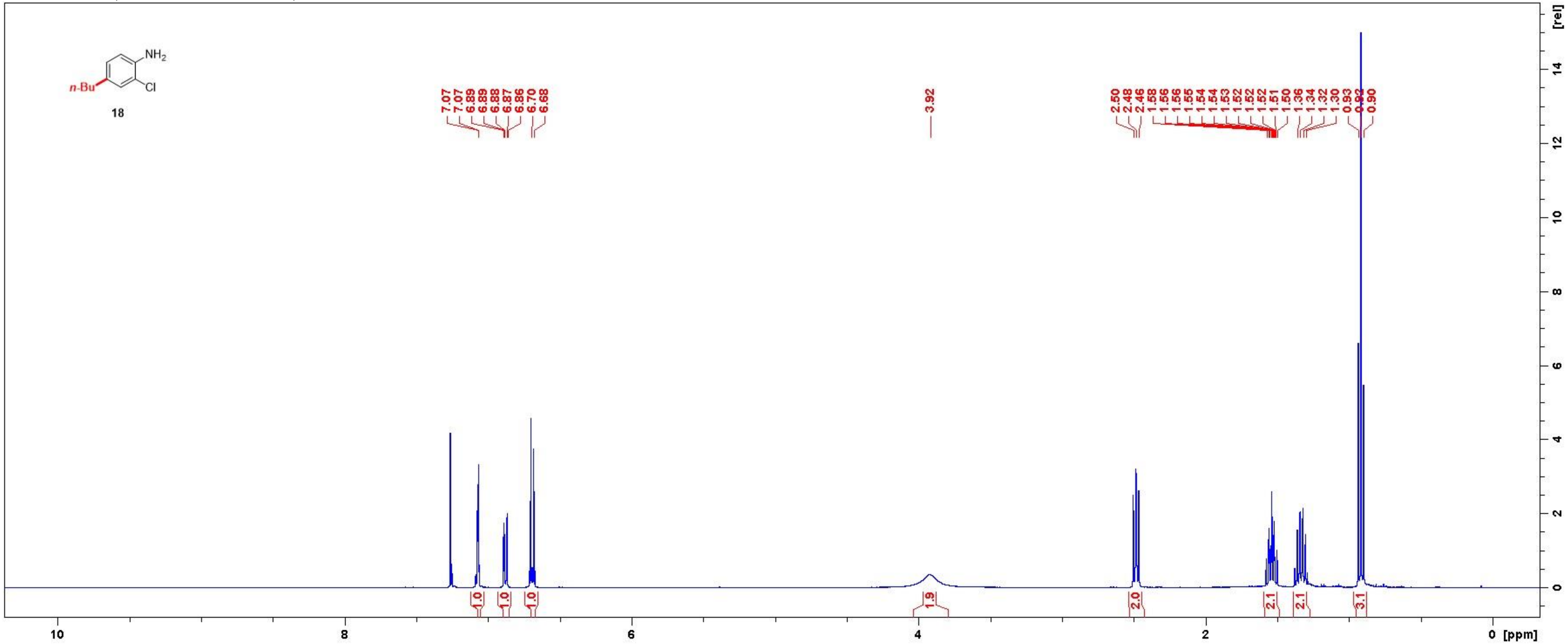

4-butyl-2-chloroaniline (18)

$^{13}C\{^1H\}$NMR ($CDCl_3$, 100MHz)

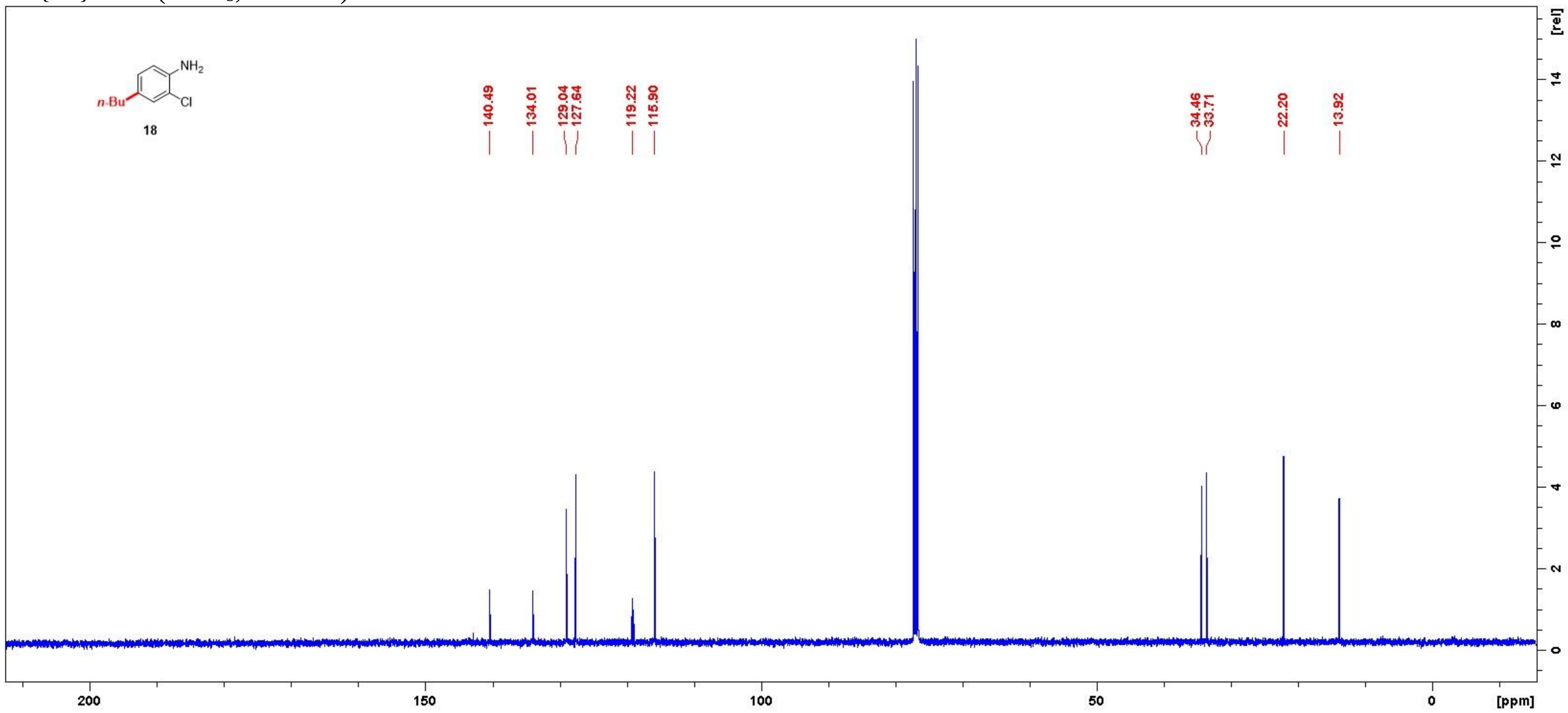

4-methylnaphthalen-1-amine (19)

^{1}H NMR ($CDCl_3$, 400MHz)

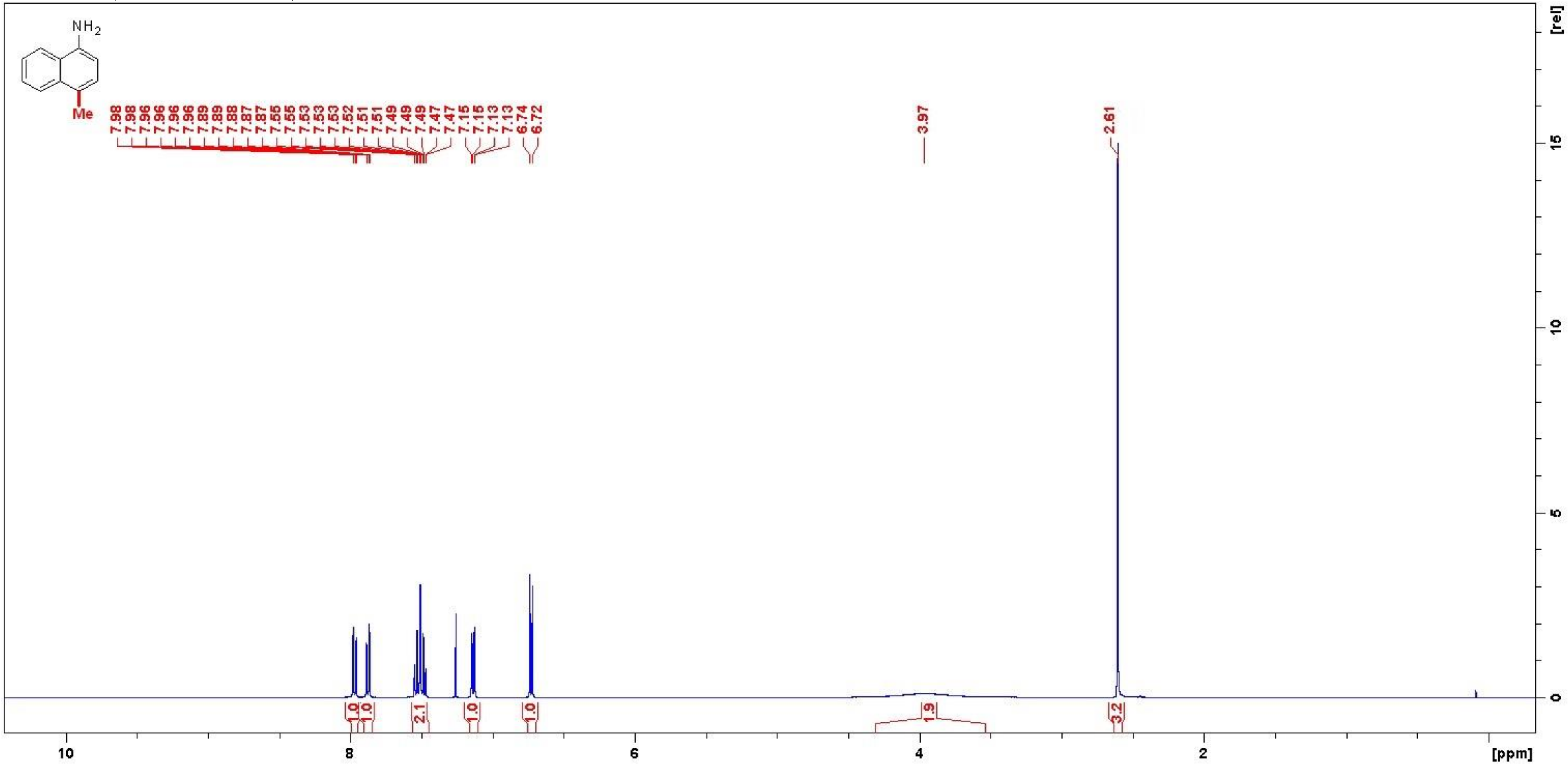

4-methylnaphthalen-1-amine (19)

$^{13}C\{^{1}H\}$NMR ($CDCl_3$, 100MHz)

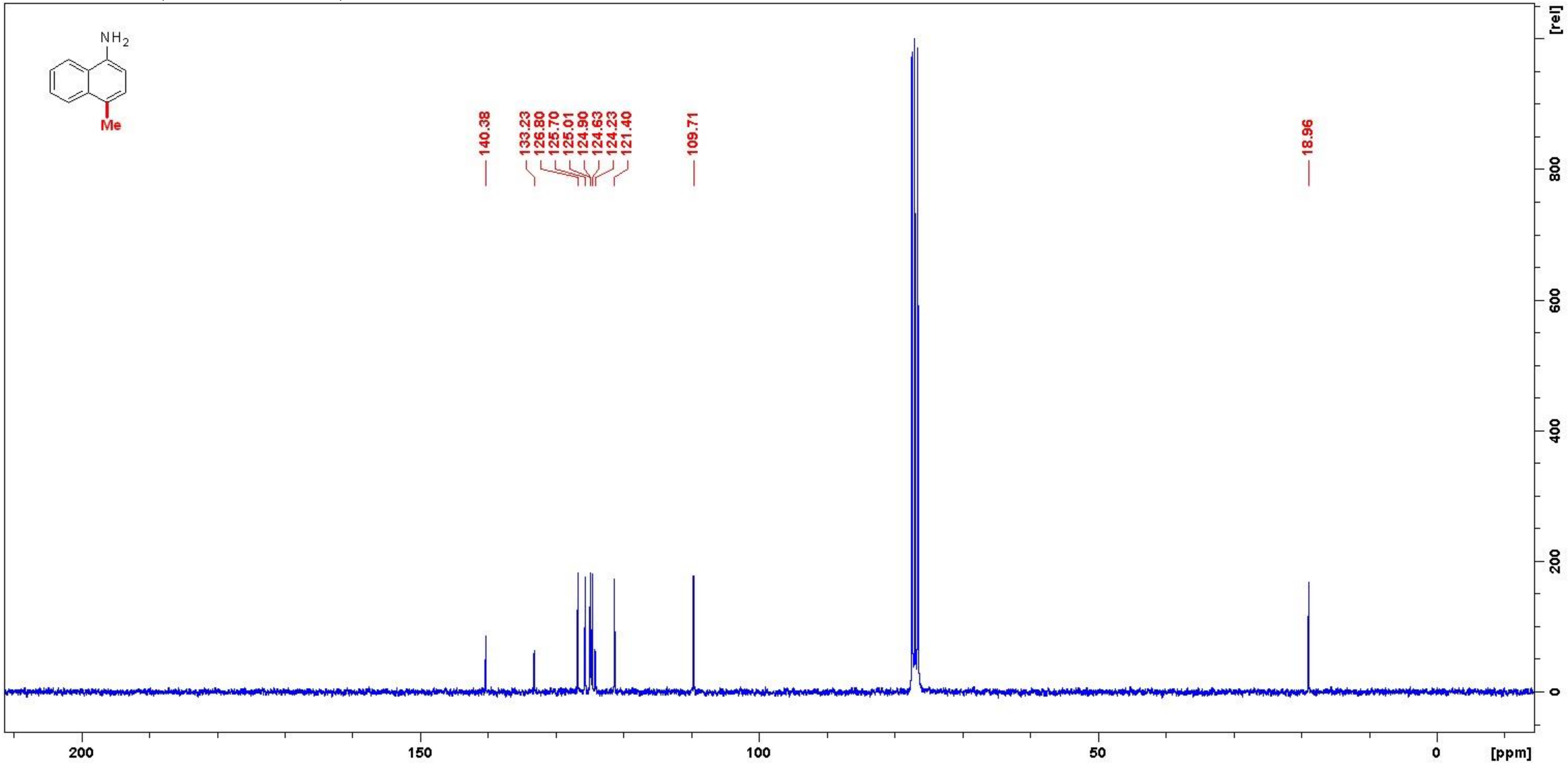

4-butylnaphthalen-1-amine (20)

^{1}H NMR ($CDCl_3$, 400MHz)

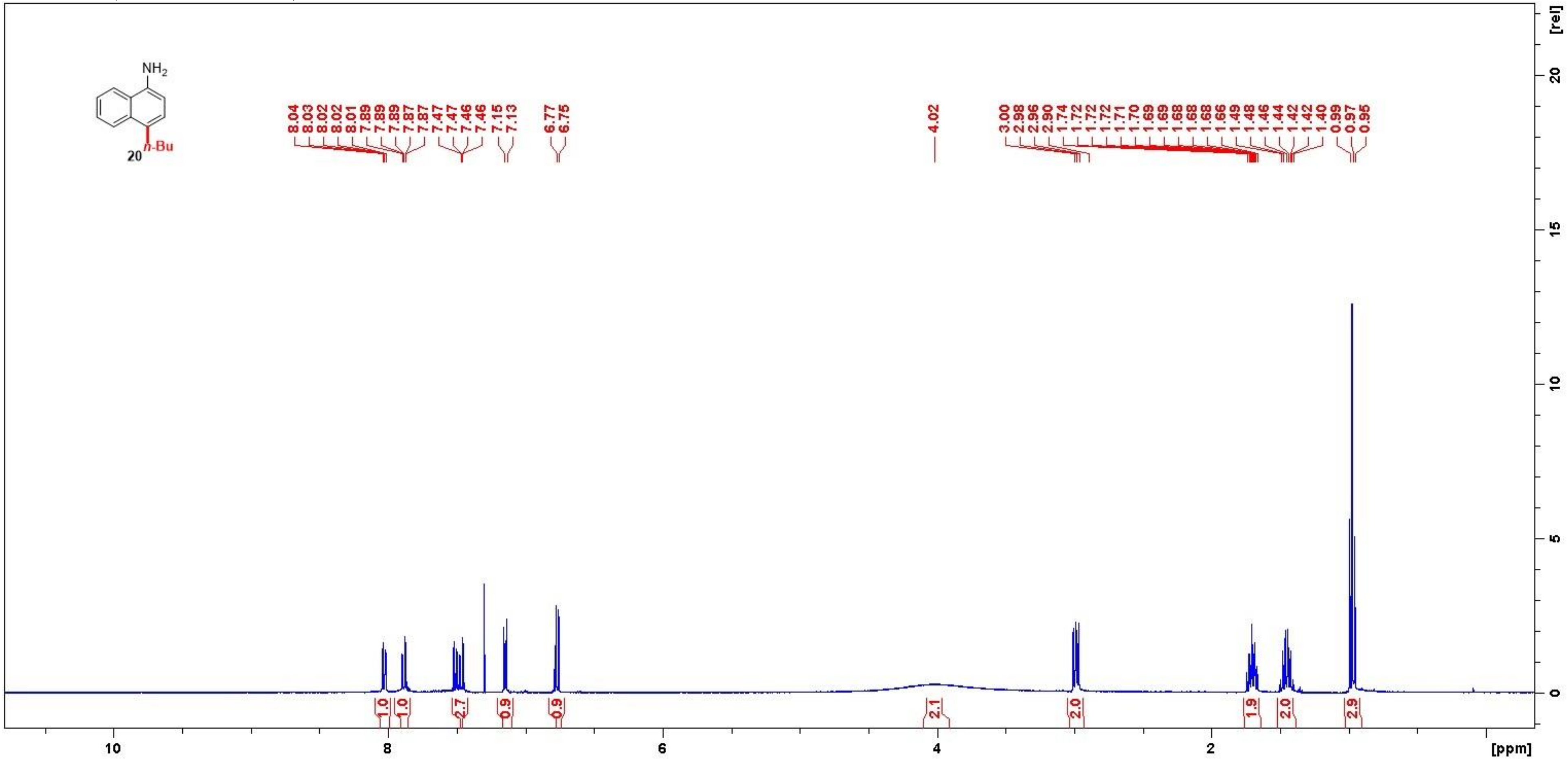

4-butylnaphthalen-1-amine (20)

$^{13}C\{^{1}H\}$NMR ($CDCl_3$, 100MHz)

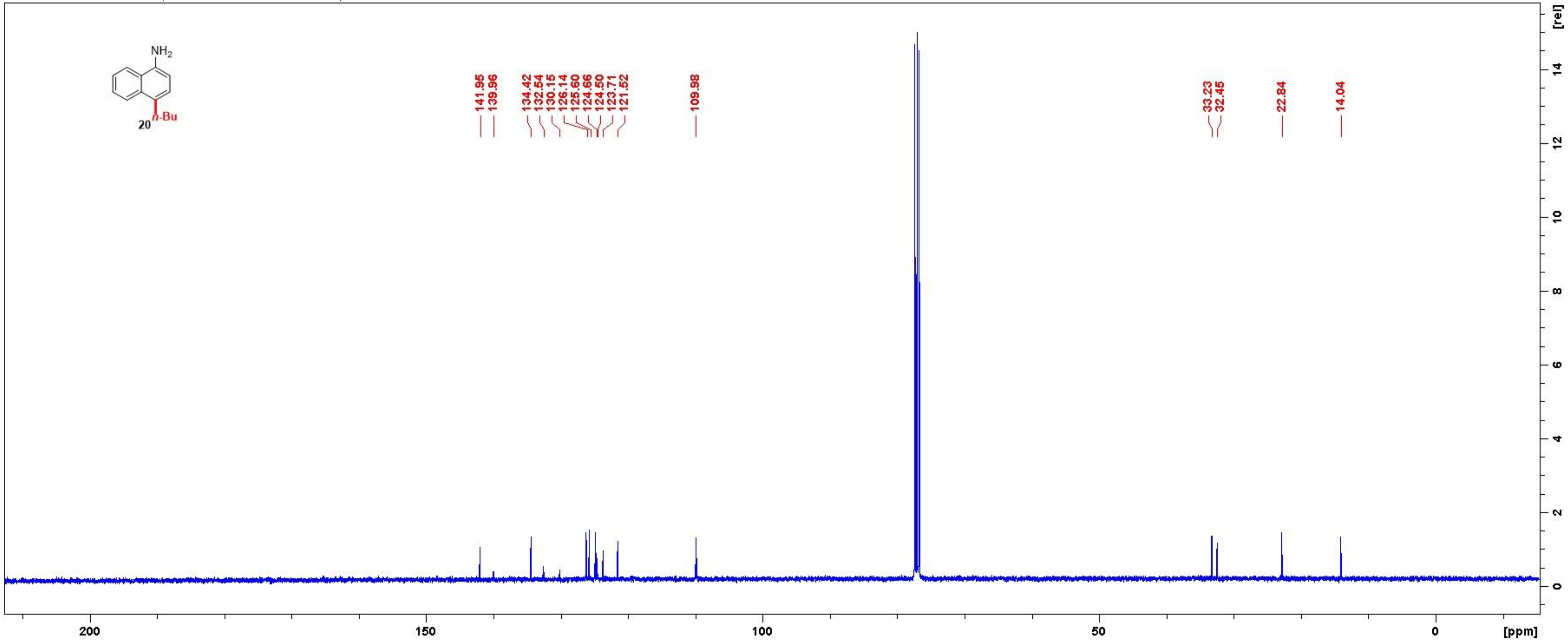

3,4-dimethylaniline (21)

^{1}H NMR ($CDCl_3$, 400MHz)

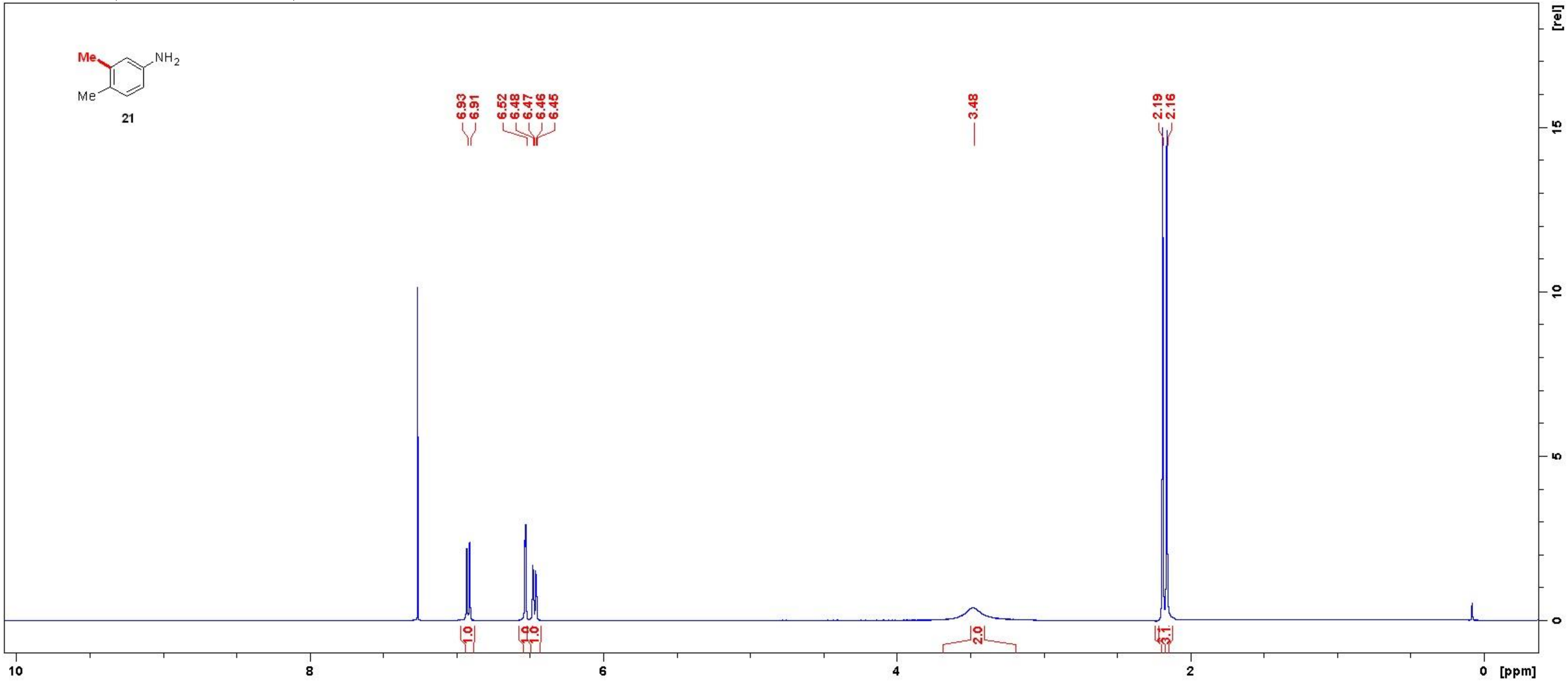

3,4-dimethylaniline (21)

$^{13}C\{^{1}H\}$NMR ($CDCl_3$, 100MHz)

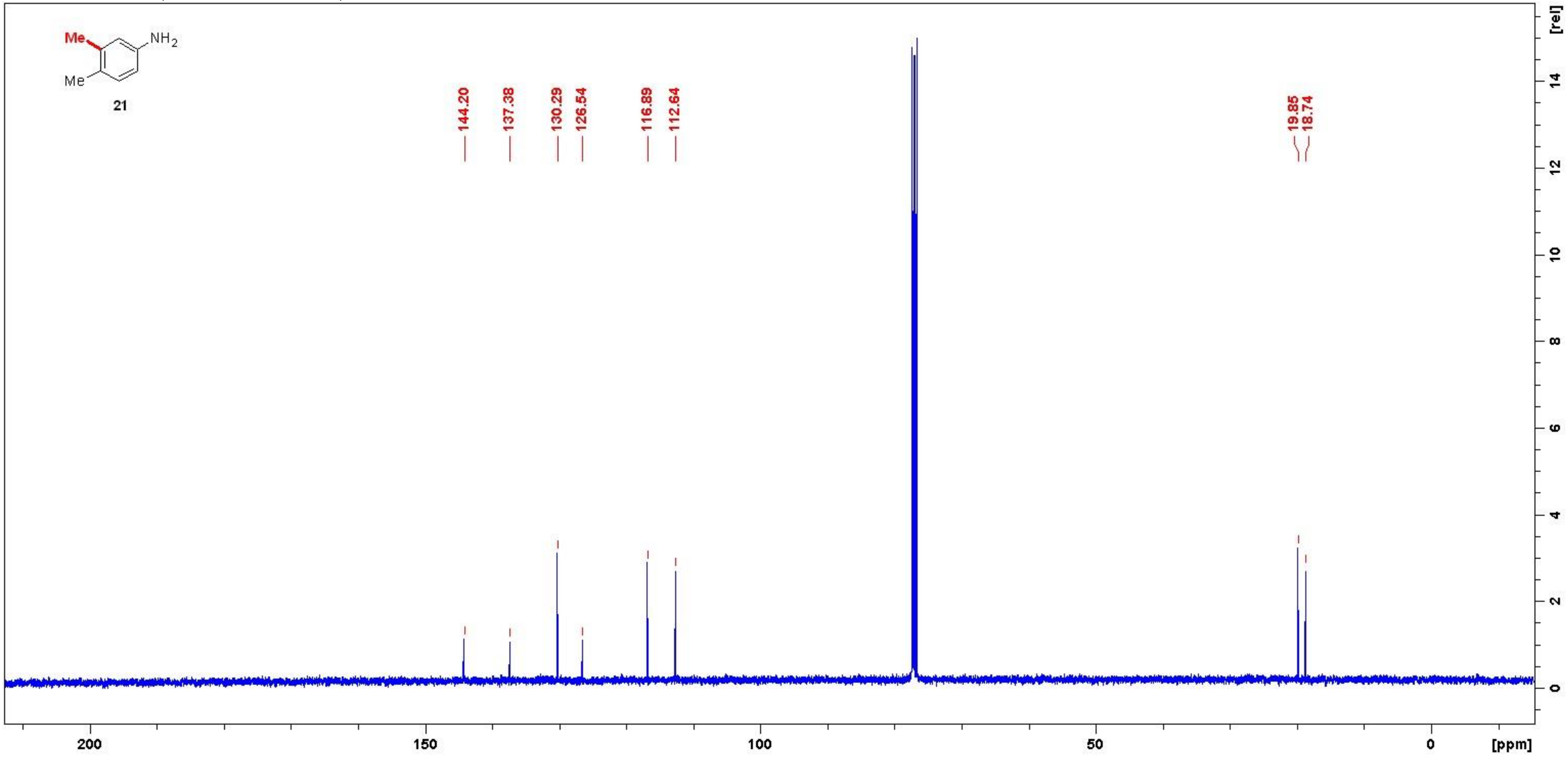

4-methylbenzene-1,2-diamine (23)

^{1}H NMR ($CDCl_3$, 400MHz)

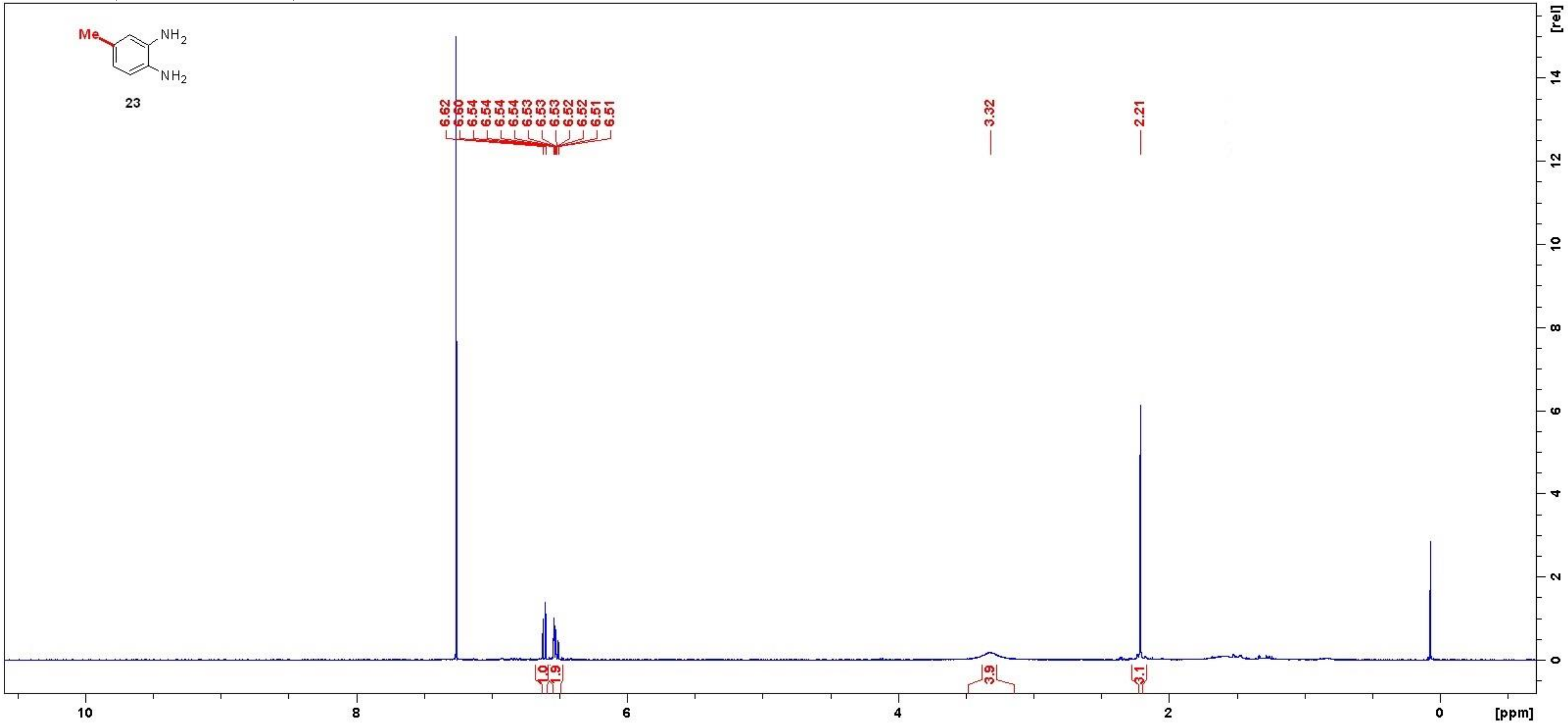

4-methylbenzene-1,2-diamine (23)

$^{13}C\{^{1}H\}$NMR ($CDCl_3$, 100MHz)

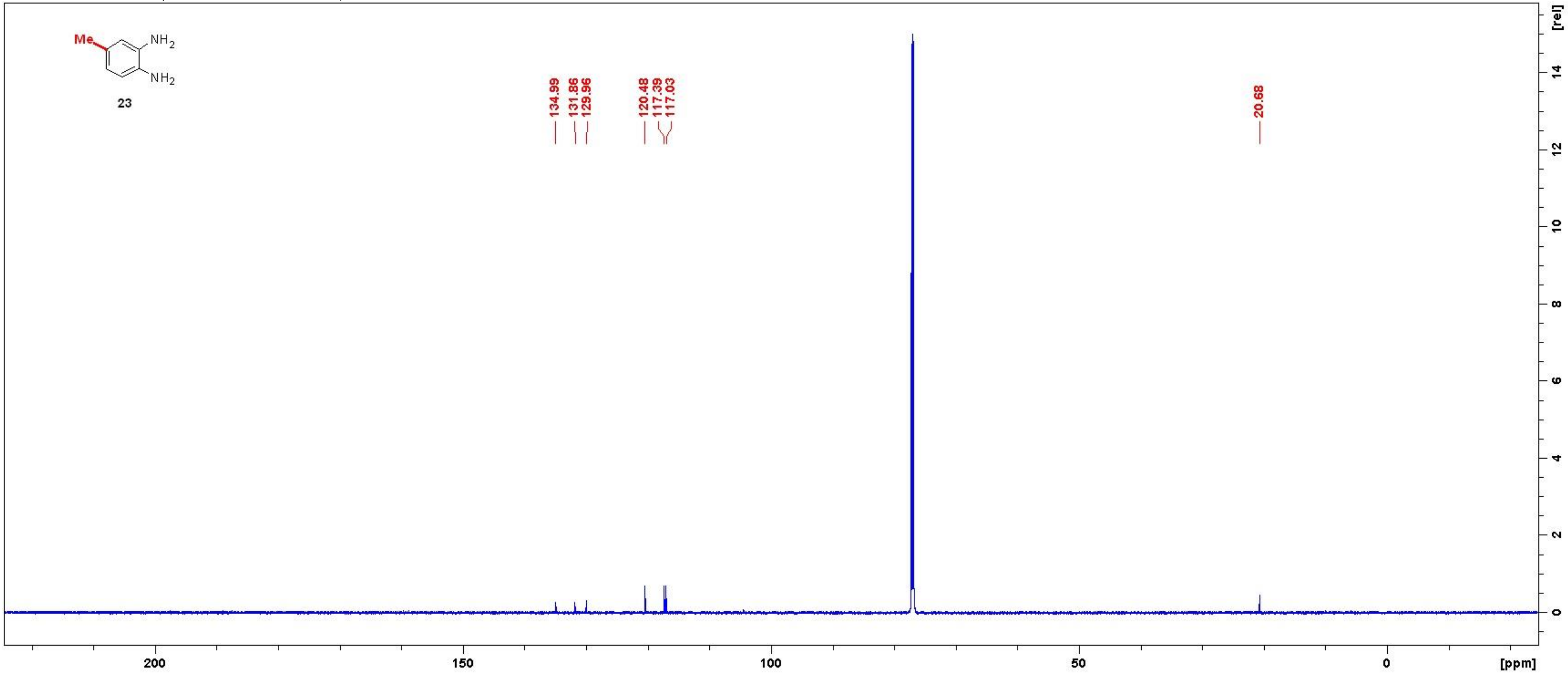

4-butylbenzene-1,2-diamine (24)

^{1}H NMR ($CDCl_3$, 400MHz)

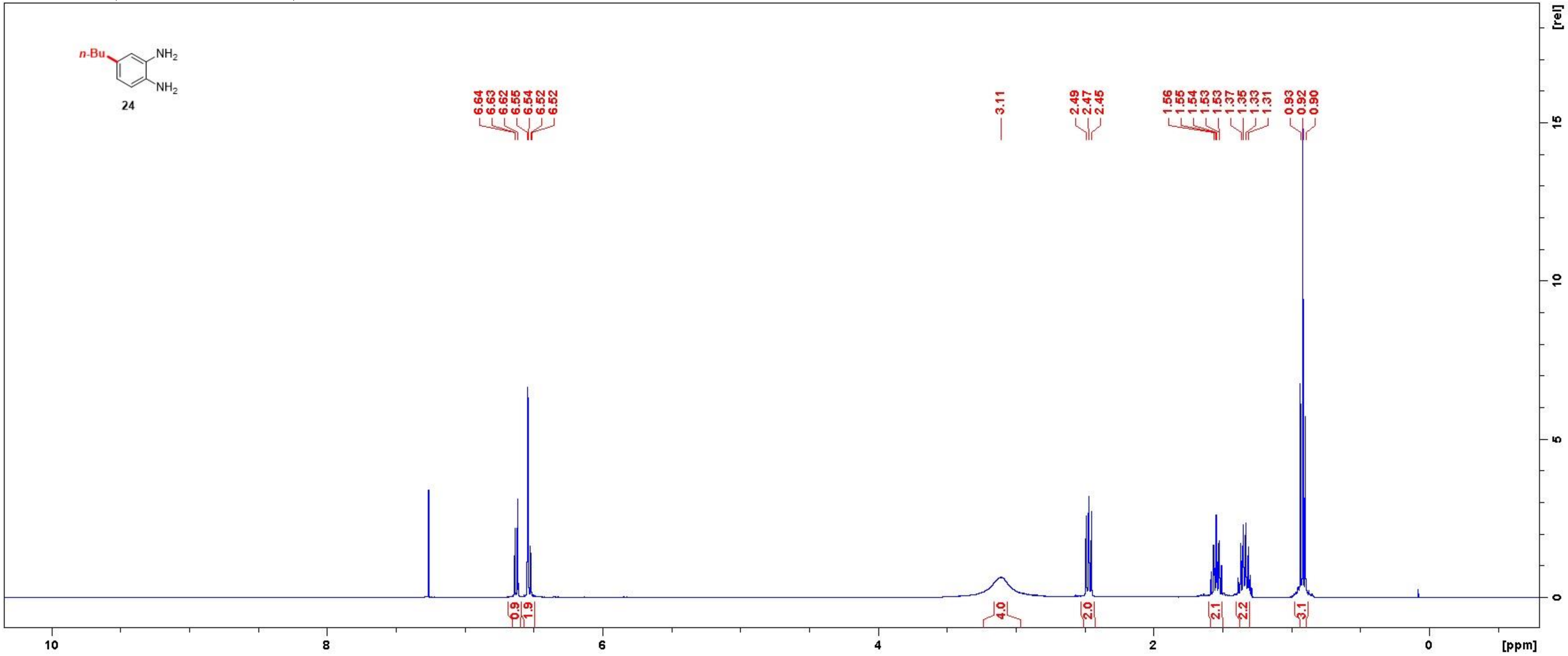

4-butylbenzene-1,2-diamine (24)

$^{13}C\{^{1}H\}$NMR ($CDCl_3$, 100MHz)

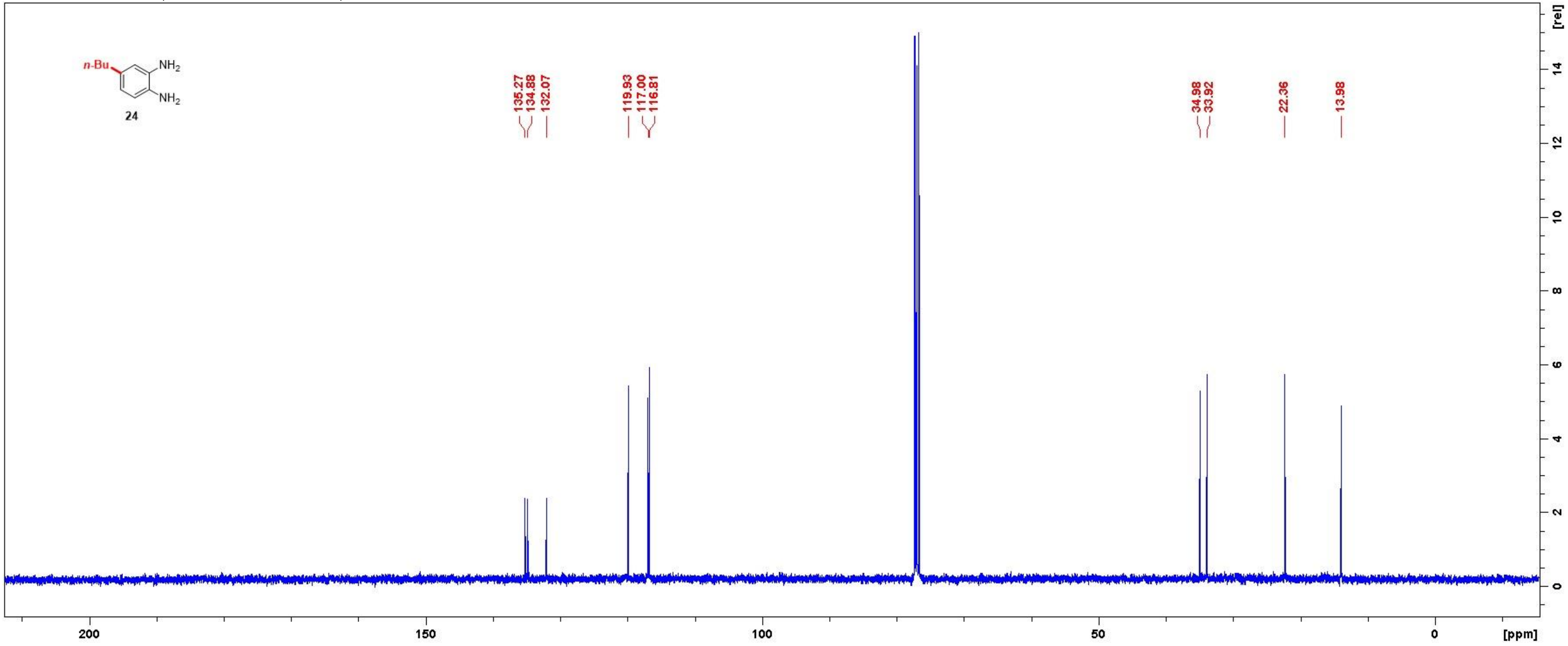

***N*,*N*,2-trimethylaniline (25)**

^{1}H NMR ($CDCl_3$, 400MHz)

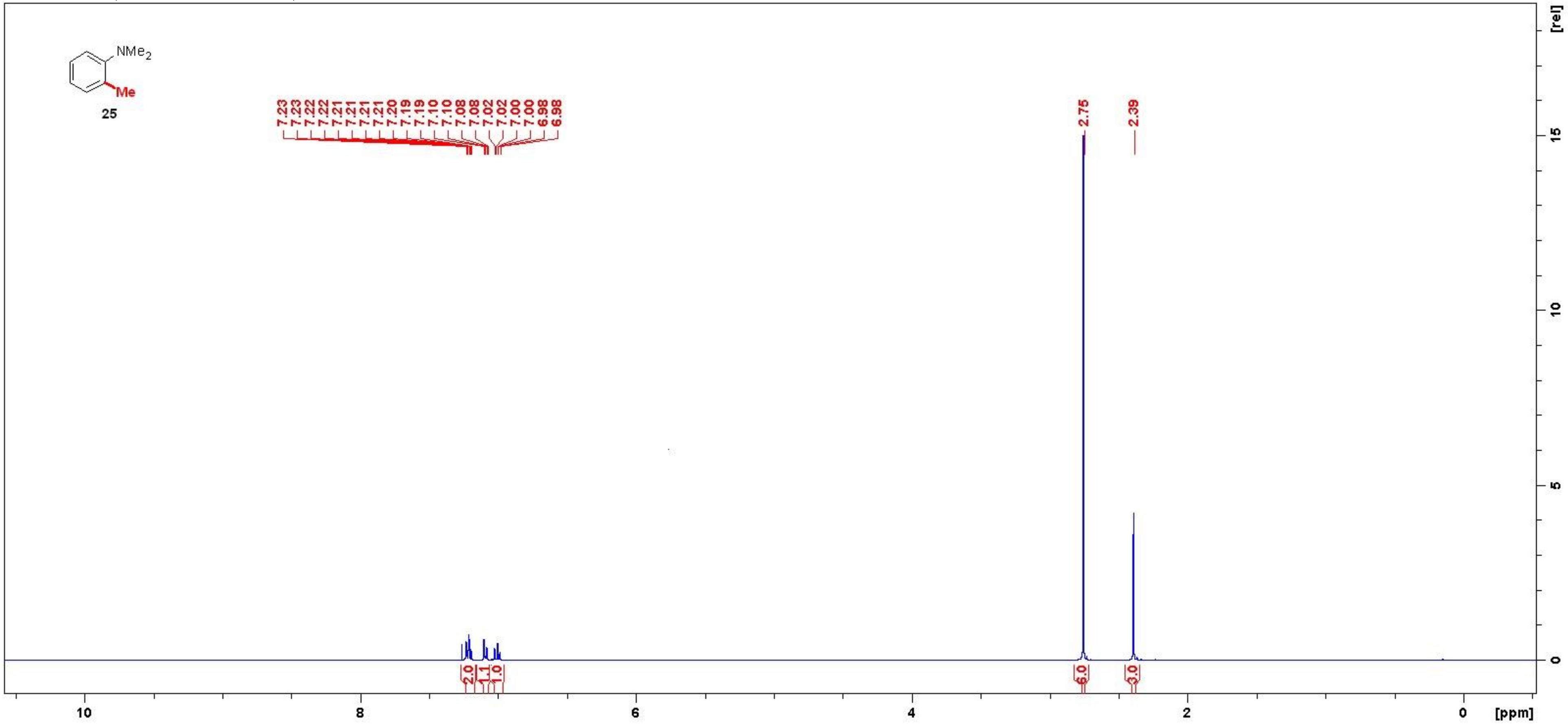

***N*,*N*,2-trimethylaniline (25)**

$^{13}C\{^1H\}$NMR ($CDCl_3$, 100MHz)

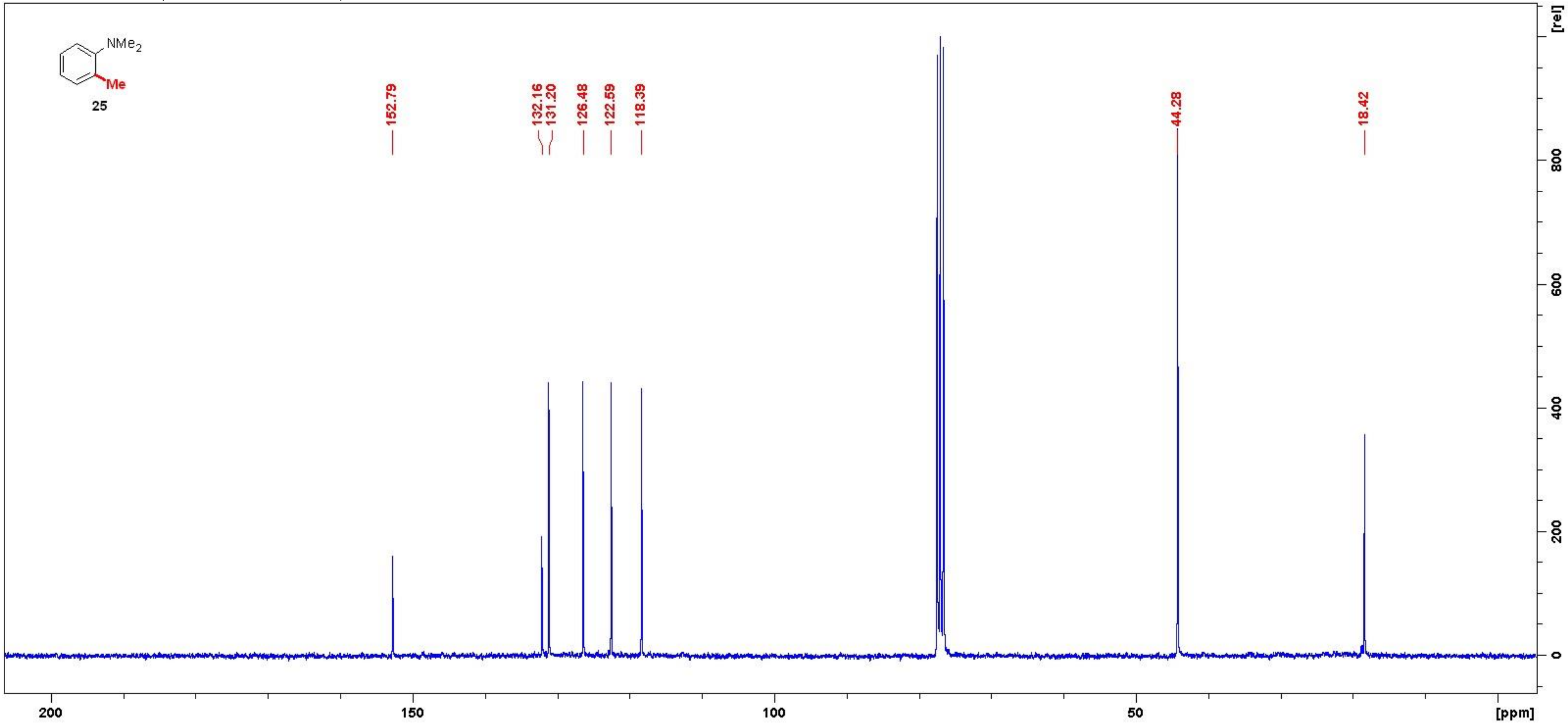

2-butyl-*N*,*N*-dimethylaniline (26)

^{1}H NMR ($CDCl_3$, 400MHz)

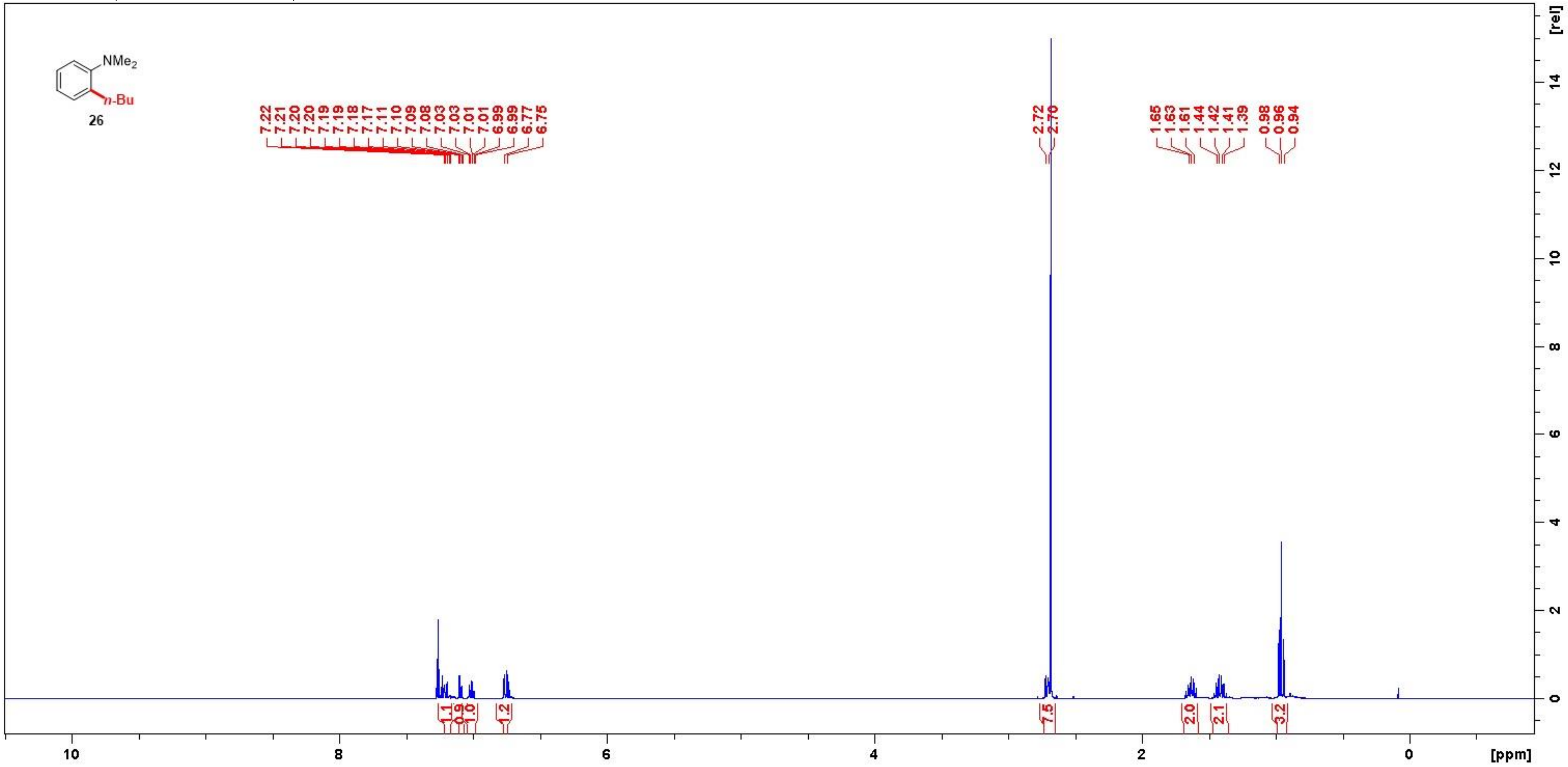

2-butyl-*N*,*N*-dimethylaniline (26)

$^{13}C\{^1H\}$NMR ($CDCl_3$, 100MHz)

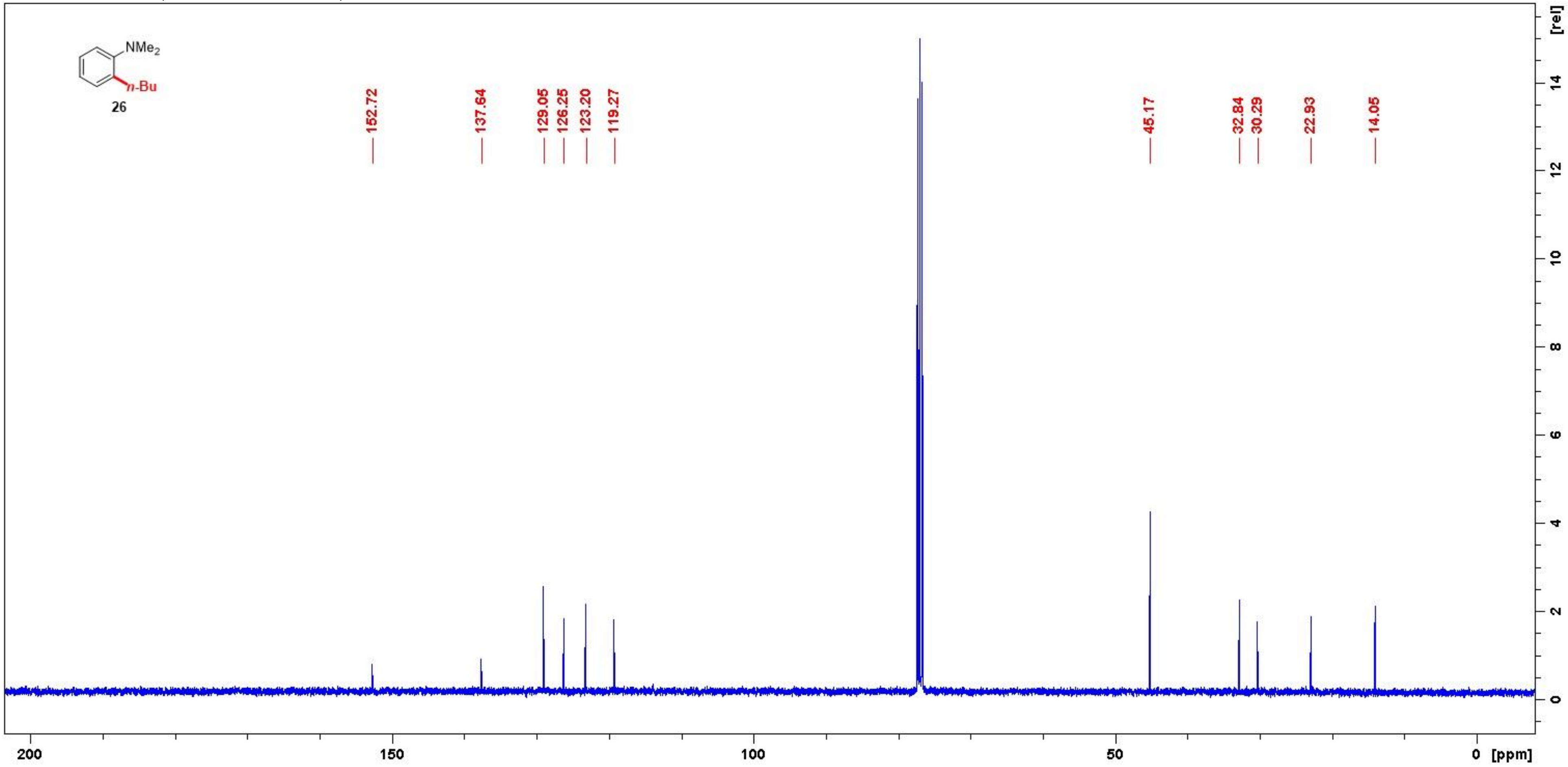

1-(*p*-tolyl)piperidine (27)

^{1}H NMR ($CDCl_3$, 400MHz)

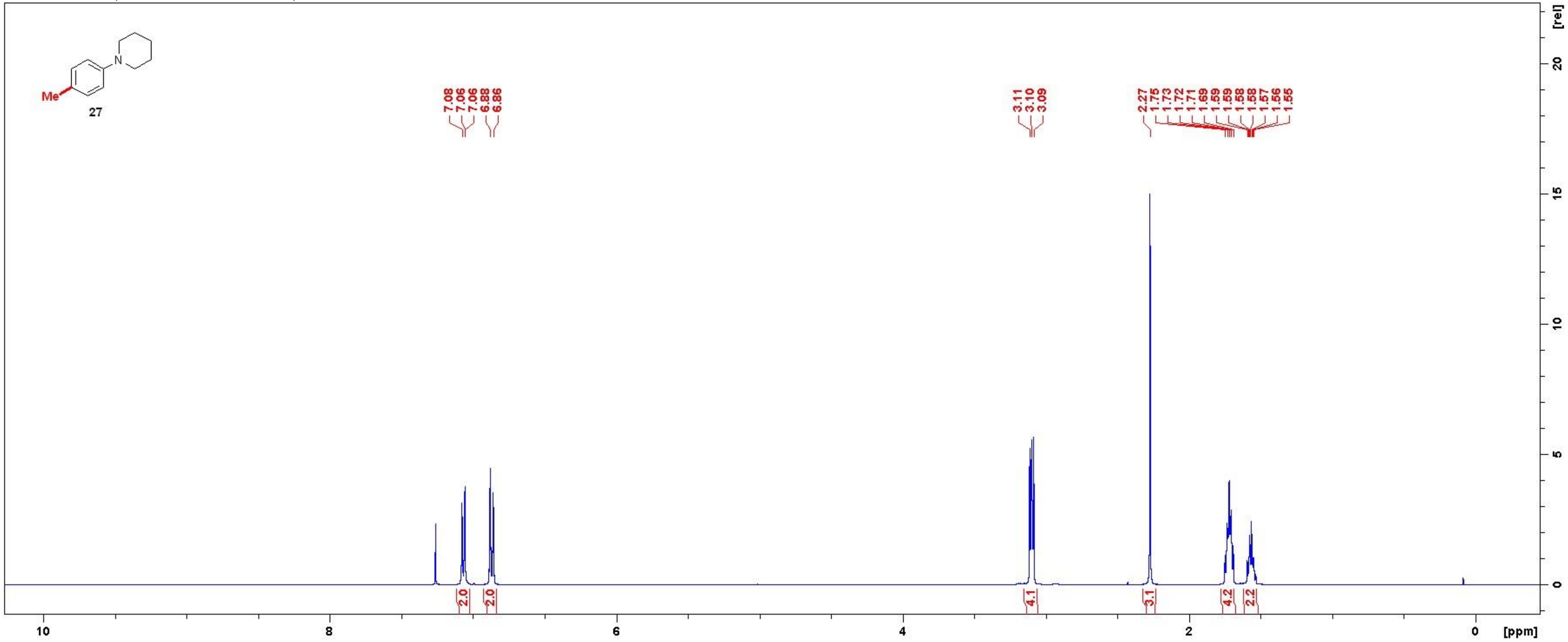

1-(*p*-tolyl)piperidine (27)

$^{13}C\{^{1}H\}$NMR ($CDCl_3$, 100MHz)

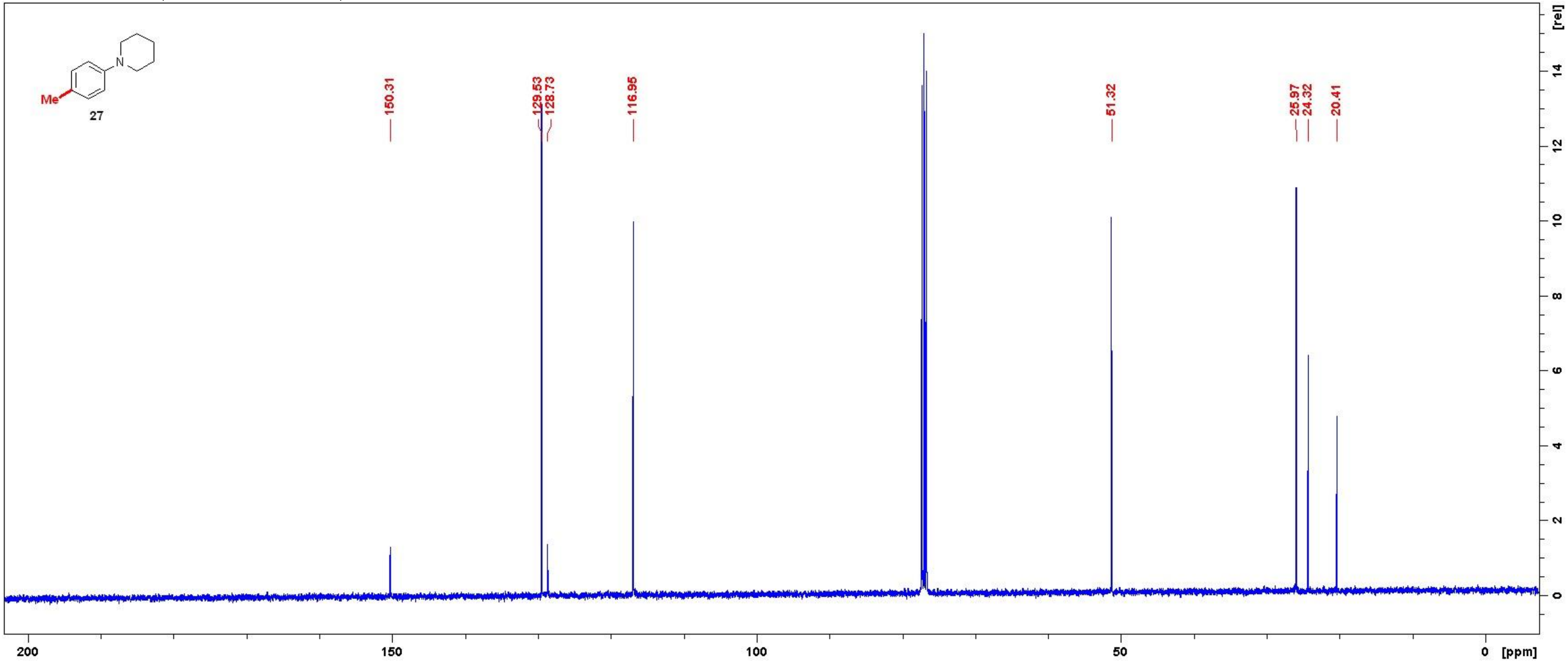

1-(4-butylphenyl)piperidine (28)

^{1}H NMR ($CDCl_3$, 400MHz)

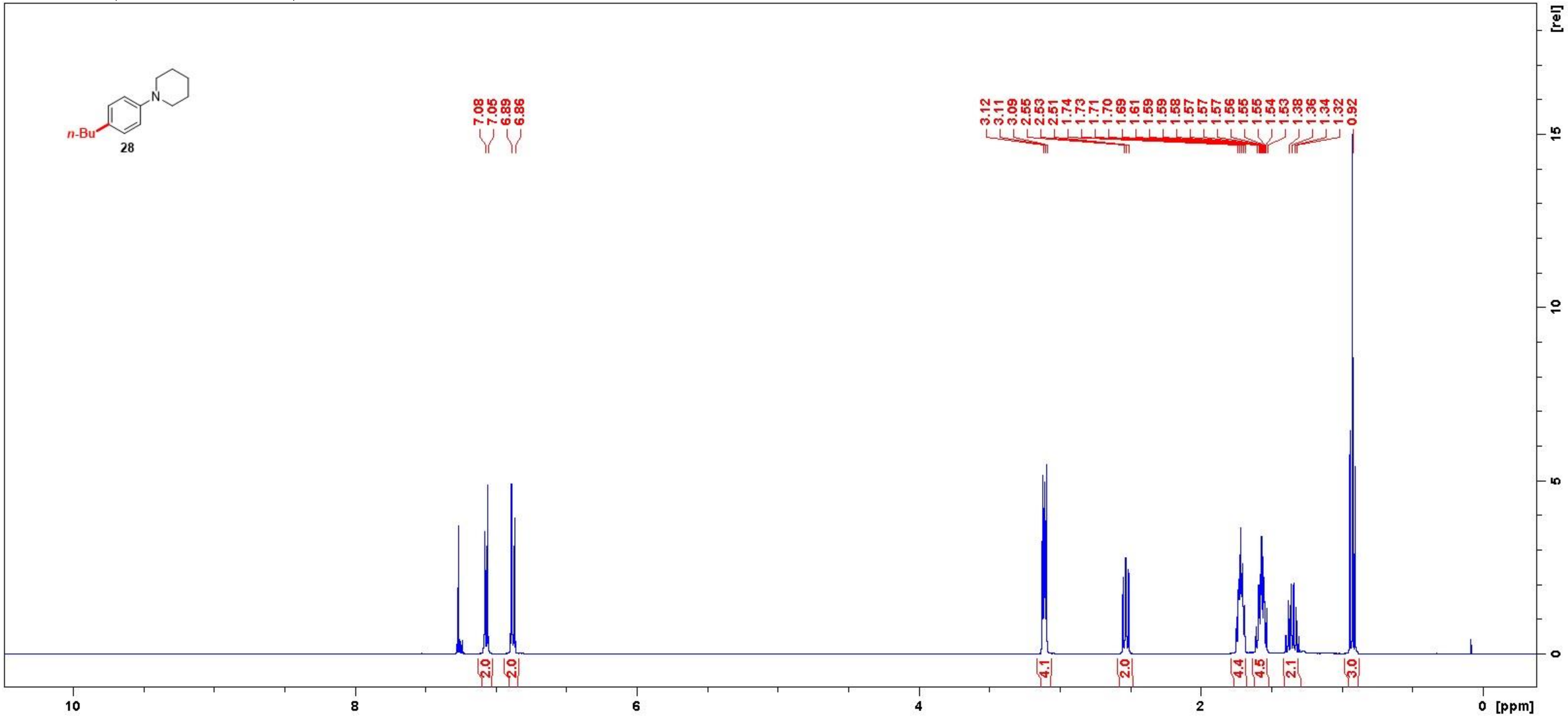

1-(4-butylphenyl)piperidine (28)

$^{13}C\{^{1}H\}$NMR ($CDCl_3$, 100MHz)

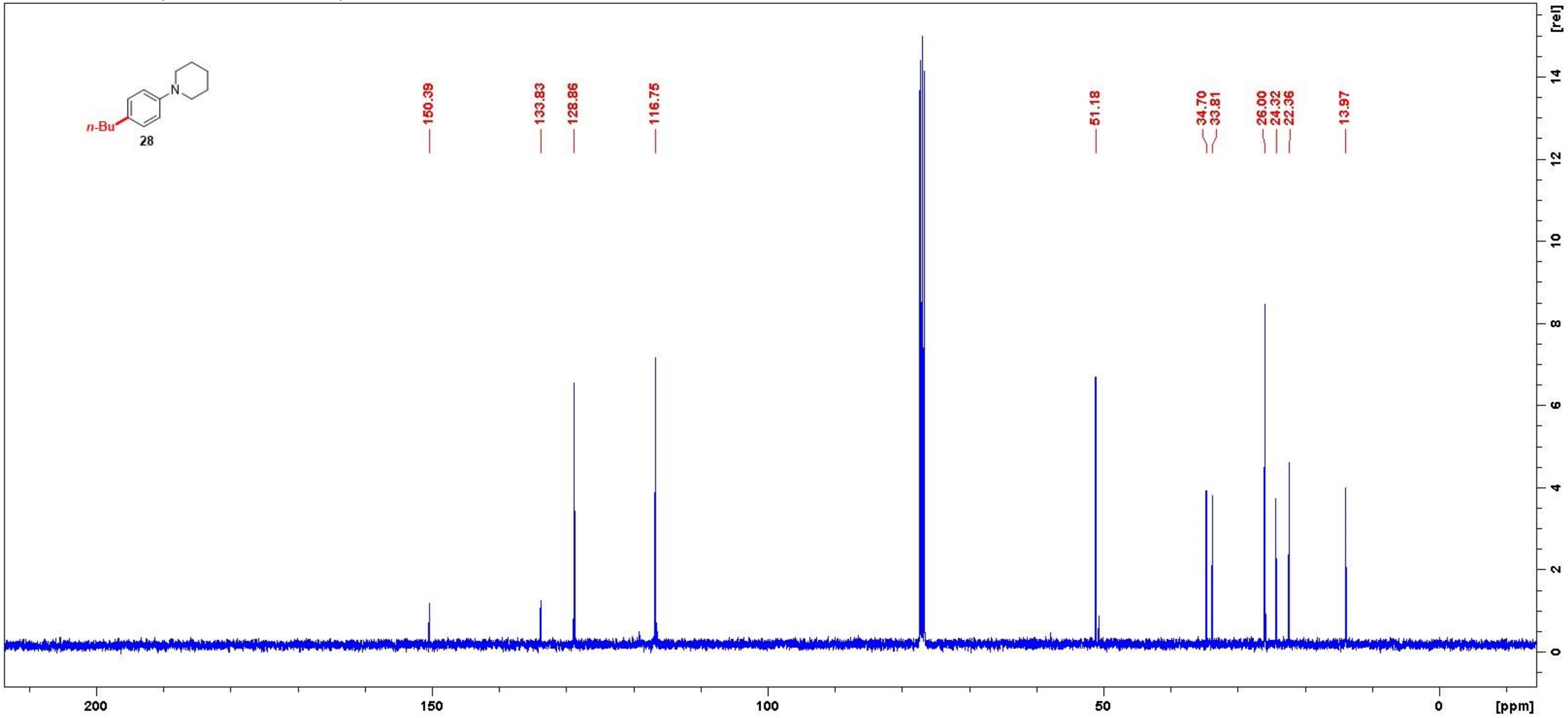

4-methyl-*N*-phenylaniline (29)

^{1}H NMR ($CDCl_3$, 400MHz)

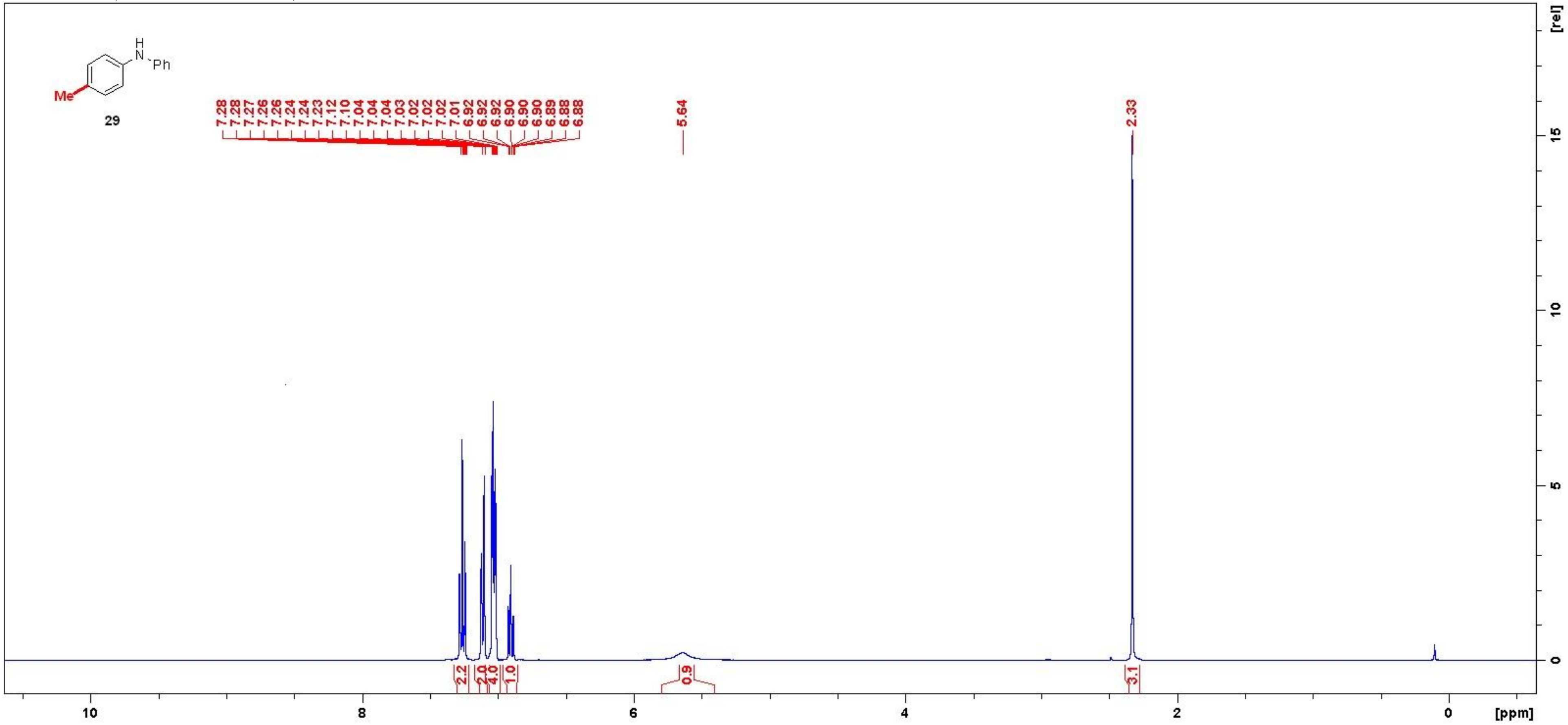

4-methyl-*N*-phenylaniline (29)

$^{13}C\{^{1}H\}$NMR ($CDCl_3$, 100MHz)

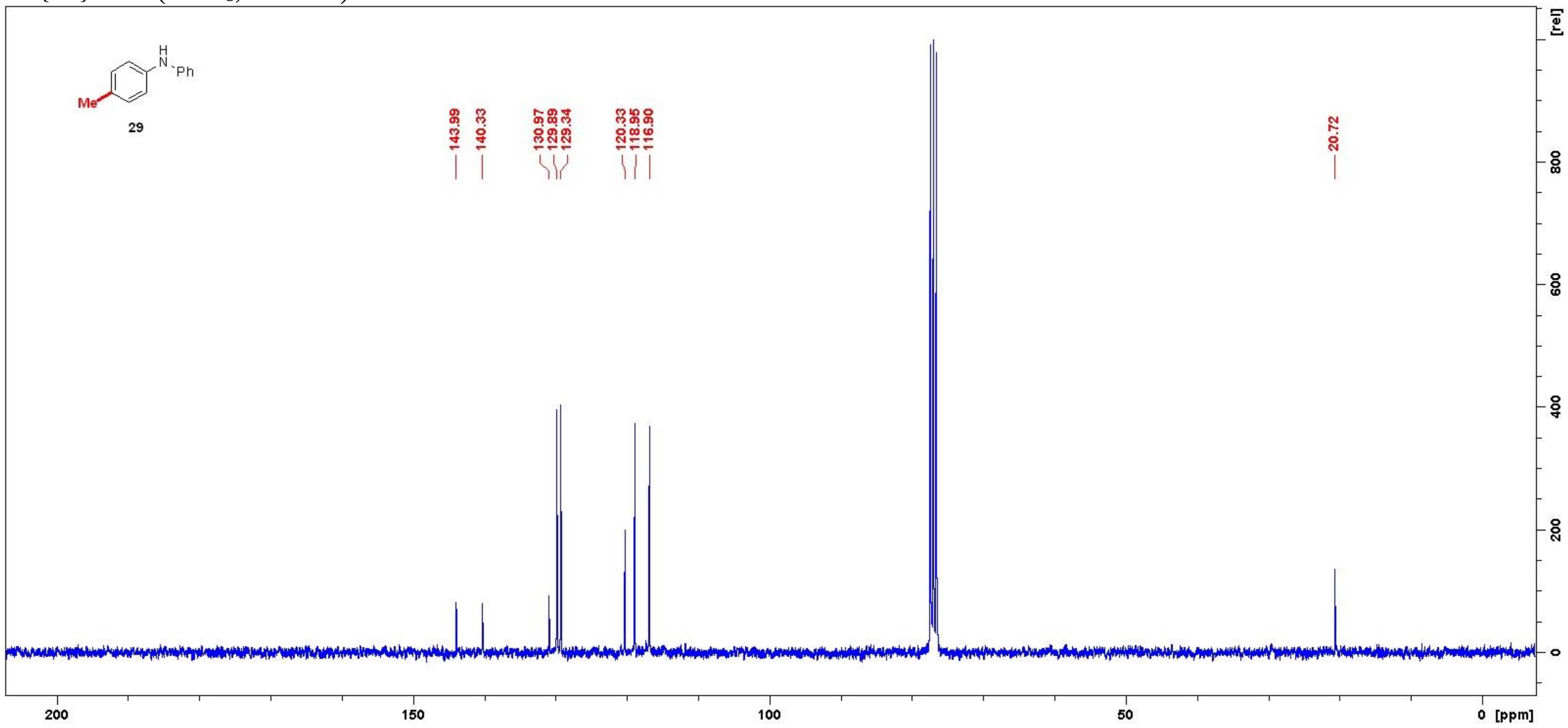

4-butyl-*N*-phenylaniline (30)

^{1}H NMR ($CDCl_3$, 400MHz)

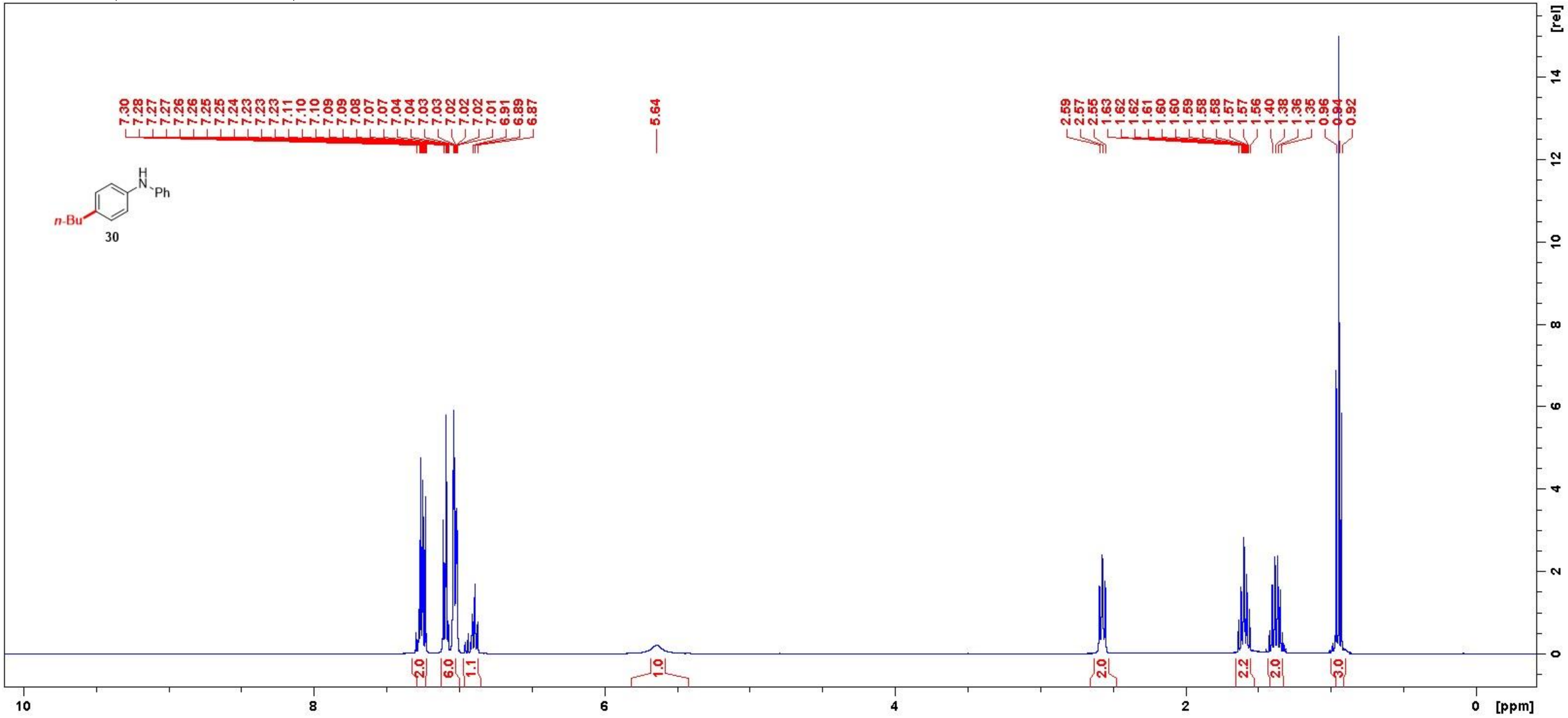

4-butyl-*N*-phenylaniline (30)

$^{13}C\{^{1}H\}NMR$ ($CDCl_3$, 100MHz)

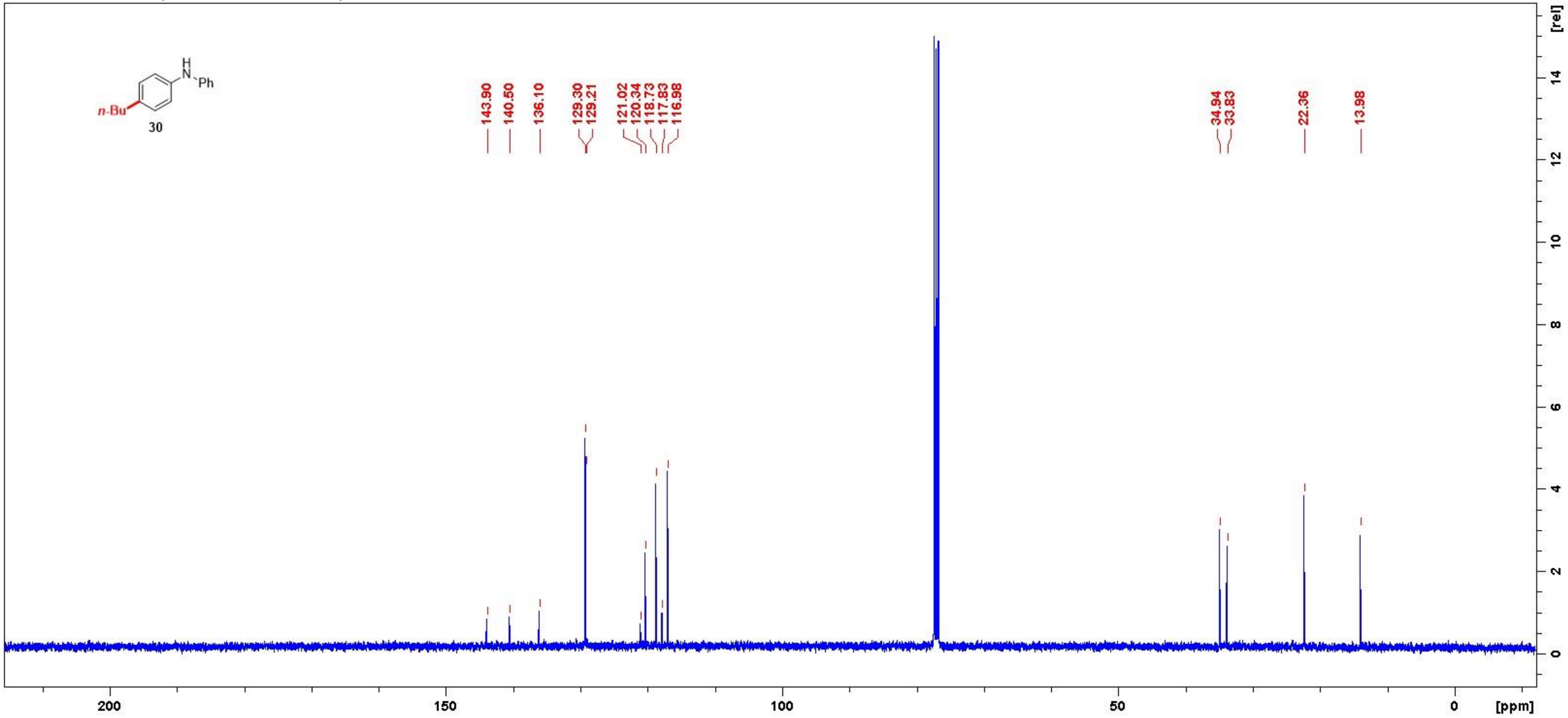

3-methyl-*N*-phenylaniline (31)

^{1}H NMR ($CDCl_3$, 400MHz)

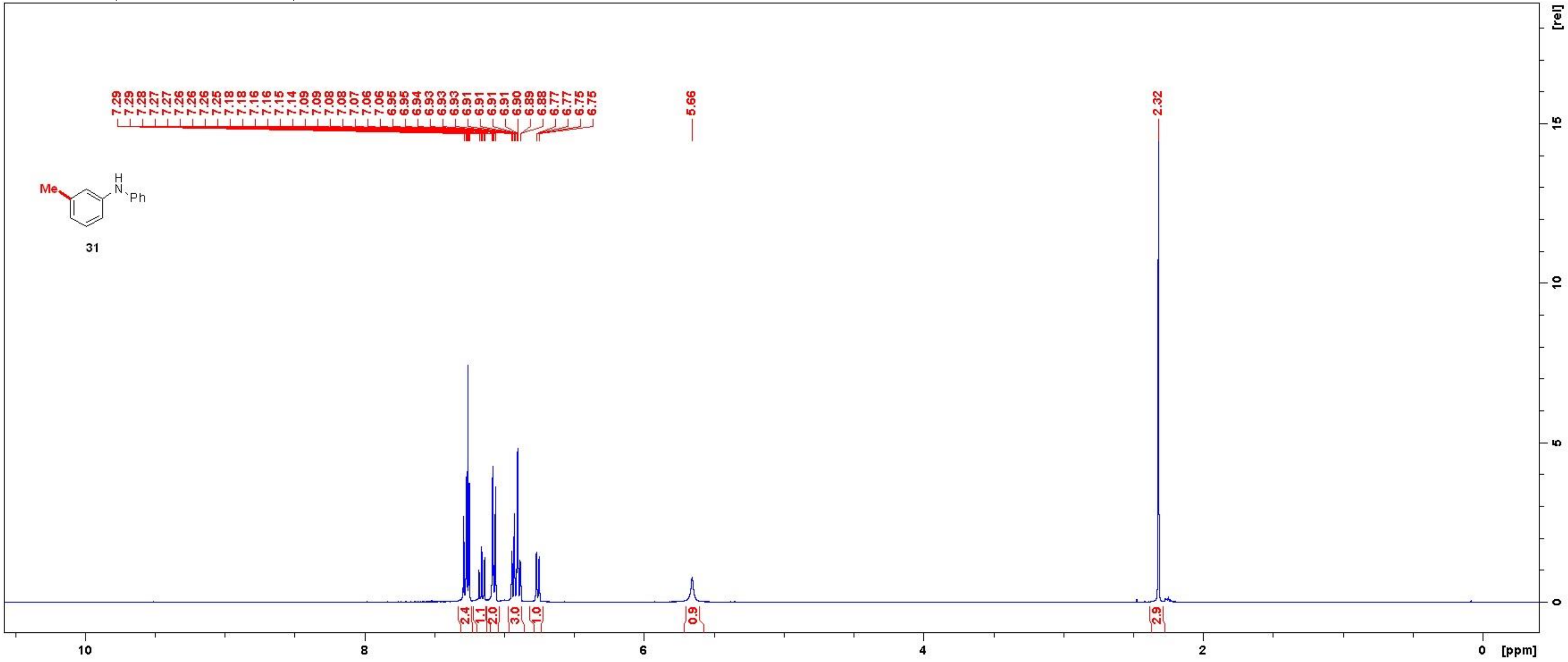

3-methyl-*N*-phenylaniline (31)

$^{13}C\{^{1}H\}$NMR ($CDCl_3$, 100MHz)

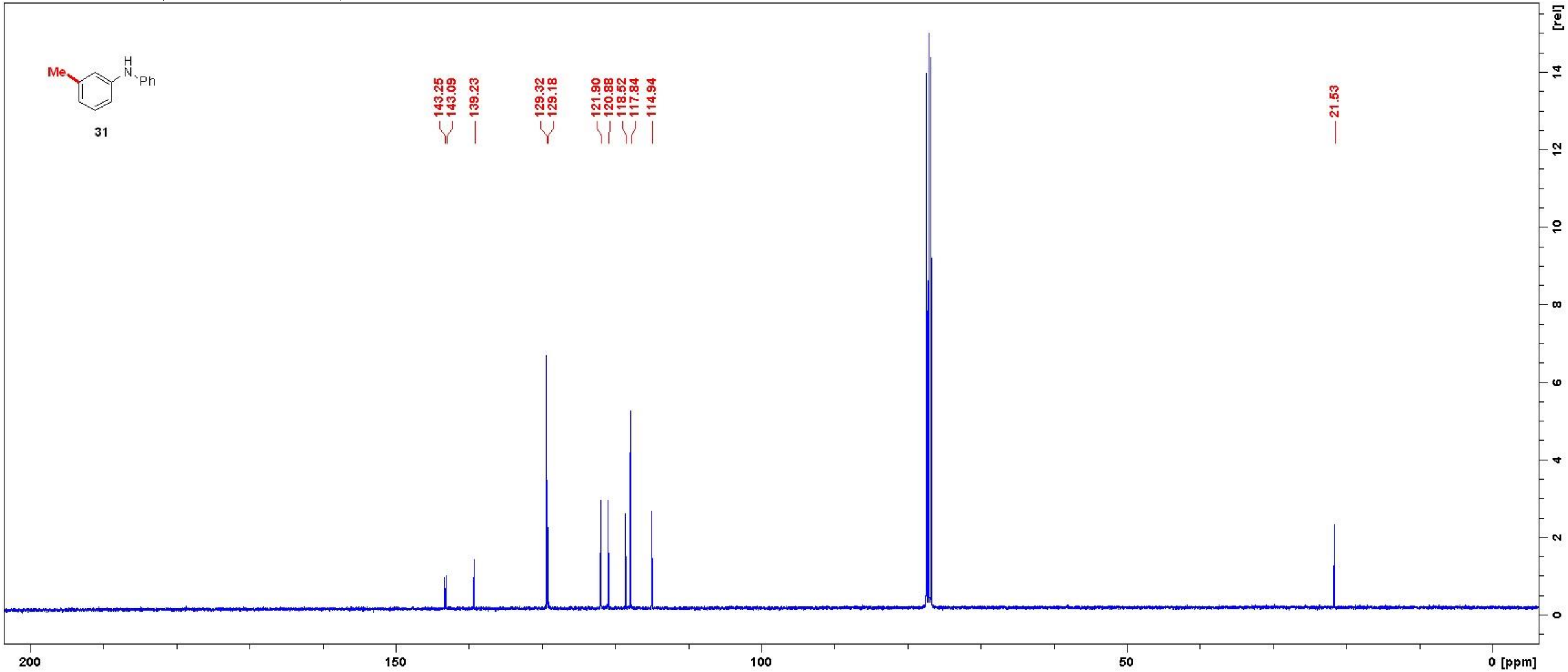

3-butyl-*N*-phenylaniline (32)

^{1}H NMR ($CDCl_3$, 400MHz)

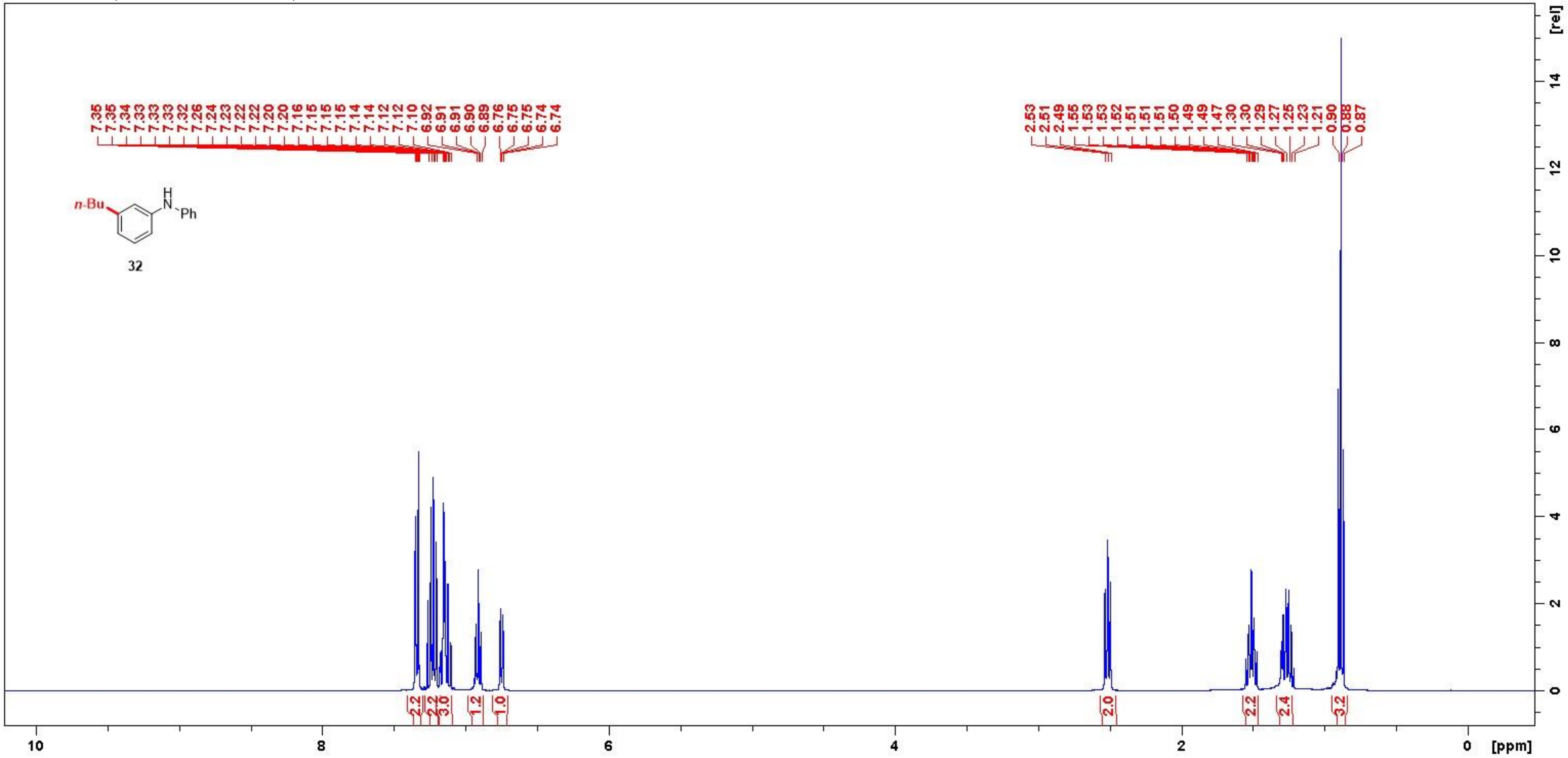

3-butyl-*N*-phenylaniline (32)

$^{13}C\{^{1}H\}$NMR ($CDCl_3$, 100MHz)

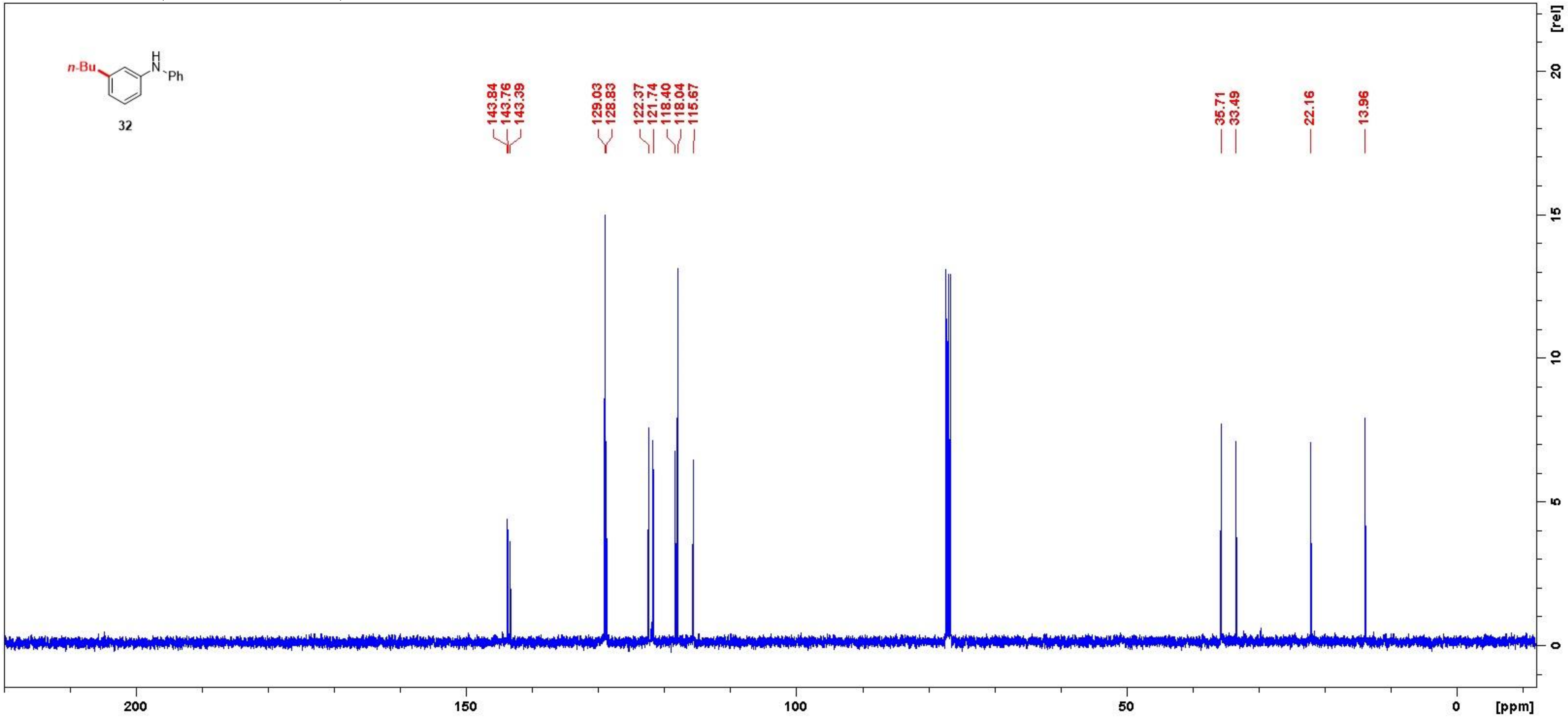

3-methyl-*N*,*N*-diphenylaniline (33)

^{1}H NMR ($CDCl_3$, 400MHz)

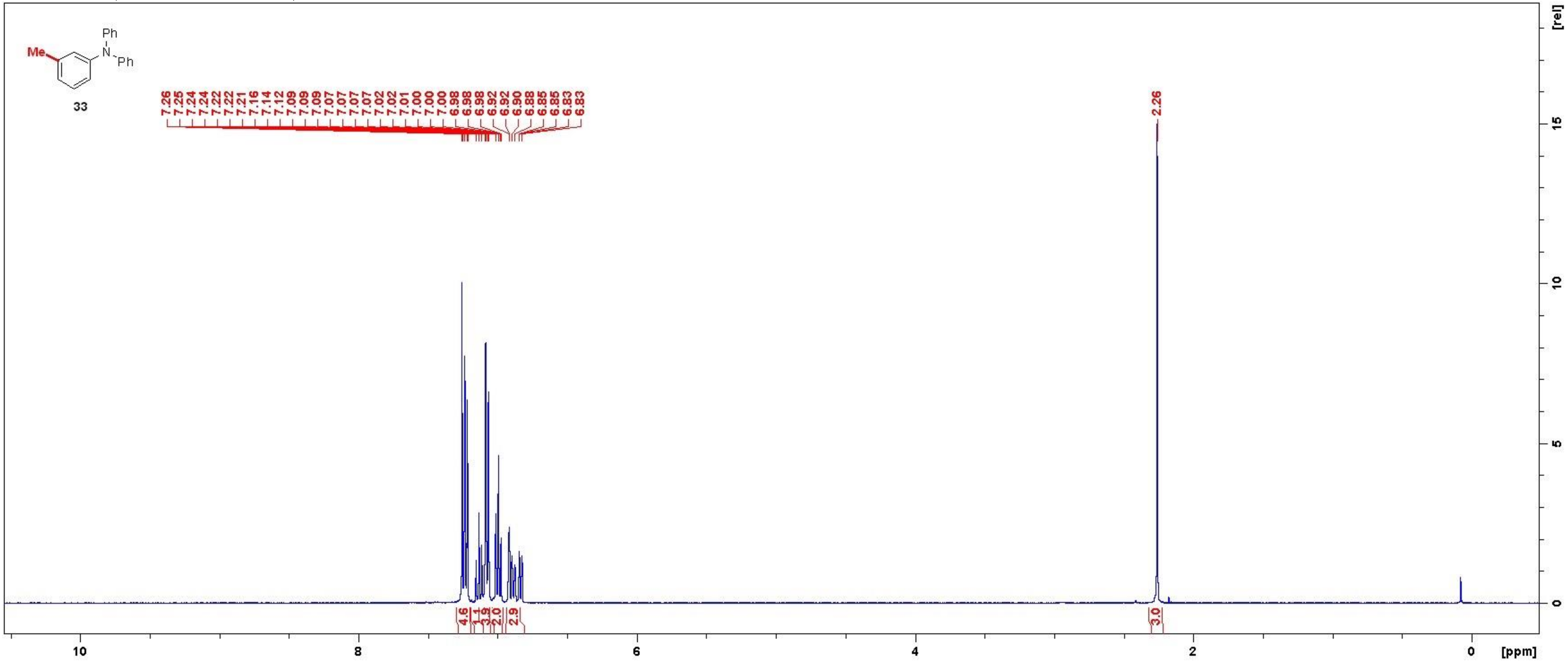

3-methyl-*N*,*N*-diphenylaniline (33)

$^{13}C\{^{1}H\}$NMR ($CDCl_3$, 100MHz)

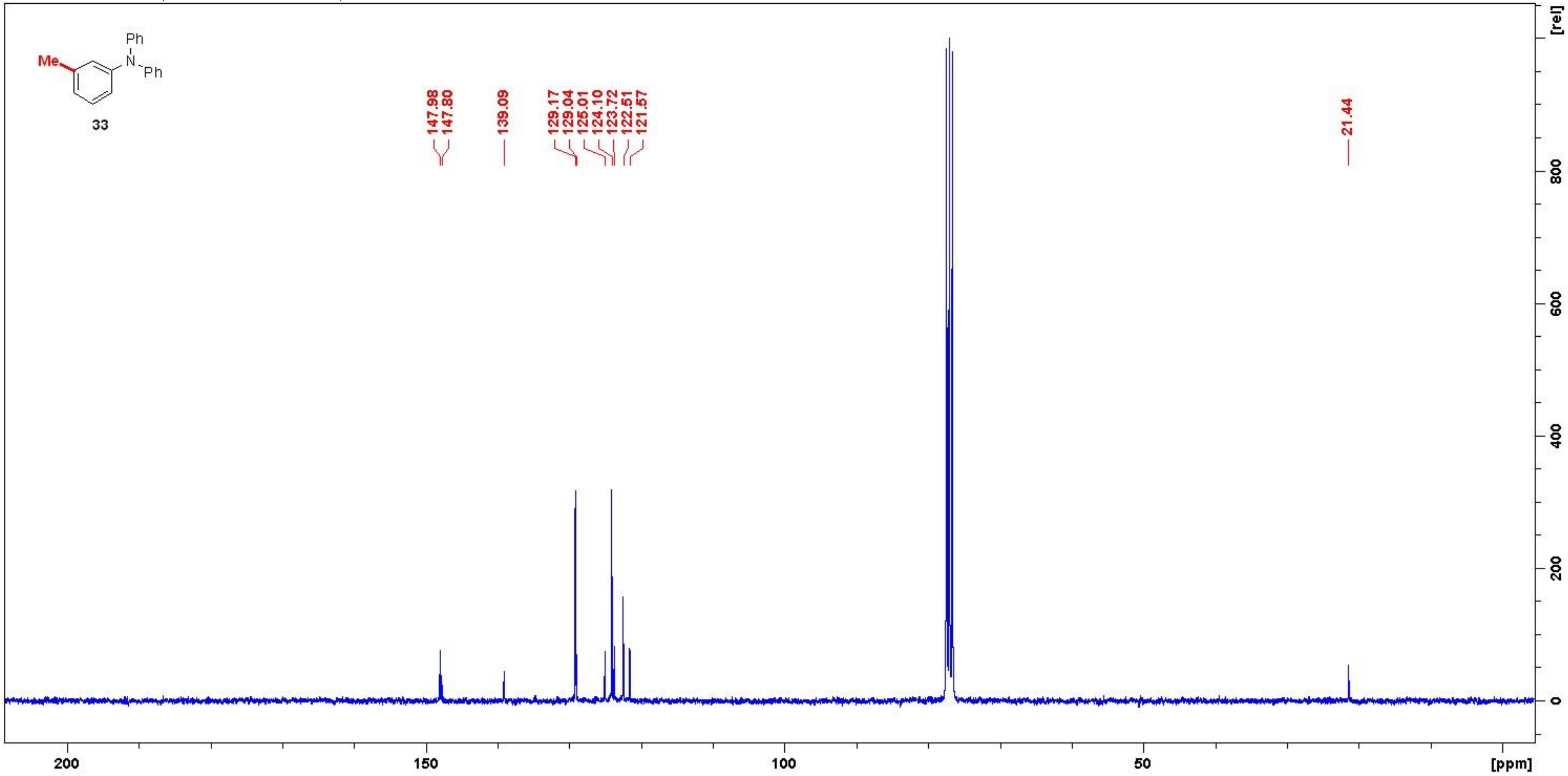

3-butyl-*N*,*N*-diphenylaniline (34)

^{1}H NMR ($CDCl_3$, 400MHz)

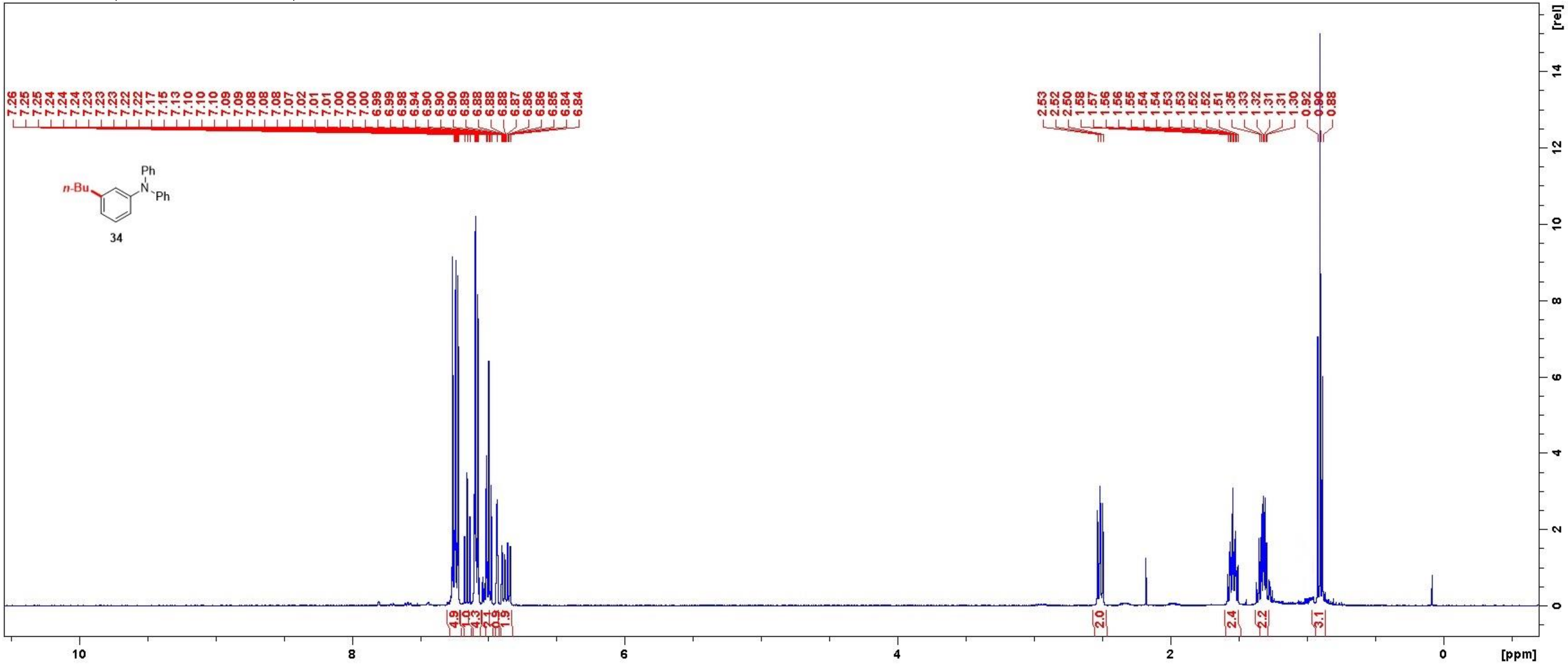

3-butyl-*N*,*N*-diphenylaniline (34)

$^{13}C\{^{1}H\}$NMR ($CDCl_3$, 100MHz)

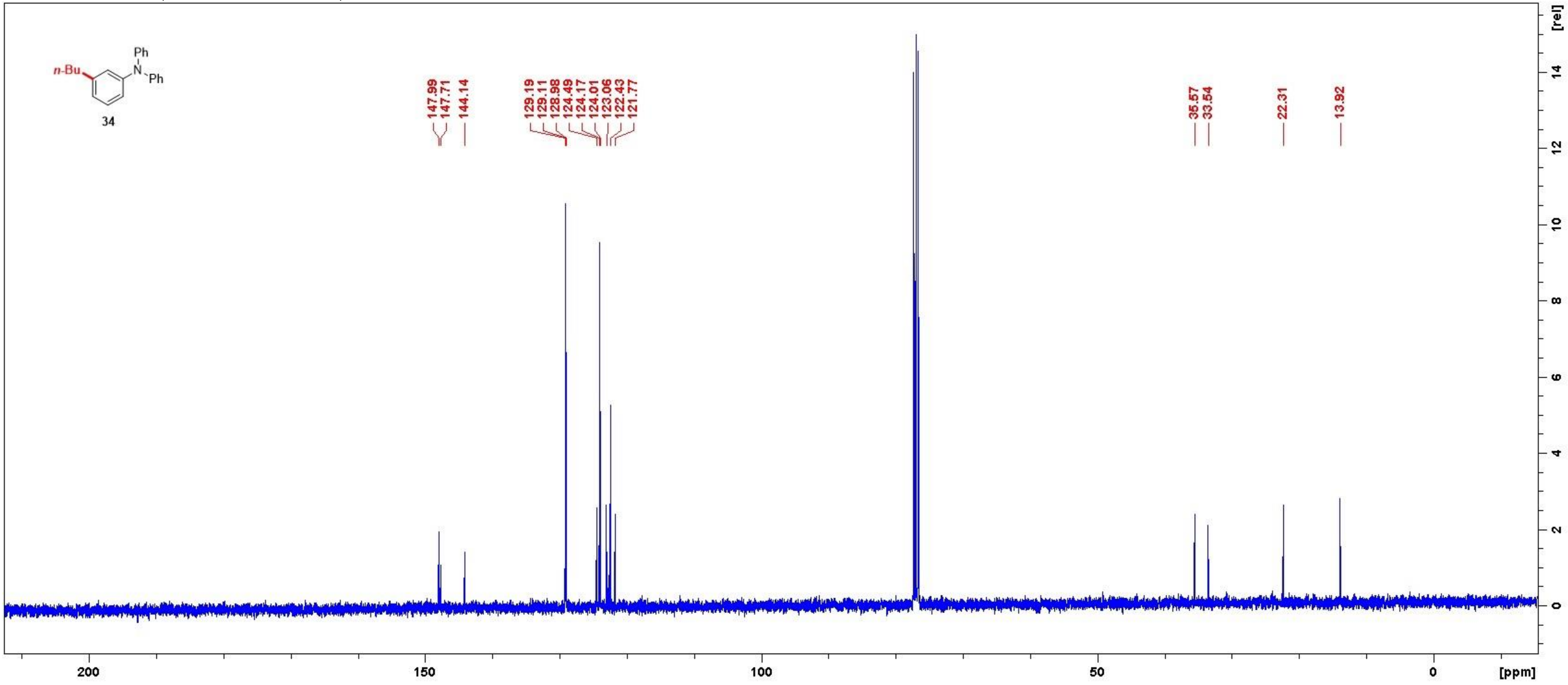

3-methyl-9*H*-carbazole (35)

^{1}H NMR ($CDCl_3$, 400MHz)

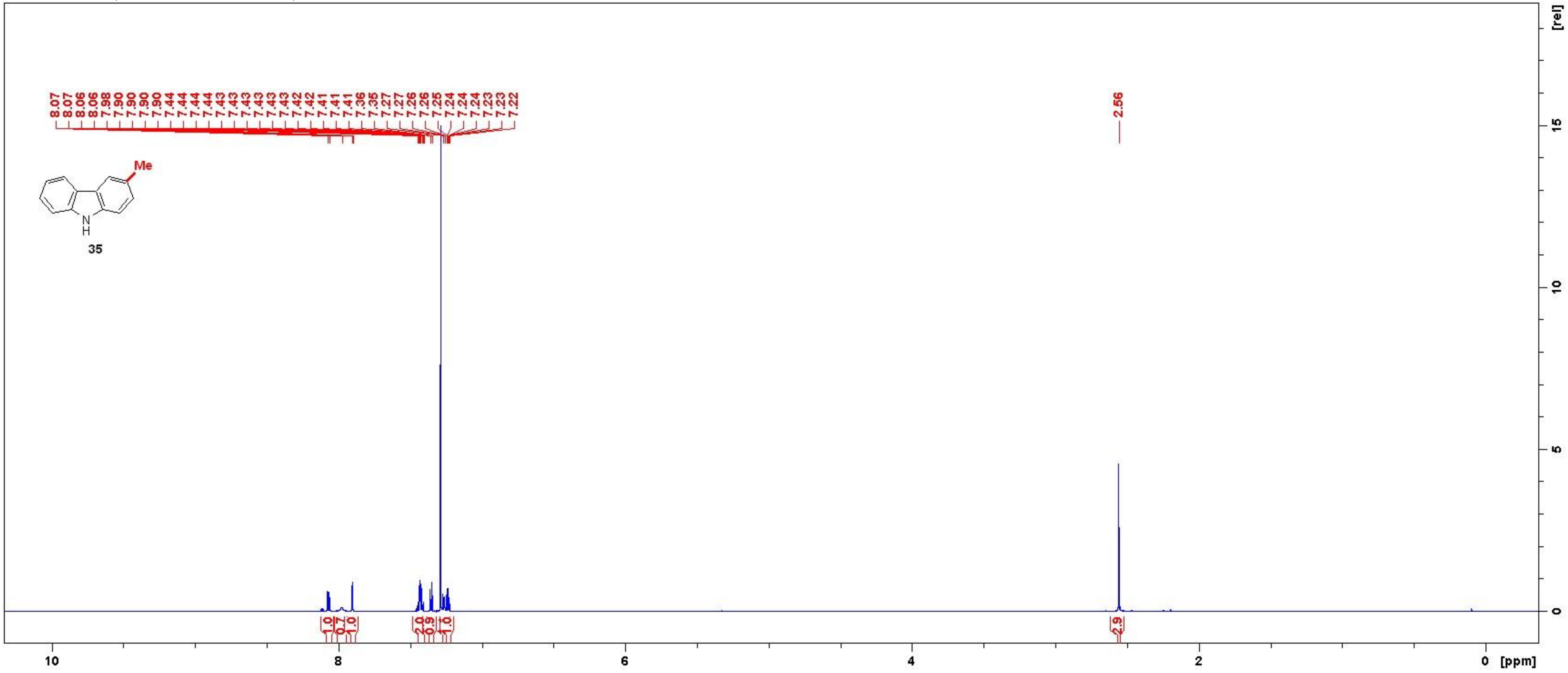

3-methyl-9*H*-carbazole (35)

$^{13}C\{^{1}H\}$NMR ($CDCl_3$, 100MHz)

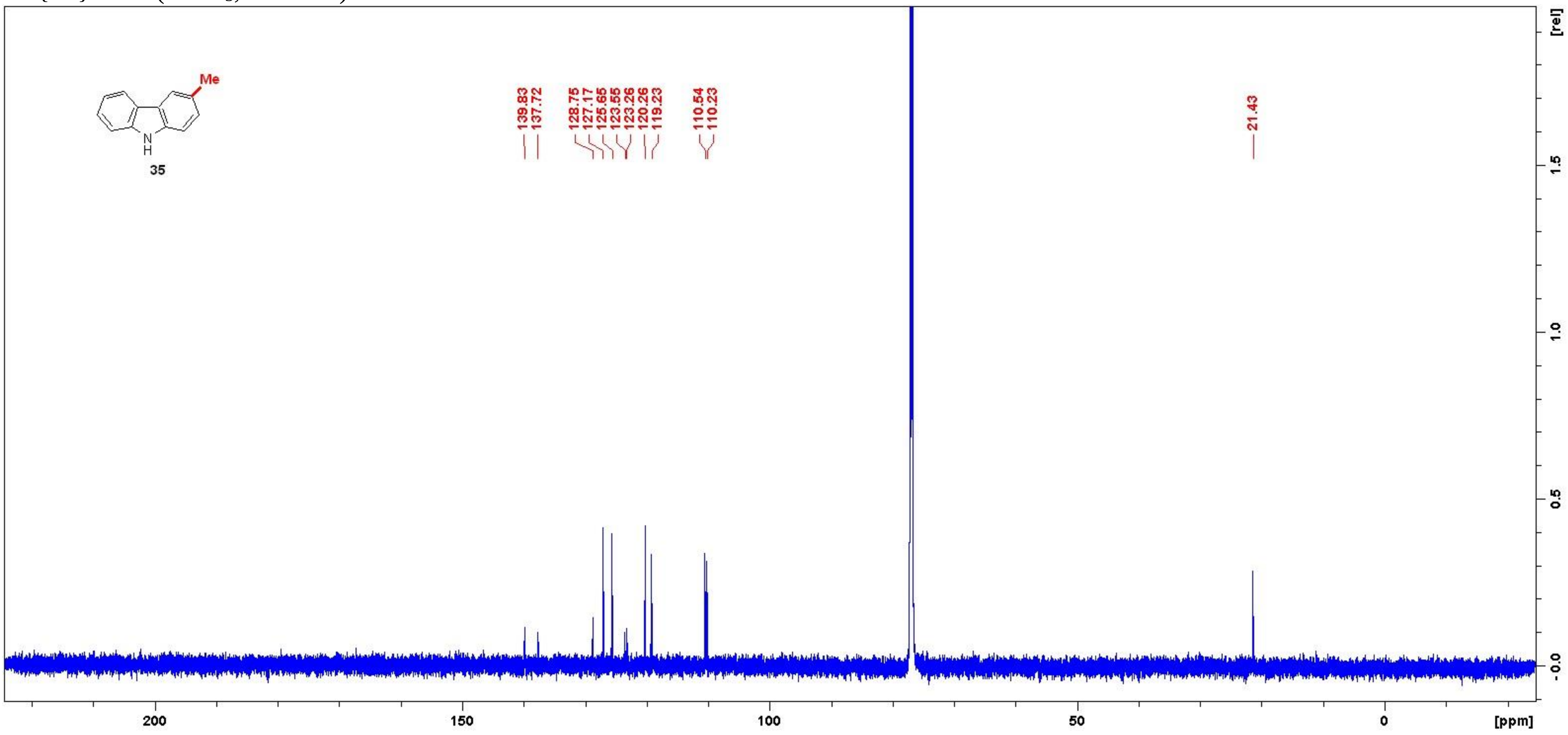

3-butyl-9*H*-carbazole (36)

^{1}H NMR ($CDCl_3$, 400MHz)

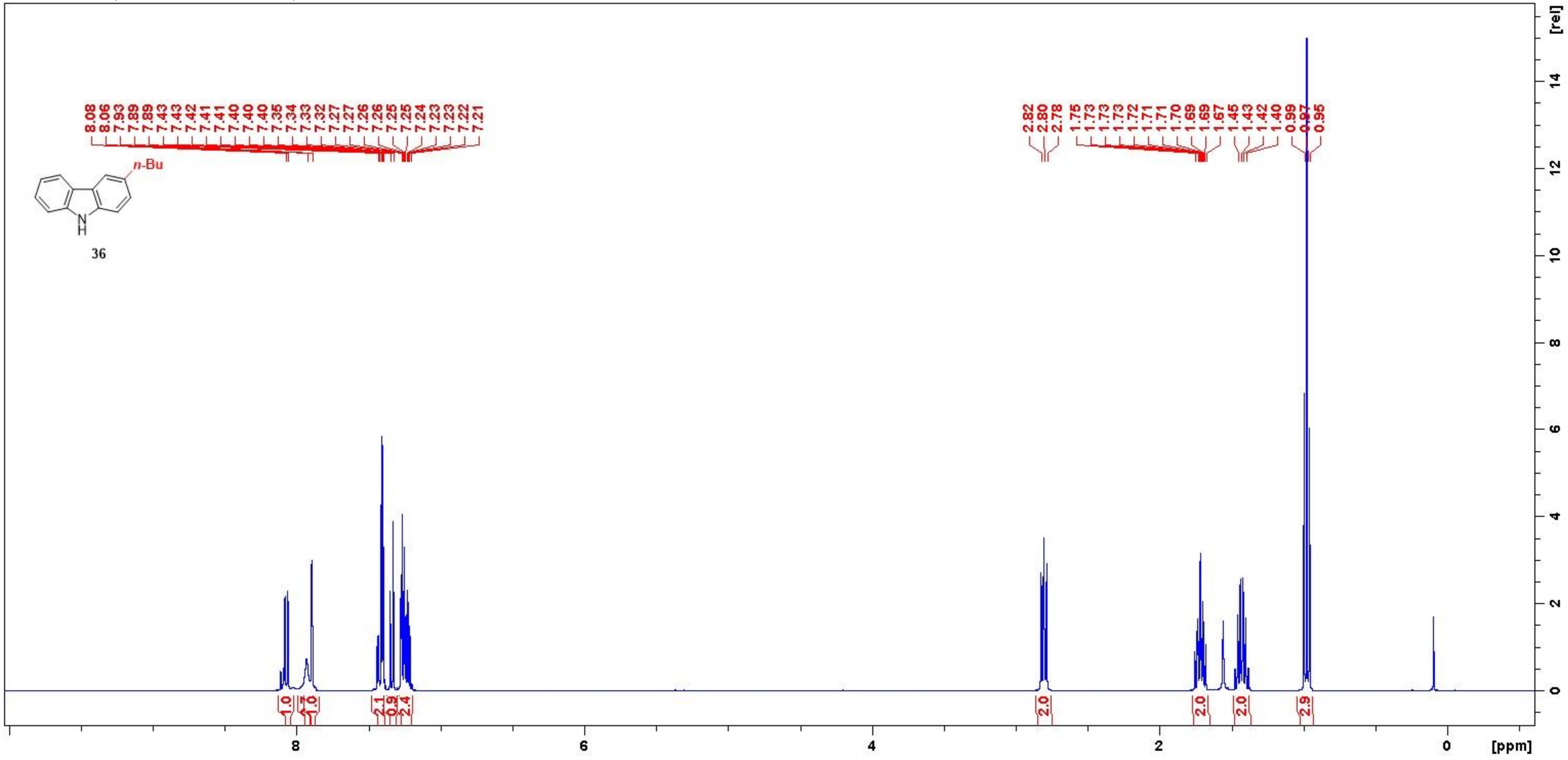

3-butyl-9*H*-carbazole (36)

$^{13}C\{^{1}H\}$NMR ($CDCl_3$, 100MHz)

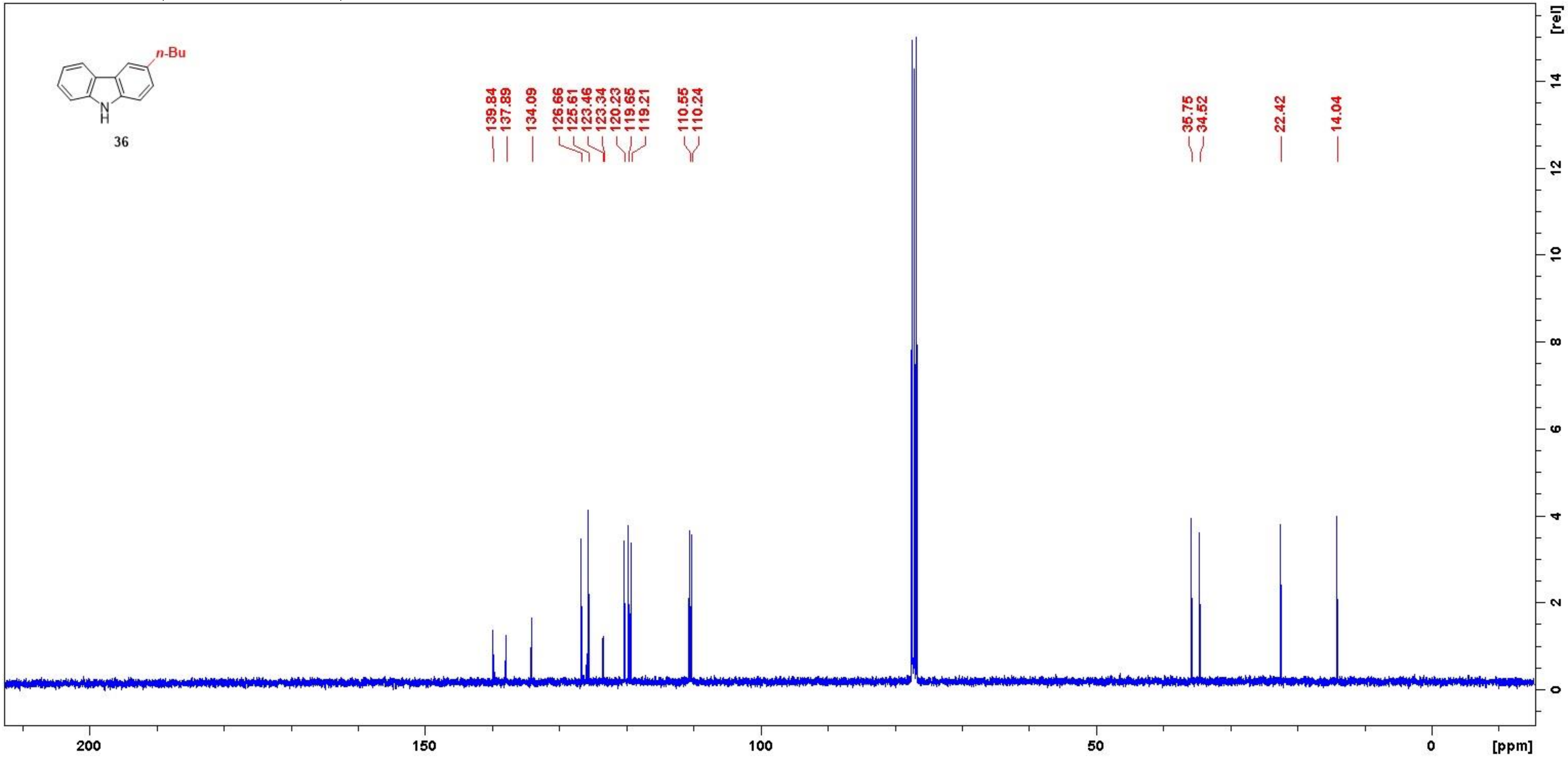

3,6-dimethyl-9*H*-carbazole (37)

^{1}H NMR (CDCl$_3$, 400MHz)

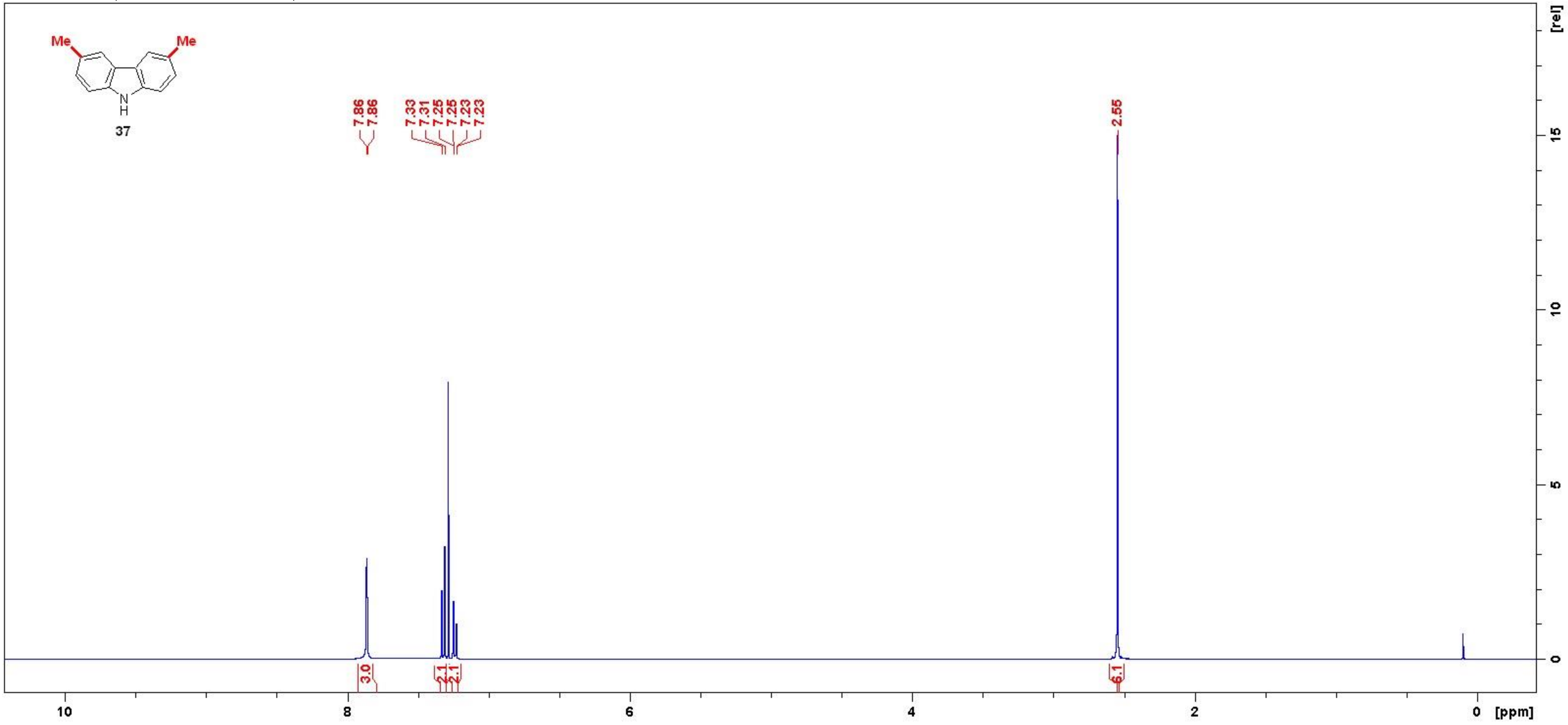

3,6-dimethyl-9*H*-carbazole (37)

$^{13}C\{^{1}H\}$NMR ($CDCl_3$, 100MHz)

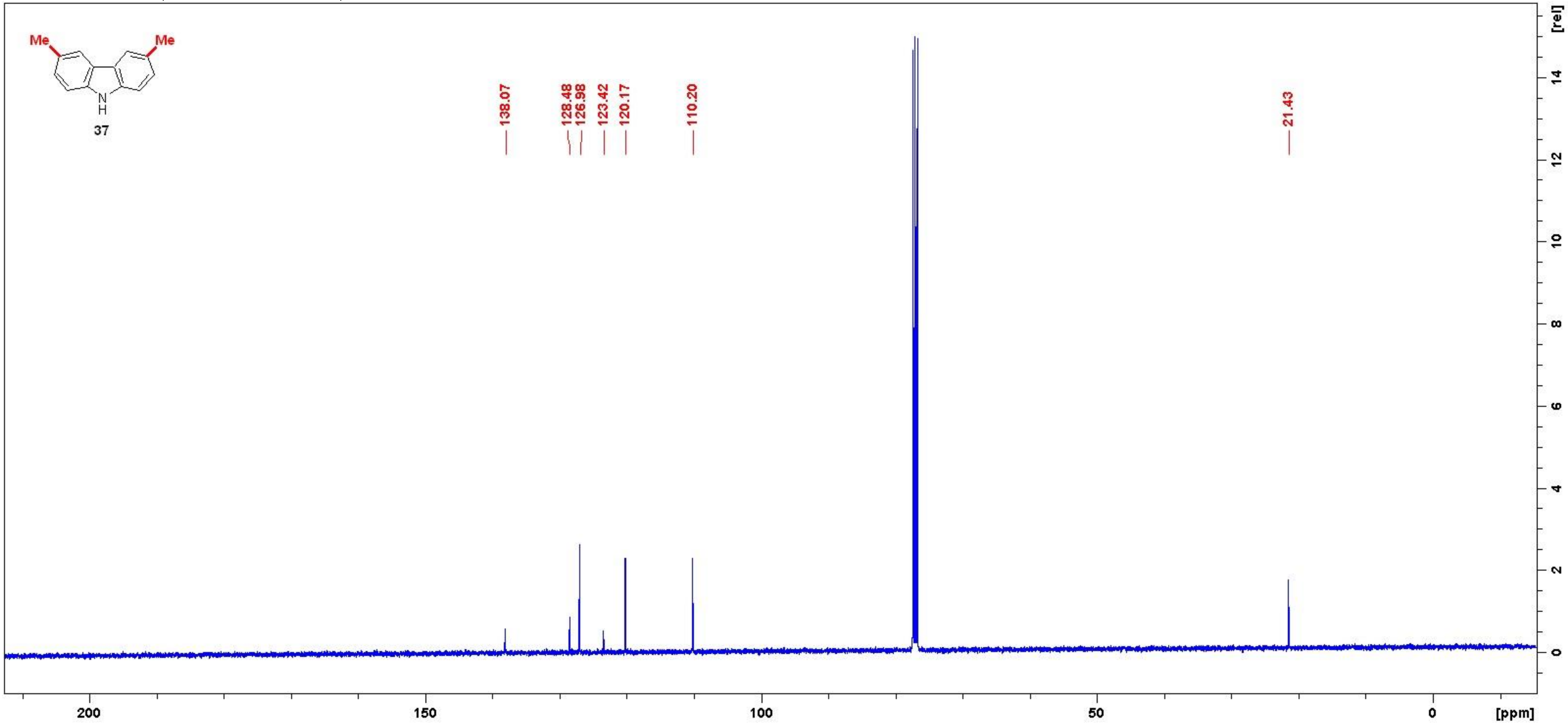

3,6-dibutyl-9*H*-carbazole (38)

^{1}H NMR ($CDCl_3$, 400MHz)

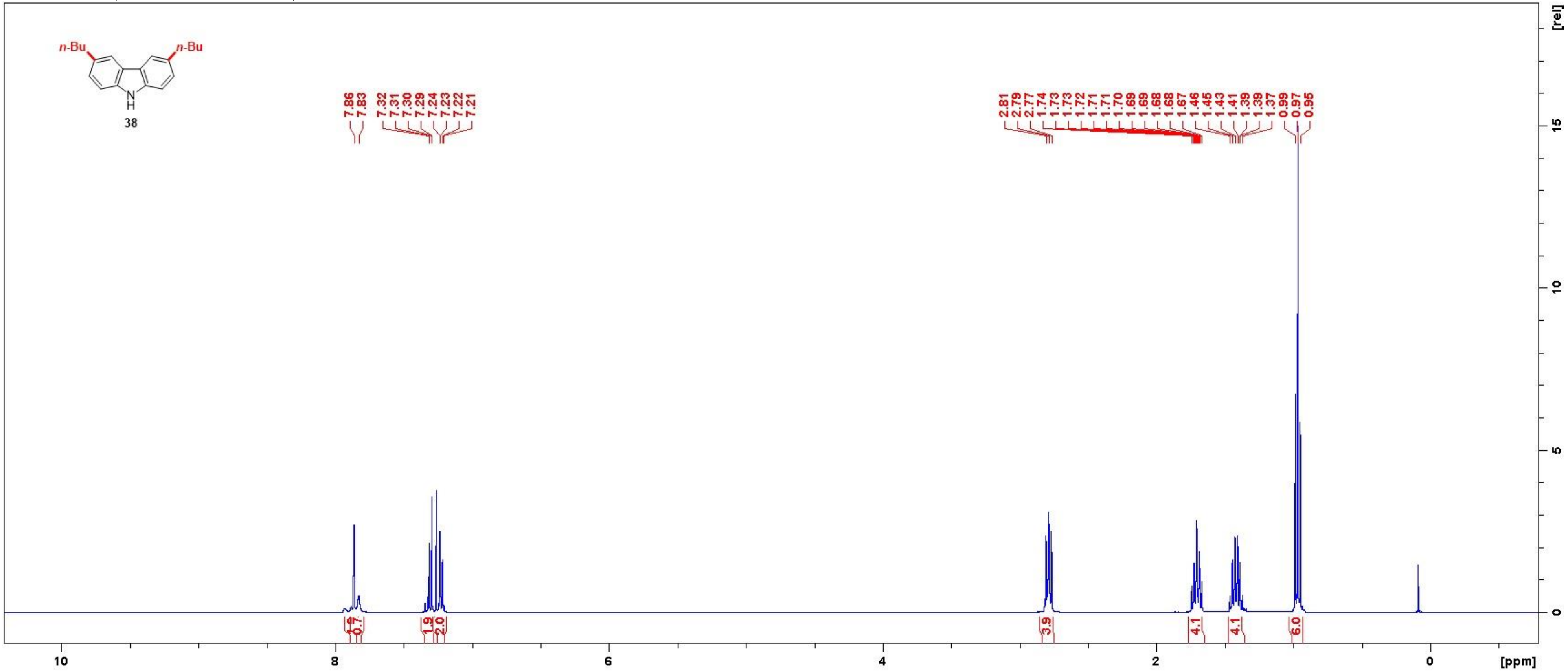

3,6-dibutyl-9*H*-carbazole (38)

$^{13}C\{^{1}H\}NMR$ ($CDCl_3$, 100MHz)

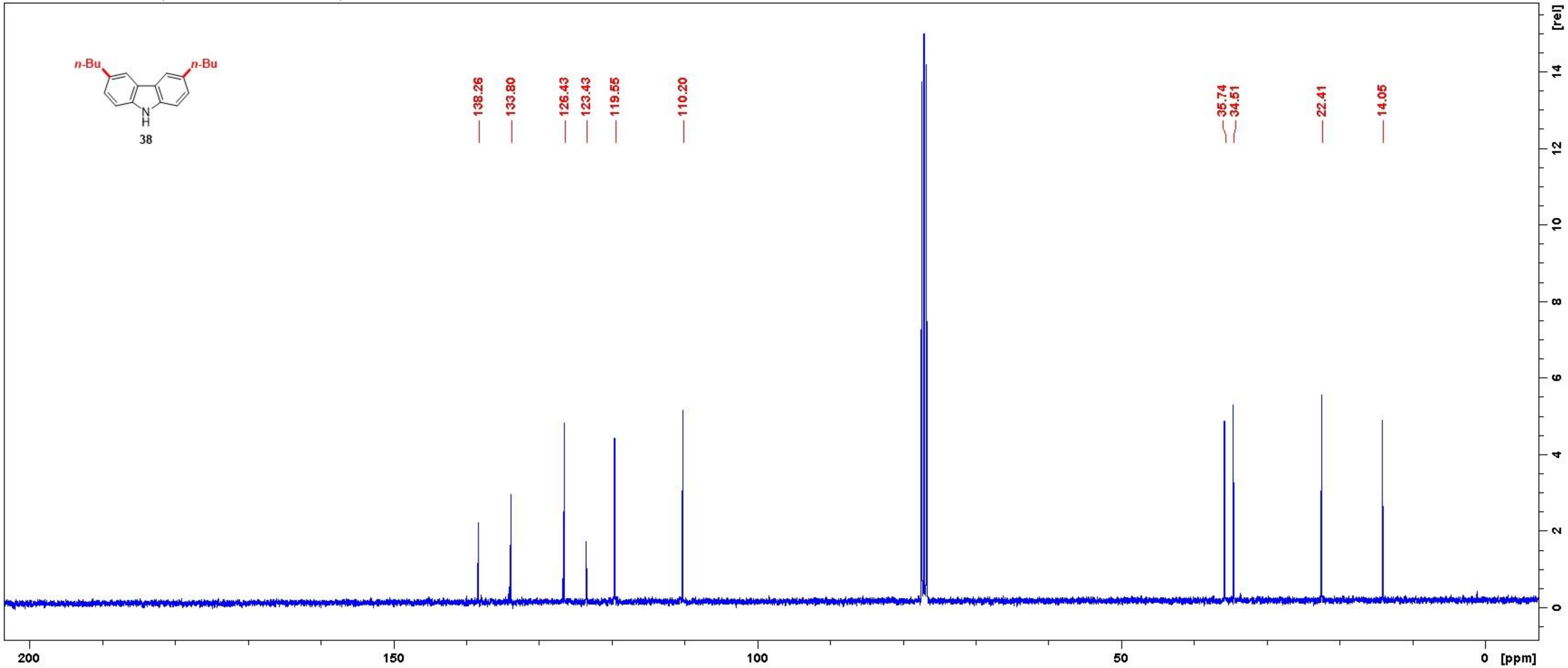

Supplement: Supplementary file 1 — jo3c01553_si_001.pdf [file jo3c01553_si_001.pdf]
